# Non-halogen aqueous electrolytes for four-electron iodine conversion

**DOI:** 10.1038/s41586-026-11282-0

**Published:** 2026-11-11

**Authors:** Ruwei Chen, Haobo Dong, Yuhang Dai, Jingyu Wang, Jie Chen, Jiyang Liu, Jianrui Feng, Yunpeng Zhong, Wei Zong, Wei Zhang, Hang Yang, Zhe Cui, Guanjie He

**Affiliations:** 1 Department of Chemistry, University College London, London, UK; 2 Department of Engineering Science, University of Oxford, Oxford, UK

**Keywords:** Batteries, Energy

## Abstract

Accessing reversible four-electron iodine redox chemistry in aqueous metal–iodine batteries offers a promising route towards high-energy aqueous energy storage, but has conventionally relied on highly concentrated, corrosive halogen-containing electrolytes to stabilize reactive hypervalent iodine cations through interhalogen complexation^1–9^. Here we show a kosmotropic anion–polar anion–nucleophilic species (Nuc) electrolyte framework that harnesses anionic chemistry to coordinate the interfacial microenvironment and molecular interactions, enabling reversible four-electron iodine conversion in halogen-free aqueous electrolytes. Polar anions establish a water-deficient, anion-enriched interfacial microenvironment, whereas kosmotropic anions regulate water–Nuc interactions to increase the availability of Nuc for stabilizing hypervalent iodine cations through Nuc–I^+^–Nuc halogen bonds. This electrolyte framework is validated across diverse Nuc and in both aqueous Zn–I_2_ and Al–I_2_ batteries. Moving beyond conventional interhalogen stabilization, these findings establish a distinct electrolyte chemistry framework and represent a mechanistic shift for reversible multi-electron iodine chemistry in aqueous environments.

## Abstract (web summary)

An electrolyte framework that harnesses anionic chemistry to coordinate the interfacial microenvironment and molecular interactions enables reversible four-electron iodine conversion in halogen-free aqueous electrolytes, and is validated in metal–iodine batteries.

## Main

The increasing integration of renewable energy calls for sustainable electrochemical energy storage systems beyond lithium-ion batteries^1^. Aqueous metal–iodine batteries offer a promising platform for large-scale energy storage by combining earth-abundant electrode materials, aqueous electrolytes and conversion-type iodine chemistry^2,3^. Unlike intercalation-type positive electrodes, iodine positive electrodes store charge by reversible valence-state changes, offering access to multi-electron redox chemistry with increased capacity and redox potential^4^. Most studies have focused on the two-electron I^−^/I^0^ redox couple, with a theoretical capacity of 211 mAh g^−1^ at a redox potential of 0.54 V versus the standard hydrogen electrode^10^. Extending iodine conversion to the successive I^−^/I^0^/I^+^ redox couples doubles the theoretical capacity to 422 mAh g^−1^ while introducing the I^0^/I^+^ redox process at a higher redox potential of 1.07 V versus the standard hydrogen electrode^11^. However, despite this potential, achieving reversible four-electron iodine conversion in aqueous environments remains a fundamental challenge^3,7^.

**Fig. 1:**
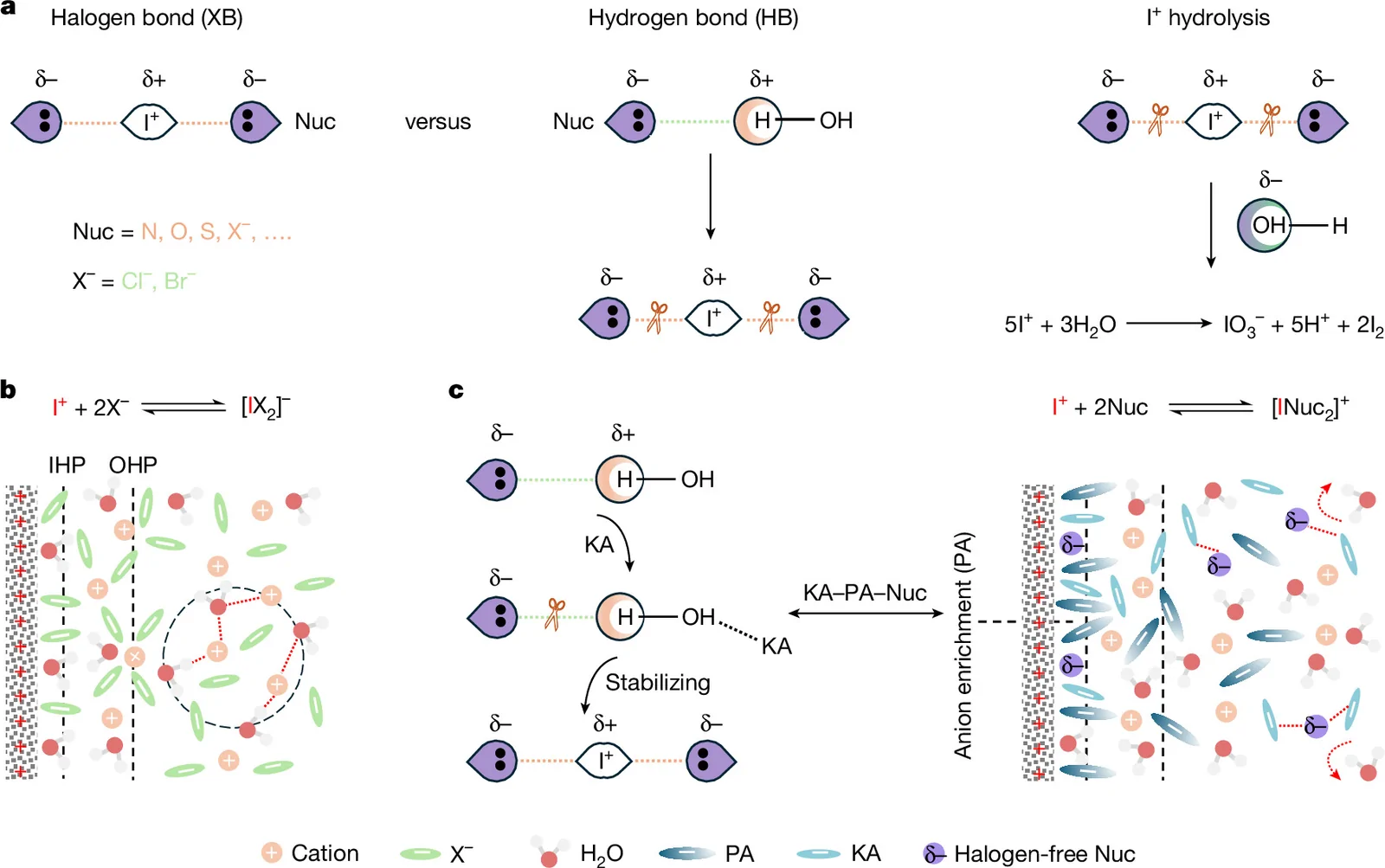
Comparisons of mechanistic frameworks for four-electron iodine conversion in aqueous electrolytes. **a**, The mechanism of the competition between halogen bond and hydrogen bond. **b**, The conventional interhalogen stabilization mechanism in highly concentrated, halogen-containing electrolytes. **c**, The KA–PA–Nuc framework levering anionic chemistry and halogen-bond stabilization in halogen-free electrolytes. IHP, inner Helmholtz plane; OHP, outer Helmholtz plane.

The crux of iodine conversion chemistry lies in interfacial reactions, where electrolyte composition and interfacial microenvironment govern reaction pathways. Electron-deficient I^+^ is strongly electrophilic and susceptible to nucleophilic attack by water, resulting in hydrolysis and poor reversibility in aqueous environments^9,12^. Previous studies have achieved four-electron iodine conversion in aqueous Zn–I_2_ and Al–I_2_ batteries by stabilizing reactive I^+^ through interhalogen complexes (for example, ICl_2_ or IBr_2_) in highly concentrated chloride- or bromide-containing electrolytes^5,6,13^. In these systems, electrophilic I^+^ coordinates with two nucleophilic halide ions (Cl^−^ or Br^−^) through non-covalent interactions^8,14^. However, at water-enriched interfaces, hydration of nucleophilic species (Nuc) competes with these interactions and destabilizes I^+^ complexes (Fig. 1a). Highly concentrated, halogen-containing electrolytes mitigate this competition by reducing water activity and increasing halide availability, but their high salt contents and corrosivity constrain broader implementation (Fig. 1b and Supplementary Fig. 1), necessitating new electrolyte chemistry and a mechanistic framework that move beyond reliance on halogen-containing electrolytes and conventional interhalogen stabilization^5,8^.

Given the competition between I^+^ stabilization and hydration at water-enriched interfaces, overcoming this challenge requires simultaneous regulation of the interfacial environment and water–Nuc interactions. Anionic chemistry offers a powerful lever to coordinate these interfacial and molecular interactions^15–18^. Herein we establish a kosmotropic anion (KA)–polar anion (PA)–Nuc electrolyte framework for reversible I^−^/I^0^/I^+^ conversion in halogen-free aqueous electrolytes (Fig. 1c). Systematic experiments and theoretical calculations reveal how the synergistic effects of PAs and KAs coordinate the interfacial microenvironment and molecular interactions, thereby stabilizing interfacial electrochemical behaviour and promoting reversible four-electron iodine conversion. The applicability of this electrolyte framework is demonstrated across four halogen-free Nuc (urea, acetamide, pyridine and nicotinamide) and in both aqueous Zn–I_2_ and Al–I_2_ batteries (Supplementary Fig. 2 and Supplementary Tables 1 and 2). Beyond establishing a halogen-free electrolyte chemistry, this work reveals how electrolyte chemistry governs iodine conversion across interfacial, molecular and electrochemical scales, providing a mechanistic basis for reversible multi-electron iodine chemistry in aqueous environments.

## Halogen-free electrolyte framework

**Fig. 2:**
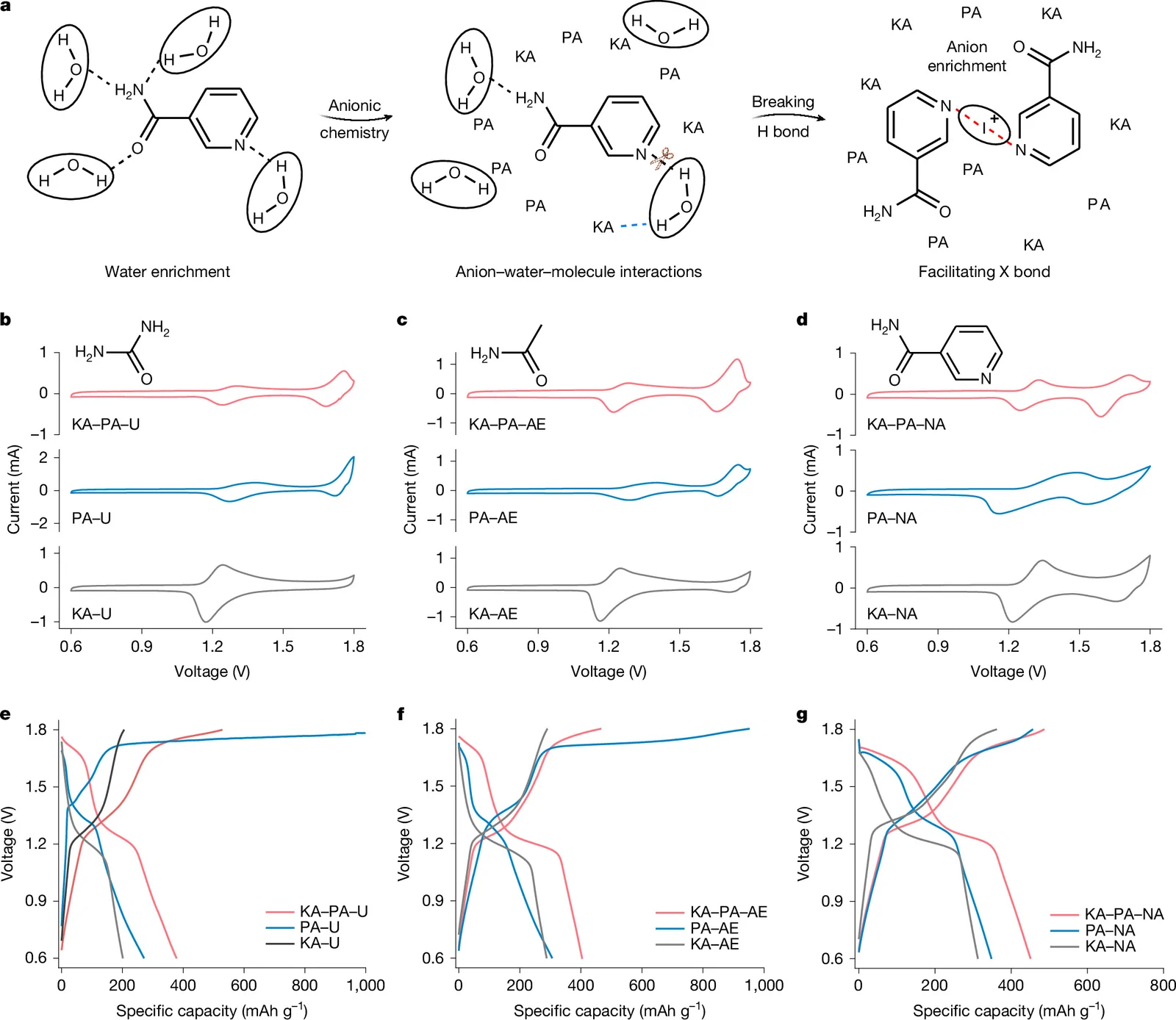
Demonstration of the proposed KA–PA–Nuc electrolyte framework. **a**, Molecular interplay governed by anionic chemistry and halogen bonding. **b**, Cyclic voltammetry profiles of iodine conversion reaction using urea as Nuc at 0.6 mV s^−1^. **c**, Cyclic voltammetry profiles using acetamide as Nuc at 0.6 mV s^−1^. **d**, Cyclic voltammetry profiles using nicotinamide as Nuc at 0.6 mV s^−1^. **e**–**g**, Corresponding GCD profiles for **b**–**d** at 1 A g^−1^, respectively. All electrochemical measurements were performed in Zn–I_2_ coin cells with an iodine mass loading of 2.2 mg cm^−2^. Source data

Stabilizing I^+^ against hydrolysis is essential for reversible four-electron iodine conversion in aqueous electrolytes. Previous studies have relied on highly concentrated, corrosive halogen-containing electrolytes for interhalogen stabilization^19,20^, whereas more recent studies have explored specific additive-halogen nucleophilic substitution reactions^21,22^. These approaches primarily address I^+^ stabilization through specific molecular interactions, whereas how electrolyte chemistry collectively regulates the interfacial microenvironment, molecular stabilization and the resulting iodine conversion pathways remains less understood. This work establishes a mechanistic framework that integrates anionic chemistry with halogen-bond stabilization in halogen-free aqueous electrolytes. As illustrated in Fig. 2a, strong PAs establish a water-deficient, anion-enriched interface, whereas KAs weaken Nuc–water hydrogen bonding to facilitate halogen-bond stabilization of I^+^. These complementary effects underpin the halogen-free KA–PA–Nuc electrolyte framework, whose composition was subsequently optimized (Supplementary Figs. 3 and 4) and exhibits sufficient electrochemical stability (Supplementary Fig. 5) for four-electron iodine conversion.

The KA–PA–Nuc electrolyte framework was validated in aqueous Zn–I_2_ batteries across four different Nuc (Supplementary Fig. 6). Electron-deficient I^+^ can interact with two electron-rich Nuc molecules to form linear Nuc–I^+^–Nuc halogen bonds. These halogen bonds provide the molecular basis for I^+^ stabilization, as supported by Nuc-free controls, nuclear magnetic resonance (NMR) spectroscopy and theoretical calculations^23^ (Supplementary Figs. 7–11). Using urea (U) as a representative Nuc, KA–U exhibits only the I^−^/I^0^ redox process, whereas PA–U enables further I^0^/I^+^ oxidation but with poor reversibility (Fig. 2b,e). The excessive capacity at the second charge plateau is attributed to irreversible iodine oxidation coupled with parasitic water oxidation (Supplementary Figs. 12 and 13). Combining KA and PA in KA–PA–U produces two reversible redox peaks and corresponding charge–discharge plateaus, demonstrating their synergistic role in enabling reversible I^−^/I^0^/I^+^ conversion (Supplementary Fig. 14). Replacing U with acetamide (AE), nicotinamide (NA) or pyridine (PD) shows the same phenomenon, with reversible I^−^/I^0^/I^+^ conversion achieved only in the KA–PA–Nuc electrolytes, including under identical ionic strength conditions, indicating the broad applicability of this electrolyte framework (Fig. 2c,d,f,g and Supplementary Figs. 15–18).

## The role of polar anions

**Fig. 3:**
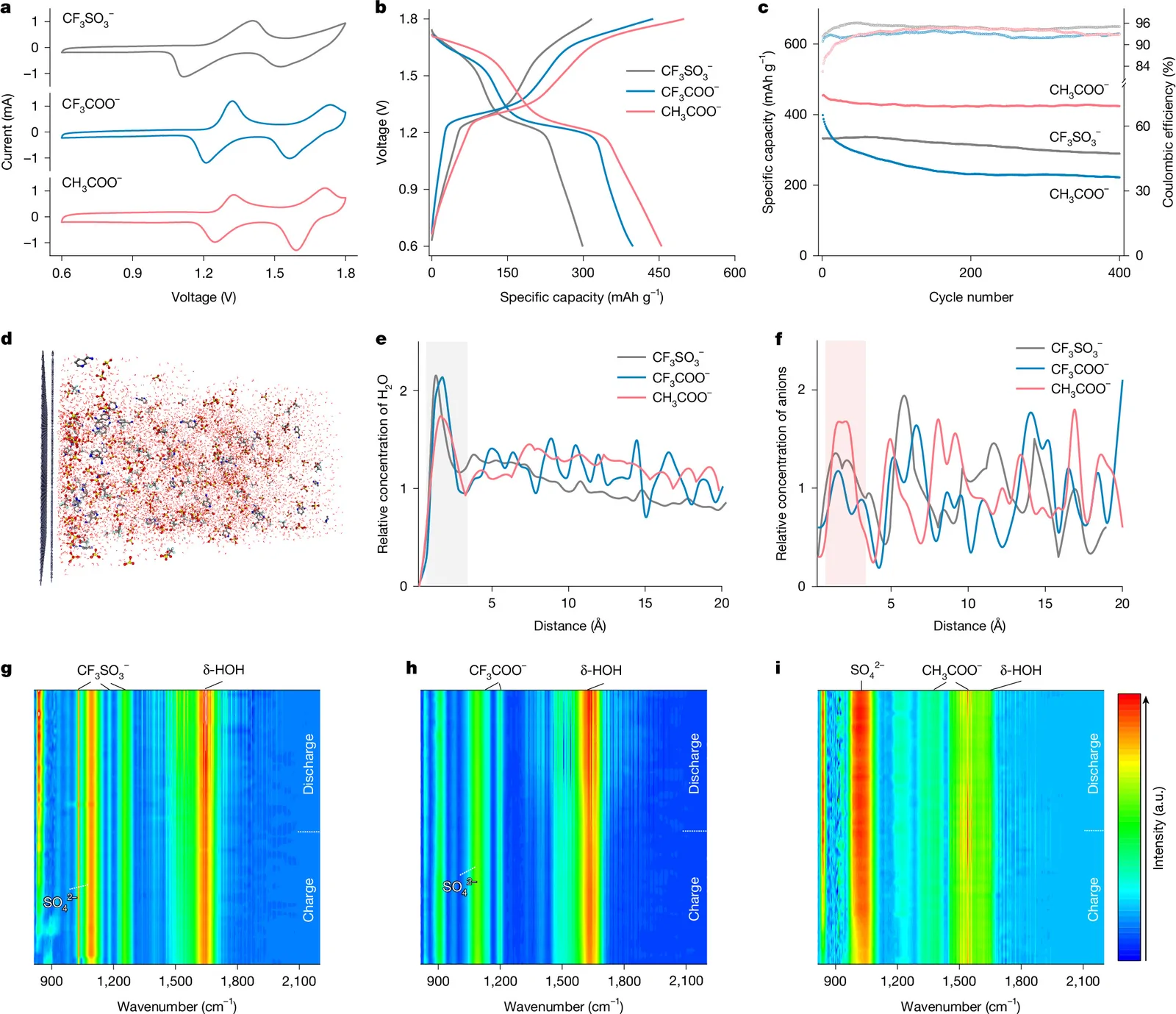
Critical role of polar anions. **a**, Cyclic voltammetry profiles in electrolytes with different anions. **b**, GCD profiles in electrolytes with different anions. The rising and falling voltage curves correspond to charging and discharging processes, respectively. **c**, Cycling stability in electrolytes with different anions, with upper and lower curves representing Coulombic efficiency and specific capacity, respectively. Electrochemical measurements were performed in Zn–I_2_ coin cells with an iodine mass loading of 2.2 mg cm^−2^. **d**, Schematic snapshot of the interfacial structure in the electrolyte with CH_3_COO^−^. **e**, Corresponding interfacial cumulated density profiles of H_2_O in electrolytes with different anions, with the grey shaded region highlighting interfacial variations. **f**, Corresponding interfacial cumulated density profiles of anions in electrolytes with different anions, with the pink shaded region highlighting interfacial variations. **g**–**i**, In situ ATR-IR spectra in the electrolytes with CF_3_SO_3_^−^ (**g**), CF_3_COO^−^ (**h**) and CH_3_COO^−^ (**i**). Source data

To isolate the role of PAs in the KA–PA–Nuc electrolyte framework, SO_4_^2−^ was maintained as the KA, whereas structurally related CF_3_SO_3_^−^, CF_3_COO^−^ or CH_3_COO^−^ was introduced as the second anion. Density-functional-theory calculations reveal a polarity sequence of CH_3_COO^−^ > CF_3_COO^−^ > CF_3_SO_3_^−^ (Supplementary Fig. 19), providing a basis for examining how anion polarity influences interfacial behaviours^24,25^. Molecular-dynamics simulations show similar water-enriched bulk environments across the three electrolytes (Supplementary Figs. 20–22), but distinct interfacial structures (Fig. 3d). CH_3_COO^−^ reduces the interfacial water density while increasing anion enrichment, whereas CF_3_COO^−^ and CF_3_SO_3_^−^ retain high interfacial water intensity (Fig. 3e,f). In situ attenuated total reflection infrared (ATR-IR) spectroscopy further supports this interfacial redistribution. The δ-HOH vibration at 1,640 cm^−1^ remains pronounced with CF_3_SO_3_^−^ or CF_3_COO^−^ (Fig. 3g,h and Supplementary Fig. 23), but is suppressed with CH_3_COO^−^ alongside pronounced anion-associated signals^26–29^ (Fig. 3i). Notably, highly concentrated ZnCl_2_ retains a water-enriched interface despite its water-deficient bulk environment (Supplementary Figs. 24 and 25). Differential capacitance measurements indicate stronger interfacial adsorption of CH_3_COO^−^ than of CF_3_COO^−^ and CF_3_SO_3_^−^ (Supplementary Figs. 26 and 27), linking preferential adsorption of strongly PAs to the formation of a water-deficient, anion-enriched interfacial microenvironment^30^.

These polarity-dependent interfacial structures lead to distinct iodine conversion behaviour. CF_3_SO_3_^−^ shows a negligible I^0^/I^+^ process, whereas CF_3_COO^−^ enables I^0^/I^+^ oxidation but with limited reversibility. By contrast, CH_3_COO^−^ supports reversible I^−^/I^0^/I^+^ conversion with improved stability and capacity (Fig. 3a–c and Supplementary Fig. 28). In situ ultraviolet–visible spectra and micro-computed-tomography analyses further link these differences to iodine species distribution, showing that CH_3_COO^−^ confines iodine species within the electrode and suppresses their migration into the bulk electrolyte (Supplementary Figs. 29 and 30). Together, these results establish a mechanistic relationship between anion polarity and the resulting water-deficient, anion-enriched interfacial microenvironment that governs the reversibility of four-electron iodine conversion.

## The role of kosmotropic anions

**Fig. 4:**
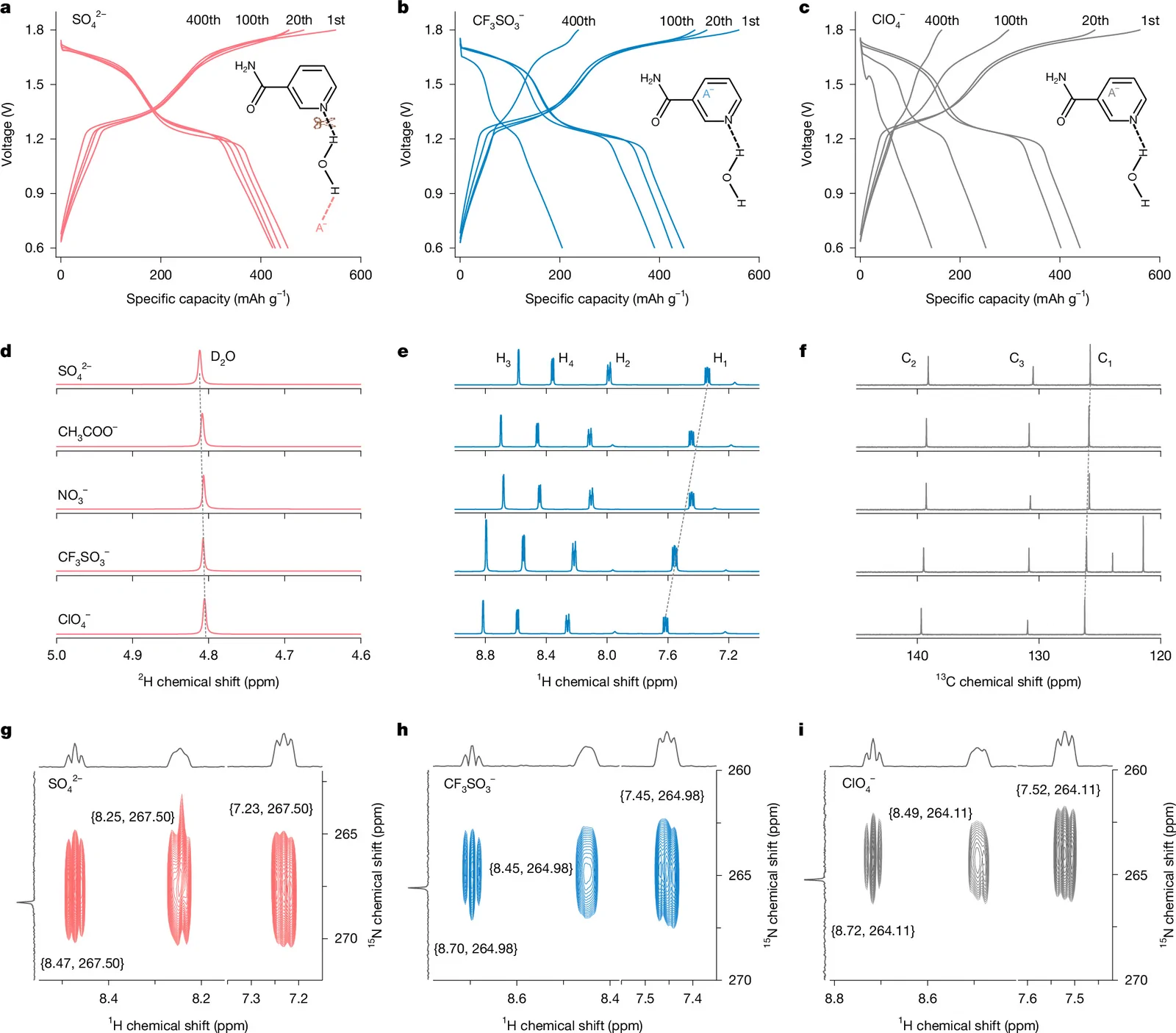
Critical role of kosmotropic anions. **a**, GCD profiles in the electrolyte containing SO_4_^2−^ at different cycles. **b**, GCD profiles in the electrolyte containing CF_3_SO_3_^−^ at different cycles. **c**, GCD profiles in the electrolyte containing ClO_4_^−^ at different cycles. Electrochemical measurements were performed in Zn–I_2_ coin cells with an iodine mass loading of 2.2 mg cm^−2^. **d**, ^2^H NMR spectra of electrolytes containing different anions. **e**, ^1^H NMR spectra of electrolytes containing different anions. **f**, ^13^C NMR spectra of electrolytes containing different anions. **g**, Two-dimensional ^1^H–^15^N HMBC NMR spectrum of the electrolyte containing SO_4_^2−^. **h**, Two-dimensional ^1^H–^15^N HMBC NMR spectrum of the electrolyte containing CF_3_SO_3_^−^.** i**, Two-dimensional ^1^H–^15^N HMBC NMR spectrum of the electrolyte containing ClO_4_^−^. Source data

Building on the understanding of PAs, the critical role of KAs in the KA–PA–Nuc framework was further explored. Nuc stabilize I^+^ through electron-rich nitrogen or oxygen centres, which also interact with water through hydrogen bonding and therefore compete with halogen-bond formation (Supplementary Fig. 31). Inspired by anion-specific effects on water–solute interactions, SO_4_^2−^, CH_3_COO^−^, NO_3_^−^, CF_3_SO_3_^−^ and ClO_4_^−^ were compared in the same PA–Nuc baseline^31–34^ (Supplementary Fig. 32). Although these anions have little effect on the initial capacity, they produce different cycling stability (Supplementary Fig. 33). SO_4_^2−^ maintains nearly overlapping galvanostatic charge–discharge (GCD) profiles, whereas CH_3_COO^−^ and CF_3_SO_3_^−^ show intermediate cycling stability and ClO_4_^−^ undergoes rapid deterioration (Fig. 4a–c and Supplementary Fig. 34), giving a stability sequence of SO_4_^2−^ > CH_3_COO^−^ > CF_3_SO_3_^−^ > ClO_4_^−^. NO_3_^−^ deviates from this sequence owing to its limited electrochemical stability within the applied voltage range^35^ (Supplementary Fig. 35a,b).

NMR spectroscopy and molecular-dynamics simulations reveal the molecular origin of these electrochemical differences. The ^2^H chemical shift follows SO_4_^2−^ > CH_3_COO^−^ > NO_3_^−^ > CF_3_SO_3_^−^ > ClO_4_^−^ (Fig. 4d), indicating progressively stronger anion-induced water polarization and restructuring of the aqueous hydrogen-bond network^36,37^. These differences in water–anion coordination translate into distinct water–Nuc interactions. The corresponding ^15^N chemical shifts, together with the inverse trends observed in ^1^H and ^13^C chemical shifts, indicate that KAs such as SO_4_^2−^ polarize hydration water and thereby weaken Nuc–water hydrogen bonding, whereas chaotropic anions such as ClO_4_^−^ interact directly with the Nuc backbone and facilitate Nuc hydration^32^ (Fig. 4e,f, and Supplementary Figs. 32 and 36–38). Nuc solubility measurements, two-dimensional ^1^H–^15^N heteronuclear multiple bond correlation (HMBC) NMR spectra, and calculated Nuc–water distances and hydrogen-bond populations further support this interpretation (Fig. 4g–i, and Supplementary Figs. 35c, 39 and 40). By weakening Nuc–water interactions, SO_4_^2−^ increases the availability of Nuc for halogen-bond stabilization of I^+^, thereby promoting reversible iodine conversion. These results reveal how anionic chemistry regulates molecular water–Nuc interactions to govern the reversibility of four-electron iodine conversion.

**Extended Data Fig. 1:**
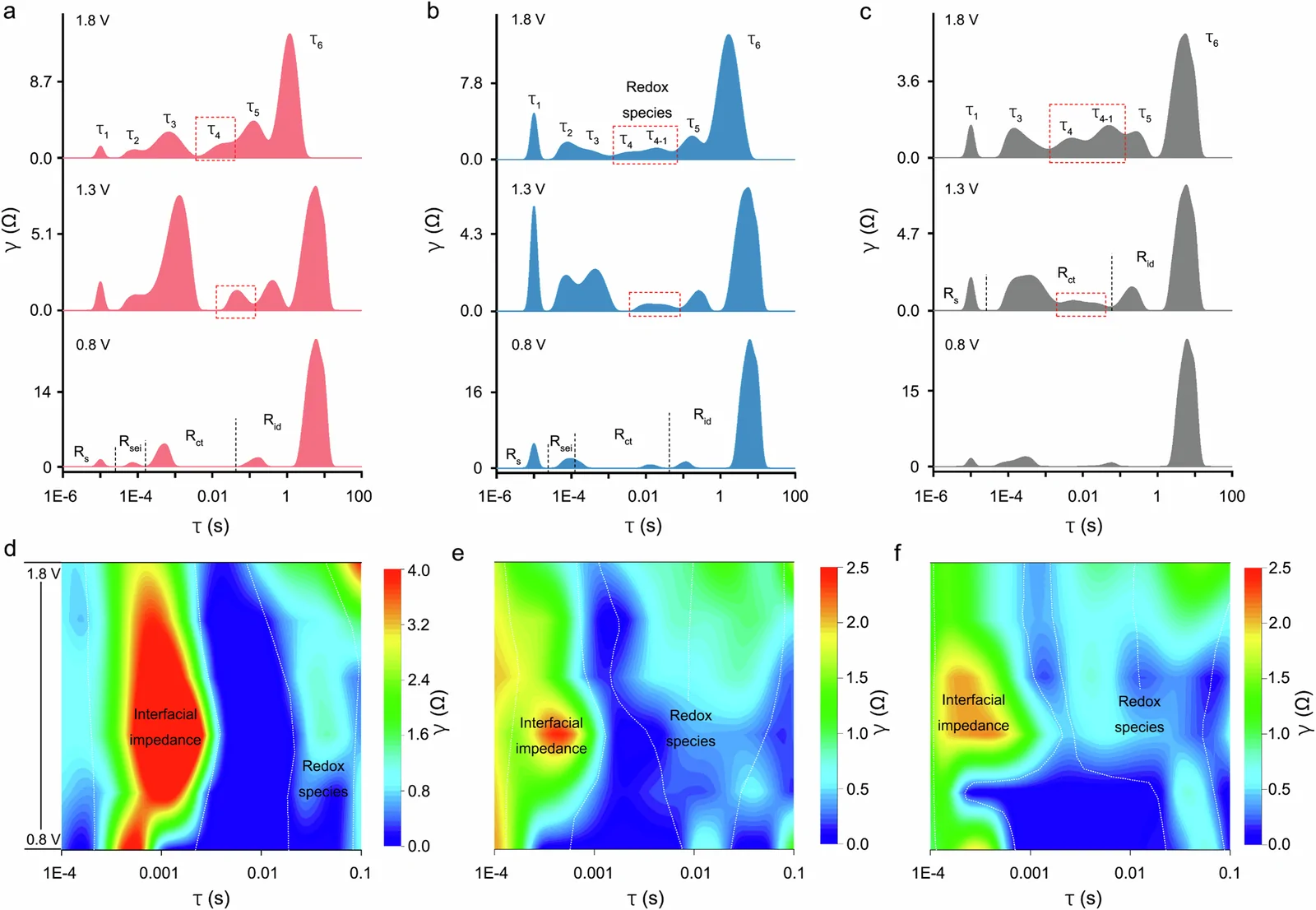
Electrolyte-dependent interfacial electrochemical behaviour during iodine conversion. a, DRT profiles of the iodine positive electrode at different states in the KA (SO_4_^2−^)-PA (CH_3_COO^−^)-Nuc electrolyte. b, DRT profiles of the iodine positive electrode at different states in the CF_3_SO_3_^−^-PA (CH_3_COO^−^)-Nuc electrolyte without KA. c, DRT profiles of the iodine positive electrode at different states in the KA (SO_4_^2−^)-CF_3_SO_3_^−^-Nuc electrolyte without PA. d-f, Corresponding DRT contour plots for the KA (SO_4_^2−^)-PA (CH_3_COO^−^)-Nuc, CF_3_SO_3_^−^-PA (CH_3_COO^−^)-Nuc, and KA (SO_4_^2−^)-CF_3_SO_3_^−^-Nuc electrolytes, respectively. Measurements were performed using the specialized three-electrode configuration shown in Supplementary Fig. 41a. The iodine loading on the positive electrode was 2.2 mg cm^−2^.

Having established the complementary roles of PA and KA in interfacial and molecular regulation, we next examined how these effects manifest in the electrochemical processes governing iodine conversion. In situ electrochemical impedance spectroscopy (EIS) combined with distribution of relaxation times (DRT) analysis was used to decouple the complex impedance response and identify individual electrochemical processes^38–40^ (Supplementary Figs. 41–43). In the absence of either KA or PA, the DRT responses evolve distinctly with additional competing interfacial processes, whereas their combined presence maintains stable interfacial electrochemical behaviour throughout iodine conversion^41,42^ (Extended Data Fig. 1). Rate-dependent analysis identifies charge transfer, rather than ion transport, as the dominant kinetic contribution to iodine conversion in the KA–PA–Nuc electrolyte (Supplementary Figs. 44–50). In situ Raman spectroscopy further links these electrochemical processes to iodine chemistry, revealing the reversible evolution of the Nuc–I^+^–Nuc species during cycling (Supplementary Figs. 51 and 52).

**Extended Data Fig. 2:**
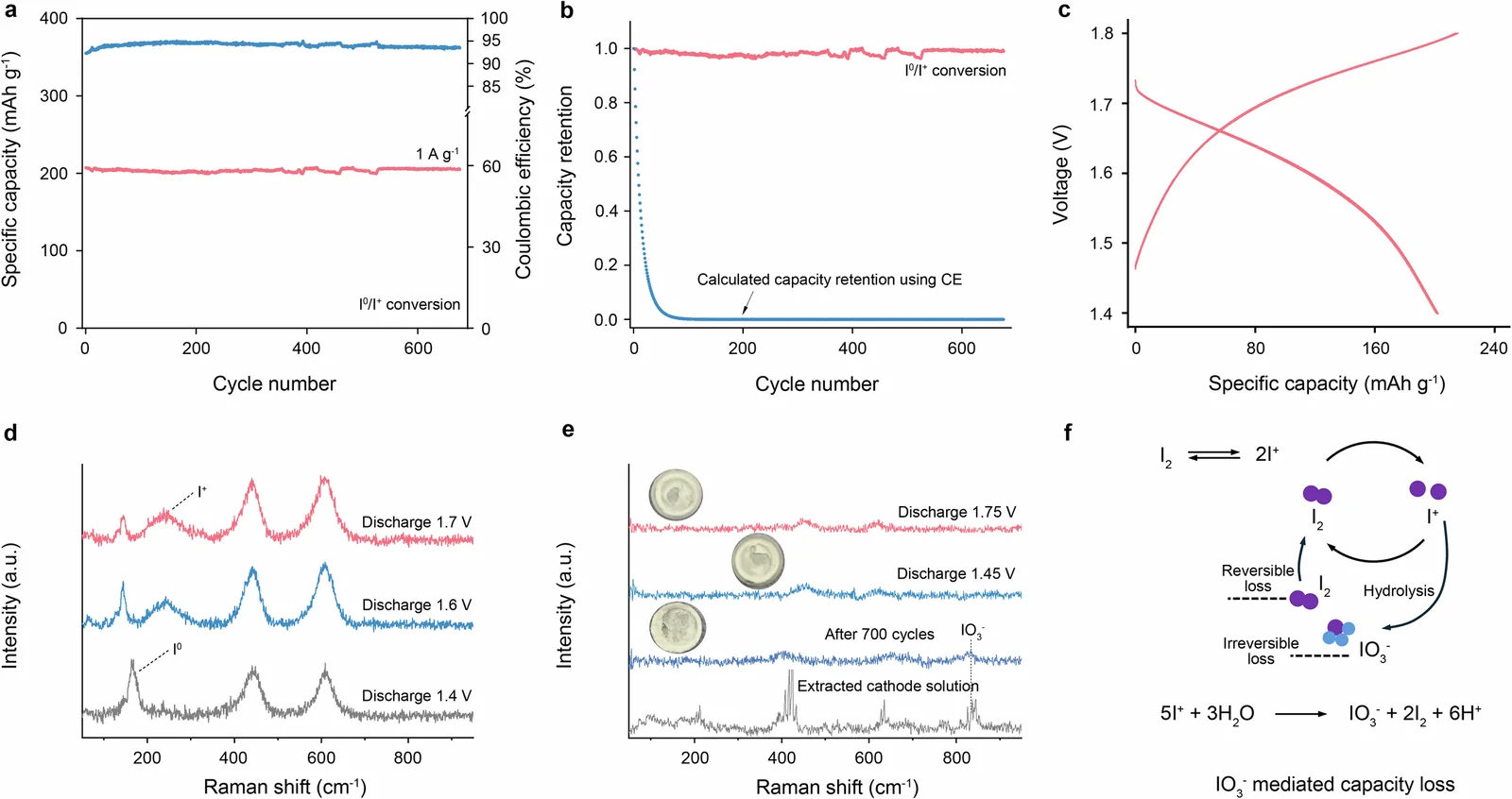
Mechanistic analysis of the decoupled I^0^/I^+^ conversion in the KA-PA-Nuc electrolyte. a, Cycling performance of the I^0^/I^+^ conversion at 1 A g^−1^. b, Comparison of the measured capacity retention with that calculated from CE values. c, GCD profiles of the I^0^/I^+^ conversion. d, In situ Raman spectra of the iodine positive electrode at different discharge states. e, Ex situ Raman spectra of the cycled separators and extracted iodine positive electrode solution; inset images show the corresponding separators. f, Schematic illustration of the I^0^/I^+^ conversion and associated degradation pathways. Electrochemical measurements in a-c were performed using Zn-I_2_ coin cells, whereas the in situ Raman measurements in d were conducted using the dedicated in situ Zn-I_2_ cell configuration described in the Methods. Ex situ characterization in e was performed using Zn-I_2_ Swagelok cells to facilitate cell disassembly and post-cycling sample collection. The iodine mass loading was 2.2 mg cm^−2^ for all cell configurations.

**Extended Data Fig. 3:**
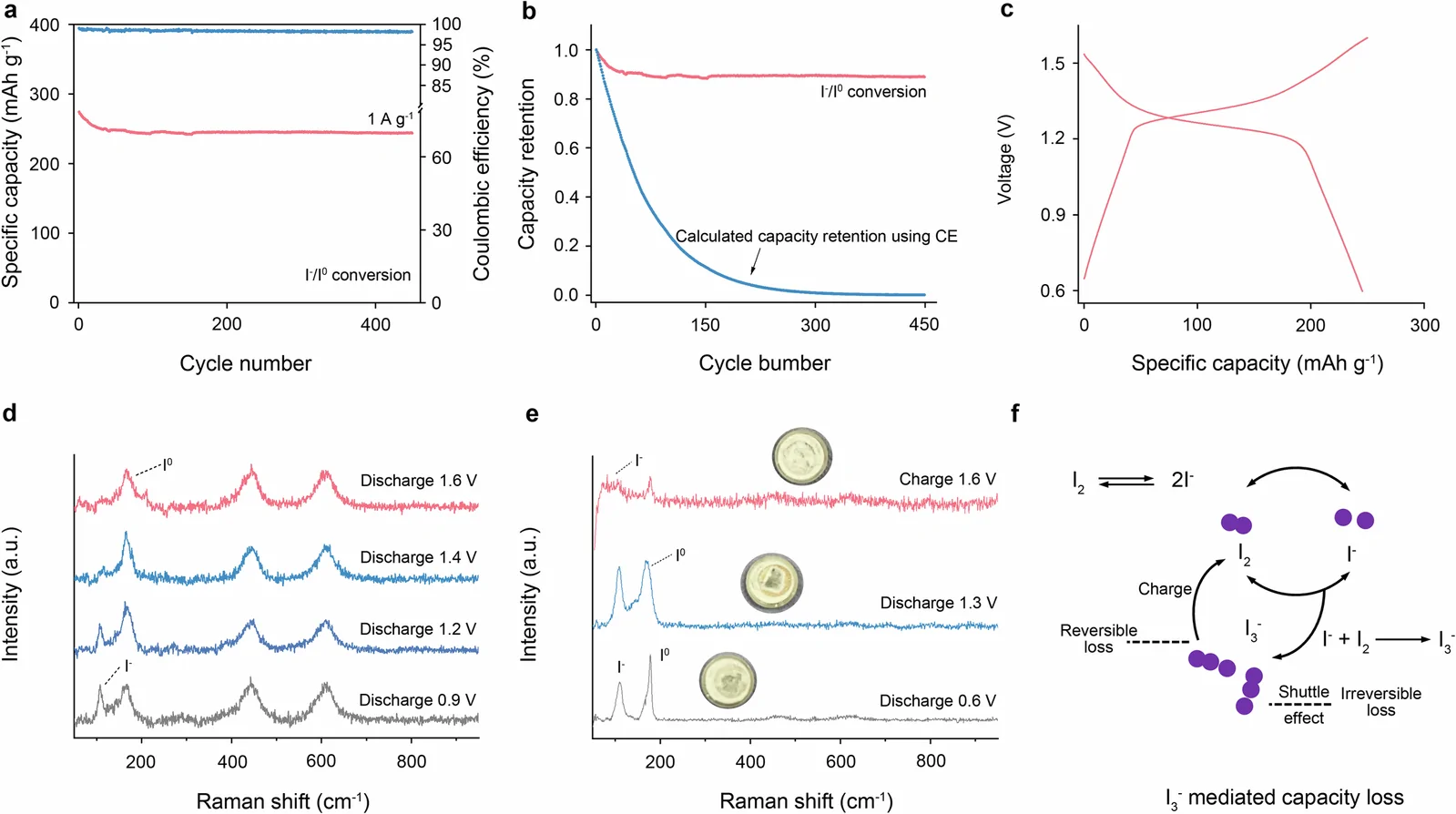
Mechanistic analysis of the decoupled I^−^/I^0^ conversion in the KA-PA-Nuc electrolyte. a, Cycling performance of the I^−^/I^0^ conversion at 1 A g^−1^. b, Comparison of the measured capacity retention with that calculated from CE values. c, GCD profiles of the I^−^/I^0^ conversion. d, In situ Raman spectra of the iodine positive electrode at different discharge states. e, Ex situ Raman spectra of the cycled separators; inset images show the corresponding separators. f, Schematic illustration of the I^−^/I^0^ conversion and associated degradation pathways. Electrochemical measurements in a-c were performed using Zn-I_2_ coin cells, whereas the in situ Raman measurements in d were conducted using the dedicated in situ Zn-I_2_ cell configuration described in the Methods. Ex situ characterization in e was performed using Zn-I_2_ Swagelok cells to facilitate cell disassembly and post-cycling sample collection. The iodine mass loading was 2.2 mg cm^−2^ for all cell configurations.

**Extended Data Fig. 4:**
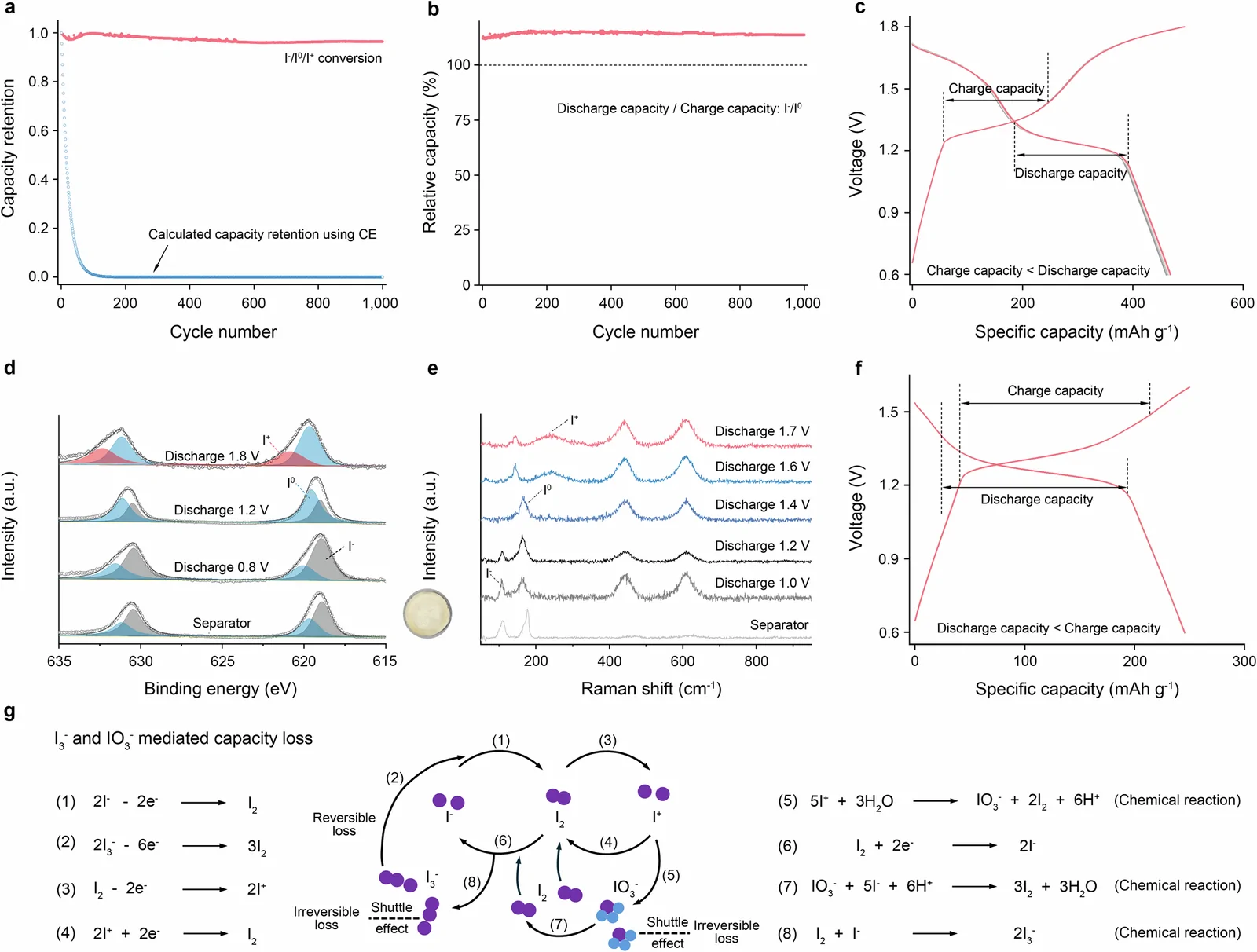
Mechanistic analysis of the coupled I^−^/I^0^/I^+^ conversion in the KA-PA-Nuc electrolyte. a, Comparison of the measured capacity retention with that calculated from CE values. b, Ratio of the discharge to charge capacities of the I^−^/I^0^ plateau during coupled I^−^/I^0^/I^+^ conversion. c, GCD profiles of the coupled I^−^/I^0^/I^+^ conversion, showing that the discharge capacity for the I^−^/I^0^ process exceeds the corresponding charge capacity. d, X-ray photoelectron spectroscopy (XPS) spectra of the cycled separator and iodine positive electrodes at different discharge states. e, Raman spectra of the cycled separator and iodine positive electrodes at different discharge states. f, GCD profiles of the decoupled I^−^/I^0^ conversion, showing that the discharge plateau is lower than the corresponding charge plateau. g, Schematic illustration of the coupled I^−^/I^0^/I^+^ conversion and associated degradation pathways. Electrochemical measurements were performed using Zn-I_2_ coin cells, whereas Zn-I_2_ Swagelok cells were used for the ex situ characterization to facilitate cell disassembly and post-cycling sample collection. The iodine mass loading was 2.2 mg cm^−2^ for all cell configurations.

The KA–PA–Nuc framework supports four-electron iodine conversion across different operating conditions and cell configurations. Zn–I_2_ cells retain 96% of their capacity after 1,000 cycles at 1 A g^−1^ and 94% after 2,000 cycles at 2 A g^−1^, while sustaining four-electron conversion at iodine loadings up to 12.2 mg cm^−2^ (Supplementary Figs. 53–55, 58a and 59). This electrolyte framework is also compatible with different current collectors and pouch-cell configurations and extends to aqueous Al–I_2_ cells using Al–Zn alloy negative electrodes (Supplementary Figs. 56–58b,c, 60 and 61). A techno-economic analysis under standardized modelling assumptions further illustrates its theoretical cost-reduction potential by avoiding highly concentrated, halogen-containing electrolytes and costly current collectors (Supplementary Fig. 58d–f and Supplementary Tables 3 and 4). Mechanistic and post-mortem analyses further reveal that the apparent low Coulombic efficiency and capacity fading involve coupled reversible and irreversible iodine inventory loss, with the latter becoming more pronounced at increased iodine loading (Supplementary Figs. 62–67 and Extended Data Figs. 2–4). Together, these results clarify the iodine conversion pathways while defining the applicability and remaining limitations of the KA–PA–Nuc framework, showing that sustained reversible four-electron iodine conversion requires not only stabilization of hypervalent iodine species but also broader management of the entire iodine inventory.

## Methods

All chemicals were used as received without further purification unless otherwise stated. The manufacturer and purity of each material are specified at its first occurrence in Methods and are not repeated thereafter unless a different source or purity was used.

### Preparation of electrolytes

The KA–PA–Nuc electrolytes were prepared by dissolving KA, PA and Nuc into deionized water at room temperature. For aqueous Zn–I_2_ batteries, KA and PA were 1.5 M ZnSO_4_ (>99%, Sigma-Aldrich) and 0.5 M Zn (CH_3_COO)_2_ (>98%, Sigma-Aldrich), respectively. To demonstrate the broad applicability of the KA–PA–Nuc framework, various Nuc were used, including 2 M urea (>99.5%, Sigma-Aldrich), 2 M acetamide (>99%, Sigma-Aldrich), 0.2 M pyridine (>99%, Sigma-Aldrich) or 0.4 M nicotinamide (>99.5%, Sigma-Aldrich). To explore the role of PAs, a baseline electrolyte containing 1.5 M ZnSO_4_ and 0.4 M nicotinamide was prepared. Zn(CH_3_COO)_2_, Zn(CF_3_COO)_2_ (purity not specified, Sigma-Aldrich) or Zn(CF_3_SO_3_)_2_ (>98%, Sigma-Aldrich) was then added at a concentration of 0.5 M to obtain electrolytes containing different PAs. To evaluate the role of KAs, a baseline electrolyte containing 0.5 M Zn(CH_3_COO)_2_ and 0.4 M nicotinamide was prepared. ZnSO_4_, Zn(NO_3_)_2_ (>99%, Sigma-Aldrich), Zn(CF_3_SO_3_)_2_ or Zn(ClO_4_)_2_ (purity not specified, Sigma-Aldrich) was then added at a concentration of 1.5 M for comparative analysis. To compare electrolytes performance under a standardized ionic strength condition, the ionic strength of each electrolyte was calculated according to the equation:

$${I}_{i}=\frac{1}{2}\mathop{\sum }\limits_{i=1}^{n}{C}_{i}{Z}_{i}^{2}$$

where *C*_*i*_ and *Z*_*i*_ represent the molar concentration and charge number of ion *i*, respectively. For aqueous Al–I_2_ batteries, the KA–PA–Nuc electrolyte was prepared by dissolving 1.5 M Al_2_(SO_4_)_3_ (>99%, Sigma-Aldrich), 0.5 Mg (CH_3_COO)_2_ (>99%, Sigma-Aldrich) and 0.5 M nicotinamide into deionized water.

### Preparation of electrodes

Iodine-activated carbon (I_2_–AC) composites were prepared using an evaporation–adsorption method. In brief, 0.5 g iodine (>99.5%, Thermo Scientific Acros) and 0.5 g activated carbon (AC; YP-80F, purity not specified, Kuraray) were manually ground in an agate mortar with a pestle for 10 min under ambient conditions. The resulting mixture was sealed in a Teflon reactor and heated at 120 °C for 6 h. For the preparation of iodine positive electrodes using titanium (80 mesh, >99.5%, Hebei Conway Metal Products) or stainless steel mesh (80 mesh, 316L, Hebei Conway Metal Products) as the current collector, I_2_–AC, polytetrafluoroethylene (PTFE, 60 wt% dispersion H_2_O, Sigma-Aldrich) and Super P (purity not specified, Canrd) were combined at a mass ratio of 8:1:1. For each batch, 40 mg I_2_–AC, 5 mg Super P and 5 mg PTFE (calculated on a dry-PTFE basis) were manually mixed with deionized water using an agate mortar and pestle under ambient conditions. The resulting mixture was rolled into a self-standing film using a roller press (Etelux) and subsequently dried in an oven at 60 °C for 3 h in air. The areal iodine loading was controlled by adjusting the thickness of the composite film. The dried film was then pressed onto the titanium or stainless steel mesh using the roller press. The resulting electrodes were punched into 12 mm disks using a manual disk punch (T07, ‌Hefei Kejing Material Technology).

For iodine positive electrodes using carbon paper (TGP-H-060, Toray) as the current collector, I_2_–AC, polyvinylidene fluoride (PVDF, weight-average molecular weight, *M*_w_ ≈ 534,000 g mol^−1^ by gel permeation chromatography, powder, Sigma-Aldrich) and Super P were combined at a mass ratio of 8:1:1. For each batch, 160 mg I_2_–AC, 20 mg PVDF and 20 mg Super P were manually mixed with *N*-methyl-2-pyrrolidone (>99.5%, Thermo Scientific Acros) using an agate mortar and pestle in ambient conditions. The resulting slurry was manually and uniformly spread onto the carbon paper using a laboratory spatula. The coated electrodes were dried in a vacuum oven at 60 °C for 8 h and subsequently cut into 12-mm disks using the manual disk punch. Commercial Zn foils (70 µm, >99.99%, ‌Qinghe Haoxuan Metal Materials) were used as negative electrodes as received.

To assess the potential of the KA–PA–Nuc electrolyte framework in multi-electron aqueous Al–I_2_ batteries, a Ag/AgCl electrode in saturated KCl (Beijing Jingke Instrument Science Instrument) was used as the reference, and AC electrodes were prepared as the counter electrode. The measured potentials are reported directly versus the Ag/AgCl reference electrode. For the preparation of AC electrodes, AC, PTFE and Super P were combined at a mass ratio of 8:1:1. For each batch, 160 mg AC, 20 mg Super P and 20 mg PTFE (calculated on a dry-PTFE basis) were manually mixed with deionized water using an agate mortar and pestle at ambient conditions. The resulting mixture was rolled into a self-standing film using the roller press and subsequently dried in an oven at 60 °C for 3 h in air. The dried film was then pressed onto titanium mesh using the roller press. By adjusting the electrode thickness, the capacity of the AC electrode was controlled to be much higher than that of the iodine working electrode, ensuring its capability as the counter electrode for evaluating the electrochemical performance of the KA–PA–Nuc electrolyte. To further validate the applicability of the KA–PA–Nuc electrolyte framework in multi-electron aqueous Al–I_2_ batteries, Al–Zn alloys were fabricated as negative electrodes following a previous study^43^. In brief, symmetric cells were assembled using Zn foils (70 µm, 16 mm in diameter) as electrodes and 2 M Al (CF_3_SO_3_)_3_ (>99.9%, Sigma-Aldrich) as the electrolyte. During charging, Al^3+^ in the electrolyte deposited on and reacted with the metal Zn substrate, forming the Zn-supported Al–Zn alloys.

### Synthesis of halogen-bond complexes

Halogen-bond complexes were synthesized via a cation-exchange reaction using corresponding silver analogues, following a previously reported procedure with some modifications^44^. All operations were carried out under a standard Schlenk line. In a typical synthesis, 0.94 mmol of the corresponding silver salt (Ag_2_SO_4_, >99%, Sigma-Aldrich; AgCH_3_COO, >99%, Sigma-Aldrich; AgCF_3_COO, >98%, Sigma-Aldrich; AgCF_3_SO_3_, >99%, Sigma-Aldrich; AgNO_3_, >99%, Sigma-Aldrich; or AgClO_4_, >97%, Sigma-Aldrich) was added to a 20-ml vial equipped with a stir bar. The vial was sealed with a rubber septum and evacuated. Subsequently, 6.0 ml dry CH_2_Cl_2_ (purity not specified, VWR Chemicals) and 1.90 mmol pyridine (3.80 mmol for Ag_2_SO_4_) were added at room temperature under a nitrogen atmosphere. The mixture was stirred for 10 min to form the Ag(I)–pyridine *bis*-coordination complex. Next, 2.00 mmol of iodine (4.00 mmol for Ag_2_SO_4_) was added, and the reaction was allowed to proceed for an additional 20 min under continuous stirring. The supernatant was then carefully transferred to another vial via cannula filtration, followed by the addition of 15 ml dry *n*-hexane (>99%, Macron Fine Chemicals) to induce precipitation of the halogen-bond complex. The mixture was stored at −20 °C overnight to complete precipitation. Afterwards, the supernatant was removed using a syringe, and the resulting solid was washed with 20 ml dry *n*-hexane to remove any residual I_2_. Finally, the product was dried under vacuum on the Schlenk line.

### Materials characterizations

#### In situ ATR-IR measurements

In situ ATR-IR spectra were recorded by a Thermo Scientific Nicolet iS50 spectrometer equipped with a liquid nitrogen-cooled MCT-A detector. Gold-coated silicon plates were used as working electrodes, with Zn foils (70 µm, 10 mm in diameter) and glass fibre (GF/D, 12 mm in diameter, Whatman) used as reference electrodes and separators, respectively. To assess the critical role of the PA in the interfacial environment, 1.5 M KA electrolyte was maintained as the baseline, and 0.5 M Zn(CH_3_COO)_2_, 0.5 M Zn(CF_3_COO)_2_ or 0.5 M Zn(CF_3_SO_3_)_2_ was separately added. An electrolyte volume of 500 μl was used for each measurement. During tests, the assembled batteries were connected to a Gamry Interface 1000 potentiostat to run a cyclic voltammetry programme at a scan rate of 0.2 mV s^−1^ for a full charge and discharge cycle. All spectra were collected at a resolution of 4 cm^−1^, with 50 scans per measurement. Measurements were performed at an ambient laboratory temperature of approximately 18–25 °C.

#### In situ Raman measurements

Raman spectra were recorded using a Bruker Senterra II Raman spectrometer with a 532-nm excitation wavelength. The in situ Zn–I_2_ cell comprised an iodine positive electrode (12 mm in diameter, iodine mass loading 2.2 mg cm^−2^), a Zn negative electrode (70 µm, 12 mm in diameter) and a glass fibre (GF/D, 12 mm in diameter). An electrolyte volume of 200 μl was used. Raman spectra were collected during GCD cycling using an external Gamry Interface 1000 potentiostat. Measurements were performed at an ambient laboratory temperature of approximately 18–25 °C.

FTIR spectra of various electrolytes were obtained using an ATR-FTIR spectrometer (BRUKER, Platinum-ATR). Scanning electron microscopy characterization of electrodes was performed using a JEOL 7600 microscope. Ultraviolet–visible measurements were conducted on a Shimadzu UV-3600i Plus spectrophotometer. For in situ ultraviolet–visible measurements, a two-electrode Zn–I_2_ cuvette cell was connected to a Neware battery test system for GCD cycling. NMR analyses, including ^1^H, ^13^C and ^1^H–^15^N HMBC spectra, were carried out on a Bruker NMR instrument (Avance Neo 500). ^15^N spectra were collected using a Bruker NMR instrument (Avance Neo 700). Deuterium oxide (>99.9%, Fluorochem) was used as the solvent for electrolyte characterization, and dideuteromethylenechloride (>99.9%, Sigma-Aldrich) was used as the solvent for characterizing halogen-bond complexes. Micro-computed-tomography imaging was conducted using a Zeiss Xradia 620 Versa system with a ×4 objective, operating at a tube voltage of 80 kV and an exposure time of 8 s, achieving a voxel resolution of 0.8 μm. For each sample, a disk with a diameter of 2 mm was prepared to balance X-ray attenuation and representative sampling volume. The acquired computed-tomography datasets were subsequently processed and analysed using Avizo software.

### Electrochemical measurements

#### Coin-cell and Swagelok-cell configurations

Electrochemical studies of halogen-free electrolytes were performed using CR2025 coin cells, whereas those of halogen-containing electrolytes were performed using Swagelok cells. For CR2025 Zn–I_2_ coin cells, the iodine positive electrode, Zn negative electrode (70 µm) and separator (GF/D) had diameters of 12 mm, 16 mm and 19 mm, respectively. For Swagelok Zn–I_2_ cells, corresponding electrodes and separators had the same diameter of 12 mm. For CR2025 Al–I_2_ coin cells, the iodine positive electrode had a diameter of 12 mm, the Al–Zn alloy negative electrode had a diameter of 16 mm, and a GF/D separator with a diameter of 19 mm was used. An electrolyte volume of 100 μl was used for each cell. Coin cells were mechanically crimped at 50 kg cm^−2^ using a hydraulic crimping machine (MSK-110, Hefei Kejing Material Technology) during assembly. Swagelok cells were manually tightened during assembly.

#### Pouch-cell configuration

For the 1.1-Ah pouch cell, 2 double-side coated iodine positive electrodes (10 × 15 cm), and 3 Zn negative electrodes (70 µm, 10 × 15 cm) were stacked alternately. The areal iodine loading of positive electrodes is controlled at 10 mg cm^−2^. Four glass fibre separators (GF/D, 10.1 × 15.1 cm) were utilized, and 1.5 ml electrolyte was added to each separator. The electrolyte consisted of 1.5 M ZnSO_4_, 0.5 M Zn(CH_3_COO)_2_ and 0.4 M nicotinamide. Titanium foils were connected to electrodes as tabs. After lamination, the assembled battery was encapsulated in an aluminium–plastic film and mechanically constrained using a customized model during electrochemical measurements. The applied pressure was not specifically quantified.

#### Other electrochemical tests

GCD tests were conducted using a Neware battery test system in a room maintained at an ambient temperature of approximately 25–28 °C. All other electrochemical measurements described below were performed at an ambient laboratory temperature of approximately 18–25 °C. Cyclic voltammetry, linear sweep voltammetry and EIS measurements were performed using a Biologic VMP-3 potentiostat. For the electrochemical stability window analysis, linear sweep voltammetry measurements were performed using a three-electrode configuration consisting of a titanium mesh working electrode (1 × 2 cm), an AC counter electrode (1 × 2 cm) and an Ag/AgCl reference electrode at a scan rate of 2 mV s^−1^. The measured current was normalized to an exposed geometric area of 1 cm², and the potentials are reported directly versus Ag/AgCl. The polarization measurements were performed in asymmetric Zn–Ti CR2025 coin cells using Zn foil (16 mm in diameter), titanium foil (12 mm in diameter) and a glass fibre separator (19 mm in diameter) at a scan rate of 5 mV s^−1^. The measured current was normalized to the geometric area of the titanium electrode. For in situ EIS tests, a thin Zn wire was inserted into the coin cell as an additional reference electrode to establish a three-electrode configuration. This reference electrode was placed between the positive electrode and negative electrode, separated by two layers of separators (GF/A, Whatman). For measurements of the individual iodine positive electrode, the potential is reported versus the Zn reference electrode as potential (V versus Zn/Zn²^+^), whereas the full-cell and Zn negative electrode responses are reported as voltage (V) owing to the voltage was applied on full cell. For peak assignment, the assembled cells were connected to a Biologic VMP-3 potentiostat to perform staircase potentio EIS with a potential range of approximately 0.6–1.8 V. For rate-step identification, the assembled cells simultaneously connected to a Biologic VMP-3 potentiostat and a Neware battery test system to conduct potentio EIS and GCD cycling, respectively. All Nyquist plots were fitted using EC-Lab software based on the equivalent circuit (R1 + Q2/R2 + Q3/R3 + W4). DRT data were calculated from EIS data using MatlabR2019 with the DRT tool developed by Francesco Ciucci’s research group. The differential capacitance was calculated using the following equation:

$$C=-{(\omega {Z}_{\mathrm{im}})}^{-1}$$

where *C* is the differential capacitance, *ω* is the angular frequency and *Z*_im_ is the imaginary component of the impedance. In this work, a specific frequency of 1,000 Hz was used. Measurements were performed in asymmetric Zn–Ti cells assembled in CR2025 coin-cell configuration, using Zn foil (16 mm in diameter) and titanium foil (12 mm in diameter) as the two electrodes and a glass fibre separator (19 mm in diameter). The potential was varied from 1.0 V to 1.7 V versus Zn/Zn^2+^. No ohmic-drop (*iR*) correction was applied to the potentials or voltages reported in this work.

### Theoretical calculations

#### Classical molecular-dynamics simulations

All molecular-dynamics simulations were performed using the LAMMPS package^45^ to investigate the bulk solvation structure of various aqueous electrolytes. The initial configurations were constructed using Packmol^46^, which randomly packed electrolyte components into a periodic cubic box according to the target molar ratios, ensuring no initial atomic overlaps. The simulation cell was then relaxed by energy minimization before subsequent equilibration. Force-field parameters for organic molecules were taken from the OPLS-AA family^47,48^, whereas others were adopted from validated aqueous zinc salt parameter sets in the literature. To partially account for polarization effects in concentrated electrolytes, the atomic charges were uniformly scaled (scaling factor 0.8)^49^. Interaction of water was represented by TIP3P^50^, and all X–H bonds were constrained using the SHAKE algorithm^51^. Each system was first energy-minimized and equilibrated in the isothermal–isobaric ensemble (300 K, 1 atm) using a Nosé–Hoover thermostat until both the density and potential energy were stable. Subsequently, production runs were performed in the canonical ensemble for 5 ns (5,000 ps) with a 1-fs integration step to ensure complete equilibration while avoiding structural drift.

#### Structural and hydrogen-bond analysis

Radial distribution functions *g*_*ij*_(*r*) were computed to analyse ion pairing and solvation structure. Coordination numbers were obtained by integrating *g*_*ij*_(*r*) up to their first minimum. Both radial distribution functions and hydrogen bonds were identified using the hydrogen-bond analysis tools implemented in VMD^52^. Average hydrogen-bond numbers and distributions were determined over the equilibrated trajectories to characterize the reorganization of the hydrogen-bond network by different anions.

#### Electric-field simulations

To probe field-induced ion and molecular redistribution, an additional set of simulations was conducted in the canonical ensemble with a uniform static electric field applied along the *z* axis. The field strength corresponded to the experimentally estimated potential gradient.

#### Halogen-bond analysis

Density-functional-theory calculations were performed using the Gaussian16 program package, using the M06-2X exchange-correlation functional^53^. Geometry optimizations were conducted using a mixed basis-set approach: the 6-311+G(d,p) basis set was applied to all elements excluding iodine^54^. Iodine was described using an effective core potential from the Stuttgart/Dresden group^55^. Solvent effects were accounted for using the polarizable continuum model, with water selected as the solvent^56^.

## Online content

Any methods, additional references, Nature Portfolio reporting summaries, source data, extended data, supplementary information, acknowledgements, peer review information; details of author contributions and competing interests; and statements of data and code availability are available at https://doi.org/10.1038/s41586-026-11282-0.

## Supplementary information

Supplementary Information Supplementary Figs. 1–67, Tables 1–4 and References.

Peer Review File

## Source data

Source Data Fig. 2

Source Data Fig. 3

Source Data Fig. 4

## Data Availability

The data that support the findings of this study are available within this article and its Supplementary Information. Further data are available from the corresponding author upon request. Source data are provided with this paper.
