# Supplementary material for: Non-halogen aqueous electrolytes for four-electron iodine conversion
Source: Nature. 2026 Nov 11;659(8140):372–7. doi: 10.1038/s41586-026-11282-0 (PMC13754563; doi:10.1038/s41586-026-11282-0)
Supplement: Supplementary file 1 [file 41586_2026_11282_MOESM2_ESM.pdf]

nature portfolio

Peer Review File

Non-Halogen Aqueous Electrolytes for Four-Electron Iodine Conversion

Corresponding Author: Professor Guanjie He

Any redactions in this file are there to maintain patient confidentiality, the confidentiality of unpublished data, or to remove third-party material.

This file contains all reviewer reports in order by version, followed by all author rebuttals in order by version.

Version 0:

Reviewer comments:

Referee #1

(Remarks to the Author)
This manuscript presents a highly novel and systematic approach to overcoming the long-standing challenge of reversible four-electron iodine conversion in aqueous batteries. The authors propose a universal KA–PA–Nuc electrolyte design paradigm, ingeniously leveraging anionic chemistry to eliminate the dependence on corrosive, high-concentration halogen-containing electrolytes. The work combines insightful theoretical design, extensive molecular-level analyses, and rigorous electrochemical evaluations.

In my opinion, the manuscript represents a significant advance in aqueous metal–iodine battery chemistry, not only by breaking the conventional electrolyte design bottleneck but also by introducing an elegant model to balance halogen bonding and hydrogen bonding interactions through ion-specific effects. The scope, clarity, and depth of the study are impressive, and the results are expected to open new directions in sustainable energy storage chemistry.

I recommend the manuscript for publication in Nature, pending consideration of the following comments and clarifications.

1. Originality and significance
The proposed KA–PA–Nuc strategy is, to my knowledge, the first electrolyte design that enables reversible $I^-/I^0/I^+$ redox processes at low concentration (2.4 M) without halogen ions. This represents a paradigm shift from conventional strategies relying on Cl^-/Br^- for halogen bonding.

The introduction of kosmotropic and polar anions to respectively weaken water-Nuc interactions and modulate the electrolyte interface is both mechanistically novel and practically impactful.

The universal applicability of the approach is strongly validated across multiple nucleophilic species (urea, acetamide, pyridine, nicotinamide) and in both Zn-I_2 and Al-I_2 systems, greatly strengthening the generalizability claim.

The cost reduction achieved (6.34-fold lower cell cost vs. 30 m $ZnCl_2$-based electrolytes) further highlights the real-world relevance of this study for grid-scale storage.

Assessment: The novelty is high, and the impact is substantial.

2. Scientific Rigor and Clarity
The authors present a comprehensive experimental and theoretical framework, supported by:

Electrochemical measurements: CV, GCD, EIS, DRT analyses for various electrolyte systems provide consistent evidence for improved reversibility and kinetics in KA–PA–Nuc electrolytes.

Spectroscopic evidence: In situ ATR-IR, NMR (^1H, ^{13}C, ^{15}N, 2D HMBC) and Raman clearly trace interfacial water structure and I^+–Nuc interactions.

Molecular dynamics (MD) simulations: Support the polar anion adsorption and hydration structure hypotheses.

DFT calculations: Used to probe anion polarities and explain halogen bond formation.

Techno-economic analysis: Provides valuable insight into the cost advantages and scalability potential.

Figures are well-constructed and informative. The logical flow from problem identification to solution implementation is coherent and well justified.

3. Specific Comments and Suggestions
(1) Electrochemical stability window
The manuscript would benefit from including linear sweep voltammetry (LSV) data or discussion regarding the electrochemical stability window (ESW) of the KA–PA–Nuc electrolyte, especially given the use of acetamide and pyridine as Nuc species.

(2) Quantification of halogen bonding
While the authors suggest $INuc_2^+$ complexes based on peak evolution and CV/GCD profiles, is there quantitative evidence (e.g., binding energy calculations, ^{15}N shift trends, or XPS spectra) to distinguish stable halogen bond formation from transient complexation?

(3) Long-term chemical stability
Although cycling stability is excellent over 1000–2000 cycles, it would be helpful to report any ex situ structural changes to the electrodes or the electrolyte composition post-cycling (e.g., via XRD, FTIR, or HPLC analysis) to confirm electrolyte chemical robustness.

(4) Hydrogen bonding model in KA effect
The MD simulations and NMR shifts provide qualitative support for water-Nuc hydrogen bond disruption by kosmotropic anions. However, a more quantitative correlation (e.g., hydrogen bond lifetime from MD or hydration energy data) would further solidify this conclusion.

(5) Compatibility with commercial current collectors
Given the practical orientation of the work, the use of titanium mesh is reasonable. Still, a brief discussion on the compatibility of the proposed electrolyte with cheaper stainless steel or carbon-based current collectors would be beneficial for readers.

(6) Rate-limiting step identification
In the DRT and in situ Raman analyses, the evolution of T_3/T_4 peaks suggests potential bottlenecks at high voltages. Could the authors elaborate on whether ion transport or interfacial charge transfer becomes dominant under fast charging?

4. Minor Points
Line 33: "provides a new avenue" → consider specifying: "a new electrolyte design avenue"

Line 112: "this work establishes a new paradigm" → consider softening to "this work introduces a new design paradigm"

Figures: Please ensure color consistency for KA-only, PA-only, and KA–PA curves across CV, GCD, and DRT plots.

Figure 6d-e: Units in cost breakdown charts could be labeled more explicitly (USD/kWh)

A table summarizing electrolyte compositions and corresponding performance (capacity, retention, redox reversibility) would help readers.

5. Summary and Recommendation
Strengths:

Conceptually innovative electrolyte strategy

Systematic and cross-validated mechanistic analyses

Excellent electrochemical performance and cycle life

Broad compatibility and economic advantage

Weaknesses (relatively minor):

Lacks direct quantification of halogen bonding in some systems

Deeper insight into ESW and long-term electrolyte integrity would be beneficial

Referee #2

(Remarks to the Author)
This manuscript presents an electrolyte design strategy aimed at achieving the reversible four-electron reaction of iodine-based cathode materials. It tries to establish a fundamental compositional framework comprising kosmotropic anions, polar anions, and nucleophilic species. While the study addresses a relevant and timely topic, the rationale underlying some of the key conclusions appears to be somewhat overstated when evaluated in terms of robustness, validity, and reliability. The conclusions would benefit from a more cautious and data-driven interpretation, with closer alignment to the experimental evidence. To enhance the logical coherence and scientific rigor of the manuscript, further clarification and justification of several aspects of the experimental interpretation are recommended.

- In Figures 2b–2g, the authors compare the reversibility of the I0/I+ redox couple using various electrolyte combinations. However, this experimental design appears to rely on the underlying assumption that I+ is stabilized predominantly through complexation with nucleophilic species (i.e., forming a Nuc-I-Nuc complex). It should be noted, however, that I+ may also be stabilized via complexation with other anions present in the electrolyte, rather than exclusively by nucleophiles. To clearly rule out this alternative pathway, it is recommended that the authors include CV and GCD data for the KA-PA electrolyte system, which does not contain nucleophilic species.
The authors base their assumption of Nuc-I-Nuc complex formation on Ref. 21. However, in that reference, the molar ratio of pyridine derivatives is twice that of anions, whereas in the present study, anions are present in overwhelming excess. This discrepancy makes a direct comparison of complex formation behavior difficult. Therefore, the potential stabilization of I+ by anions, especially polar ones, should be carefully considered.

- Additionally, the KA-PA-Nuc, PA-Nuc, and KA-Nuc electrolytes in Figure 2 appear to differ in total ionic concentration or ionic strength. Given that these parameters can substantially affect electrolyte conductivity and water molecule hydration, the reviewer recommends performing comparative experiments under standardized ionic strength to ensure more rigorous and interpretable results.

- Furthermore, in Figure 2, it is difficult to confirm the current scale of each cyclic voltammogram due to the absence of clearly defined y-axis values or scale indicators. To improve the clarity and comparability of the data, it is recommended that the authors include scale bars or explicitly label the current axis for each panel.

- As noted by the authors, the PA-Nuc electrolyte in Figures 2e and 2f exhibits a second charge plateau with a notably large capacity. Theoretically, if all I0 were irreversibly oxidized to IO3-, the maximum charge capacity should be up to five times that of the I-/I0 redox couple (first charge plateau). However, the experimentally observed capacity appears to exceed this limit. Therefore, further discussion is needed to clarify the nature of the reactions occurring during the second charge plateau.

- In Figure 3, the authors propose that polar anions preferentially adsorb onto the electrode surface, creating an anion-enriched and water-deficient electrolyte/electrode interface that enhances the reversibility of the four-electron reaction. However, it remains unclear whether the oxidation products (I+ or Nuc-I-Nuc) are immobilized at the electrode surface or can diffuse or migrate into the bulk electrolyte. Clarification on the spatial localization of these species is necessary. Moreover, the assertion that the water distribution within 5 Å of the electrode governs reaction reversibility requires more robust justification.

- Regarding the ATR-IR results in Figures 3g-3i, it is unclear under which electrochemical condition (e.g., charging or discharging) the data were collected. As the polarity of the electrode changes during cycling, the interfacial ion distribution is expected to vary significantly. Therefore, clarification of the measurement conditions is essential for proper interpretation. If ATR-IR spectra were collected under both charging and discharging conditions, the interpretation of interfacial changes would be significantly clearer.

- In Figure 3i, it is unclear why the nonpolar sulfate anion appears in such high abundance. Further explanation is needed to clarify this observation.

- The rationale for excluding halide anions from the study of kosmotropic effects is not clearly stated. Given that the main objective of this work is to propose electrolyte design rules, including halide anions to complete the observed trends would enhance the comprehensiveness and impact of the study.

- In the NMR analysis presented in Figure 4, the authors claim that the chemical shift of the 2H peak indicates strong coordination between sulfate ions and water molecules. However, stronger anion-water coordination is typically expected to increase electron density around the hydrogen nuclei, leading to a shielding effect and an upfield shift. Similarly, weaker hydrogen bonding between water and the nitrogen atoms in Nuc molecules would increase local electron density around nitrogen, also resulting in an upfield shift. In contrast, the present study reports pronounced downfield shifts in both 2H and 15N spectra for the sulfate anion system. A more convincing interpretation is needed to reconcile these observations with the proposed coordination behavior.

- To better understand the interfacial electrochemical behavior, DRT analysis was conducted. While DRT is a useful tool for probing reaction mechanisms, it typically requires support from complementary in situ techniques to enable reliable peak assignments. In this study, however, the assignments of key peaks—particularly t2, t3, and t4—remain unclear. Moreover, a reduction in impedance alone does not necessarily indicate suppression of the I+ hydrolysis reaction, as the I0/I+ redox

process likely consists of sequential charge-transfer and subsequent chemical hydrolysis steps.

- In Figure 5e, the assignment of the Raman peak to the Nuc-I-Nuc complex is not clearly justified. Given the presence of other anions in the system, alternative complexation pathways should also be taken into account.

- The specific capacity values (mAh/g) reported in Figure 6 appear to be a key performance metric. If these values are based on the mass of iodine loaded on the cathode, it would be helpful to specify the iodine loading (mg/cm2). This would allow estimation of the areal capacity (mAh/cm2) and facilitate evaluation of the practical relevance of the single-cell tests.

Referee #3

(Remarks to the Author)
This manuscript presents an interesting and impactful study on the development of cost-effective, halogen-free aqueous electrolytes that enable reversible four-electron iodine redox reactions, overcoming a long-standing challenge in the field of aqueous metal-iodine batteries. The authors demonstrate a novel electrolyte design strategy guided by anionic chemistry, breaking away from traditional reliance on concentrated, halogen-rich systems. Such a universal anionic chemistry design principle successfully decouples hydrogen bonding from halogen bonding, allowing stable and reversible four-electron iodine redox reactions in a low-concentration and halogen-free aqueous environment. The universal electrolyte principle proposed here is broadly applicable and can inspire similar innovations across other aqueous redox systems. Therefore, the manuscript may be considered in Nature, but the following points must be addressed,

1.The authors investigated the interfacial microenvironment of the electrolytes with varying anion polarities using in situ ATR-IR. However, the manuscript lacks a comparative analysis of high-concentration halogen-containing electrolytes. Specifically, what is the mass distribution at the electrode–electrolyte interface under such conditions, and how does this influence the electrochemical behavior?

2.I am worried that Figure 6f is overclaimed. The cost of LIB based on LFP is very, very cheap nowadays. This relies on two points, one is its very high energy density (denominator), the other is its very mature supply chain, leading to a low BOM (Numerator). The current aqueous batteries, with a much lower energy density (the energy density of LPF-based LIB can be over 400Wh/L), make it impossible to have a lower Wh cost. I suggest the authors do a more realistic comparison instead of an exaggerated one. In fact, cost can be even more difficult than performance.

3.The CEs, revealed in figures 4 and 6, are all very low. What are the reasons, the cathode, anode or other components?

4.In addition to the interfacial microenvironment, the solvation structure within the bulk electrolyte plays a critical role in governing electrochemical performances. Therefore, comprehensive investigations of the bulk properties (such as ion solvation structure and coordination environment) across different electrolyte systems must be provided.

5.The authors present the final optimized electrolyte formulation without detailing the rationale or optimization process behind its selection, lacking an explanation of how electrolyte optimization could address critical issues in four-electron iodine redox reactions.

6.The I^+ cation is well known to undergo hydrolysis in aqueous environments, posing a major challenge for achieving stable multi-electron iodine redox reactions. The authors propose that the electrophilic I^+ species is stabilized via the formation of linear halogen bonds with nucleophilic components in the designed electrolyte. However, this important claim warrants further evidence. Additional mechanistic insights—such as spectroscopic validation, structural characterization, or theoretical modeling—would be valuable to substantiate the proposed halogen-bonding interactions and their role in stabilizing I^+ in aqueous media.

7.In the context of linear halogen bonding, the iodine cation (I^+) carries a positive charge, while the two coordinating nucleophilic species are electrically neutral. As a result, the resulting halogen-bonded complex is positively charged and requires counterions for charge compensation. This raises an important question regarding the role of anions in the proposed electrolyte system. Beyond modulating the interfacial microenvironment and influencing molecule-water interactions, do the anions also contribute to charge stabilization or participate in other critical functions?

8.When presenting the analysis based on the DRT, it is important to also provide the corresponding electrochemical impedance spectroscopy (EIS) data. Analyzing the original EIS spectra alongside the DRT results will enhance the credibility of the interpretation and allow for a more comprehensive understanding of the underlying electrochemical processes.

9.The manuscript employs Ti mesh as the current collector; however, titanium is relatively expensive and may not be suitable for practical, large-scale applications. Could the authors clarify the rationale for selecting Ti mesh in this study? Additionally, it would be valuable to assess whether more cost-effective alternatives-such as stainless steel-could be used without compromising electrochemical performances. This would enhance the practical relevance and scalability of the proposed system. Data with other current collectors should be provided.

10.To evaluate the performance of the proposed electrolyte standard in multi-electron aqueous $Al–I_2$ batteries, the authors employed activated carbon electrodes as both the reference and counter electrodes. However, the use of activated carbon

as a reference electrode is not appropriate, as it does not provide a stable or well-defined potential. This compromises the accuracy of the electrochemical measurements. A standard three-electrode configuration can be used, incorporating a reference electrode with a known and stable potential.

Version 1:

Reviewer comments:

Referee #2

(Remarks to the Author)
The authors have adequately addressed the comments by this reviewer and significantly improved the manuscript. I recommend to accept this work at its present form.

Referee #3

(Remarks to the Author)
The authors' response generally meets my expectations. But the paper still has some issues to improve its quality:
A lot of grammatical and typographical errors, such as (even on the first few pages)
industry grids should be industrial grid,
and electronic devices call for should be and electronic devices calls for
determined by the number of electron transfer should be determined by the number of electrons transferred
Giving that the competition between halogen should be Given that the competition between halogen
while, anion-specific effect, should be while, anion-specific effects
Many others, the authors should carefully check.

Another issue is that since the I-I+ reactions have been realized in halogen electrolytes, the high-voltage plateau contribution to the overall capacity should be compared with that of halogen electrolytes.

When electrochemical impedance spectroscopy (EIS) measurements are presented in a complex impedance plot (i.e., the Nyquist plot), the graph must be ortho-normed (i.e., same scale on X and Y axes), with raw impedance data reported as symbols and fitted data as lines.

The Al anode should be significantly more negative than Zn, but the charge-discharge profile of Al-I2 is quite similar to that of Zn-I2. Is the Al working, or is the Zn still working, since the Al-Zn alloy is used as an anode? What about the gas evolution of the Al-I2 batteries?

Referee #4

(Remarks to the Author)
Having read the revised manuscript, the comments from three reviewers, and the rebuttal from the authors, I offer the following thoughts.
Overall, the authors have done an exceptionally thorough job in addressing the bulk of the comments from the reviewers, who, in turn, raised a comprehensive list of issues that certainly required attention. As a result, the revised manuscript is clearly, in my view, much stronger, particularly in providing more information on the key interactions between the three electrolyte species (KA, PA and Nuc). There are, though, aspects of the electrochemical behaviour that cast doubt on some of the claims made for this version of the iodine electrode.

Comment 6 from R2 questions the evidence provided for the immobilization of $I^+(Nuc)_2$ at, or close to, the electrode surface once it is formed. The response, based on UV-vis monitoring of triiodide does not really answer the question posed. The formation and migration of triiodide will affect the characteristics of the first redox process but as long as it is stable in the bulk solution, there is no reason for it not to be eventually consumed in the first oxidation wave (charge capacity data seem to support this).
Later in their response to this comment, the authors mention their ATR-IR results, shown in Fig.3g-i. For the most polar anion (acetate, Fig 3i), there is certainly a lower concentration of water present. But the concentration is still appreciable. The designation ‘deficient’ is accurate, but is it ‘deficient enough’ to prevent side reactions related to water?

Comment 3 from R3 notes that the CEs are low. I would also add that specific values of CE are nowhere to be found in the manuscript. All CE axis scales (with the exception of SI Fig 53) are drawn with ‘90%’ as the highest marked value. This gives the impression of a metric to which the authors do not wish to draw attention. In response to R3’s comment, the authors provide a commentary that mainly draws comparisons between their best and worst electrolyte formulations – this does not explain the issue. But they also note the following: ‘This phenomenon primarily arises from the intrinsic characteristics of high-valent iodine redox process, which inherently involve multi-step electron transfer and transient intermediate species. Similar behavior has been widely reported in previous studies employing high-concentration, halogen-containing electrolytes, reflecting the inherent complexity and intrinsic instability of high-valent iodine redox chemistry’. I think this indicates that there are grounds for concern here. As is well-known, CE can be tied directly to battery cycleability.

Exceptions occur with lead-acid and some nickel-based aqueous systems, where excess charge can feed a close-loop overcharge reaction that effectively maintains full capacity (other failure modes notwithstanding of course). In the present case though, one has to wonder about the effects of the 'instability' that is mentioned here.
I think that evidence of underlying instability becomes more obvious in the data provided for the higher loaded cells. SI Figs 54d,e show that capacity and CE plots become decidedly 'noisy' at the highest loading. Such behaviour, along with the increasing rate of capacity loss, is certainly consistent with the greater impact of effects related to instability. Another indicator of background instability is in SI Fig 51b (GCD performance at variable rates). Specifically, the apparent inverse relationship between rate and CE, where CE only approaches 100% at the highest rates, thereby indicating that side reactions are present.
At this point, my impression is that the authors have buttressed the chemical evidence supporting their claims as much as possible, in line with the comments made by the reviewers. They have not, though, addressed the issue of low CE, and nor have they sought to probe the reason(s) for the cells losing capacity. These two points are undoubtedly linked, so that a proper post mortem analysis could well have provided more insight into the behaviour of this Zn-I2 system. As it stands, though, samples from the high- and ultra-high loading cells were, according to SI Figs 55-57, only taken up to the 80-cycle mark, by which point none of the cells had undergone any capacity loss. As presented, this analysis merely tells the reader that there are no indicators of significant change in the electrodes or separators, after a relatively insignificant number of cycles. And yet, these cells do fail, as seen in SI Fig 54, where almost 30% loss is noted for the 12.2 mg/cm2 cell. It is at this point where a much fuller understanding of cell behaviour would be obtained, through examination of electrodes, separators and electrolyte samples. Analyses should include a full iodine inventory, cast against the initial balance of active materials so that a true measure of the performance of this system can be established.
My concluding view is that this study cannot be considered suitable for publication until a thorough investigation is conducted into the low CE and how this is related to the underlying mechanism of capacity loss.

Final comments, related to minor side-issues

What is the reason for the very large capacity figures in the x-axis of SI Fig. 1a and of Fig. R26?

Comment 10 from R2 raises an apparently contentious issue regarding NMR proton shifts and hydrogen bonding. R2 argues for an upfield shift in the proton resonance, which is opposite to that proposed here. R2's argument appears sound, based on literature. The rebuttal talks in localized terms only, without considering the role of hydrogen bonding throughout bulk water. I think that a more extensive discussion of this issue is warranted.

Version 2:

Reviewer comments:

Referee #4

(Remarks to the Author)
The authors have now examined in commendable detail the causes of capacity loss in these Zn-I2 cells and then thoroughly explained the effects in terms of iodine chemistry. The complex relationship between charge passed in the various reactions and the many reaction pathways is well explained and, as posited by the authors, will help guide future research.

So where does all this new information leave us?

From the added discussion, it is clear that fugitive triodide, in particular, causes cycle-life-limiting effects. In the revised SI, the authors note that the abundance of basic zinc sulfate on the anode grows with cathode loading and cycle number. From the discussion in the rebuttal, but not included in the revised SI, they note that this is due to corrosion of zinc. This will come as no surprise to electrochemist readers. Most of the latter would agree that triiodide-related corrosion thwarted the progress of the Graetzel cell (before it was eventually killed off by cheap silicon).

I am concerned that zinc anode failure limits cycle-life in a cell where the zinc is presumably in significant excess. It's not clear how large the excess is though. While the technoeconomic (TE) modelling uses a factor of two, the Methods Section does not provide this parameter for the actual test cells. There is an interesting point here. While the authors show that a significant part of the charge loss responsible for low CE is associated with fundamentally reversible reactions, this is somewhat unimportant with respect to what appears to be the ultimate failure mode. Regardless of whether the triiodide formed is part of a reversible chain of reactions, the fact that some of it escapes the confines of the cathode and then reacts with the anode is clearly cell-life-limiting. In a way, the high degree of overall reversibility actually guarantees that a constant supply of triiodide will be available to react with the anode.

The TE analysis generates a comparison (Fig. 6f) in which the authors' version of Zn-I2 betters all compared chemistries by a considerable margin. However, the analysis seems to be missing some details. My main question concerns the cathode. There is no costing of the actual cathode material (i.e., iodine deposited on to activated carbon substrate). Simply costing iodine as the cathode reduces the total cost unrealistically. For the anode, does the twofold excess relate to any of the cells made and tested in this study? In other words, given the corrosion issue, what order of cycle-life would this excess provide?

The point that I raised in my review regarding the sensitivity of cycle performance to current (rate) was not addressed directly. It is clear to me that this cell chemistry will become less efficient, and lose capacity at a greater rate, as the rates of

charge and discharge are lowered. This would limit its application significantly.

In the revised version, line 439 states: ‘The KA–PA–Nuc electrolyte effectively mitigates these parasitic pathways…’
I disagree – ‘effectively’ implies that the issue of product migration has been neutralised when clearly it has not – the evidence shows that the corrosion of the zinc anode ultimately limits cycle-life.
My overall opinion is that this work is still not suitable for publication in Nature. While the authors have now described essentially every aspect of this system in great detail, I disagree with the contention that this cell chemistry is, in the guise presented here, competitive with contemporary battery technologies. The flaws in this version of Zn-I2 that have been identified will have seriously deleterious operational consequences. The fact that these flaws are lessened by the new electrolyte is important, but does not remove them from consideration. The benchmark in battery performance set by lithium-ion, and being followed closely by sodium-ion, continues to improve. In response, any new competing chemistry must be better still. At present, Zn-I2 does not look like a plausible contender, and therefore this study does not generate the impact required for publication here.

Open Access This Peer Review File is licensed under a Creative Commons Attribution 4.0 International License, which permits use, sharing, adaptation, distribution and reproduction in any medium or format, as long as you give appropriate credit to the original author(s) and the source, provide a link to the Creative Commons license, and indicate if changes were made.
In cases where reviewers are anonymous, credit should be given to 'Anonymous Referee' and the source.
The images or other third party material in this Peer Review File are included in the article’s Creative Commons license, unless indicated otherwise in a credit line to the material. If material is not included in the article’s Creative Commons license and your intended use is not permitted by statutory regulation or exceeds the permitted use, you will need to obtain permission directly from the copyright holder.
To view a copy of this license, visit https://creativecommons.org/licenses/by/4.0/

Manuscript Title: Low-Concentration and Non-Halogen Aqueous Electrolytes to Achieve Reversible Four-Electron Iodine Conversion Reactions

Manuscript number: 2025-04-08465A

Responses to Editor

Referee #1

This manuscript presents a highly novel and systematic approach to overcoming the long-standing challenge of reversible four-electron iodine conversion in aqueous batteries. The authors propose a universal KA–PA–Nuc electrolyte design paradigm, ingeniously leveraging anionic chemistry to eliminate the dependence on corrosive, high-concentration halogen-containing electrolytes. The work combines insightful theoretical design, extensive molecular-level analyses, and rigorous electrochemical evaluations. In my opinion, the manuscript represents a significant advance in aqueous metal–iodine battery chemistry, not only by breaking the conventional electrolyte design bottleneck but also by introducing an elegant model to balance halogen bonding and hydrogen bonding interactions through ion-specific effects. The scope, clarity, and depth of the study are impressive, and the results are expected to open new directions in sustainable energy storage chemistry. I recommend the manuscript for publication in Nature, pending consideration of the following comments and clarifications.

Response: We sincerely thank the reviewer for the very positive evaluation of our work and for recommending the manuscript for publication. We also appreciate the insightful and constructive comments provided to further improve this manuscript. We have carefully addressed all points raised, and the detailed point-by-point responses are listed below. We hope that the revised version meets your expectations.

Comment 1. Electrochemical stability window: The manuscript would benefit from including linear sweep voltammetry (LSV) data or discussion regarding the electrochemical stability window (ESW) of the KA–PA–Nuc electrolyte, especially given the use of acetamide and pyridine as Nuc species.

Response: According to the comment, we have included a detailed comparison and discussion of the electrochemical stability window (ESW) of various electrolytes based on linear sweep voltammetry (LSV) measurements. The LSV measurements were performed using a three-electrode configuration consisting of a Ti mesh working electrode, an activated carbon counter electrode, and an Ag/AgCl reference electrode at a scan rate of 2 mV s^{-1}. The results reveal that KA-PA-U electrolyte exhibits a low hydrogen evolution onset potential (< -1 V vs. Ag/AgCl). Compared with PA-U and KA-U electrolytes, the oxygen evolution potential is suppressed in KA-PA-U electrolyte (> 1.2 V vs. Ag/AgCl). This window fully encompasses the potential range required for the four-electron iodine conversion reactions, confirming that the electrolyte can remain stable during battery operation. Among them, PA-U shows the lowest oxygen evolution potential, which can be attributed to its relatively low salt concentration (Fig. R1a). Furthermore, similar trends were observed when acetamide, nicotinamide, and pyridine were employed as Nuc species, demonstrating that all KA–PA–Nuc electrolytes maintain sufficient electrochemical stability across the voltage range utilized in this study (Fig. R1b-d). These results confirm the compatibility of the designed electrolytes with the working potential of the four-electron iodine conversion reactions. The corresponding LSV data and discussion have been added to the revised manuscript: "*As a result, a new low-concentration, halogen-free electrolyte standard, termed KA-PA-Nuc, is proposed and optimized (Supplementary Fig. 2-3), which exhibits sufficient electrochemical stability (Supplementary Fig. 4) and offers a more sustainable way for four-electron iodine conversion reactions*".

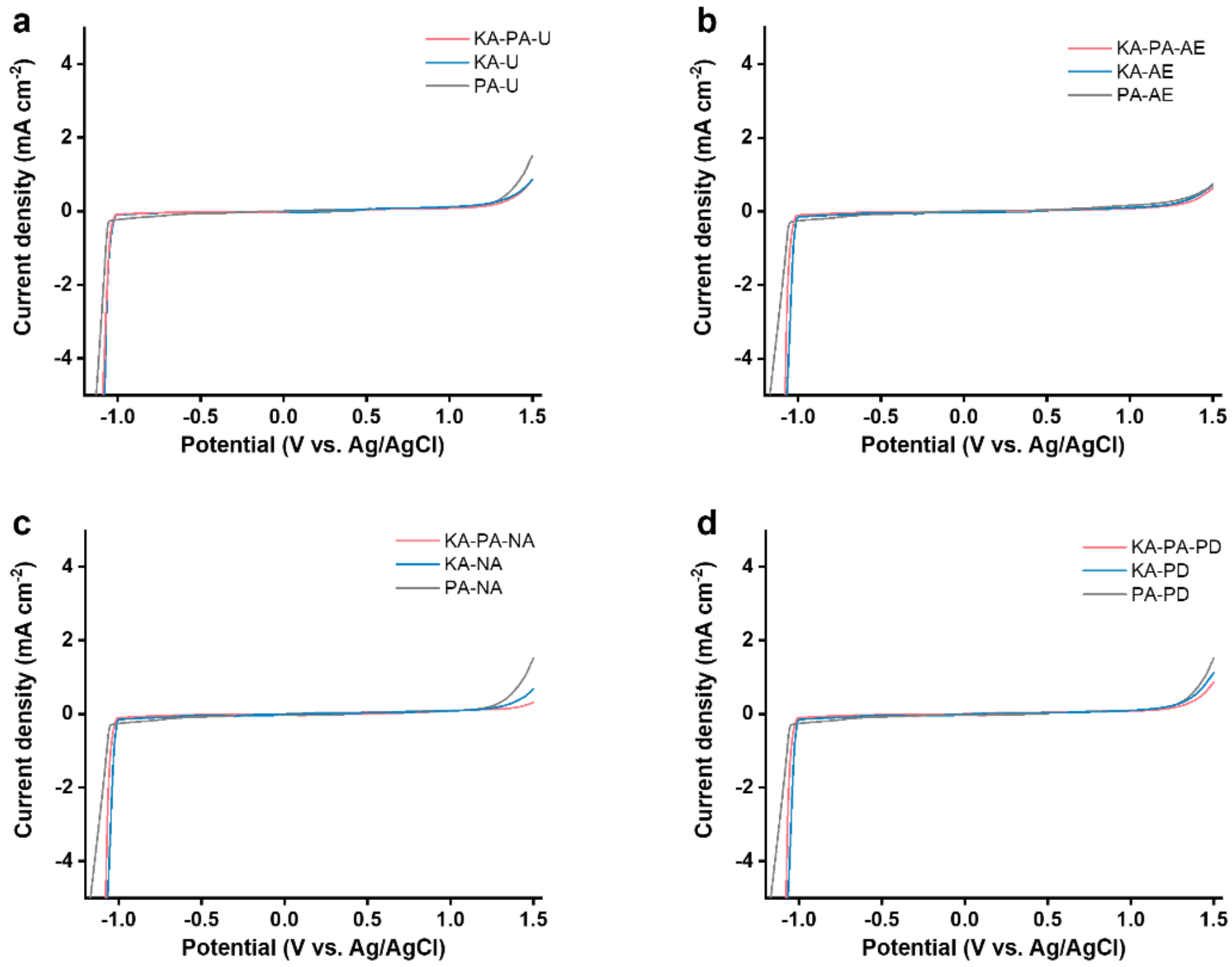

Fig. R1 Comparison of electrochemical stability window. a, Linear sweep voltammetry measurements in different electrolytes using urea (U) as Nuc. b, Linear sweep voltammetry measurements in different electrolytes using acetamide (AE) as Nuc. c, Linear sweep voltammetry measurements in different electrolytes using nicotinamide (NA) as Nuc. d, Linear sweep voltammetry measurements in different electrolytes using pyridine (PD) as Nuc.

Comment 2. Quantification of halogen bonding: While the authors suggest $INuc_2^+$ complexes based on peak evolution and CV/GCD profiles, is there quantitative evidence (e.g., binding energy calculations, ^{15}N shift trends, or XPS spectra) to distinguish stable halogen bond formation from transient complexation?

Response: To substantiate the halogen bond stabilization mechanism, we have performed a series of complementary electrochemical, spectroscopic, and theoretical investigations that collectively provide direct and quantitative evidence for Nuc–I^+–Nuc halogen bond formation. First, electrochemical control experiments show that in all

electrolytes without Nuc species, no reduction peak or discharge plateau associated with the I^0/I^+ redox process is observed (Fig. R2). The absence of I^+-related signals across all electrolytes without Nuc species confirms that I^+ cannot be stabilized by anions alone. By contrast, the presence of Nuc species enables successful I^+ conversion reaction, providing strong evidence that halogen bond interaction with Nuc species is essential for I^+ stabilization.

To directly quantify halogen bond formation, 2D ^1H-^{15}N HMBC NMR was employed, which is a sensitive tool for investigation of halogen bond formation. Pyridine was selected as a representative Nuc species. In the bare pyridine solution, coupled H2/H6-N1 and H3/H5-N1 signals appear at 314.78 ppm (Fig. R3a). Upon addition of molecular iodine (I_2), a small ^{15}N chemical shift is observed (Δ_{15N} = -22.76 ppm), consistent with weak interaction between pyridine and iodine (Fig. R3b). In sharp contrast, when pyridine is exposed to iodonium (I^+) species generated by an exchange reaction with corresponding silver analogues (Fig. R6a), a large ^{15}N chemical shift (Δ_{15N} = -107.84 ppm) is detected (Fig. R3c). This magnitude of chemical shift is characteristic of halogen bond formation, confirming direct Nuc–I^+ interaction and providing unambiguous spectroscopic evidence for the halogen bond stabilization mechanism.

To further identify the specific coordination configuration, binding energy calculations were conducted to evaluate possible geometries in our electrolyte system. The results reveal that the linear Nuc–I^+–Nuc halogen bond, accompanied by a counterion, is the most stable structure, exhibiting lower binding energy than anion-involved configurations (A^-–I^+–A^- and Nuc–I^+–A^-) (Fig. R4). These findings indicate that the linear Nuc–I^+–Nuc halogen bond is thermodynamically preferred, while anions only serve as counterions and do not directly coordinate with I^+. Moreover, when serving as counterions, different anions have negligible influence on the N–I bond length or binding energy, further supporting their non-coordination in halogen bond formation (Fig. R5). This observation is fully corroborated by additional 2D ^1H-^{15}N HMBC spectra, where the ^{15}N chemical shifts of Nuc–I^+–Nuc complexes remain

essentially unchanged across various counterions (Fig. R6). Collectively, these electrochemical, spectroscopic, and theoretical results provide multiple and quantitative evidence for the linear Nuc–I^+–Nuc halogen bond, which establish the halogen bond stabilization mechanism underlying the reversible four-electron iodine conversion reactions. Therefore, corresponding data and discussion have been added into the revised manuscript: "*These halogen bonds are the foundation of four-electron iodine conversion reactions, which has been fully demonstrated through electrochemical comparison in Nuc-free electrolytes, nuclear magnetic resonance (NMR) spectroscopy, and theoretical calculations (Supplementary Fig. 6-10)*".

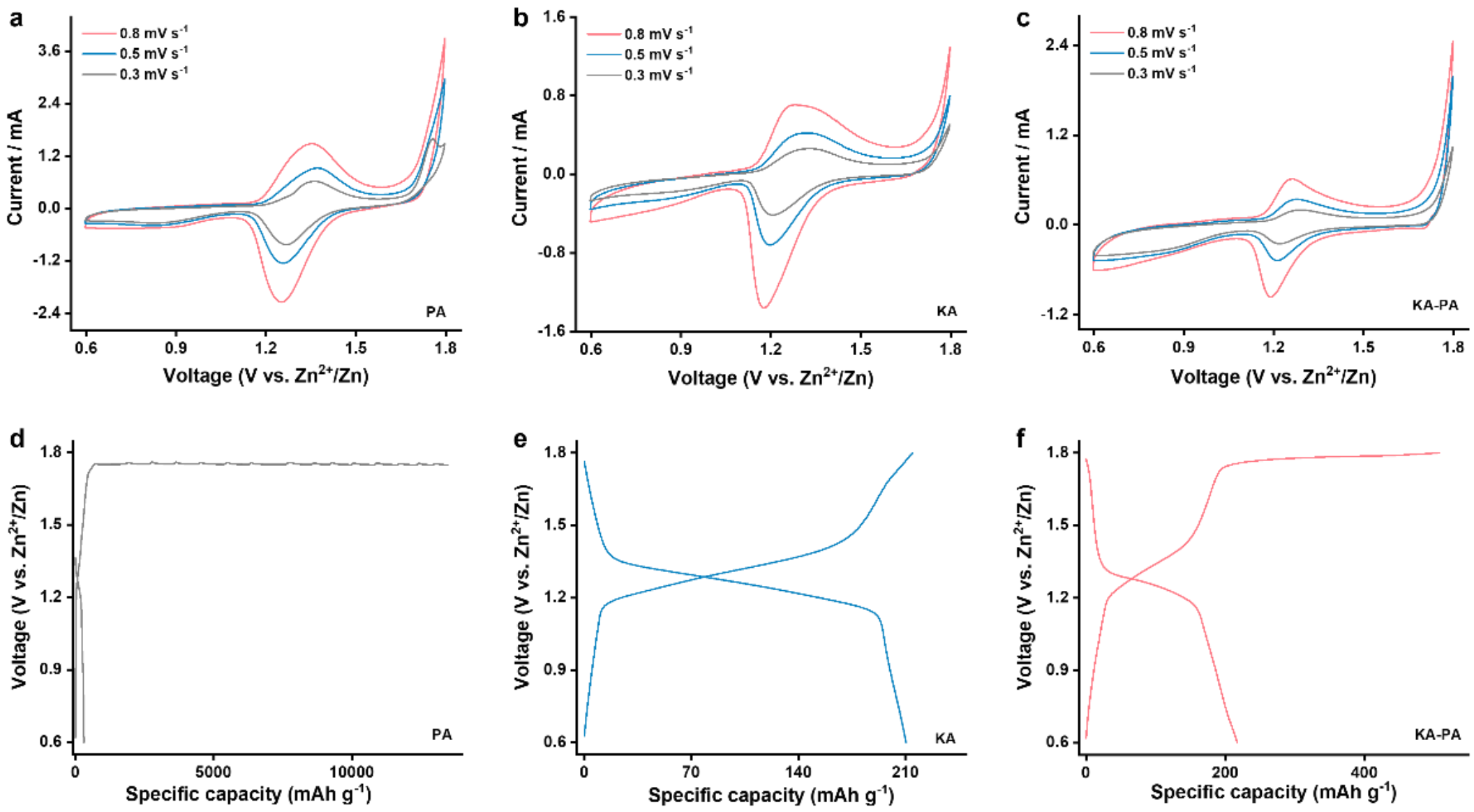

Fig. R2 Electrochemical performance of electrolytes without Nuc species. a-c, CV profiles of the iodine conversion reaction in PA, KA, and KA-PA electrolytes at various scan rates. d-f, GCD profiles of the iodine conversion reaction in PA, KA, and KA-PA electrolytes at 1 A g^{-1}.

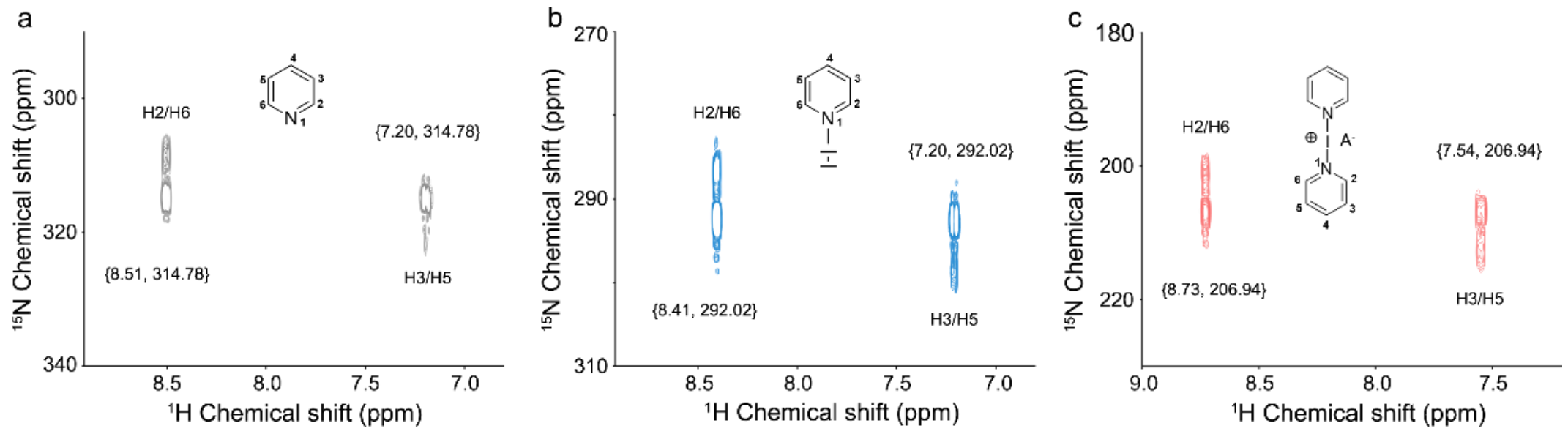

Fig. R3 Characterization of halogen bonds. a, 2D ^{1}H-^{15}N HMBC NMR spectrum of the bare Nuc solution (pyridine). b, 2D ^{1}H-^{15}N HMBC NMR spectrum of the pyridine solution with iodine. c, 2D ^{1}H-^{15}N HMBC NMR spectrum of the synthesized I^{+} complexes.

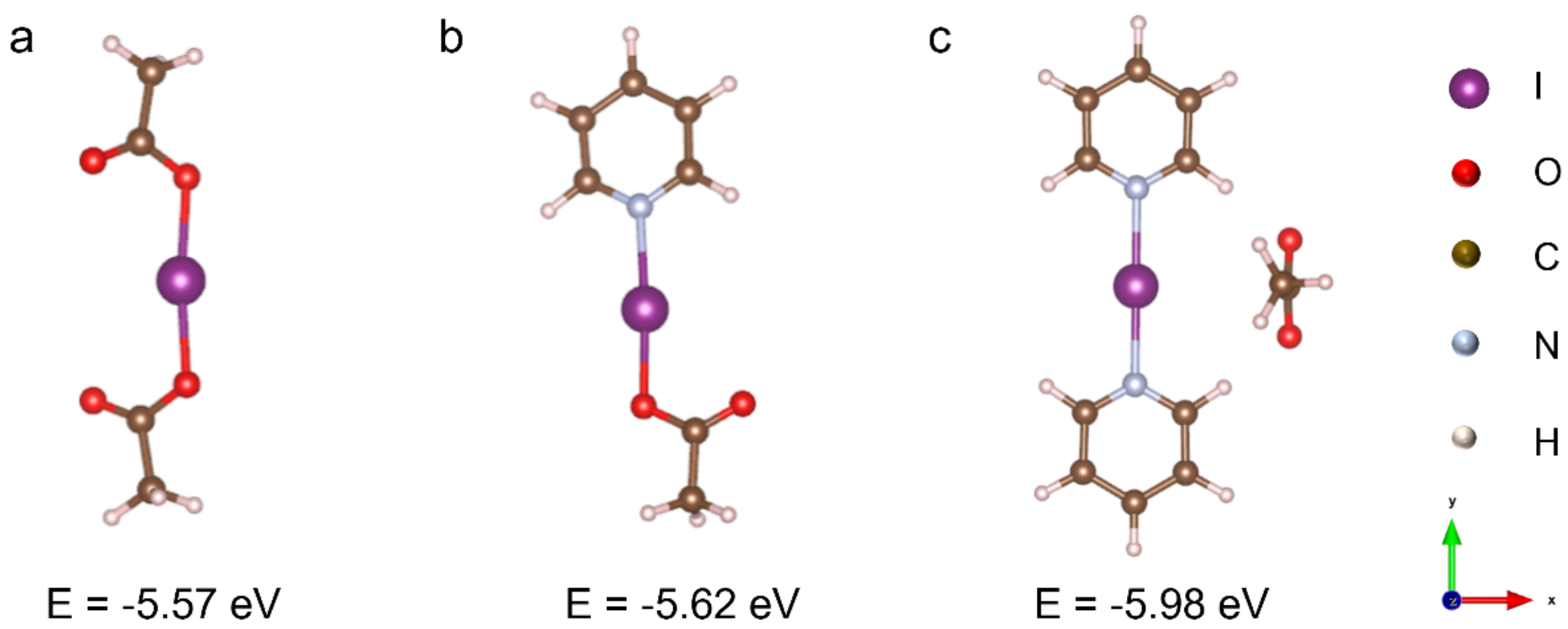

Fig. R4 Theoretical calculation of possible coordination configurations. a, The A^{-}–I^{+}–A^{-} coordination structure and corresponding binding energy. b, The Nuc–I^{+}–A^{-} coordination structure and corresponding binding energy. c, The Nuc–I^{+}–Nuc halogen bond and corresponding binding energy.

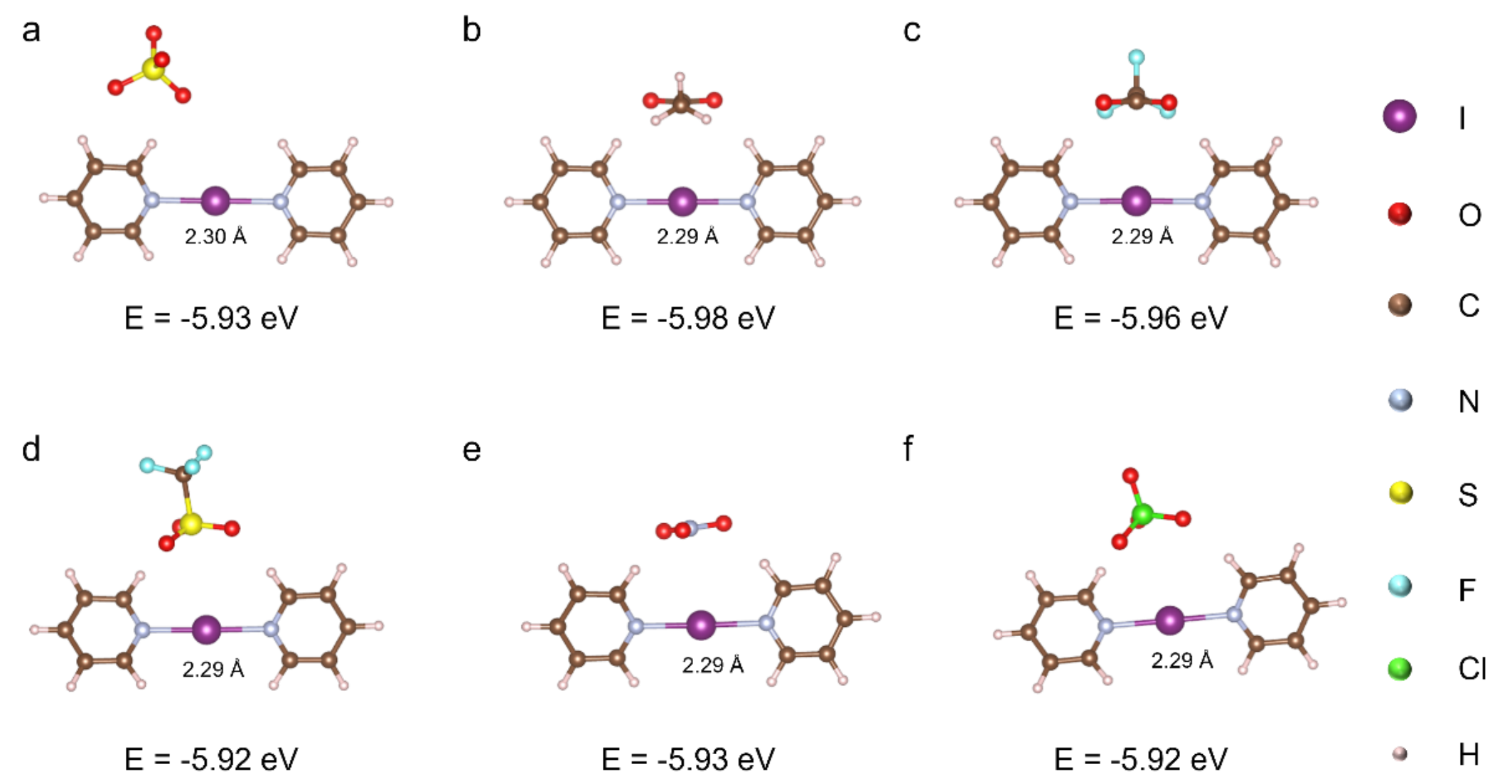

Fig. R5 Binding energy and bond length of Nuc–I$^+$–Nuc halogen bonds with different counterions. a, SO_4^{2-}. b, CH_3COO^-. c, CF_3COO^-. d, $CF_3SO_3^-$. e, NO_3^-. f, ClO_4^-.

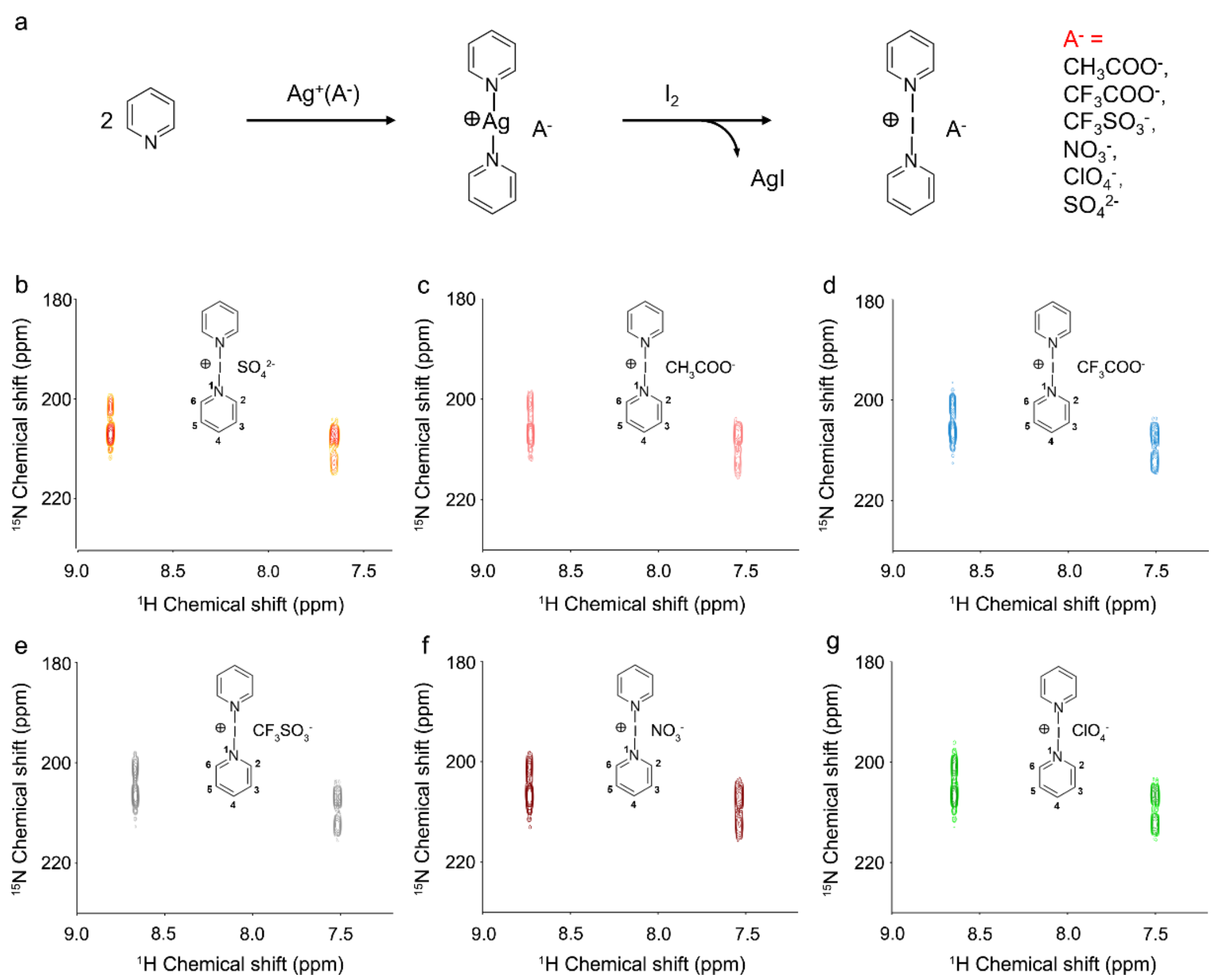

Fig. R6 Characterization of halogen bonds with different counterions. a, Schematic

illustration of the synthesis of I^+ halogen bond complexes with different counterions via a cation exchange reaction. 2D ^1H-^{15}N HMBC NMR spectrum of the synthesized I^+ complexes with different counterions: b, SO_4^{2-}; c, CH_3COO^-; d, CF_3COO^-; e, $CF_3SO_3^-$; f, NO_3^-; g, ClO_4^-.

Comment 3. Long-term chemical stability: Although cycling stability is excellent over 1000–2000 cycles, it would be helpful to report any ex situ structural changes to the electrodes or the electrolyte composition post-cycling (e.g., via XRD, FTIR, or HPLC analysis) to confirm electrolyte chemical robustness.

Response: To evaluate the long-term chemical stability, we performed a comprehensive post-mortem study of the cathode, anode and electrolyte after extended cycling. As shown in Fig. R7a, the X-ray diffraction (XRD) patterns of iodine cathodes after various cycles remain nearly identical to that of the pristine electrode, indicating excellent structural stability of the cathode in the KA-PA-Nuc electrolyte environment. The corresponding SEM images reveal no noticeable morphological changes, and EDS mappings confirm a uniform distribution of C and I elements throughout cycling, further confirming the robustness of the iodine cathode (Fig. R9).

For the Zn anode, an additional diffraction peak at approximately 7.5° appears after 10 cycles and stabilizes after 40 cycles (Fig. R7b). This peak is attributed to $Zn_4SO_4(OH)_6 \cdot xH_2O$, a commonly observed and inevitable interfacial product for aqueous zinc ion batteries with $ZnSO_4$ containing electrolytes. The stabilization of this interfacial product after the initial cycles suggests that the Zn anode attains a steady-state interface, maintaining good stability during prolonged cycling. Consistently, SEM images show a smooth, dendrite-free surface, further confirming the stability of the Zn anode in the KA-PA-Nuc electrolyte environment. (Fig. R8).

Moreover, FTIR spectra of the separator containing electrolyte exhibits only the characteristic peaks of the individual glass fiber separator and the electrolyte

components, with no new peaks emerging during cycling (Fig. R7c). This result aligns with the LSV data (Fig. R1), further confirming the compatibility of the KA-PA-Nuc electrolyte with the working potential of the four-electron iodine conversion reactions. Overall, these complementary *ex situ* analyses confirm the structural and chemical robustness of the cathode, anode, and electrolyte during long-term cycling, which collectively underpin the excellent cycling stability of the batteries. The corresponding data and discussion have been incorporated into the revised manuscript: "*Post-mortem analyses confirm the structural and chemical stability of the cathode, anode, and electrolyte after prolonged cycling, further validating the exceptional longevity of the system (Supplementary Fig. 55-57)*".

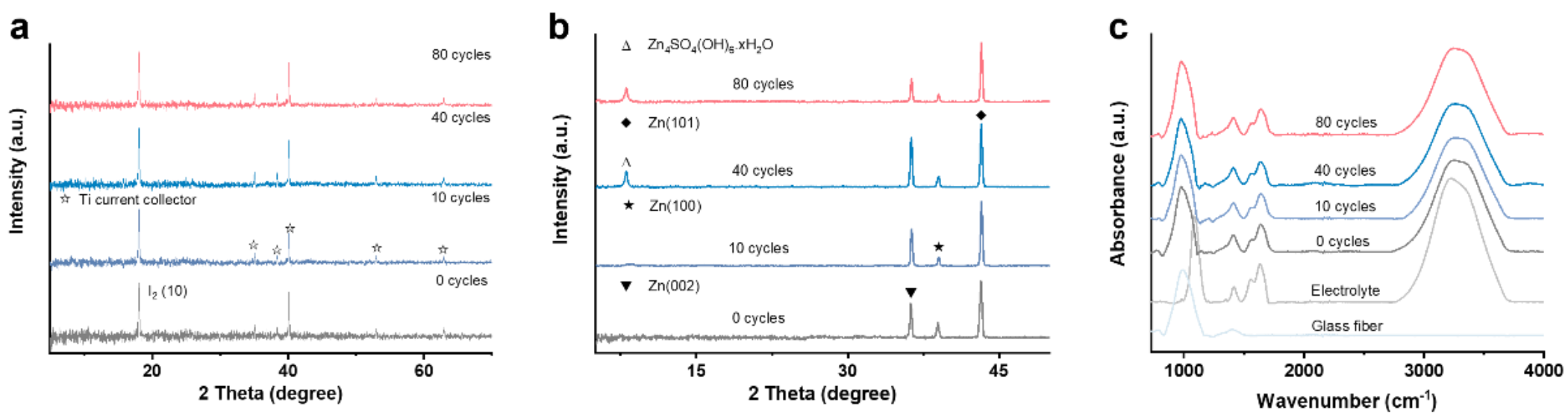

Fig. R7 Post-mortem analysis of cycled batteries. a, XRD patterns of iodine cathodes after various cycles. b, XRD patterns of Zn anodes after various cycles. c, FTIR spectra of KA-PA-NA electrolytes after various cycles.

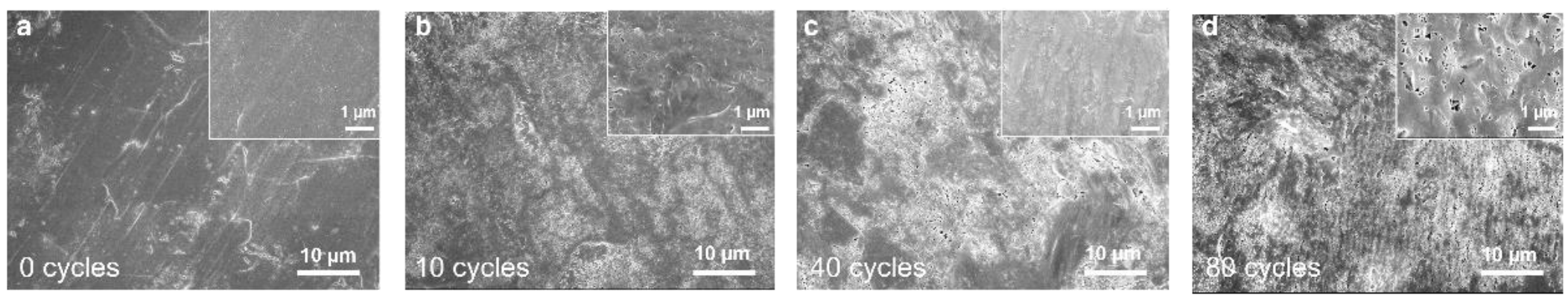

Fig. R8 Post-mortem analysis of cycled Zn anodes. a, SEM image of the pristine Zn anode. b-d, SEM images of Zn anodes after 10, 40, 80 cycles, respectively.

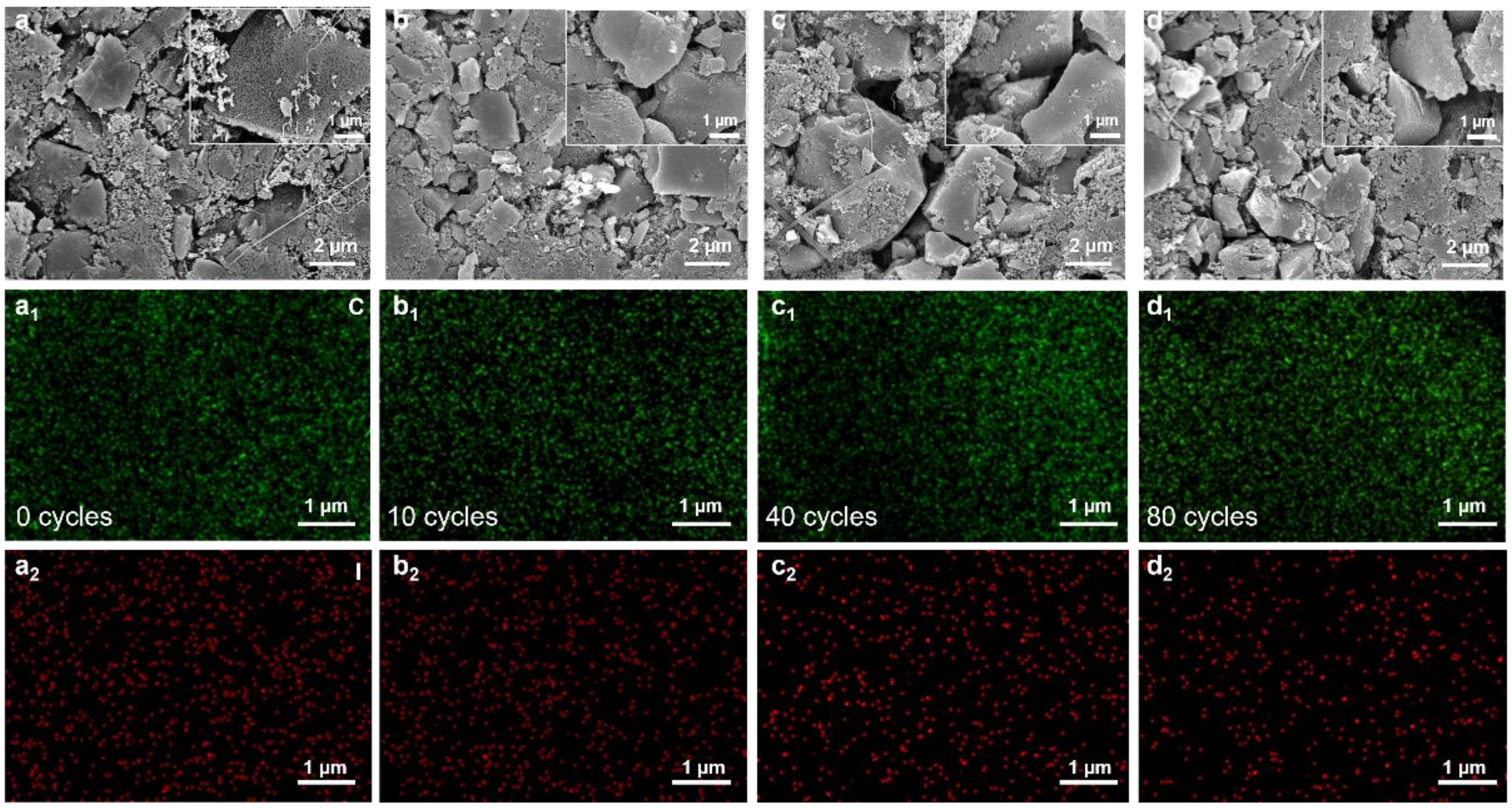

Fig. R9 Post-mortem analysis of cycled iodine cathodes. a, SEM image of the pristine iodine cathode. b-d, SEM images of iodine cathodes after 10, 40, 80 cycles, respectively. a_1, EDS mapping of the C element for the pristine iodine cathode. b_1-d_1, EDS mappings of the C element for iodine cathodes after 10, 40, 80 cycles, respectively. a_2, EDS mapping of the I element for the pristine iodine cathode. b_2-d_2, EDS mappings of the I element for iodine cathodes after 10, 40, 80 cycles, respectively.

Comment 4. Hydrogen bonding model in KA effect: The MD simulations and NMR shifts provide qualitative support for water-Nuc hydrogen bond disruption by kosmotropic anions. However, a more quantitative correlation (e.g., hydrogen bond lifetime from MD or hydration energy data) would further solidify this conclusion.

Response: In our original submission, NMR spectroscopy and MD simulations collectively demonstrated the molecular-level interactions among anions, water molecules, and Nuc species. These results underscore the critical role of kosmotropic anions, particularly SO_4^{2-}, in disrupting water-Nuc hydrogen bonds and stabilizing four-electron iodine conversion reactions. To further provide a quantitative correlation, hydrogen bond numbers between Nuc and water molecules in electrolytes with different anions were simulated. As shown in Fig. R10b, hydrogen bond numbers in electrolytes

containing kosmotropic anions reveal different trends compared with electrolytes with chaotropic anions. Chaotropic anions increase the number of water-Nuc hydrogen bonds, whereas kosmotropic anions markedly reduce them. This reduction clearly indicates the ability of kosmotropic anions to disrupt water–Nuc interactions. Among all examined anions, SO_4^{2-} exhibits the most pronounced effect, which is consistent with both the NMR data and the previous MD results (Fig. R10a). These results thus provide quantitative validation for the hydrogen bond disruption mechanism induced by kosmotropic anions, complementing the NMR and MD results presented earlier. Therefore, corresponding data and discussion have been incorporated into the revised manuscript: "*MD simulations of the N-H distance and the hydrogen bond numbers between Nuc and water molecules also confirmed that kosmotropic anions significantly reduced water-Nuc interactions, highlighting their critical role in modulating molecular interactions (Supplementary Fig. 38)*".

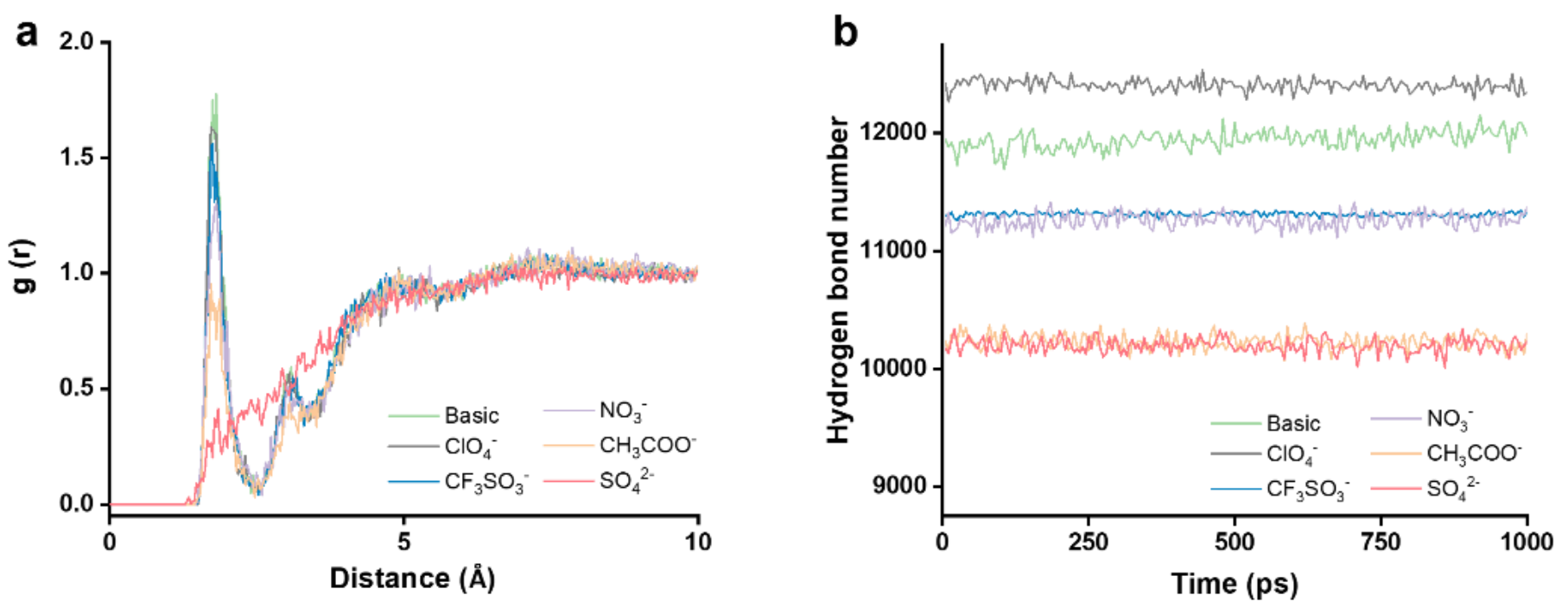

Fig. R10 Theoretical calculations of water-Nuc interactions in electrolytes with different anions. a, The radial distribution function of N-H distance between Nuc and water molecules in the presence of different anions. b, Simulated hydrogen bond numbers between Nuc and water molecules in the presence of different anions.

Comment 5. Compatibility with commercial current collectors: Given the practical

orientation of the work, the use of titanium mesh is reasonable. Still, a brief discussion on the compatibility of the proposed electrolyte with cheaper stainless steel or carbon-based current collectors would be beneficial for readers.

Response: According to the suggestion, commonly used stainless steel and carbon paper were employed as representative commercial current collectors to further evaluate the compatibility of the PA-KA-Nuc electrolyte.

Firstly, the electrochemical performance of the Zn–I_2 batteries using carbon paper as the cathode current collector was systematically investigated. As shown in Fig R11a-b, the cell in the KA–PA–Nuc electrolyte exhibits reversible four-electron iodine conversion reactions and excellent rate capability, delivering reversible capacities of 504, 458, 418, 370, 335, and 310 mAh g^{-1} at current densities of 0.5, 1, 2, 4, 6, and 8 A g^{-1}, respectively. Remarkably, the battery retained 86% of its capacity after 1,000 cycles at 1 A g^{-1}, indicating an outstanding cycling stability (Fig R11d). These results confirm the good compatibility of the KA–PA–Nuc electrolyte with carbon-based current collectors. In contrast, cells using carbon paper in the high-concentration, halogen-containing electrolyte (30m $ZnCl_2$) failed to complete even one full cycle due to continuous corrosion of battery cases (Fig. R12a). Persistent corrosion was also observed in the low-concentration, halogen-containing electrolyte (1.5$ZnCl_2$-0.5PA-0.4Nuc), leading to rapid cell failure (Fig. R12d).

Stainless steel, another inexpensive and widely used current collector, was also investigated. When employed as the cathode current collector, the battery in the KA–PA–Nuc electrolyte also displays excellent four-electron iodine conversion reactions, delivering reversible capacities of 501, 435, 380, 326, 293, and 270 mAh g^{-1} at current densities of 0.5, 1, 2, 4, 6, and 8 A g^{-1}, respectively (Fig R11c, e). In addition, a high capacity retention of 87% was maintained after 1,000 cycles at a current density of 1 A g^{-1} (Fig R11f). These results further prove the good compatibility of the KA–PA–Nuc electrolyte with stainless steel. However, batteries with stainless steel failed to operate properly in both high-concentration (30m $ZnCl_2$) and low-concentration halogen-

containing electrolytes (1.5$ZnCl_2$-0.5PA-0.4Nuc) due to severe corrosion of both battery cases and stainless steel current collector, even when tested in customized Swagelok cell configurations (Fig. R12b-c, e-f).

Collectively, these findings demonstrate that the KA–PA–Nuc electrolyte exhibits superior compatibility with various commercial current collectors, including carbon-based and stainless steel. In contrast to halogen-containing electrolytes, the KA-PA-Nuc electrolyte eliminates the dependence of specialized cell configurations and expensive titanium current collectors, thereby highlighting its immense economic benefit and broad applicability for cost-effective and sustainable energy storage solutions. Therefore, corresponding data and discussion have been incorporated into the revised manuscript: "*To assess the practical advantages of the KA-PA-Nuc electrolyte, its compatibility with other commercial current collectors was evaluated. In sharp contrast to halogen-containing electrolytes, which induce severe corrosion and rapid cell failure, the KA–PA–Nuc electrolyte exhibits excellent compatibility with various commercial current collectors (including stainless steel) without compromising electrochemical performance. This compatibility eliminates the reliance for specialized cell configurations and expensive titanium current collectors (Supplementary Fig. 58-59)*".

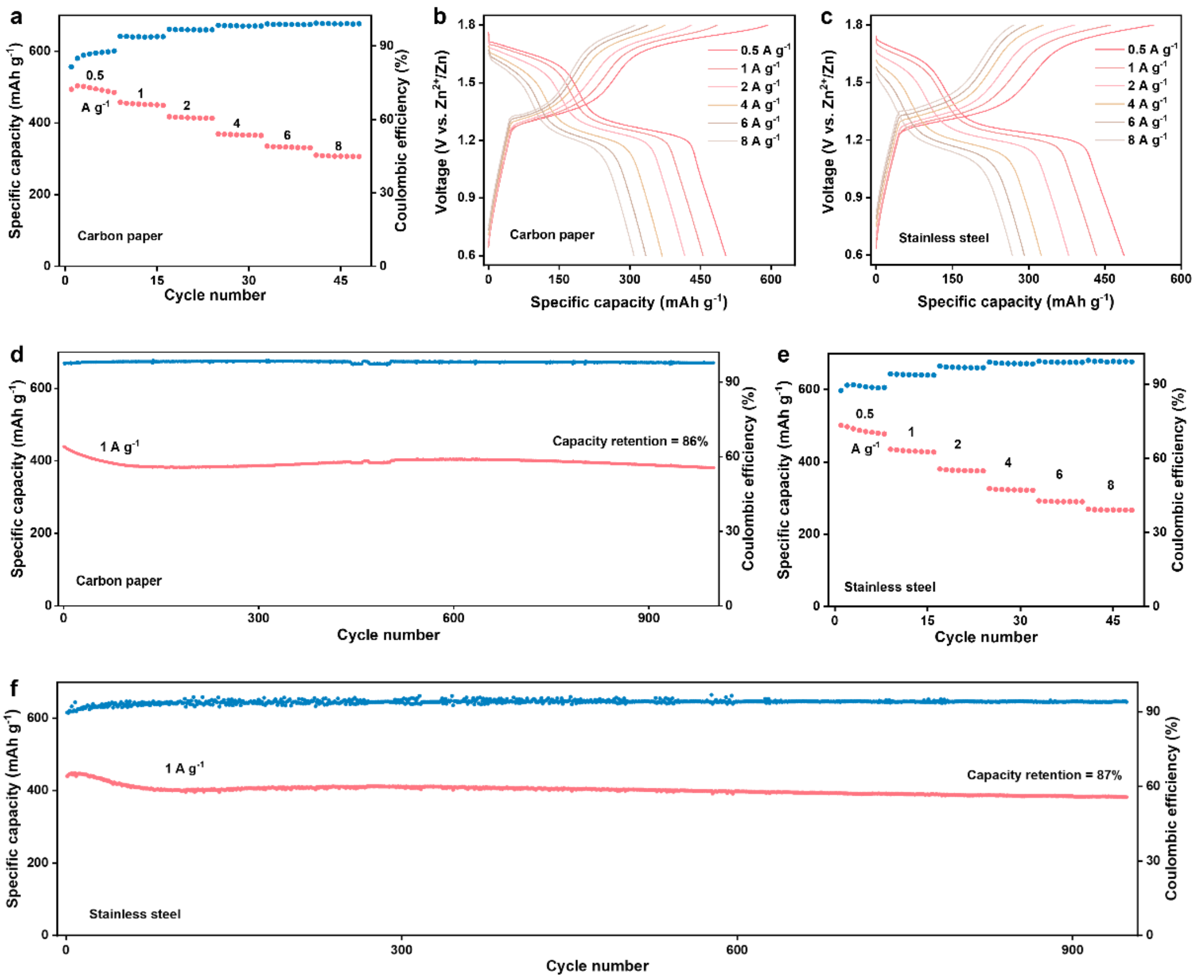

Fig. R11 Compatibility of the KA–PA–Nuc electrolyte with different commercial current collectors. a, Rate performance of Zn-I_2 batteries using carbon paper as the current collector. b, Corresponding GCD profiles of Zn-I_2 batteries using carbon paper as the current collector. c, GCD profiles of Zn-I_2 batteries using stainless steel as the current collector. d, Cycling performance of Zn-I_2 batteries using carbon paper as the current collector. e, Rate performance of Zn-I_2 batteries using stainless steel as the current collector. f, Cycling performance of Zn-I_2 batteries using stainless steel as the current collector.

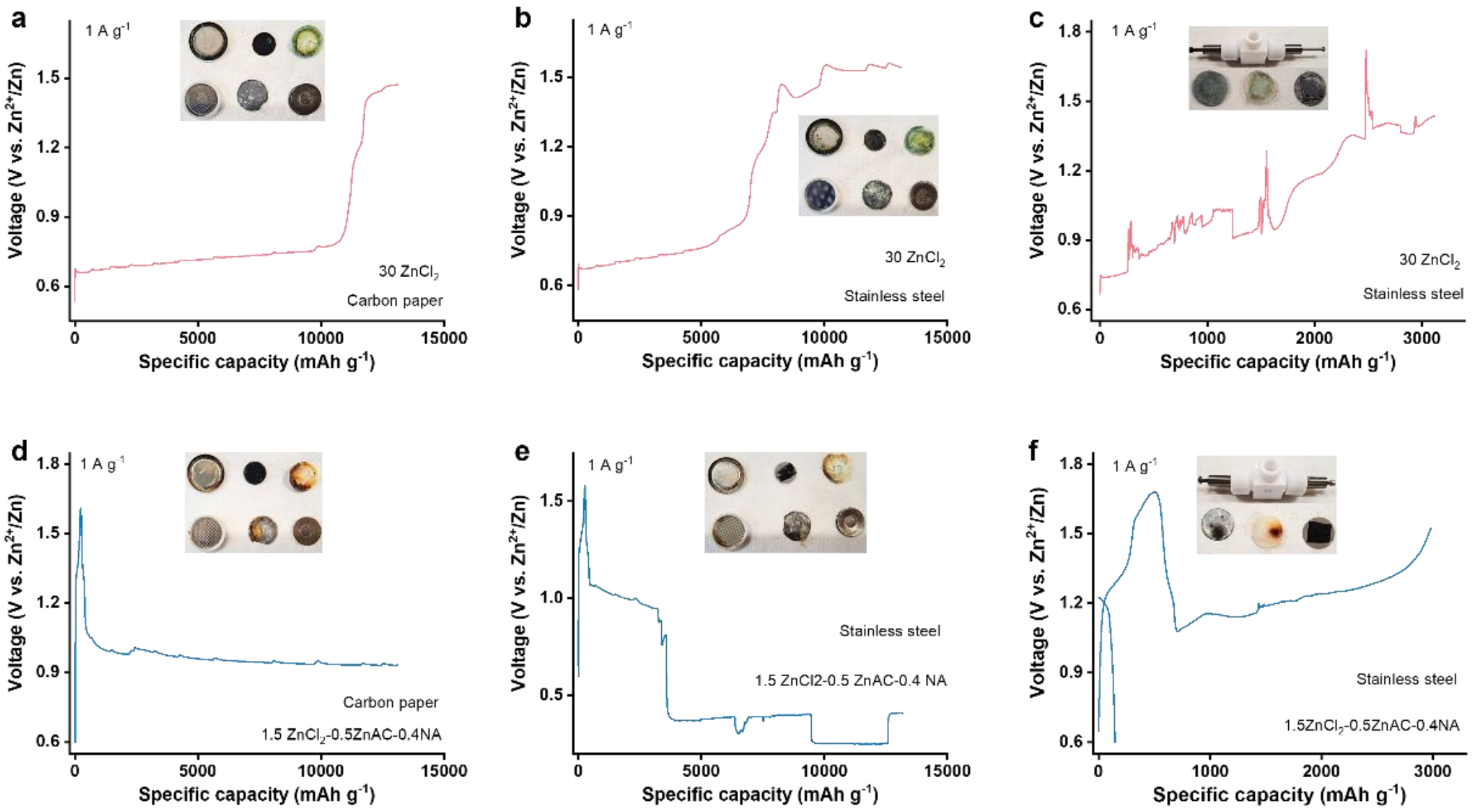

Fig. R12 Electrochemical behavior of different commercial current collectors in halogen-containing electrolytes. a, GCD profiles of Zn-I_2 batteries with 30m $ZnCl_2$ electrolyte using carbon paper as the current collector in the coin cell configuration. b, GCD profiles of Zn-I_2 batteries with 30m $ZnCl_2$ electrolyte using stainless steel as the current collector in the coin cell configuration. c, GCD profiles of Zn-I_2 batteries with 30m $ZnCl_2$ electrolyte using stainless steel as the current collector in the Swagelok-cell configuration. d, GCD profiles of Zn-I_2 batteries with 1.5$ZnCl_2$-0.5PA-0.4Nuc electrolyte using carbon paper as the current collector in the coin cell configuration. e, GCD profiles of Zn-I_2 batteries with 1.5$ZnCl_2$-0.5PA-0.4Nuc electrolyte using stainless steel as the current collector in the coin cell configuration. f, GCD profiles of Zn-I_2 batteries with 1.5$ZnCl_2$-0.5PA-0.4Nuc electrolyte using stainless steel as the current collector in the Swagelok-cell configuration. Insets show photographs of the corresponding cycled batteries.

Comment 6. Rate-limiting step identification: In the DRT and in situ Raman analyses, the evolution of τ_3/τ_4 peaks suggests potential bottlenecks at high voltages. Could the authors elaborate on whether ion transport or interfacial charge transfer becomes dominant under fast charging?

Response: We sincerely appreciate the reviewer's insightful and professional comment concerning the rate-limiting step identification, which is indeed crucial for elucidating the underlying fast-charging mechanism—an aspect that has long remained ambiguous in aqueous batteries. To resolve this important question, *in situ* EIS measurements coupled with in-depth DRT analysis using a three-electrode setup were performed at different charging rates. This setup enables the decoupling of individual electrode responses, so that the properties of each electrode and their contribution to the overall performance of the battery can be investigated individually. Leveraging this methodology, EIS curves and corresponding DRT plots of the two-electrode full cell, individual iodine cathode, and Zn anode under different charging rates were obtained (Fig. R14-19).

All DRT plots exhibit six distinct relaxation peaks associated with different electrochemical processes. As detailed in the response to Referee #2's comment 11, these peaks were systematically assigned to specific processes: intrinsic ohmic resistance (R_s-τ_1), SEI resistance (R_{sei}-τ_2), charge transfer resistance (R_{ct}-$\tau_{3\text{-}4}$), and ion diffusion resistance (R_{id}-$\tau_{5\text{-}6}$). As shown in Fig. R14-19, the relative contributions of these four electrochemical processes vary across different voltages and charging rates. To quantitatively assess these variations, the peak areas and corresponding area percentages were fitted and summarized (Fig. R13d-f). For the Zn anode, R_{ct} consistently dominates the total impedance across all voltages and charging rates, indicating that Zn deposition is primarily governed by charge transfer kinetics (Fig. R13c and Fig. R18-19). Although R_{id} slightly increases at higher charging rates due to enhanced mass transport limitations, R_{ct} remains the decisive step controlling the overall reaction kinetics. In contrast, the iodine cathode exhibits distinct behavior: R_{ct} dominates within the redox regions, whereas R_{id} becomes more significant in non-redox voltage ranges (Fig. R13b and Fig. R16-17). Therefore, the iodine conversion reaction is the charge transfer governed electrochemical process. Consequently, for the two-electrode full cell, the overall rate-limiting step can be attributed to charge transfer at both electrodes (Fig. R13a and Fig. R14-15). The impedance evolution of the full cell

closely mirrors that of the Zn anode, further supporting that the total cell resistance is predominantly governed by the anode.

In summary, comprehensive *in situ* EIS–DRT analyses conclusively demonstrate that the charge-transfer process, rather than ion transport, governs the rate-limiting step under both moderate and fast charging conditions. This mechanistic clarification fills a long-standing knowledge gap in aqueous battery research, where the nature of the kinetic bottleneck has remained uncertain. Although mass-transport impedance slightly increases at high rates, its contribution remains secondary due to the intrinsically high ionic conductivity of our low-concentration aqueous electrolyte. Both the iodine conversion at the cathode and zinc deposition at the anode involve multi-step electron transfer processes, reinforcing that the overall rate limitation originates from charge-transfer kinetics rather than bulk ion diffusion. This finding provides a crucial mechanistic insight of broad relevance to aqueous systems coupling metal anodes with conversion-type cathodes. It suggests that more attention should focus on accelerating interfacial electron transfer rather than solely improving ionic transport. By clearly identifying the rate-limiting step, this work also establishes a mechanistic foundation and strategic direction for designing fast-charging aqueous metal batteries beyond the metal–iodine chemistry. The relevant discussion and additional figures have been incorporated into the revised manuscript to clarify these findings: "*Further EIS–DRT analysis reveals that charge transfer is the rate-limiting step governing interfacial kinetics, providing the mechanistic guidance for the design of fast-charging aqueous metal–iodine batteries (Supplementary Fig. 42-48)*".

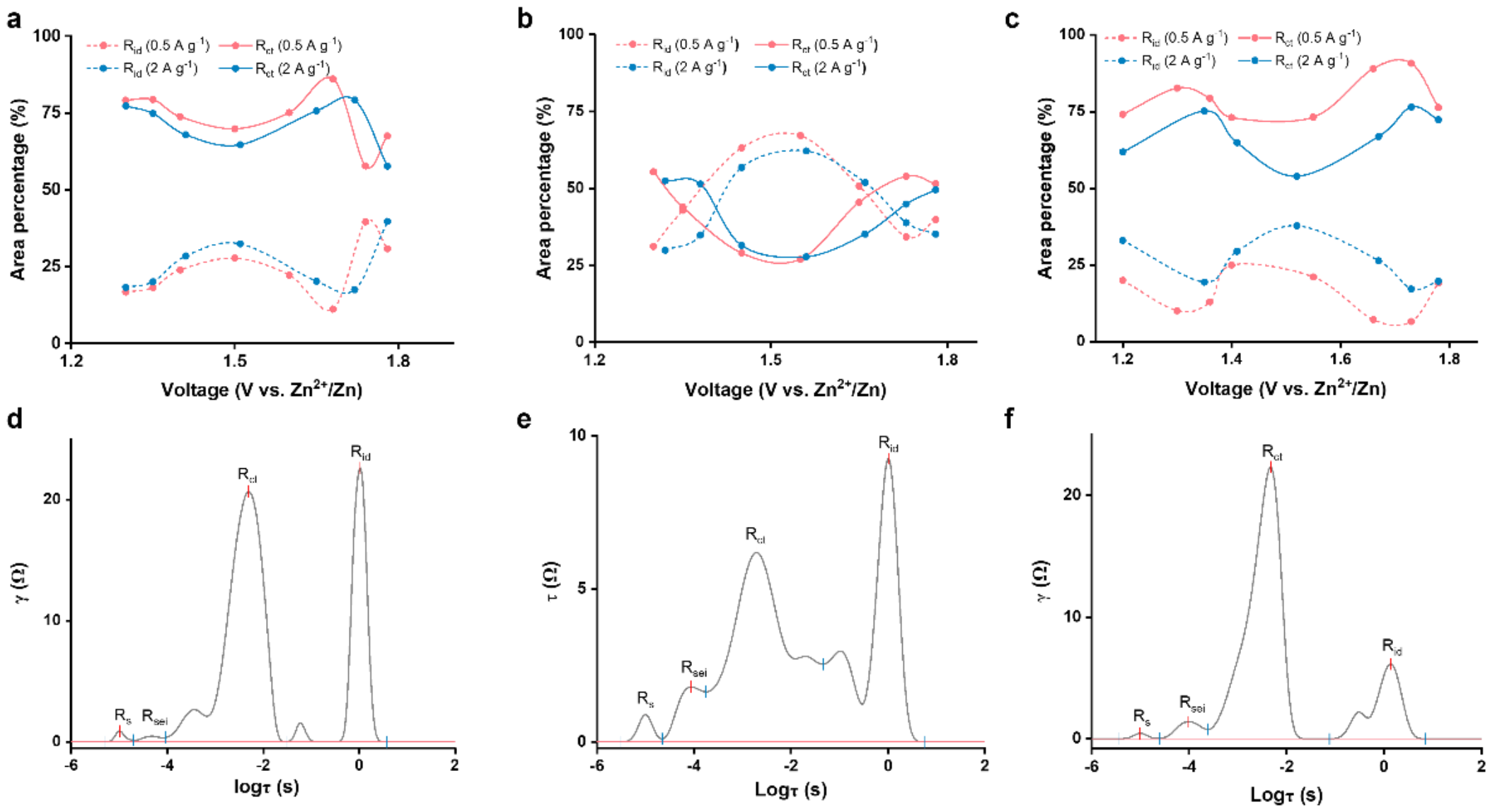

Fig. R13 Identification of the rate-limiting step. a, Fitted charge transfer resistance (R_{ct}) and ion diffusion resistance (R_{id}) at different charging rates for the two-electrode full cell (Zn-I_2). b, Fitted R_{ct} and R_{id} at different charging rates for the individual iodine cathode. c, Fitted R_{ct} and R_{id} at different charging rates for the individual Zn anode. d, Demonstration of the fitting process based on DRT results for the two-electrode full cell. e, Demonstration of the fitting process based on DRT results for the individual iodine cathode. f, Demonstration of the fitting process based on DRT results for the individual Zn anode. The EIS data were processed using DRT analysis, and the impedance contributions of different electrochemical processes (R_s, R_{sei}, R_{ct}, and R_{id}) were quantified by integrating the corresponding peak areas.

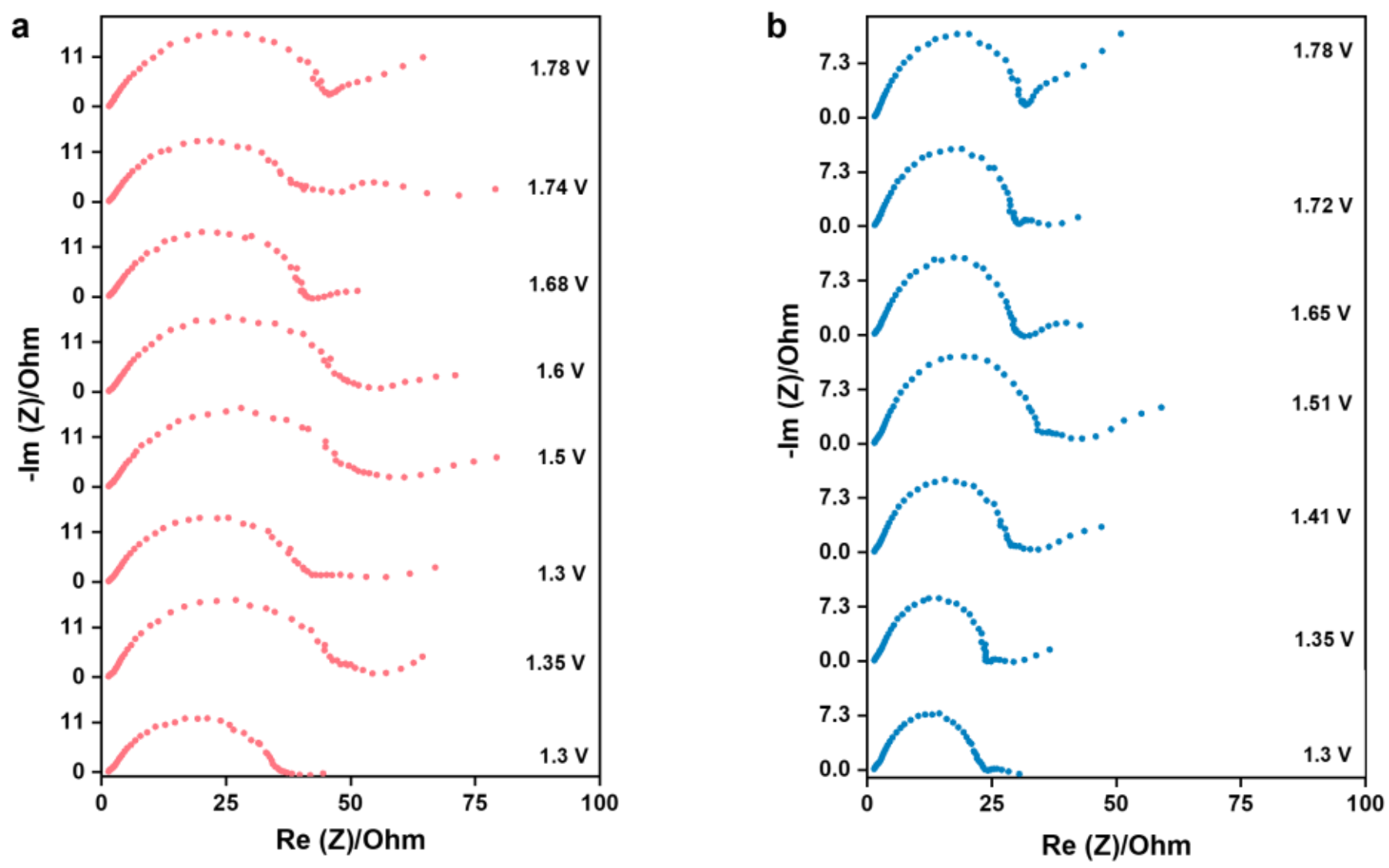

Fig. R14 *In situ* EIS profiles of the two-electrode full cell at different charging rates. a, 0.5 A g^{-1}. b, 2 A g^{-1}.

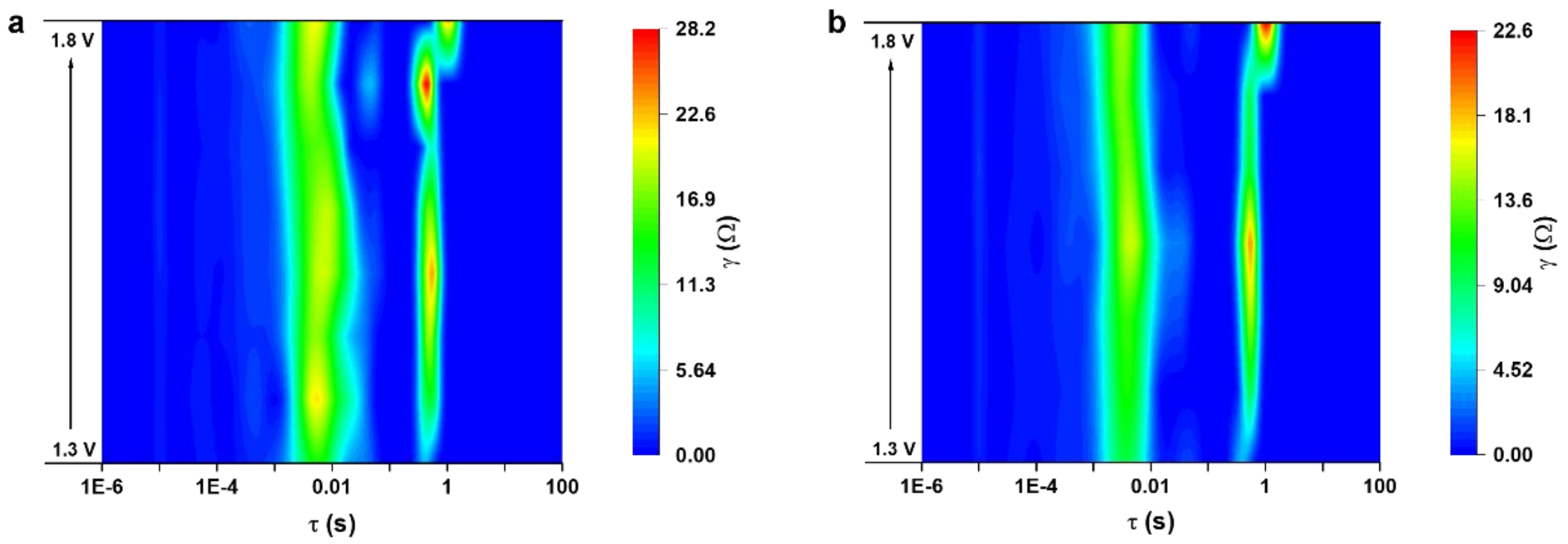

Fig. R15 Corresponding DRT spectra of the two-electrode full cell at different charging rates. a, 0.5 A g^{-1}. b, 2 A g^{-1}.

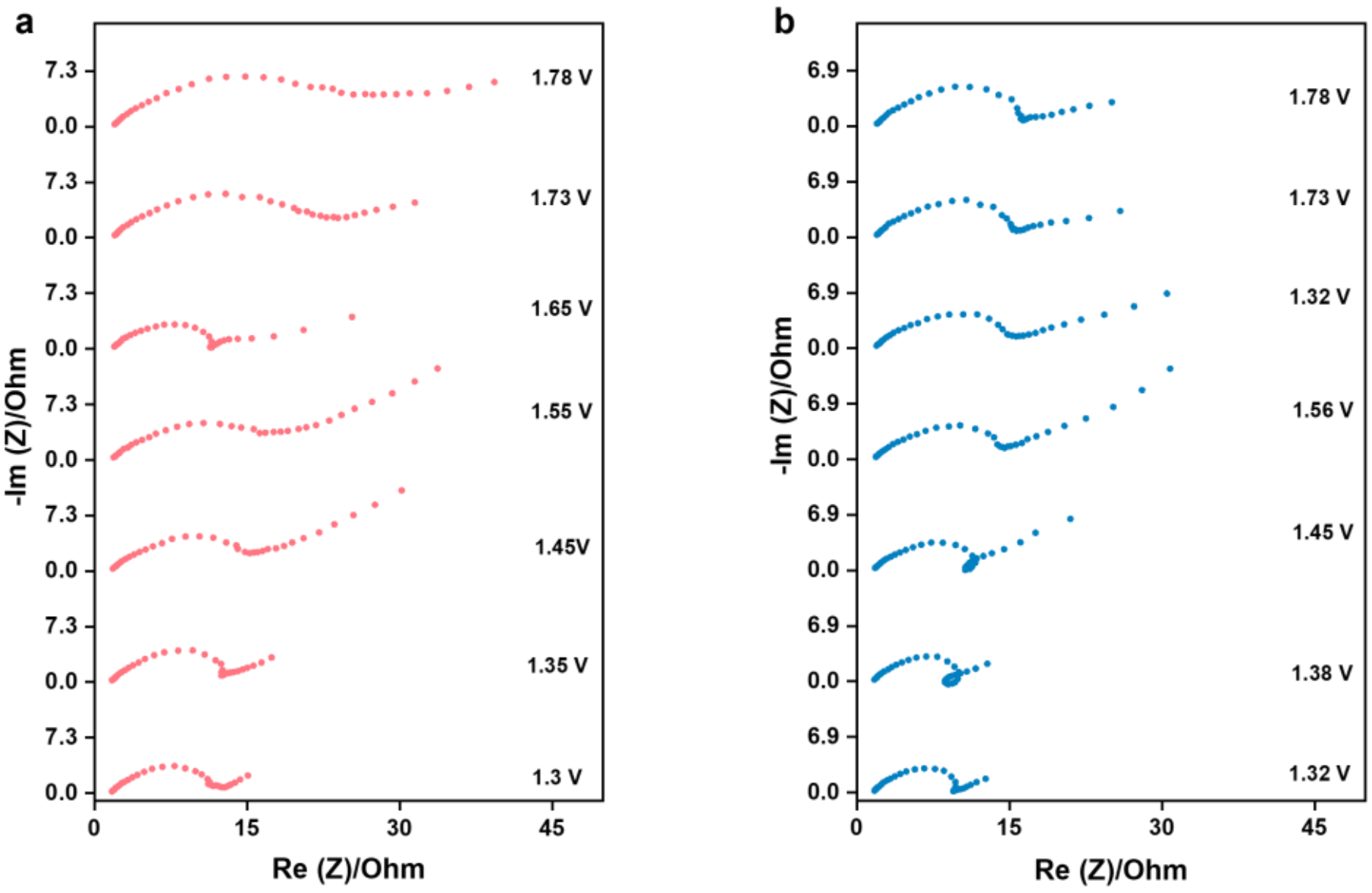

Fig. R16 ***In situ*** **EIS profiles of the individual iodine cathode at different charging rates**. a, 0.5 A g^{-1}. b, 2 A g^{-1}.

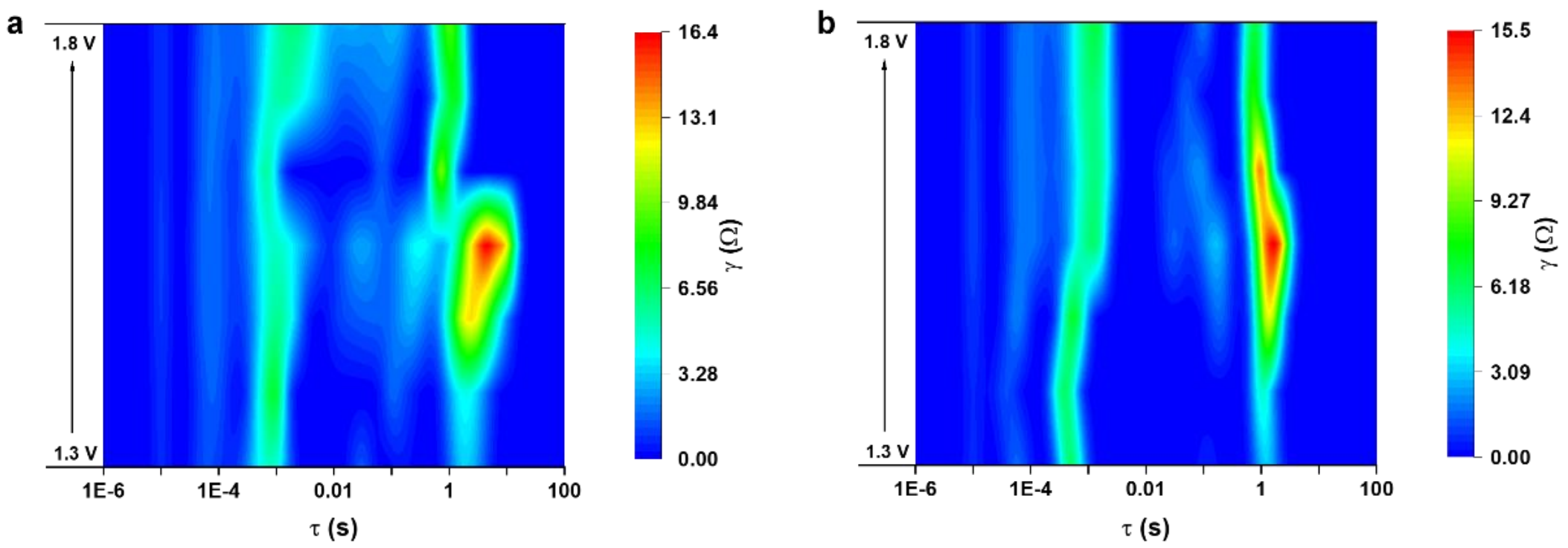

Fig. R17 Corresponding DRT spectra of the individual iodine cathode at different charging rates. a, 0.5 A g^{-1}. b, 2 A g^{-1}.

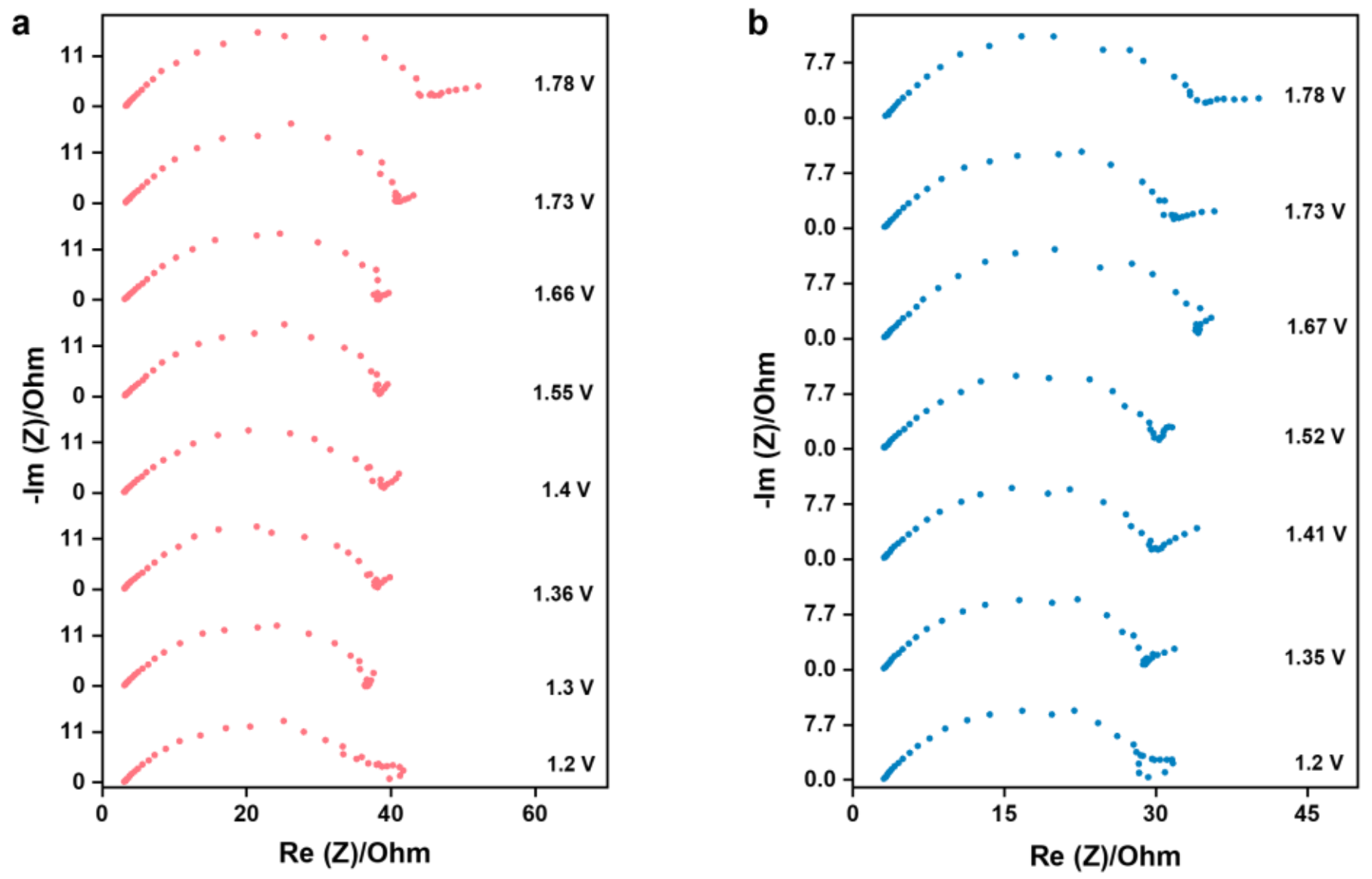

Fig. R18 *In situ* EIS profiles of the individual Zn anode at different charging rates. a, 0.5 A g^{-1}. b, 2 A g^{-1}.

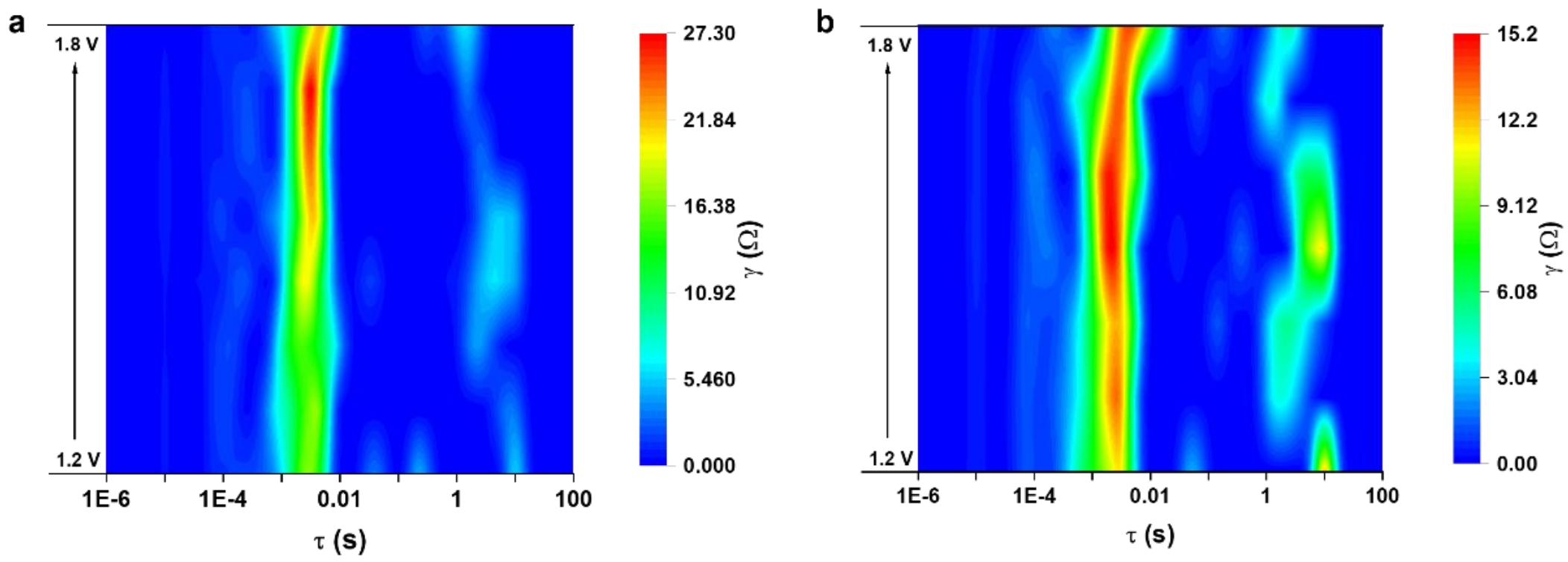

Fig. R19 Corresponding DRT spectra of the individual Zn anode at different charging rates. a, 0.5 A g^{-1}. b, 2 A g^{-1}.

Minor Points

Line 33: "provides a new avenue" → consider specifying: "a new electrolyte design avenue"

Response: We have revised the sentence accordingly.

Line 112: “this work establishes a new paradigm” → consider softening to “this work introduces a new design paradigm”

Response: We have revised this sentence in the revised manuscript.

Figures: Please ensure color consistency for KA-only, PA-only, and KA–PA curves across CV, GCD, and DRT plots.

Response: We appreciate this important comment. We have carefully reviewed all figures and have ensured consistent use of colors for the KA-only, PA-only, and KA–PA electrolytes across the CV, GCD, and DRT plots in the revised manuscript.

Figure 6d-e: Units in cost breakdown charts could be labeled more explicitly (USD/kWh)

Response: We have updated Figure 6d–e to label the units (USD/kWh) more explicitly in the revised manuscript.

A table summarizing electrolyte compositions and corresponding performance (capacity, retention, redox reversibility) would help readers.

Response: In the original manuscript, we already included a table (Table S2) summarizing the electrolyte compositions and their corresponding performance metrics (capacity, retention, and redox reversibility).

Weaknesses (relatively minor):

Lacks direct quantification of halogen bonding in some systems

Deeper insight into ESW and long-term electrolyte integrity would be beneficial

Response: In the revised manuscript, we have added a more comprehensive analysis of the halogen bond mechanism as well as an expanded discussion of the electrochemical stability window and long-term electrolyte integrity. Please refer to our detailed responses to Comments 1, 2, and 3 for further information.

Referee #2

This manuscript presents an electrolyte design strategy aimed at achieving the reversible four-electron reaction of iodine-based cathode materials. It tries to establish a fundamental compositional framework comprising kosmotropic anions, polar anions, and nucleophilic species. While the study addresses a relevant and timely topic, the rationale underlying some of the key conclusions appears to be somewhat overstated when evaluated in terms of robustness, validity, and reliability. The conclusions would benefit from a more cautious and data-driven interpretation, with closer alignment to the experimental evidence. To enhance the logical coherence and scientific rigor of the manuscript, further clarification and justification of several aspects of the experimental interpretation are recommended.

Response: We sincerely thank the reviewer for reviewing our manuscript. We greatly appreciate your insightful comments and constructive suggestions, which have helped us further strengthen the scientific rigor and clarity of the work. In the revised manuscript, we have carefully addressed all concerns and reinforced the data-driven support for our conclusions. Specifically, we have substantially clarified and justified the robustness of the proposed KA–PA–Nuc electrolyte standard by demonstrating its broad applicability across multiple nucleophilic species (urea, acetamide, pyridine, and nicotinamide) and in both Zn–I_2 and Al–I_2 battery systems. Furthermore, the key conclusions regarding the halogen bond stabilization mechanism and the synergistic contributions of polar and kosmotropic anions are comprehensively validated by electrochemical measurements, spectroscopic characterizations (including *in situ* ATR–IR, *in situ* UV–Vis, NMR, and *in situ* ESI–DRT), and theoretical calculations. In addition, all points requiring further clarification or additional experimental evidence have been addressed to enhance logical coherence and reliability of the interpretations. Additional clarification has also been added regarding the electrolyte's compatibility with various current collectors and its performance under practical high mass loading, further highlighting the reliability and applicability of the KA–PA–Nuc standard.

Detailed point-by-point responses to all comments are provided below. We hope that the revised version meets your expectations.

Comment 1. In Figures 2b–2g, the authors compare the reversibility of the I^0/I^+ redox couple using various electrolyte combinations. However, this experimental design appears to rely on the underlying assumption that I^+ is stabilized predominantly through complexation with nucleophilic species (i.e., forming a Nuc-I-Nuc complex). It should be noted, however, that I^+ may also be stabilized via complexation with other anions present in the electrolyte, rather than exclusively by nucleophiles. To clearly rule out this alternative pathway, it is recommended that the authors include CV and GCD data for the KA-PA electrolyte system, which does not contain nucleophilic species.

Response: We fully agree that I^+ may also be stabilized via complexation with anions in our system and this concern needs further clarification. To evaluate this possibility, electrochemical performance of electrolytes without Nuc species was comprehensively studied according to the suggestion. As shown in Fig. R2a, the CV profiles of the PA electrolyte (without Nuc) exhibit a single redox peak corresponding to the I^-/I^0 conversion reaction, followed by a sharp, irreversible current increase at higher potentials. This behavior indicates that I^+ cannot be stabilized through complexation with PA. In GCD profiles (Fig. R2d), only one pair of charge–discharge plateaus are observed for the I^-/I^0 couple, while the I^0/I^+ region displays a long charging tail without a corresponding discharge plateau, which is consistent with the CV results. This irreversible feature is consistent with uncontrolled iodine and water oxidation processes, as detailed in our response to Comment 5.

Similarly, both KA and KA–PA electrolytes show analogous behavior (Fig. R2b-c, e-f), with no reduction peak or discharge plateau corresponding to the I^0/I^+ redox process. These consistent results across all electrolytes without Nuc confirm that anions alone cannot stabilize I^+ via direct complexation. Collectively, these findings demonstrate that the stabilization of I^+ arises predominantly from coordination with nucleophilic species (i.e., the formation of Nuc–I–Nuc complexes) rather than any

interaction with anions. This clarification and the supporting data have been incorporated into the revised manuscript: "*These halogen bonds are the foundation of four-electron iodine conversion reactions, which has been fully demonstrated through electrochemical comparison in Nuc-free electrolytes, nuclear magnetic resonance (NMR) spectroscopy, and theoretical calculations (Supplementary Fig. 6-10)*".

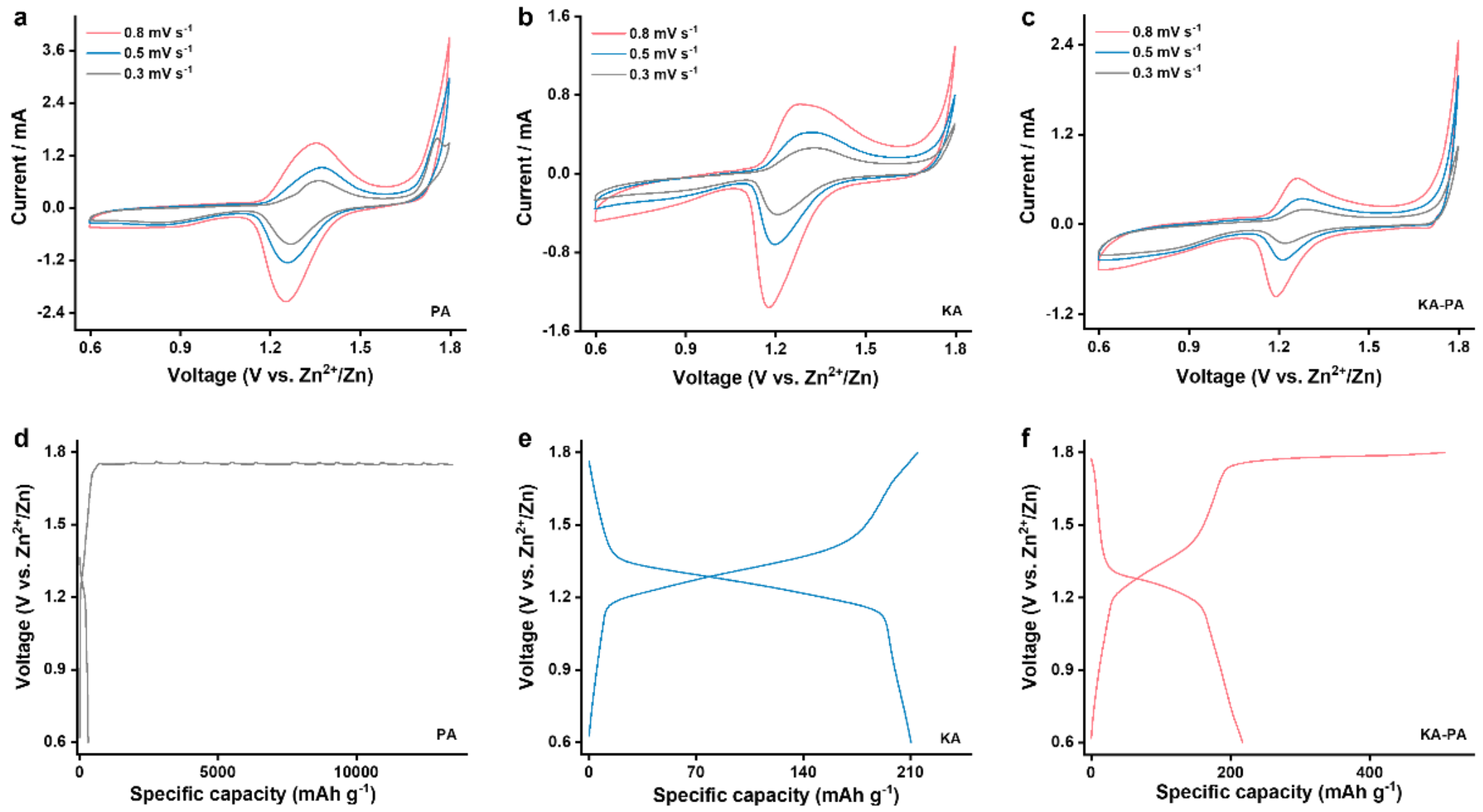

Fig. R2 Electrochemical performance of electrolytes without Nuc species. a-c, CV profiles of the iodine conversion reaction in PA, KA, and KA-PA electrolytes at various scan rates. d-f, GCD profiles of the iodine conversion reaction in PA, KA, and KA-PA electrolytes at 1 A g^{-1}.

Comment 2. The authors base their assumption of Nuc-I-Nuc complex formation on Ref. 21. However, in that reference, the molar ratio of pyridine derivatives is twice that of anions, whereas in the present study, anions are present in overwhelming excess. This discrepancy makes a direct comparison of complex formation behavior difficult. Therefore, the potential stabilization of I^+ by anions, especially polar ones, should be carefully considered.

Response: We agree that this discrepancy could raise questions and merits further

clarification. In Ref. 21, the reported $(N–I–N)^+$ complexes were obtained via a cation exchange reaction from corresponding silver analogues ($(N–Ag–N)^+$), where one cation (Ag^+ or I^+) coordinates with two pyridine derivatives. In that case, the 2:1 ratio between pyridine derivatives and anions arises solely from the charge balance in silver salts, rather than reflecting a mechanistic requirement for halogen bond formation.

To evaluate the possibility of I^+ stabilization by anions (A^-) in our system, we carried out a series of electrochemical, spectroscopic, and theoretical analyses. First, control electrolytes without nucleophilic species show no detectable I^+-related redox features (Fig. R2), demonstrating that anions alone do not support I^+ stabilization through direct complexation under our conditions (excluding $A^-–I^+–A^-$ configuration). By contrast, the presence of nucleophilic species enables successful I^+ conversion reaction, providing strong evidence that halogen bond interaction with Nuc species is essential for I^+ stabilization. To directly probe halogen bond interaction, 2D ^1H-^{15}N HMBC NMR was employed, which is a sensitive tool for investigation of halogen bond formation. In the bare pyridine solution, coupled H2/H6-N1 and H3/H5-N1 signals appear at 314.78 ppm (Fig. R3a). Upon addition of molecular iodine (I_2), a small ^{15}N chemical shift is observed (Δ_{15N} = -22.76 ppm), consistent with weak interaction between pyridine and iodine (Fig. R3b). In sharp contrast, when pyridine is exposed to iodonium (I^+) species generated by an exchange reaction with corresponding silver analogues (Fig. R6a), a large ^{15}N chemical shift (Δ_{15N} = -107.84 ppm) is detected (Fig. R3c). This magnitude of chemical shift is characteristic of halogen bond formation, confirming direct Nuc–I^+ interaction.

Theoretically, the I^+ cation accepts electron through its two empty p-orbitals and therefore typically adopts a two-coordinate geometry. Above electrochemical comparison and NMR analysis confirm the direct Nuc–I^+ interaction. However, given the composition of our electrolyte system, two coordination configurations remain possible: Nuc–I^+–A^- and Nuc–I^+–Nuc. To further identify the specific coordination configuration, binding energy calculations were conducted for these two possible

geometries. The results reveal that the linear Nuc–I^+–Nuc halogen bond, accompanied by a counterion, is the most stable structure, exhibiting lower binding energy than anion-involved configurations (A^-–I^+–A^- and Nuc–I^+–A^-) (Fig. R4). These findings indicate that the linear Nuc–I^+–Nuc halogen bond is thermodynamically preferred, while anions only serve as counterions and do not directly coordinate with I^+. Importantly, variations in counterions have negligible effects on N–I bond lengths and binding energies, further supporting their non-coordination in halogen bond formation (Fig. R5). This observation is fully corroborated by additional 2D ^1H-^{15}N HMBC spectra, where the ^{15}N chemical shifts of Nuc–I^+–Nuc complexes remain essentially unchanged across various counterions (Fig. R6). Collectively, these electrochemical, spectroscopic, and theoretical results demonstrate that the stabilization of I^+ arises from coordination with nucleophilic species via a linear Nuc–I^+–Nuc halogen bond, rather than through direct coordination with anions.

Although the total anions concentration in our electrolytes is much higher than that of the Nuc species, the amount of Nuc relative to iodine within the cathode remains overwhelmingly higher, ensuring sufficient Nuc availability for I^+ complexation and stabilization. Therefore, the fundamental stabilization mechanism remains unchanged. Meanwhile, anions play an indirect but indispensable role by modulating the interfacial environment: creating water-deficient, anion-enriched interface and tuning hydrogen bond networks and Nuc availability. The corresponding clarification and discussion about halogen bond stabilization mechanism have been incorporated into the revised manuscript: "*These halogen bonds are the foundation of four-electron iodine conversion reactions, which has been fully demonstrated through electrochemical comparison in Nuc-free electrolytes, nuclear magnetic resonance (NMR) spectroscopy, and theoretical calculations (Supplementary Fig. 6-10)*".

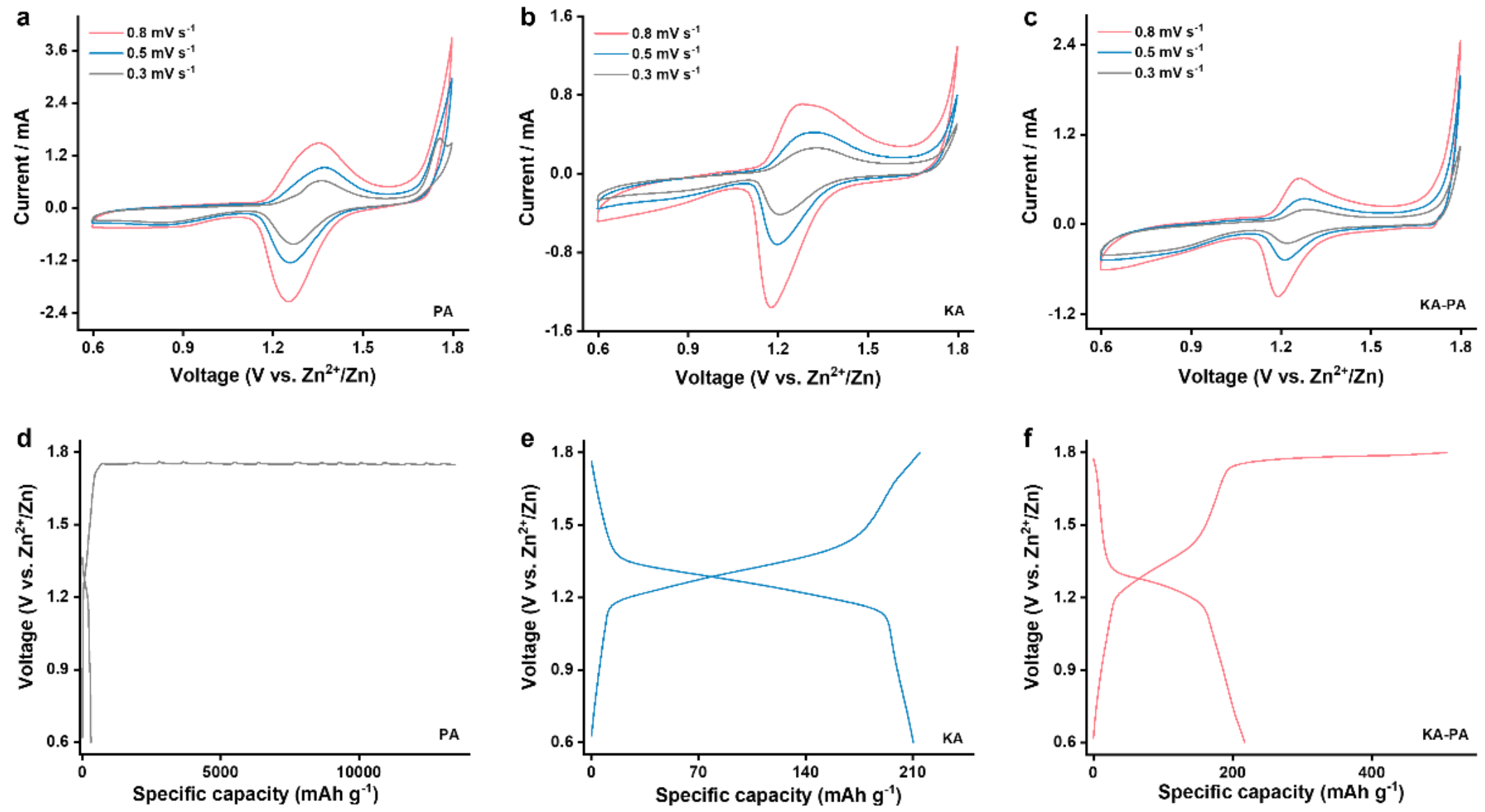

Fig. R2 Electrochemical performance of electrolytes without Nuc species. a-c, CV profiles of the iodine conversion reaction in PA, KA, and KA-PA electrolytes at various scan rates. d-f, GCD profiles of the iodine conversion reaction in PA, KA, and KA-PA electrolytes at 1 A g^{-1}.

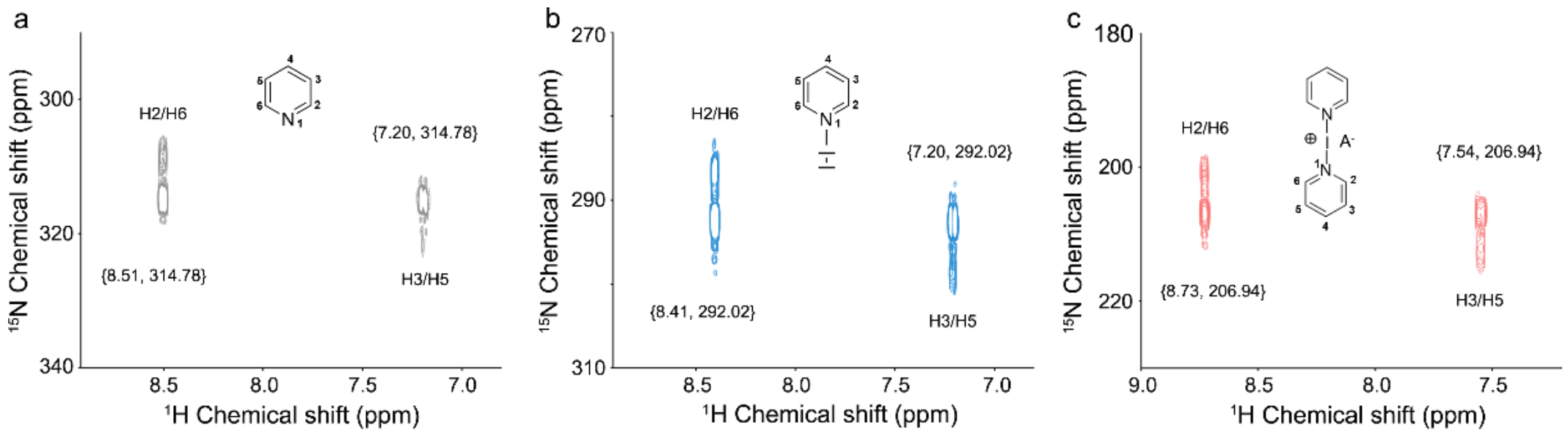

Fig. R3 Characterization of halogen bonds. a, 2D ^{1}H-^{15}N HMBC NMR spectrum of the bare Nuc solution (pyridine). b, 2D ^{1}H-^{15}N HMBC NMR spectrum of the pyridine solution with iodine. c, 2D ^{1}H-^{15}N HMBC NMR spectrum of the synthesized I^+ complexes.

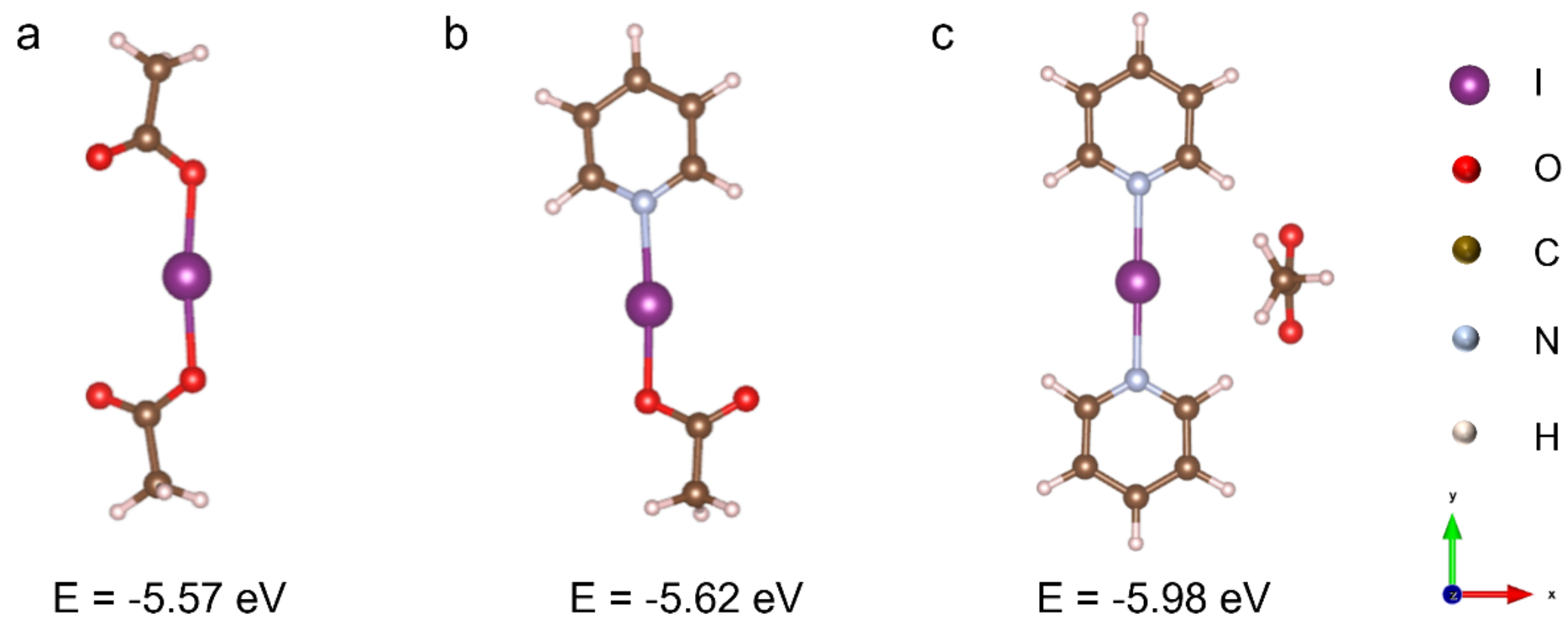

Fig. R4 Theoretical calculation of possible coordination configurations. a, The A^-–I^+–A^- coordination structure and corresponding binding energy. b, The Nuc–I^+–A^- coordination structure and corresponding binding energy. c, The Nuc–I^+–Nuc halogen bond and corresponding binding energy.

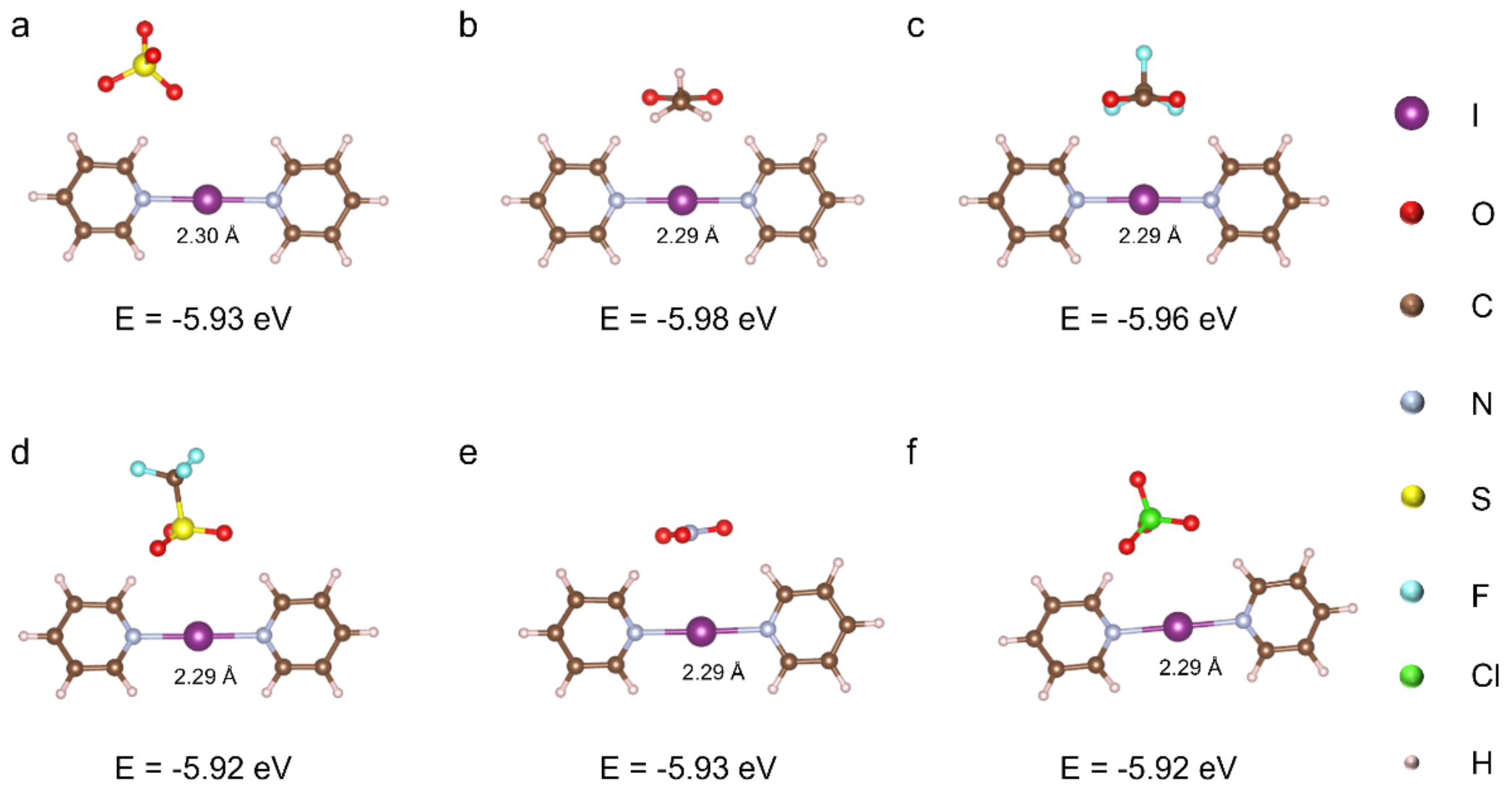

Fig. R5 Binding energy and bond length of Nuc–I^+–Nuc halogen bonds with different counterions. a, SO_4^{2-}. b, CH_3COO^-. c, CF_3COO^-. d, $CF_3SO_3^-$. e, NO_3^-. f, ClO_4^-.

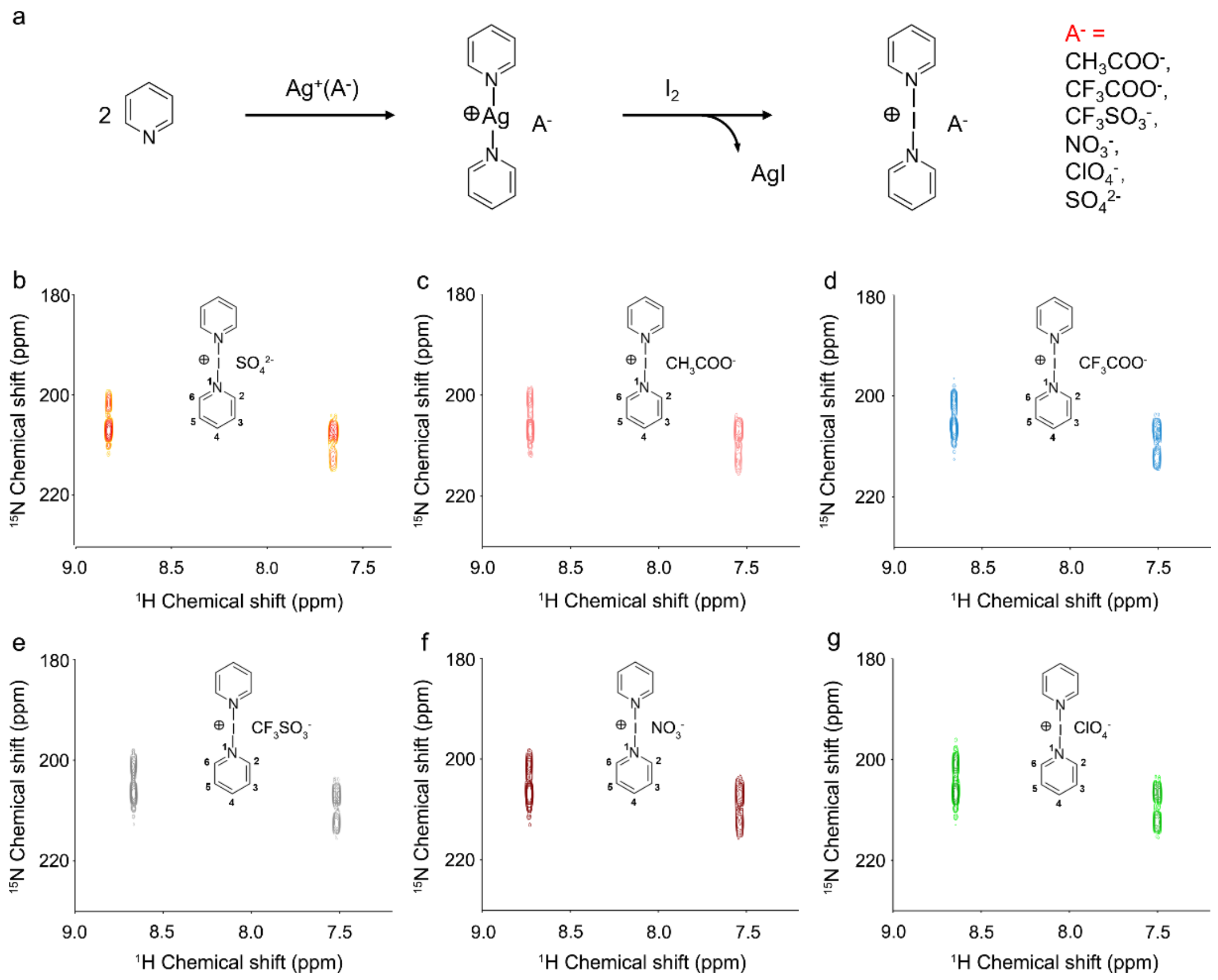

Fig. R6 Characterization of halogen bonds with different counterions. a, Schematic illustration of the synthesis of I^+ halogen bond complexes with different counterions via a cation exchange reaction. 2D ^1H-^{15}N HMBC NMR spectrum of the synthesized I^+ complexes with different counterions: b, SO_4^{2-}; c, CH_3COO^-; d, CF_3COO^-; e, $CF_3SO_3^-$; f, NO_3^-; g, ClO_4^-.

Comment 3. Additionally, the KA-PA-Nuc, PA-Nuc, and KA-Nuc electrolytes in Figure 2 appear to differ in total ionic concentration or ionic strength. Given that these parameters can substantially affect electrolyte conductivity and water molecule hydration, the reviewer recommends performing comparative experiments under standardized ionic strength to ensure more rigorous and interpretable results.

Response: We sincerely appreciate the reviewer's constructive comment regarding the potential influence of electrolyte ionic concentration and ionic strength. We agree that

variations in these parameters may raise concerns about the comparability of their electrochemical performance.

To rigorously assess this, comparative experiments were performed under a standardized ionic strength condition. The ionic strength of each electrolyte was calculated according to the equation: $I_i = \frac{1}{2}\sum_{i=1}^{n} C_i Z_i^2$, where C_i and Z_i represent the molar concentration and charge number of ion i, respectively. Taking 1.5m PA-Nuc as the baseline, the corresponding compositions of KA–Nuc and KA–PA–Nuc electrolytes with identical ionic strength were determined to be 1.125m KA-Nuc and 0.9m KA-0.3m PA-Nuc, respectively.

The electrochemical performance of these electrolytes was systematically studied and compared. Fig. R20a shows the CV profiles with urea (U) as the Nuc species, where the KA-U electrolyte only displays a single redox peak corresponding to the I^-/I^0 conversion reaction. In contrast, the PA-U electrolyte exhibits an additional but highly irreversible redox peak associated with the I^0/I^+ conversion reaction. Notably, the KA-PA-U electrolyte shows two distinct and reversible redox peaks, indicating well-defined four-electron iodine conversion reactions. This trend is more obvious and intuitive in corresponding GCD profiles (Fig. R20d). The GCD profiles of the KA-U electrolyte only exhibit one pair of plateaus attributed to the I^-/I^0 couple, where the PA-U electrolyte presents a long charging tail with a negligible discharge plateau at the I^0/I^+ region. Differently, the KA-PA-U electrolyte shows two pairs of reversible plateaus, confirming the successful realization of reversible four-electron iodine conversion reactions. Similar results can be obtained when acetamide and nicotinamide were used as Nuc species, highlighting the generality of this observation (Fig. R20b-c, e-f).

As a result, even under identical ionic strength, the electrochemical trends and mechanistic interpretations remain entirely consistent with those reported in the main text. These findings confirm that the differences in total ionic concentration or ionic strength do not alter the intrinsic reaction pathways and synergistic contributions of KA and PA. Moreover, they further reinforce the essential and complementary functions of

KA and PA in enabling reversible four-electron iodine conversions and underscore the robustness and broad applicability of the KA–PA–Nuc electrolyte design. Therefore, corresponding data and discussion have been added into the revised manuscript: "*Moreover, the KA-PA-Nuc electrolyte standard was further demonstrated using other Nuc, including acetamide (AE), nicotinamide (NA), and pyridine (PD). In all cases, even at identical ionic strength, reversible four-electron iodine conversion reactions were achieved under the synergistic contributions of PA and KA, indicating the broad applicability of the KA-PA-Nuc standard (Fig. 2c-d, Fig. 2f-g, and Supplementary Fig. 14-17)*".

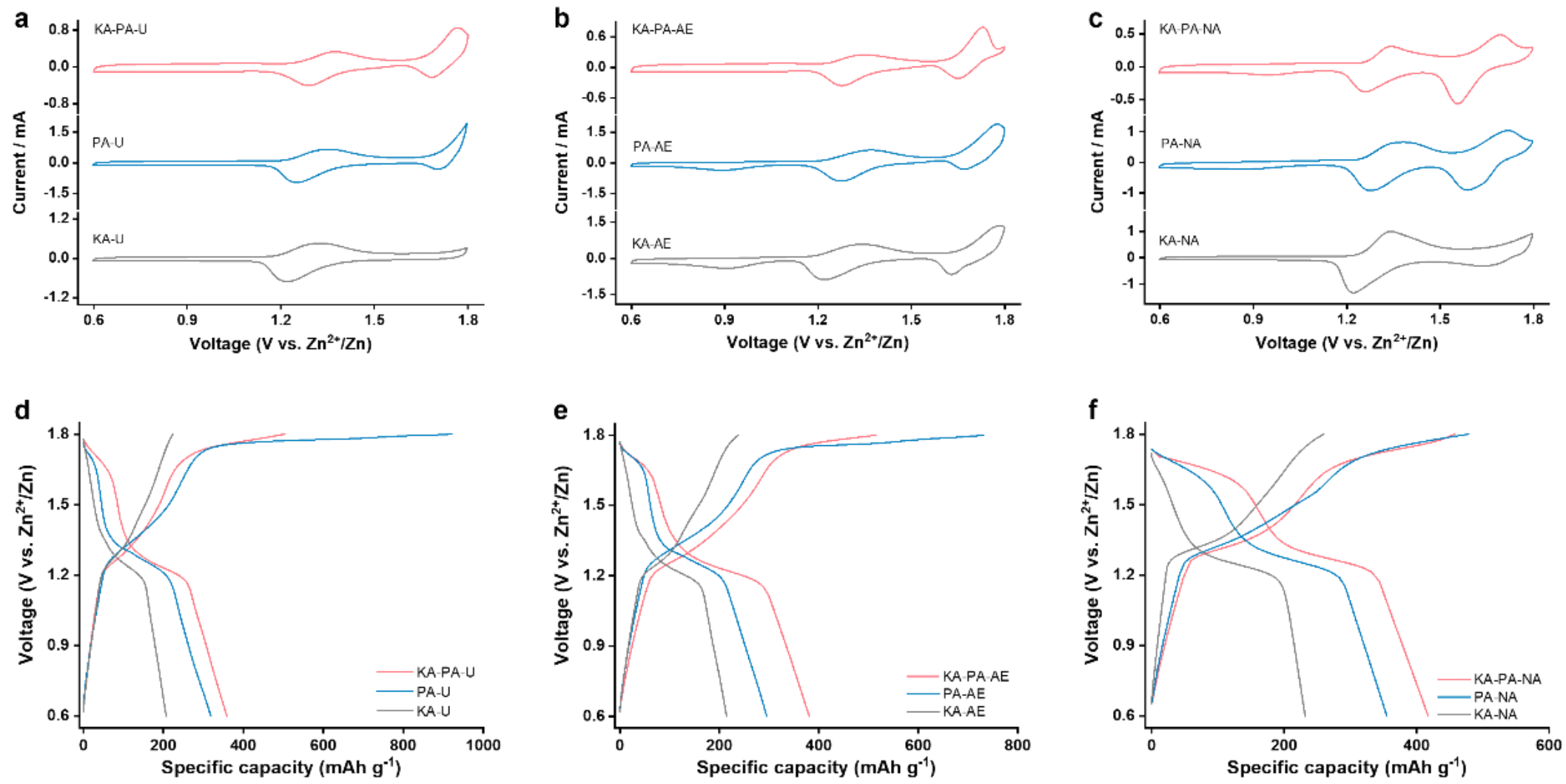

Fig. R20 Electrochemical performance comparison of electrolytes with identical ionic strength. a, CV profiles of iodine conversion reaction using urea as Nuc. b, CV profiles using acetamide as Nuc. c, CV profiles using nicotinamide as Nuc. d-f, Corresponding GCD profiles for a-c at 1 A g^{-1}, respectively.

Comment 4. Furthermore, in Figure 2, it is difficult to confirm the current scale of each cyclic voltammogram due to the absence of clearly defined y-axis values or scale indicators. To improve the clarity and comparability of the data, it is recommended that the authors include scale bars or explicitly label the current axis for each panel.

Response: Following the reviewer's suggestion, we have now included clearly defined y-axis labels and scale indicators for all CV profiles to improve clarity and facilitate direct comparison among different electrolytes. In the original version, y-axis values were intentionally omitted because the numerical labels at both ends of adjacent panels overlapped, which compromised figure readability. To resolve this issue, we increased the spacing between adjacent panels, enabling the inclusion of well-defined y-axis values without visual interference. With these improvements, the revised figures more clearly demonstrate that the KA–PA–Nuc electrolytes exhibit significantly more reversible redox currents compared with control systems, further confirming the successful realization of reversible four-electron iodine conversion reactions and underscoring the broad applicability of the KA–PA–Nuc electrolyte design.

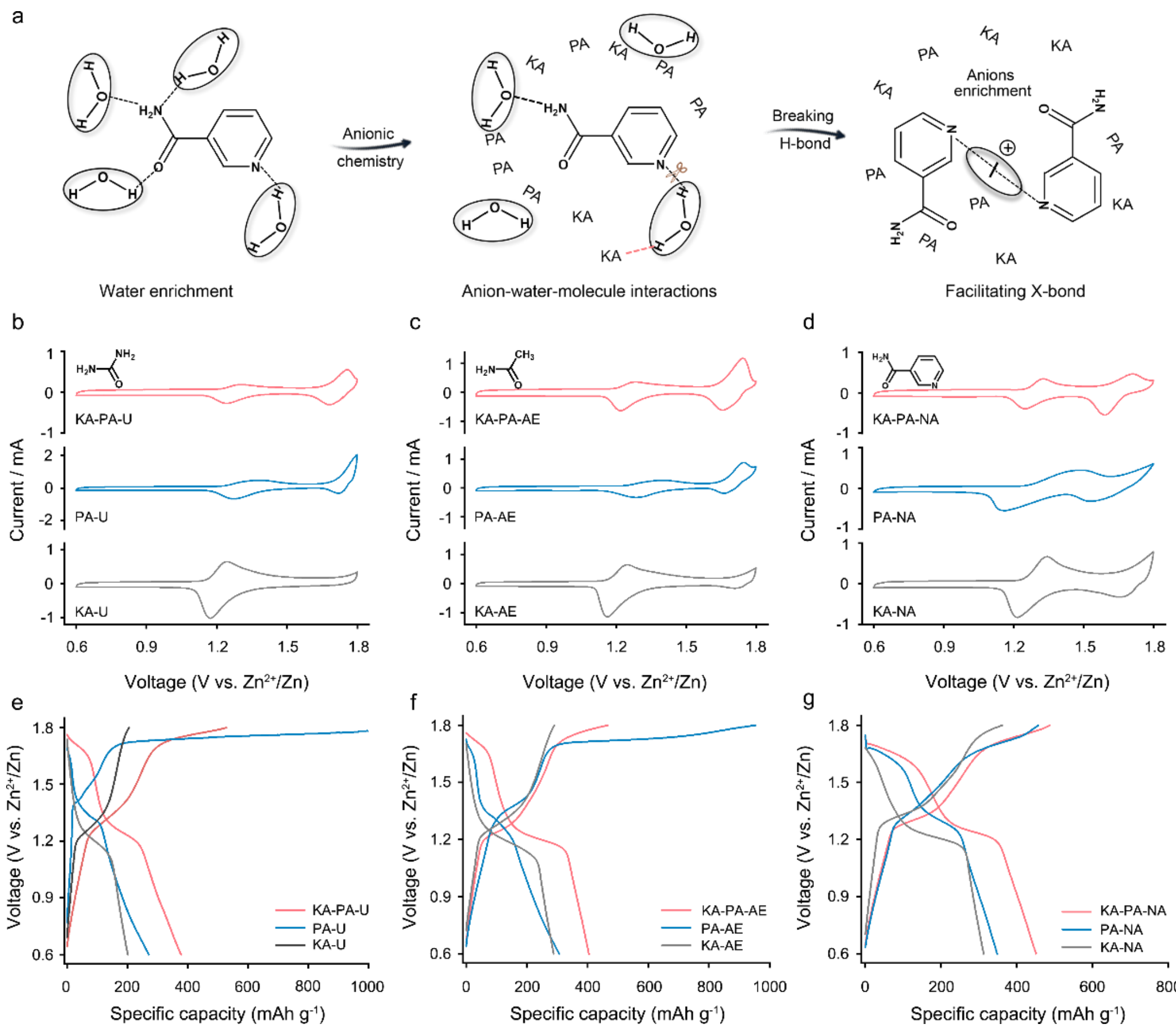

Fig. 2 Versatility of proposed KA-PA-Nuc electrolyte standard. a, Molecular interplay governed by anionic chemistry and halogen bonding. b, Cyclic voltammetry (CV) profiles of iodine conversion reaction using urea as Nuc at 0.6 mV s^{-1}. c, CV

profiles using acetamide as Nuc at 0.6 mV s^{-1}. d, CV profiles using nicotinamide as Nuc at 0.6 mV s^{-1}. e-g, Corresponding galvanostatic charge-discharge (GCD) profiles for b-d at 1 A g^{-1}, respectively.

Comment 5. As noted by the authors, the PA-Nuc electrolyte in Figures 2e and 2f exhibits a second charge plateau with a notably large capacity. Theoretically, if all I^0 were irreversibly oxidized to IO_3^-, the maximum charge capacity should be up to five times that of the I^-/I^0 redox couple (first charge plateau). However, the experimentally observed capacity appears to exceed this limit. Therefore, further discussion is needed to clarify the nature of the reactions occurring during the second charge plateau.

Response: We thank the reviewer for raising this important point. To gain a more reliable mechanistic understanding of the second charge plateau, we conducted additional analyses discussion accordingly.

To disclose the nature of the reactions occurring during the second charge plateau, ultraviolet-visible spectroscopy (UV/Vis) was utilized to monitor the electrolyte composition at different states of charge. A home-made beaker cell was assembled to facilitate electrolyte sampling at defined charging states for *ex situ* characterization (Fig. R21a). As shown in Fig. R21a, the charging profile in the beaker cell closely matches that of the coin-cell configuration, confirming comparable electrochemical behavior. At the early charging stages (points A and B), the UV–Vis spectra of the extracted electrolytes show no significant difference compared to the bare electrolyte, suggesting a relatively stable I^-/I^0 conversion reaction. However, as the charging states approaching the second charging plateau, distinct absorption features correspond to I^- and IO_3^- species emerge and intensify (consistent with a previous report, *Angew. Chem. Int. Ed. 2024, 63, e202410994*), indicating continuous and irreversible oxidation processes involve iodine species.

To further assess whether iodine chemistry alone accounts for the excessive

capacity, electrochemical performance of the activated carbon (AC) cathode in PA-Nuc electrolytes was investigated (Fig. R22). The AC electrode was prepared and tested following identical protocols to the iodine cathode but contained no iodine species. Interestingly, the AC cathode also exhibits a prolonged charging plateau at high potential region in PA-Nuc electrolytes, demonstrating that an additional irreversible process occurs. Given the chemical stability of the AC cathode, this observation strongly suggests electrolyte oxidation under high potentials. This interpretation aligns with electrochemical stability window measurements (Fig. R1), which reveal that PA–Nuc electrolytes possess the lowest oxygen evolution potential among the tested systems. This reduced anodic stability promotes parasitic water oxidation at the potential range corresponding to the second plateau.

Taken together, these findings indicate that the large capacity associated with the second plateau arises from a combination of irreversible oxidation of iodine species and parasitic oxygen evolution reaction originating from electrolyte decomposition. The corresponding interpretation in the manuscript has been revised to reflect this refined mechanistic understanding: "*In the PA-U electrolyte, a second charge plateau accompanied by a long tail emerges, attributed to the further oxidation of iodine and water, but the corresponding discharge plateau is negligible, indicating the irreversibility of the I^0/I^+ conversion reaction (Supplementary Fig. 11-12)*".

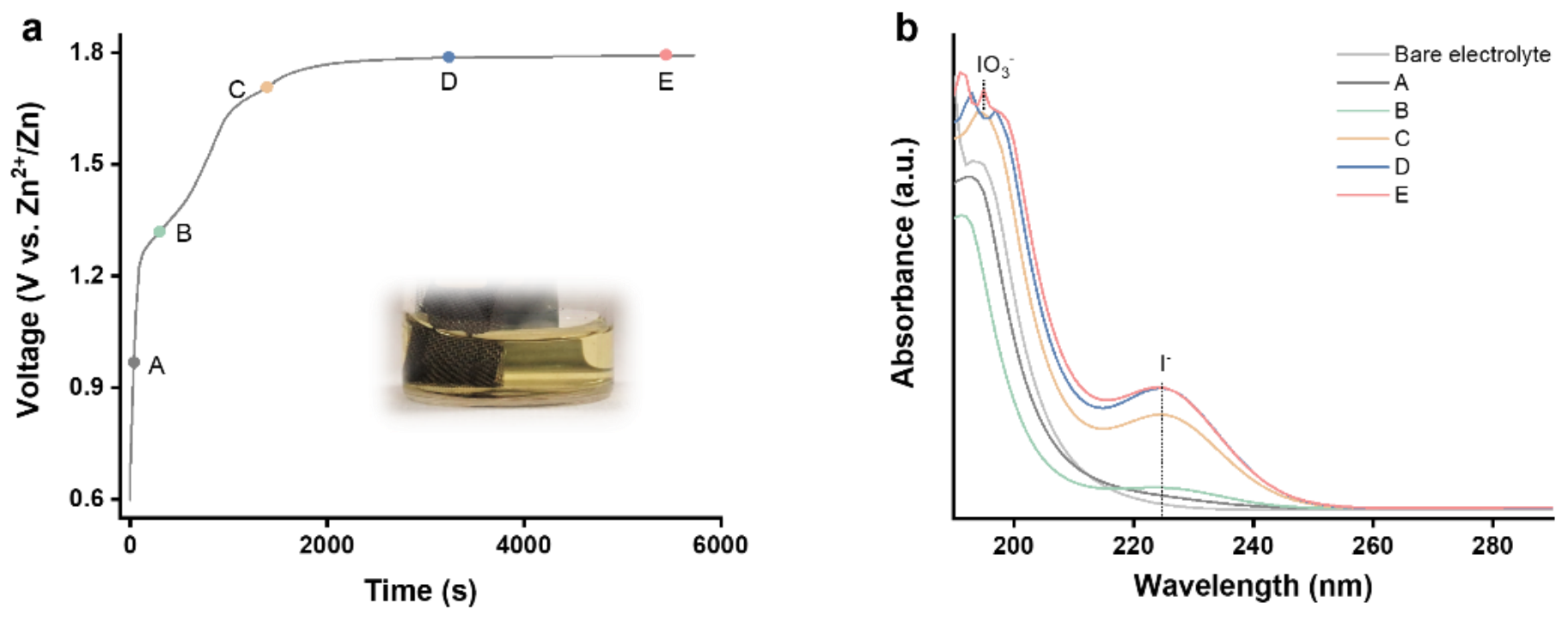

Fig. R21 Analysis of electrochemical products in the PA-Nuc electrolyte. a,

Charging profile of the iodine cathode in the PA-Nuc electrolyte. The inset shows the corresponding picture of the charged beaker cell. b, UV–Vis spectra of the PA-Nuc electrolyte at different states of charge.

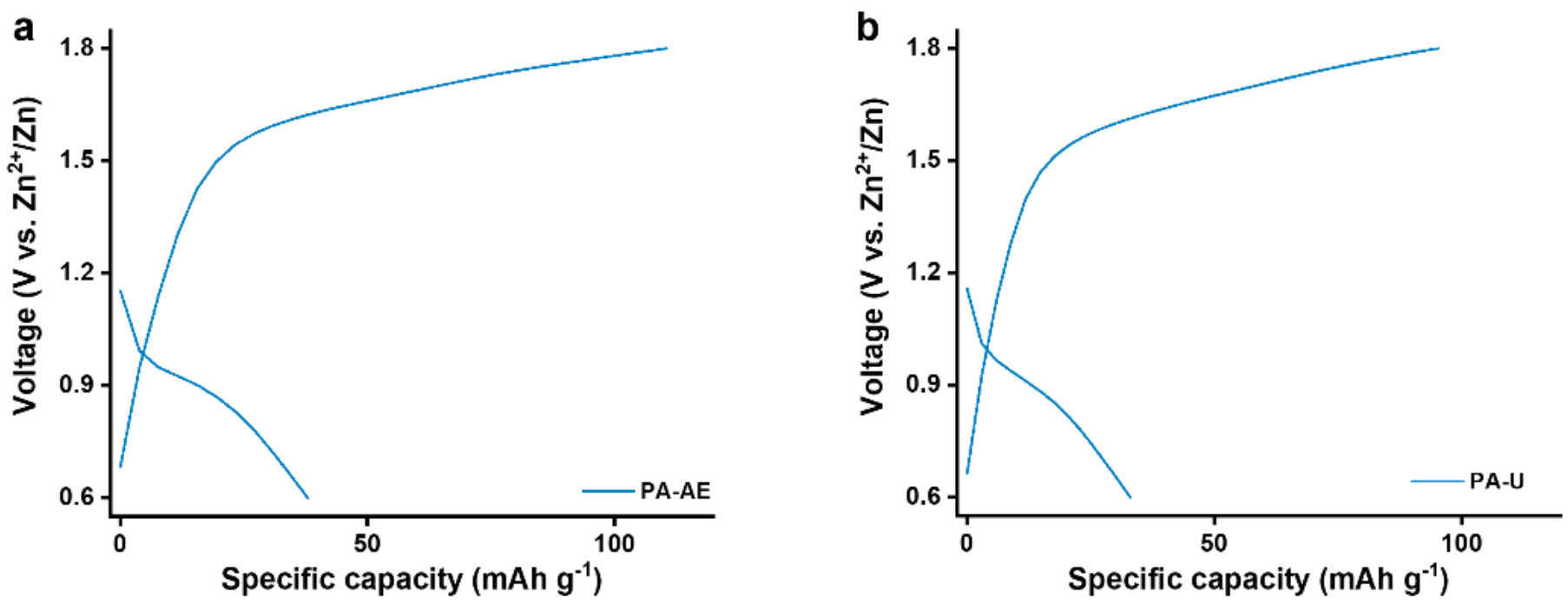

Fig. R22 Electrochemical performance of the AC cathode in PA-Nuc electrolytes. a, GCD profile of the AC cathode in the PA-AE electrolyte. b, GCD profile of the AC cathode in the PA-U electrolyte.

Comment 6. In Figure 3, the authors propose that polar anions preferentially adsorb onto the electrode surface, creating an anion-enriched and water-deficient electrolyte/electrode interface that enhances the reversibility of the four-electron reaction. However, it remains unclear whether the oxidation products (I^+ or Nuc-I-Nuc) are immobilized at the electrode surface or can diffuse or migrate into the bulk electrolyte. Clarification on the spatial localization of these species is necessary. Moreover, the assertion that the water distribution within 5 Å of the electrode governs reaction reversibility requires more robust justification.

Response: To clarify the spatial localization of the oxidation products, *in-situ* UV–Vis spectroscopy was employed to monitor the evolution of polyiodide species in different electrolytes during cycling, which in turn provides evidence regarding the stability and mobility of the oxidation products. In the *in situ* UV–Vis spectra (Fig. R23), the characteristic absorption peak at ~350 nm corresponds to the formation of I_3^-, which

signifies the instability of the oxidation products and the migration of iodine species into the bulk electrolyte. For the electrolyte containing $CF_3SO_3^-$, the I_3^- absorption peak gradually intensifies with prolonged cycling, suggesting a partially irreversible iodine conversion process accompanied by moderate iodine diffusion (Fig. R23a). Consistently, the electrolyte near the electrode surface turns from transparent to light yellow after cycling, further confirming moderate migration of iodine species (inset of Fig. R23d).

For the electrolyte containing CF_3COO^-, the I_3^- absorption peak increases rapidly during cycling, indicating a highly irreversible iodine conversion reaction with extensive migration of iodine species into the bulk electrolyte (Fig. R23b). The corresponding electrolyte becomes uniformly yellow, further proving the severe diffusion of iodine species (Fig. R23e). In contrast, for the electrolyte containing CH_3COO^-, no discernible I_3^- absorption appears with prolonged cycling, and the electrolyte at the electrode surface remains transparent (Fig. R23c, f). This observation demonstrates that the oxidation products are effectively immobilized in the electrode and iodine conversion reaction is reversible.

Notably, the relative intensity of the UV–Vis signals follows the order CF_3COO^- > $CF_3SO_3^-$ > CH_3COO^-, reflecting distinct interfacial behaviors among the three electrolytes. Corresponding GCD profiles support this trend. In the electrolyte containing $CF_3SO_3^-$, only one pair of redox plateaus associated with I^-/I^0 couple appears in GCD profiles (Fig. R23d), indicating that the I^0/I^+ conversion reaction is not activated in this electrolyte environment. Compared with I^0/I^+ conversion reaction, I^-/I^0 conversion reaction is relatively stable, thereby the overall reaction is relatively reversible, and the absorption signal is moderate. In contrast, the electrolyte containing CF_3COO^- activates the I^0/I^+ conversion reaction but in a highly irreversible manner, leading to severe side reactions and extensive iodine species migration (Fig. R23e). Remarkably, the electrolyte containing CH_3COO^- forms an anion-enriched, water-deficient interface that effectively confines the oxidation products in the electrode,

thereby enabling a highly reversible four-electron iodine conversion reaction (Fig. R23f). These findings are fully consistent with the electrochemical performance trends, where the electrolyte containing CH_3COO^- shows the highest capacity and the best long-term stability, the electrolyte containing $CF_3SO_3^-$ exhibits moderate cycling stability but the lowest capacity, and the electrolyte containing CF_3COO^- displays the poorest cycling performance (Fig. 3c). Collectively, these results clearly demonstrate that the spatial distribution and reversibility of iodine redox species are strongly governed by the interfacial structure determined by the anion type.

Moreover, regarding the reviewer's comment about "the water distribution within 5 Å of the electrode governs reaction reversibility", further justification was conducted accordingly. This distance of ~5 Å corresponds well to the effective thickness of the inner Helmholtz layer (IHL) within the electric double layer (EDL), typically reported to be 3–5 Å, where solvent molecules and specifically adsorbed ions directly interact with the electrode surface (*Bard, A. J. & Faulkner, L. R. Electrochemical Methods: Fundamentals and Applications Wiley & Sons (2001); Chem. Soc. Rev., 2009,38, 2520-2531*). This range is consistent with recent studies (*Science, 2021, 371, 6524, 46-51; Nat. Commun. 2024, 15, 1, 6471; Adv. Mater. 2025, 37, 2501049; Angew. Chem. 2023, 135, e202302302*). Within this compact interfacial layer, the distribution of ions and solvent molecules defines the microenvironment for mass diffusion, charge transfer and redox reactions.

Our MD simulations, *in situ* ATR-IR spectra, and differential capacitance data jointly reveal that the strongly polar anions (CH_3COO^-) preferentially accumulate within this IHL, displacing interfacial water molecules and forming an anion-enriched, water-deficient zone. This microenvironment reduces interfacial water activity, effectively suppressing parasitic reactions between water and high-valent iodine species, while immobilizing reactive intermediates (proved by *in-situ* UV–Vis spectra and electrochemical data). In contrast, weakly polar anions (CF_3COO^- and $CF_3SO_3^-$) exhibit limited specific adsorption, leading to a water-rich interface that cannot prevent side

reactions and iodine species migration. Therefore, the water and anion distributions within the compact layer critically govern the reversibility of the iodine conversion reaction by modulating the interfacial microenvironment for mass diffusion, charge transfer, and redox reactions. This mechanistic interpretation aligns with classical EDL theory fully supported by our theoretical and experimental observations (including MD simulations, *in situ* ATR-IR spectra, *in-situ* UV–Vis spectra, and electrochemical data). Therefore, corresponding data and clarification have been incorporated into the revised manuscript: "*Among these processes, ions involved electrolyte-electrode interface critically determines the microenvironment for mass diffusion, charge transfer, and redox reactions*"; "*Importantly, in situ UV–Vis spectra, revealing the spatial distribution and reversibility of iodine redox species, are fully consistent with the electrochemical trends, highlighting the pivotal role of the anion-enriched, water-deficient interface created by the strongly polar anion in enabling reversible four-electron iodine conversion reactions (Supplementary Fig. 28)*".

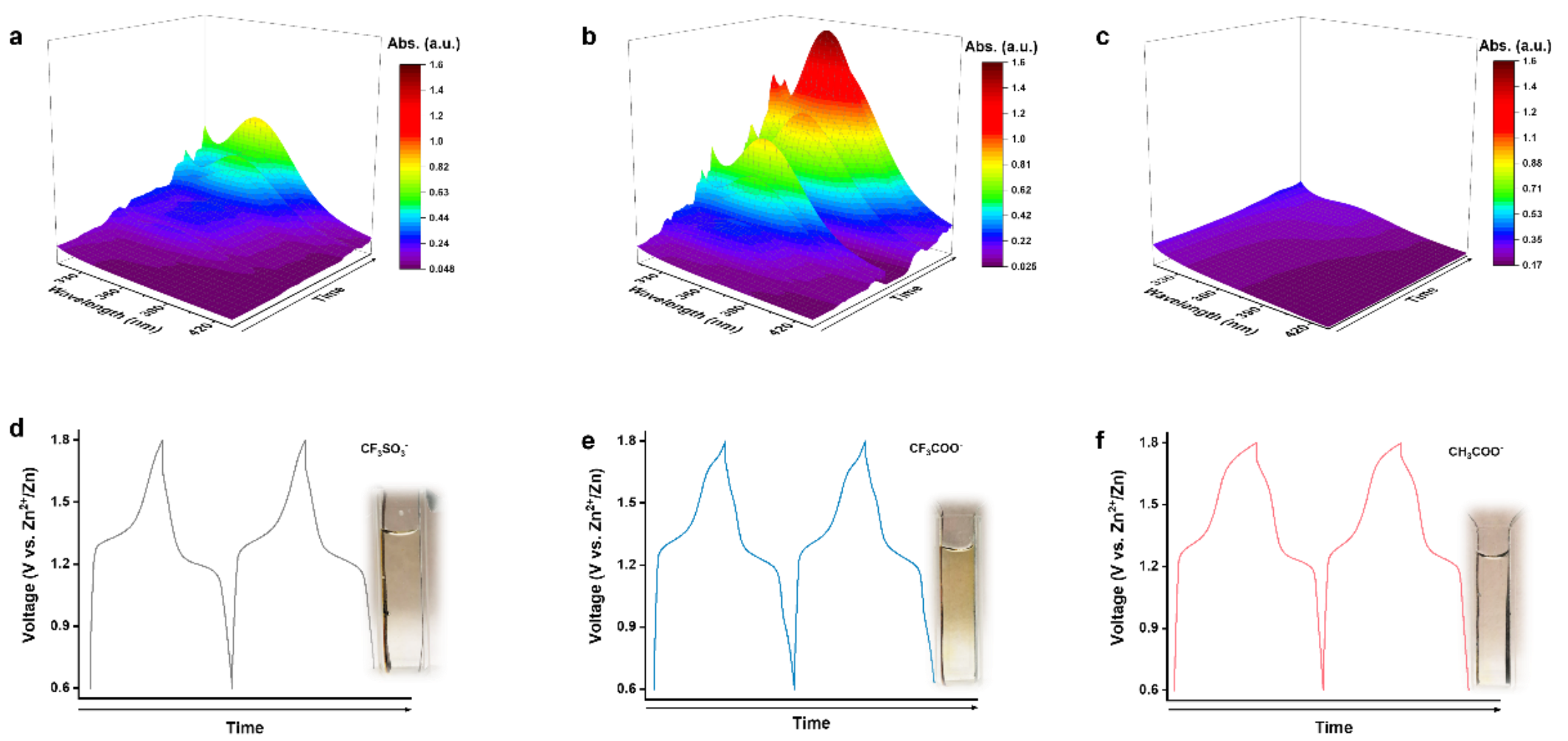

Fig. R23 Characterization of the spatial distribution of iodine species. a-c, *In-situ* UV–Vis spectra in electrolytes with $CF_3SO_3^-$, CF_3COO^-, CH_3COO^-, respectively. d-f, Corresponding GCD profiles in electrolytes with $CF_3SO_3^-$, CF_3COO^-, CH_3COO^-, respectively. Insets show the corresponding pictures of cuvette cells.

Comment 7. Regarding the ATR-IR results in Figures 3g-3i, it is unclear under which electrochemical condition (e.g., charging or discharging) the data were collected. As the polarity of the electrode changes during cycling, the interfacial ion distribution is expected to vary significantly. Therefore, clarification of the measurement conditions is essential for proper interpretation. If ATR-IR spectra were collected under both charging and discharging conditions, the interpretation of interfacial changes would be significantly clearer.

Response: Following the suggestion, ATR-IR spectra were collected under a full charging/discharging cycle, and corresponding measurement conditions have now been added to the revised manuscript. Specifically, *in situ* ATR-IR spectra were recorded by a Thermo Scientific Nicolet iS50 spectrometer equipped with a liquid nitrogen-cooled MCT-A detector. Gold-coated silicon plates were employed as working electrodes, with Zn foils and glass fiber used as reference electrodes and separators, respectively. To assess the critical role of polar anion in regulating interfacial environment, 1.5 M KA electrolyte was maintained as the baseline, while 0.5 M $Zn(CH_3COO)_2$, 0.5 M $Zn(CF_3COO)_2$, and 0.5 M $Zn(CF_3SO_3)_2$ were introduced separately. During the test, the assembled cells were connected to a Gamry Interface 1000 potentiostat to run a Cyclic Voltammetry (CV) program at a scan rate of 0.2 mV s^{-1} for a full charge and discharge cycle. All spectra were collected at a resolution of 4 cm^{-1}, with 50 scans per measurement.

According to the ATR-IR spectra, the evolution of the electrode/electrolyte interfacial environment during charging and discharging differs markedly across the three anions. In the ATR-IR spectra, the distinct peak located at 1640 cm^{-1} corresponds to the δ-HOH vibration of interfacial water, while other peaks are associated with corresponding anions. For electrolytes containing $CF_3SO_3^-$ or CF_3COO^-, the δ-HOH vibration remains dominant with only slight fluctuation during charge/discharge cycle (Fig. 3g-h). Corresponding anion-associated peaks are consistently weak throughout

the cycle, revealing a consistently water-enriched, anion-deficient interfacial environment. In sharp contrast, in the electrolyte with CH_3COO^-, the δ-HOH vibration is markedly suppressed throughout the full cycle, while the characteristic peaks of SO_4^{2-} and CH_3COO^- remain prominent and further intensify at high potentials (Fig. 3i). These results reveal a continuously anion-enriched, water-deficient interfacial environment. This observation is fully consistent with the interfacial ion distribution mechanism elaborated in our response to Comment 8, further highlighting the decisive role of strongly polar CH_3COO^- in creating an anion-enriched, water-deficient interface. Therefore, corresponding data and discussion have been updated: "*As an experimental validation, in situ attenuated total reflection infrared (ATR-IR) spectroscopy was conducted to examine mass distribution at the electrolyte-electrode interface. The distinct peak located at 1640 cm^{-1} corresponds to the δ-HOH vibration of interfacial water, while other peaks are associated with corresponding anions (Supplementary Fig. 22).[24-27] For electrolytes containing $CF_3SO_3^-$ or CF_3COO^-, the δ-HOH vibration remains dominant with only slight fluctuation during charge/discharge cycle (Fig. 3g-h). Corresponding anion-associated peaks are consistently weak throughout the cycle, revealing a persistently water-enriched, anion-deficient interfacial microenvironment. Conversely, in the electrolyte with CH_3COO^-, the δ-HOH vibration is markedly suppressed throughout the full cycle, while the characteristic peaks of SO_4^{2-} and CH_3COO^- remain prominent and further intensify at high potentials, suggesting a continuously anion-enriched, water-deficient interfacial environment (Fig.3i)*".

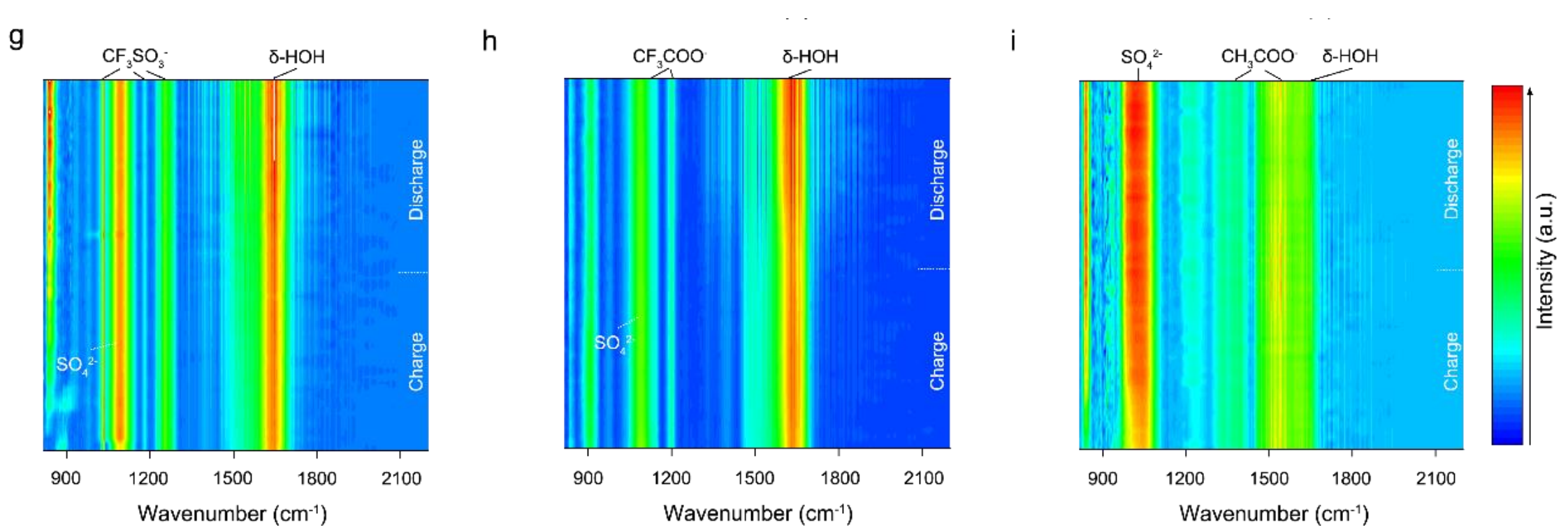

Fig. 3 Critical role of polar anions. g-i, *In situ* ATR-IR spectra in the electrolytes with $CF_3SO_3^-$, CF_3COO^-, CH_3COO^-, respectively.

Comment 8. In Figure 3i, it is unclear why the nonpolar sulfate anion appears in such high abundance. Further explanation is needed to clarify this observation.

Response: To prove this observation, we repeated the relevant measurements multiple times and consistently reached the same conclusion. To disclose the mechanism underlying the high abundance of the sulfate anion, evolution of the interfacial environment in different electrolytes were comprehensively studied. First, the specific adsorption behavior of various anions with the same concentration was assessed by differential capacitance. The results show that strongly polar CH_3COO^- exhibits the highest specific adsorption, which is significantly higher than other anions, highlighting the critical role of CH_3COO^- in establishing an anion-enriched, water-deficient electrolyte-electrode interface (Fig. R24). Interestingly, SO_4^{2-} displays the second specific adsorption, exceeding that of CF_3COO^- and $CF_3SO_3^-$. Although SO_4^{2-} is nonpolar, its low negative electrostatic potential enables a low adsorption energy towards the positively polarized electrode surface, as supported by recent studies (*Nat. Sustain., 2025, 1-11; Energy Environ. Sci., 2023, 16, 2910–2923; ACS Energy Lett., 2021, 6, 2704−2712*). This behavior accounts for the high abundance of SO_4^{2-} in the Figure 3i.

To further elucidate the interplay among anions, SO_4^{2-}-based KA electrolyte was maintained as the baseline, while additional anions with varying polarities were introduced. The evolution of interfacial environment in KA electrolytes before and after the addition of another anion was explored. As discussed above, although SO_4^{2-} shows inferior specific adsorption capacity than strongly polar CH_3COO^-, it is also beneficial for promoting anion-enriched interface. Correspondingly, the baseline KA electrolyte

possesses a decent differential capacitance and a SO_4^{2-} abundant interface (Fig. R25). Upon the introduction of CF_3COO^- or $CF_3SO_3^-$, differential capacitances dramatically decrease, reflecting the disruption of the original SO_4^{2-}-enriched interface and the reintroduction of water at the interface (Fig. R25b-c, e-f). In sharp contrast, the addition of CH_3COO^- significantly increases the differential capacitance, confirming that CH_3COO^- displaces interfacial water while retaining SO_4^{2-} at the interface, thereby producing an anion-enriched, water-deficient interface (Fig. R25a, d). Moreover, even though SO_4^{2-} has inferior specific adsorption than CH_3COO^-, its concentration is much higher. These results align well with MD simulations and *in situ* ATR-IR spectra, which further explain the high interfacial abundance of SO_4^{2-} in the KA electrolytes containing CH_3COO^-.

Collectively, these findings explain the observed high interfacial abundance of SO_4^{2-} and highlights the decisive role of strongly polar CH_3COO^- in creating an anion-enriched, water-deficient interface in the KA electrolyte. These findings further explain why electrolytes containing only SO_4^{2-} are insufficient to activate and stabilize four-electron iodine conversion reactions. Therefore, corresponding data and explanation have been added to the revised manuscript: "*Furthermore, differential capacitance was employed to quantify specific adsorption behavior and elucidate the interplay among anions. Strongly polar CH_3COO^- exhibits the highest specific adsorption, which is significantly higher than other anions (Supplementary Fig. 25). This strong adsorption capacity effectively stabilizes the interfacial microenvironment, whereas weak CF_3COO^- and $CF_3SO_3^-$ severely disrupt the interfacial microenvironment (Supplementary Fig. 26)*".

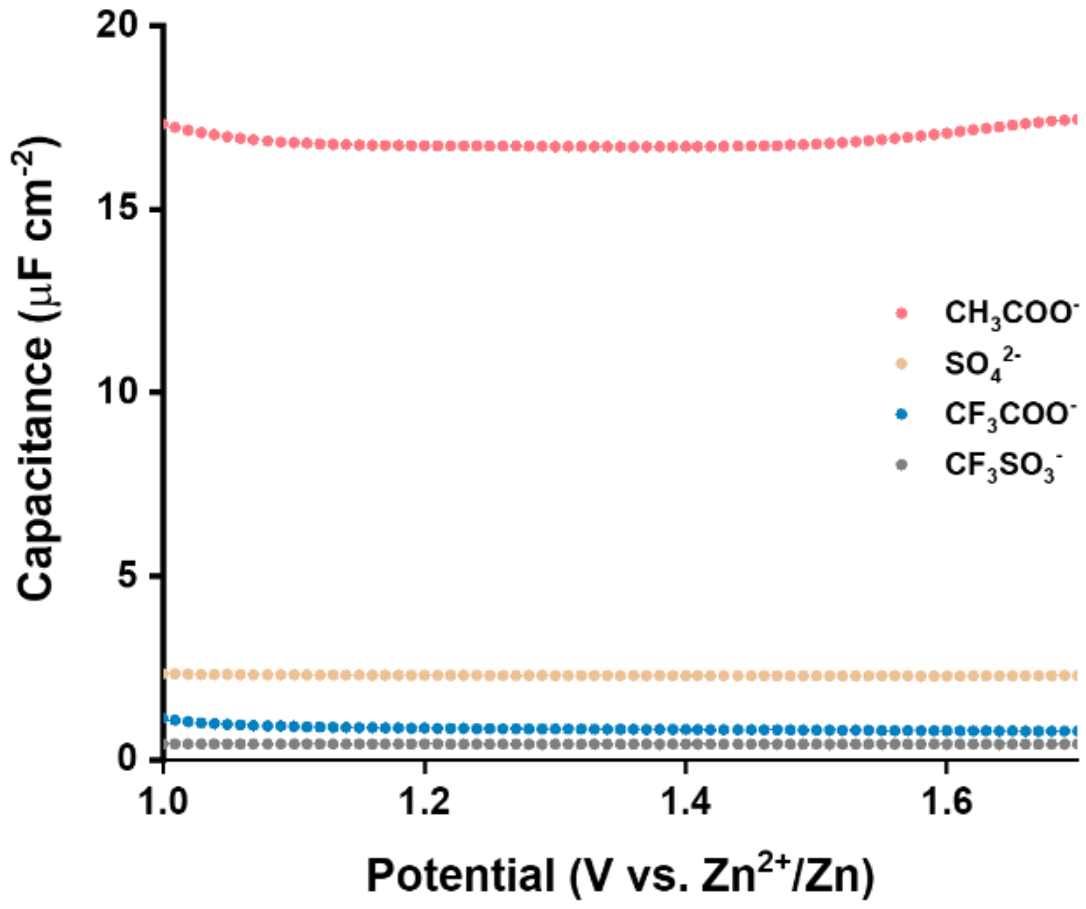

Fig. R24 Differential capacitance curves in different electrolytes.

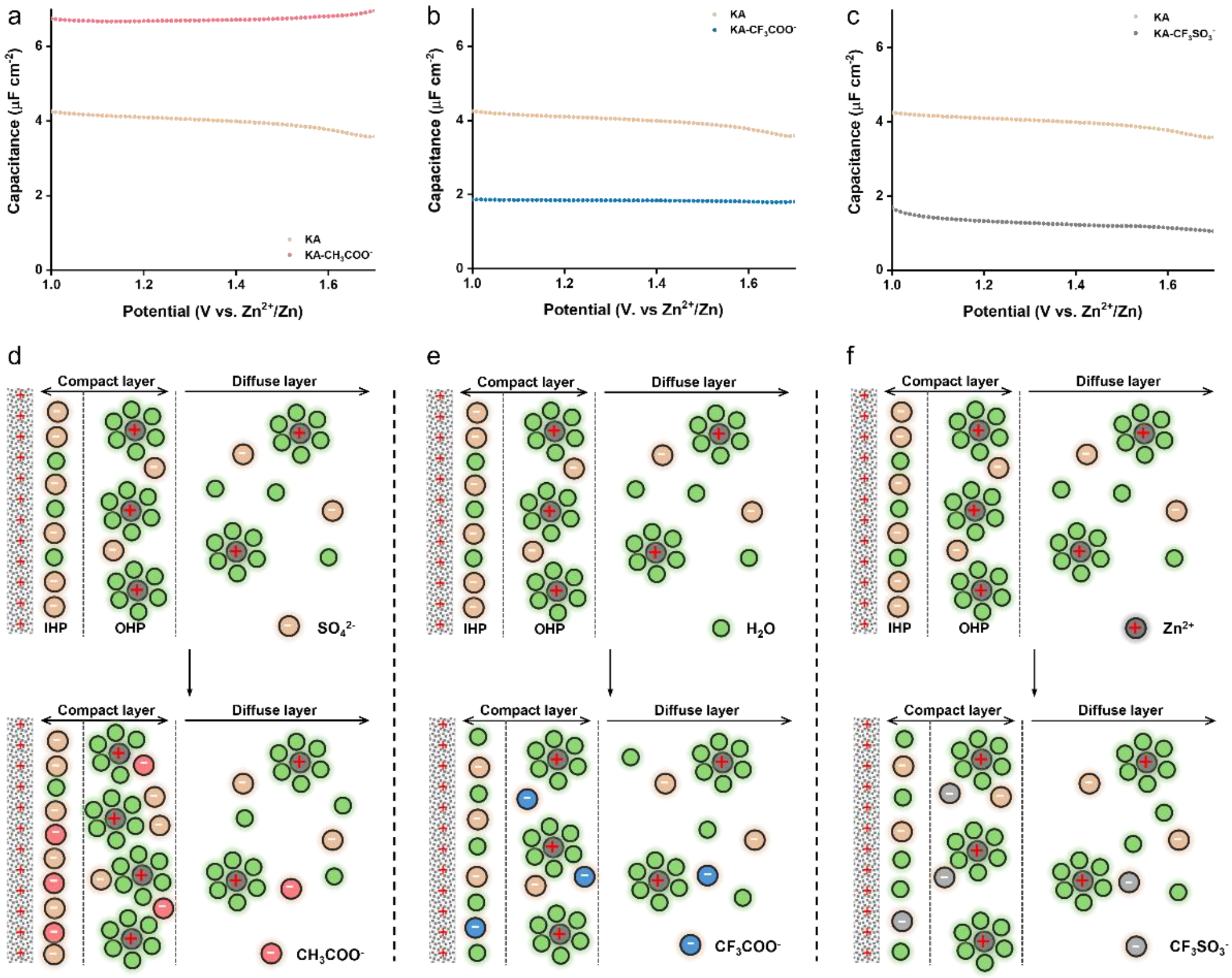

Fig. R25 Evolution of the interfacial environment in KA (SO_4^{2-}) electrolytes with different anions. a, Differential capacitance curves of KA electrolytes before and after the addition of CH_3COO^-. b, Differential capacitance curves of KA electrolytes before and after the addition of CF_3COO^-. c, Differential capacitance curves of KA electrolytes before and after the addition of $CF_3SO_3^-$. d, Schematic illustration of the interfacial evolution in KA electrolytes upon introduction of CH_3COO^-. e, Schematic illustration

of the interfacial evolution in KA electrolytes upon introduction of CF_3COO^-. f, Schematic illustration of the interfacial evolution in KA electrolytes upon introduction of $CF_3SO_3^-$.

Comment 9. The rationale for excluding halide anions from the study of kosmotropic effects is not clearly stated. Given that the main objective of this work is to propose electrolyte design rules, including halide anions to complete the observed trends would enhance the comprehensiveness and impact of the study.

Response: We thank the reviewer for this important comment. Indeed, halide anions are commonly included in the anion-specific series in other fields. However, we excluded halide anions from this study for practical reason. From a practical perspective, the central objective of this study is to establish a versatile, halogen-free, and low-concentration electrolyte standard for reversible four-electron iodine conversion reactions. This design aims to eliminate reliance on conventional high-concentration, halogen-containing electrolytes, which are corrosive, costly, and limited in applicability. In our preliminary tests, halogen-containing electrolytes caused severe corrosion of battery cases and rapid cell failure. Therefore, excluding halide anions ensures the practical viability, safety, and sustainability of the proposed electrolyte framework (Fig. R26). Therefore, the rationale for this exclusion has been clarified in the revised manuscript: "*excluding halide anions to maintain practicability and halogen-free electrolyte standard*".

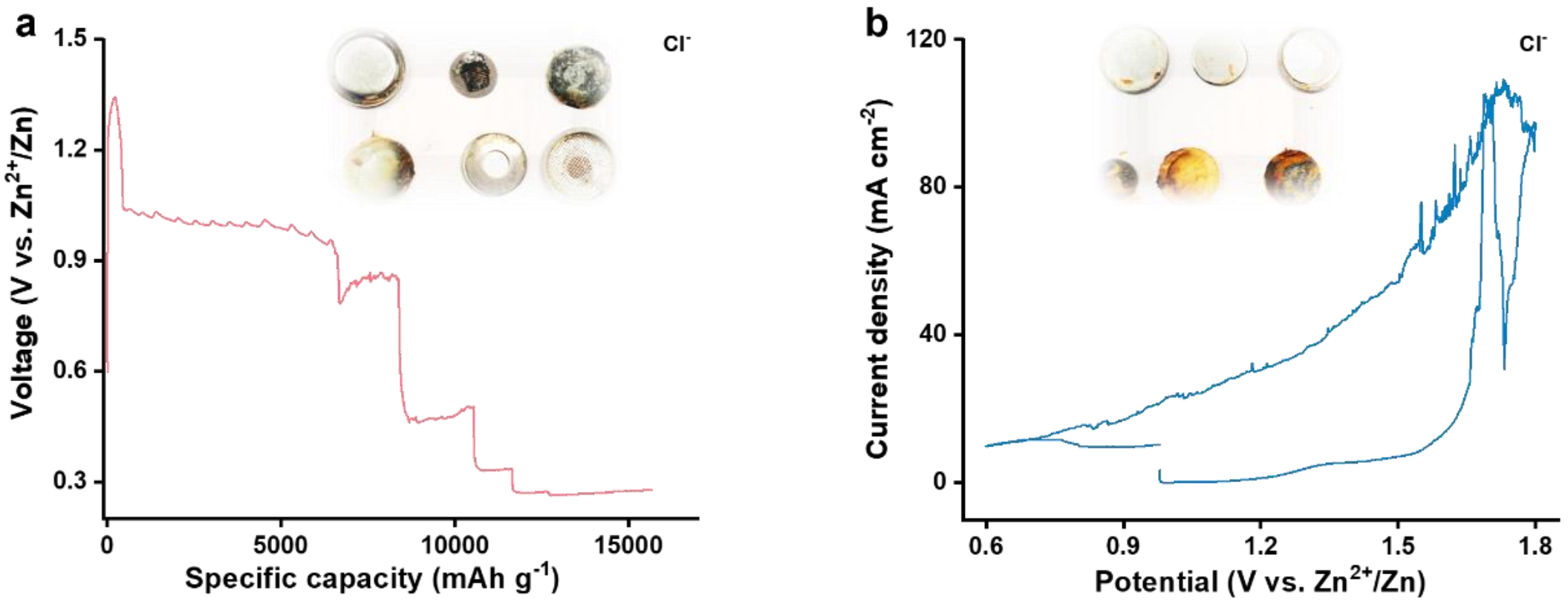

Fig. R26 Electrochemical performance of halogen-containing electrolytes. a, GCD profile of the iodine cathode in the 1.5m $ZnCl_2$-PA-Nuc electrolyte. b, CV profile of the iodine cathode in the 1.5m $ZnCl_2$-PA-Nuc electrolyte.

Comment 10. In the NMR analysis presented in Figure 4, the authors claim that the chemical shift of the 2H peak indicates strong coordination between sulfate ions and water molecules. However, stronger anion-water coordination is typically expected to increase electron density around the hydrogen nuclei, leading to a shielding effect and an upfield shift. Similarly, weaker hydrogen bonding between water and the nitrogen atoms in Nuc molecules would increase local electron density around nitrogen, also resulting in an upfield shift. In contrast, the present study reports pronounced downfield shifts in both 2H and 15N spectra for the sulfate anion system. A more convincing interpretation is needed to reconcile these observations with the proposed coordination behavior.

Response: We thank the reviewer for this perceptive comment. The observed NMR trends can be more rationalized based on fundamental hydrogen bonding principles. The hydrogen bond (X–H···Y) is a type of noncovalent interaction in which a hydrogen atom (H) covalently bonded to an electronegative atom (X) interacts with another electronegative atom (Y, such as O, N, or F). Upon hydrogen bond formation, the electronegative atom Y withdraws electron density from the hydrogen nucleus (X–H),

reducing the shielding of the hydrogen nucleus and resulting in a downfield shift in NMR, while electron density around electronegative atom Y increases, enhancing shielding and leading to an upfield shift. This interpretation aligns with the IUPAC definition of hydrogen bond: "The hydrogen bond leads to characteristic NMR signatures that typically include pronounced proton deshielding for H in X–H" (*Pure Appl. Chem., Vol. 83, No. 8, pp. 1637–1641, 2011*).

In our study, the anion-water coordination involves electronegative anions withdrawing electron density from the hydrogen atoms of coordinated water molecules (hydrogen atoms become more positively polarized). This polarization deshields the hydrogen nuclei, producing the observed downfield ^2H shift (Fig. 4d), consistent with prior reports (*Adv. Mater. 2022, 34, 2110140; Nat. Sustain. 2025, 8, 1349–1359*). Thus, the progressive downfield trend (SO_4^{2-} > CH_3COO^- > NO_3^- > $CF_3SO_3^-$ > ClO_4^-) directly reflects increasing anion–water coordination strength and enhanced polarization of hydration water, correlate well with the anion-specific effect.

The downfield shift in the ^{15}N spectrum of Nuc molecules further supports this interpretation (Fig. 4g-i). Strong anion–water coordination sequesters and polarizes the hydration water around Nuc molecules, thereby weakening or reducing hydrogen bonds between water and the nitrogen atoms in Nuc. The reduced electron donation from hydrogen to nitrogen decreases local electron density around the nitrogen atoms, resulting in deshielding and a corresponding downfield ^{15}N shift. Collectively, these results confirm that the observed NMR shifts are consistent with the proposed anion-specific behavior and clearly disclose the anion-water-Nuc interactions, which highlight the critical role of kosmotropic anions (particularly SO_4^{2-}) in disrupting water-Nuc interactions and stabilizing four-electron iodine conversion reactions.

Comment 11. To better understand the interfacial electrochemical behavior, DRT analysis was conducted. While DRT is a useful tool for probing reaction mechanisms, it typically requires support from complementary in situ techniques to enable reliable peak assignments. In this study, however, the assignments of key peaks—particularly

t_2, t_3, and t_4—remain unclear. Moreover, a reduction in impedance alone does not necessarily indicate suppression of the I^+ hydrolysis reaction, as the I^0/I^+ redox process likely consists of sequential charge-transfer and subsequent chemical hydrolysis steps.

Response: We sincerely thank the reviewer for their insightful and professional comment regarding the DRT analysis. EIS is indeed a powerful, non-destructive diagnostic tool that evaluates battery impedance across a wide frequency range, providing valuable information about various electrochemical processes. However, accurately interpreting EIS data can be challenging due to the overlapping frequency responses arising from different electrochemical processes. In this context, the DRT technology can deconvolute these overlapping and intertwined electrochemical processes into distinct relaxation processes on different time scales, thereby improving the accuracy of interpretation. Nevertheless, the accurate identification of individual electrochemical processes in a full cell using DRT is still difficult. Full cell comprises an anode and a cathode detached by a separator containing the electrolyte, where electrochemical processes occur and interact simultaneously at the cathode, anode, and multiple interfaces. Such intricate compositions and interactions elevate the challenge of accurate identification of individual electrochemical process, which in turn hinders a precise mechanistic understanding.

To resolve this challenge and enable more reliable peak assignments, we integrated an additional reference electrode into the full cell configuration to establish three-electrode setup. In this setup, a thin zinc wire was placed between the cathode and anode (Fig. 5a), allowing the decoupling of individual electrode responses, so that the properties of each electrode and their contribution to the overall performance of the battery can be investigated individually. As shown in Nyquist plot (Fig. 5b), the impedance of the two-electrode cell closely matches the sum of two individual electrode impedances, confirming the reliability and accuracy of the reference electrode in decoupling individual electrode contribution of full cell during the *in situ* EIS measurement. Similarly, the corresponding DRT spectra demonstrates that the DRT plot

of the two-electrode cell is approximately equivalent to the combination of DRT plots of the individual electrodes, further validating the reliability and accuracy of this methodology (Fig. 5c). Notably, both EIS and DRT spectra indicate that the overall impedance of the full cell is predominantly governed by the anode.

To accurately assign each characteristic relaxation process, systematic control experiments and comprehensive analyses were conducted. The Nyquist plots of both anode and cathode show a small arc and a pronounced semicircle in the high-frequency region, followed by an oblique line at low frequencies (Fig. R27a, c). Typically, the semicircle corresponds to the parallel combination of the charge-transfer resistance and a capacitive element, whereas the oblique line reflects the ion diffusion process within the electrode. Corresponding DRT spectra reveal six distinct relaxation peaks (τ_1-τ_6) across different frequency domains (Fig. R27b, d). Among them, τ_1-τ_4 appear in the high-frequency region and are associated primarily with charge transfer related processes, while τ_5-τ_6 are found in the low-frequency region and correspond to ion transport or diffusion phenomena. Specifically, the τ_1 peak is located at a very high frequency with a very small characteristic time scale of approximately 10^{-5} s. This process is attributed to the intrinsic resistance likely from current collectors, electrode interfaces and electrode particles, which is consistent with its fast relaxation time. Moreover, this peak remains stable within different electrolytes across various potentials, further supporting its assignment (Fig. 5). The τ_2 peak, observed at around 10^{-4} s, is largely electrolyte-dependent and electrode-independent (Fig. 5). It is prominent in electrolytes containing PA (KA (SO_4^{2-})-PA (CH_3COO^-)-Nuc and $CF_3SO_3^-$ -PA (CH_3COO^-)-Nuc) but absent in the electrolyte without PA (KA (SO_4^{2-})-$CF_3SO_3^-$-Nuc). Given its timescale and electrolyte sensitivity, τ_2 is assigned to the SEI resistance at electrode/electrolyte interface. This assignment aligns with theoretical calculations and *in situ* FTIR analyses, both of which reveal the preferential adsorption of PA and the formation of an anion-rich interface that facilitates the four-electron iodine conversion process.

The mid-frequency region manifests two distinct peaks (τ_3 and τ_4), which fall within the typical category of charge transfer behavior. The τ_3 peak appears in both the Zn anode and iodine cathode across all potentials, whereas the τ_4 peak emerges only in the potential range associated with the I^-/I^0 and I^0/I^+ redox reactions of the iodine cathode but persists in the Zn anode over all potentials (Fig. 5 and Fig. R28). Moreover, τ_3 peak is electrode-dependent, its magnitude varies significantly among the Zn anode, iodine cathode, and activated carbon (AC) cathode with diverse surface properties. Meanwhile, τ_4 is completely absent in the AC cathode without iodine redox species across all potentials (Fig. R27e). During charging, the electrolyte ions migrate toward the electrode surfaces under the applied electric field, overcoming interfacial impedance barriers. Zn^{2+} ions undergo reduction on the anode surface to form metallic zinc, while iodine species undergo oxidation at the cathode once the potential reaches the corresponding redox threshold. Based on the observed behavior and correlation with electrochemical processes, τ_3 is assigned to the interfacial resistance associated with ion migration and surface adsorption, whereas τ_4 is attributed to the charge-transfer process involving redox species (Zn^{2+} and iodine). Importantly, compared with KA (SO_4^{2-})-PA (CH_3COO^-)-Nuc electrolyte, the τ_4 peak of the iodine cathode in $CF_3SO_3^-$-PA (CH_3COO^-)-Nuc and KA (SO_4^{2-})-$CF_3SO_3^-$-Nuc electrolytes gradually evolves into two distinct peaks within the potential range corresponding to I^+ formation, indicating the coexistence of side reactions and incomplete I^+ conversion (Fig. 5d-i and Fig. R29). This observation is fully consistent with the electrochemical results, confirming that DRT analysis effectively captures the interfacial dynamics and reaction pathways in different electrolyte environments.

At lower frequencies (<10 Hz), τ_5 and τ_6 appear, corresponding to ion transport and diffusion processes. These peaks reflect limitations in mass transport as ions penetrate deeper into the electrode, consistent with their large relaxation times and slower kinetics. As a result, all peaks have been reliably assigned to their corresponding electrochemical processes leveraging this *in situ* decoupling methodology. In addition, we also fully agree with the reviewer that a reduction in impedance alone does not

necessarily indicate suppression of the I^+ hydrolysis reaction. Instead, detailed analysis of the impedance evolution provides a more accurate interpretation. Notably, in the KA (SO_4^{2-})–PA (CH_3COO^-)–Nuc electrolyte, the τ_4 peak of the iodine cathode remains stable, and no additional charge-transfer-related peak emerges within the I^+ formation potential range, indicating the absence of side reactions and successful I^+ conversion. To further evaluate the suppression of I^+ hydrolysis, *in situ* ultraviolet-visible (UV-vis) spectroscopy was employed to monitor evolution of polyiodide species in different electrolytes during cycling. The adsorption bands of polyiodide species in $CF_3SO_3^-$-PA (CH_3COO^-)-Nuc and KA (SO_4^{2-})-$CF_3SO_3^-$-Nuc electrolytes gradually intensify with extended cycling time, indicating incomplete I^+ conversion and the dissolution of polyiodide byproducts. In contrast, these adsorption bands are neglectable in the KA (SO_4^{2-})–PA (CH_3COO^-)–Nuc electrolyte, further confirming the successful I^+ conversion and effective suppression of I^+ hydrolysis (Fig. R23). Moreover, *in situ* Raman spectroscopy provides additional evidence of the reversible I^+ conversion in the KA (SO_4^{2-})–PA (CH_3COO^-)–Nuc electrolyte (Supplementary Fig. 49). These complementary *in situ* spectroscopic results collectively validate the proposed mechanism and support the impedance-derived conclusions. Accordingly, all relevant data and discussion regarding EIS and DRT have been updated to ensure better accuracy and reliability (*For details, please refer to the updated discussion of Fig. 5 in the main text and Supplementary Fig. 39-41 in the supporting information*).

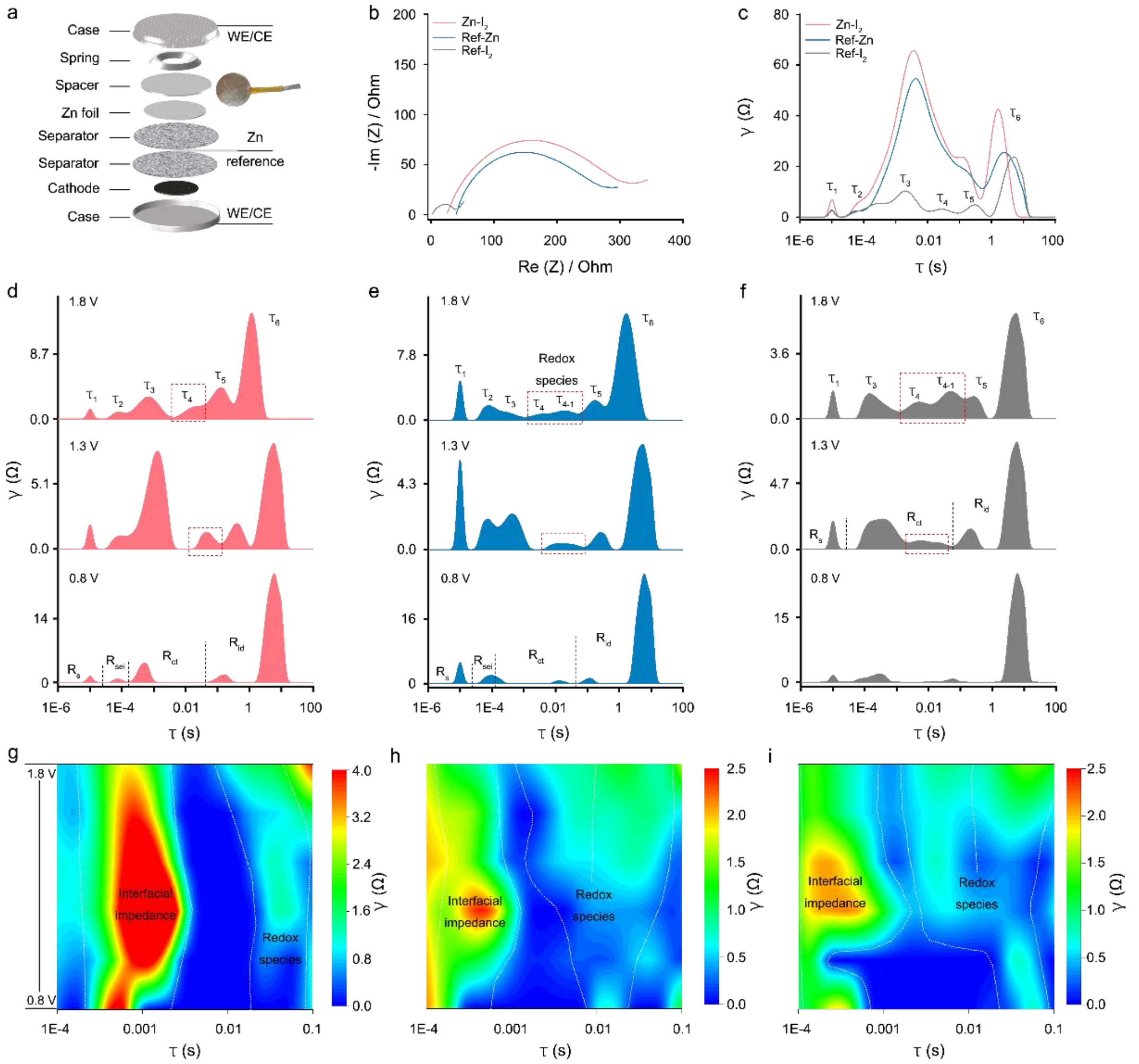

Fig. 5 Characterizations of interfacial electrochemical behaviors. a, Schematic illustration of the specialized three-electrode configuration. b, Nyquist plots of two-electrode full cell and individual electrodes. c, Corresponding DRT profiles of two-electrode full cell and individual electrodes. d, DRT profiles of the iodine cathode in the KA (SO_4^{2-})-PA (CH_3COO^-)-Nuc electrolyte at different states. e, DRT profiles of the iodine cathode in the $CF_3SO_3^-$-PA (CH_3COO^-)-Nuc electrolyte without KA (SO_4^{2-}) at different states. f, DRT profiles of the iodine cathode in the KA (SO_4^{2-})-$CF_3SO_3^-$-Nuc electrolyte without PA (CH_3COO^-) at different states. g, Contour plots of the iodine cathode in the KA (SO_4^{2-})-PA (CH_3COO^-)-Nuc electrolyte at different states. h, Contour plots of the iodine cathode in the $CF_3SO_3^-$-PA (CH_3COO^-)-Nuc electrolyte at different states. i, Contour plots of the iodine cathode in the KA (SO_4^{2-})-$CF_3SO_3^-$-Nuc electrolyte at different states.

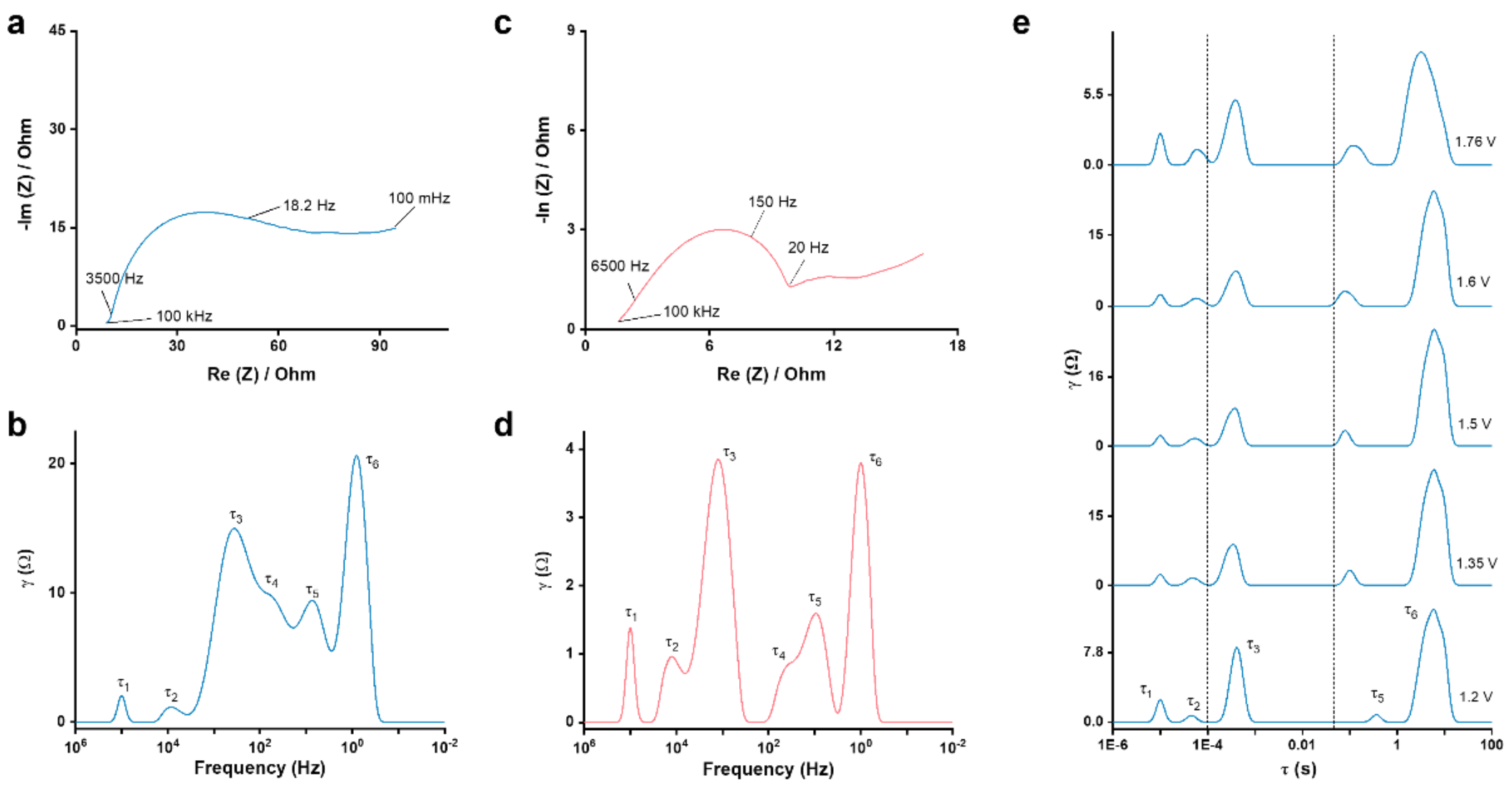

Fig. R27 In-depth analysis of interfacial electrochemical behavior. a, Nyquist plot of the individual Zn anode at 1.78 V. b, Corresponding DRT plot of a. c, Nyquist plot of the individual iodine cathode at 1.78 V. d, Corresponding DRT plot of c. e, DRT plots of individual activated carbon (AC) cathode at different voltages.

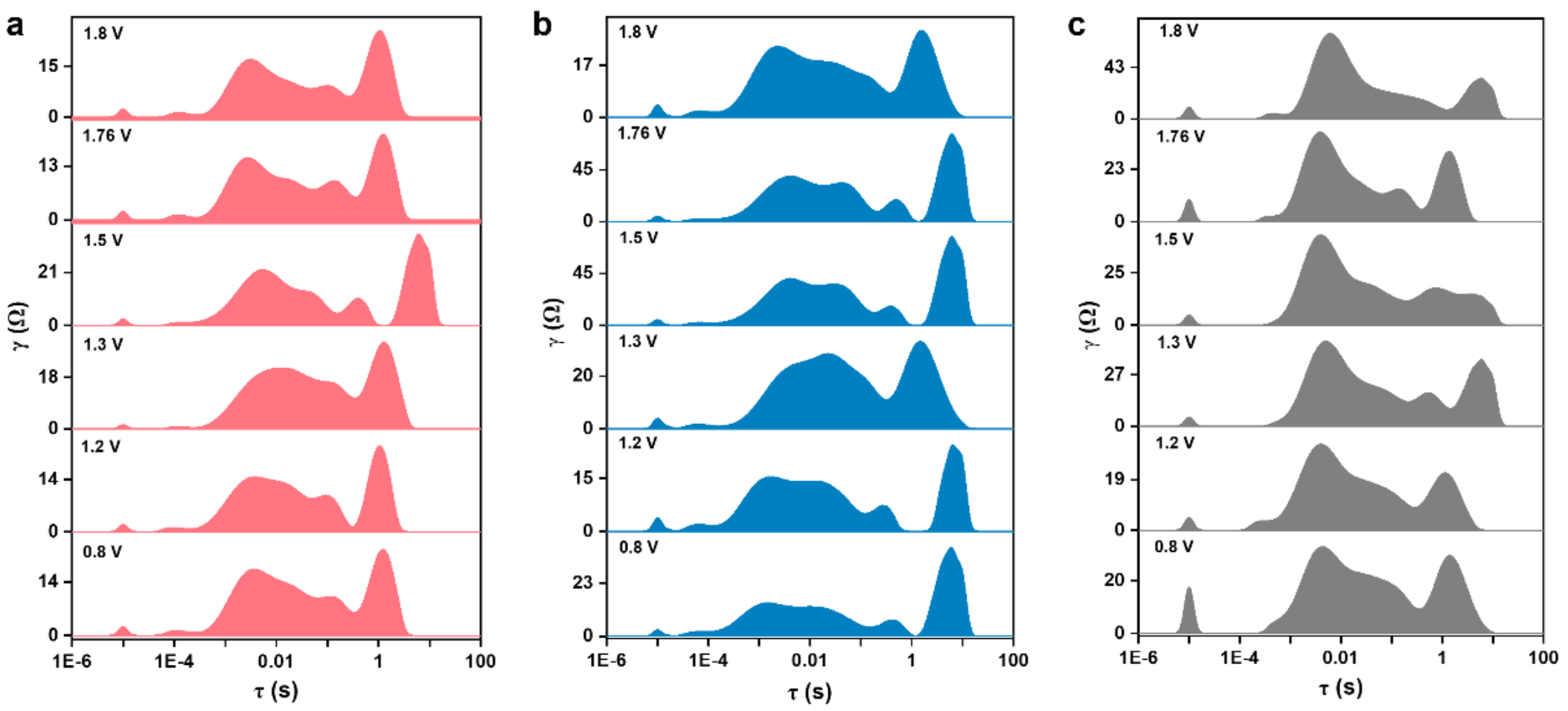

Fig. R28 DRT profiles of the Zn anode in different electrolytes at various charging states. a, KA (SO_4^{2-})-PA (CH_3COO^-)-Nuc electrolyte. b, $CF_3SO_3^-$-PA (CH_3COO^-)-Nuc electrolyte. c, KA (SO_4^{2-})-$CF_3SO_3^-$-Nuc electrolyte.

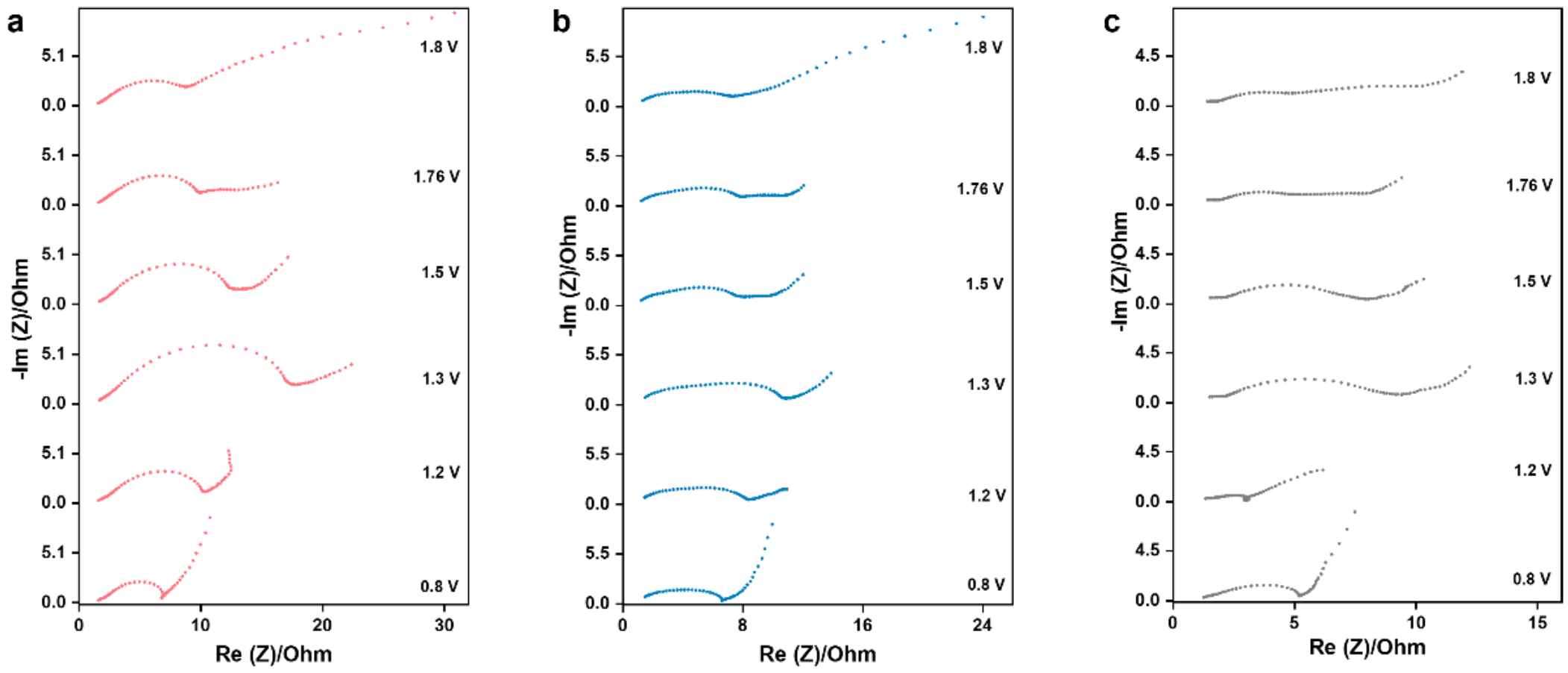

Fig. R29 *In situ* EIS profiles of the iodine cathode in different electrolytes at various charging states. a, KA (SO_4^{2-})-PA (CH_3COO^-)-Nuc electrolyte. b, $CF_3SO_3^-$-PA (CH_3COO^-)-Nuc electrolyte. c, KA (SO_4^{2-})-$CF_3SO_3^-$-Nuc electrolyte.

Comment 12. In Figure 5e, the assignment of the Raman peak to the Nuc-I-Nuc complex is not clearly justified. Given the presence of other anions in the system, alternative complexation pathways should also be taken into account.

Response: To assess the possibility of alternative anion-involved coordination pathways, we performed a series of electrochemical, spectroscopic, and theoretical analyses (see detailed discussion in the response to Comment 2). These results collectively demonstrate that the stabilization of I^+ arises from coordination with nucleophilic species via a linear Nuc–I^+–Nuc complex halogen bond, rather than through direct coordination with anions. Accordingly, this Raman peak is assigned to the Nuc–I^+–Nuc complex.

Furthermore, additional Raman spectroscopy provides direct spectroscopic evidence supporting this assignment. In the KA–PA electrolyte without Nuc species, no characteristic peak corresponding to I^+ complex was detected during charge/discharge cycling (Fig. R30). By contrast, a pronounced and reversible Raman peak emerged at

~210 cm^{-1} in the KA-PA-Nuc electrolyte (Supplementary Fig. 49). This feature is characteristic of I^{+}–ligand coordination and is consistent with previous reports (*J. Am. Chem. Soc. 2025, 147, 16350−16361; Nat. Commun. 2023, 14.1, 1856; Adv. Mater. 2024, 2405473; J. Am. Chem. Soc. 2024, 146, 31, 21377–21388*). Collectively, these findings demonstrate that I^{+} stabilization in our system arises from coordination with nucleophilic species, forming Nuc–I^{+}–Nuc complex. Therefore, the Raman peak observed at ~210 cm^{-1} is reasonably assigned to the Nuc–I^{+}–Nuc complex rather than to alternative anion-involved coordination pathways. The clarification has been added to the revised manuscript: "*Furthermore, in situ Raman spectroscopy was performed to directly track the evolution of iodine species. An obvious peak representing Nuc–I^{+}–Nuc complex appears upon charging to 1.5 V, while this peak gradually diminishes during subsequent discharging process, confirming the reversible conversion between I^{0} and I^{+} species in the KA (SO_4^{2-})-PA (CH_3COO^{-})-Nuc electrolyte (Supplementary Fig. 49-50)*".

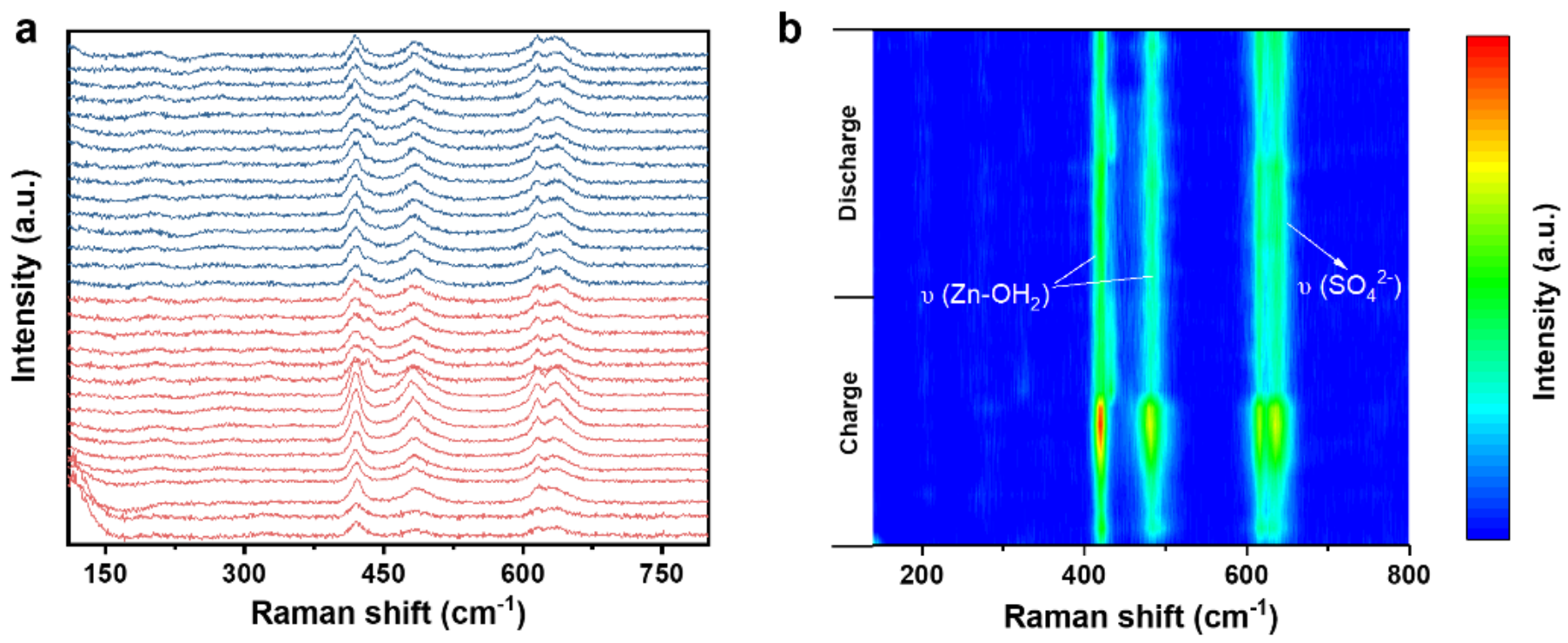

Fig. R30 *In situ* Raman spectroscopy in the electrolyte without Nuc. a, *In situ* Raman spectroscopy in the KA-PA electrolyte without Nuc. b, Corresponding contour plots of a.

Comment 13. The specific capacity values (mAh/g) reported in Figure 6 appear to be a key performance metric. If these values are based on the mass of iodine loaded on the cathode, it would be helpful to specify the iodine loading (mg/cm^2). This would allow

estimation of the areal capacity (mAh/cm^2) and facilitate evaluation of the practical relevance of the single-cell tests.

Response: We sincerely thank the reviewer for this constructive comment. We fully agree that both areal loading and areal capacity are important parameters for evaluating the practical practicability of batteries. Following the reviewer's suggestion, we have now included the corresponding areal iodine loadings and areal capacities in the revised manuscript. In Fig. 6, the iodine loading in the cathode is 2.2 mg cm^{-2}, corresponding to an initial areal capacity of 0.99 mAh cm^{-2}, which already surpasses most of previously reported four-electron aqueous metal-iodine battery.

To further assess the practical practicability of the KA-PA-Nuc standard, electrochemical performance under high iodine loadings was performed. The Zn-I_2 batteries achieved reversible four-electron iodine conversion reactions at different iodine loadings, delivering a practical areal capacity up to 4.1 mAh cm^{-2} (Fig. R31a). The rate capability was evaluated at a high iodine loading of 12.2 mg cm^{-2}, where the batteries display reversible capacities of 373, 339, 298, 247 mAh g^{-1} deliver at current densities of 0.5, 1, 2, and 4 A g^{-1}, respectively (Fig. R31b-c). The long-term cycling performance further underscores the robustness of the KA–PA–Nuc standard. At a high iodine loading of 7.6 mg cm^{-2}, the Zn-I_2 batteries delivered a capacity of 336 mAh g^{-1} with a capacity retention of 89% after 1,000 cycles at a current density of 1 A g^{-1} (Fig. R31d). Even under an ultrahigh iodine loading of 12.2 mg cm^{-2}, the Zn-I_2 batteries achieved a high capacity of 350 mAh g^{-1} with a capacity retention of 72% over 800 cycles at a current density of 0.5 A g^{-1} (Fig. R31e). Compared with previously reported four-electron aqueous metal-iodine batteries, these results represent a significant advancement in both iodine loading and areal capacity, further validating the broad applicability of the KA-PA-Nuc standard in advancing practical four-electron aqueous metal-iodine batteries. Therefore, corresponding information has been added into the revised manuscript: "*Zn-I_2 batteries with a moderate iodine loading of 2.2 mg cm^{-2} in the KA-PA-Nuc electrolyte*"; "*To further evaluate the performance benefits under*

practical conditions, electrochemical performance under high iodine loadings was performed. Remarkably, Zn-I_2 batteries with the KA-PA-Nuc electrolyte exhibited reversible and robust four-electron iodine conversion reactions even at an ultrahigh iodine loading of 12.2 mg cm^{-2}, delivering a practical areal capacity up to 4.1 mAh cm^{-2} (Supplementary Fig. 54)".

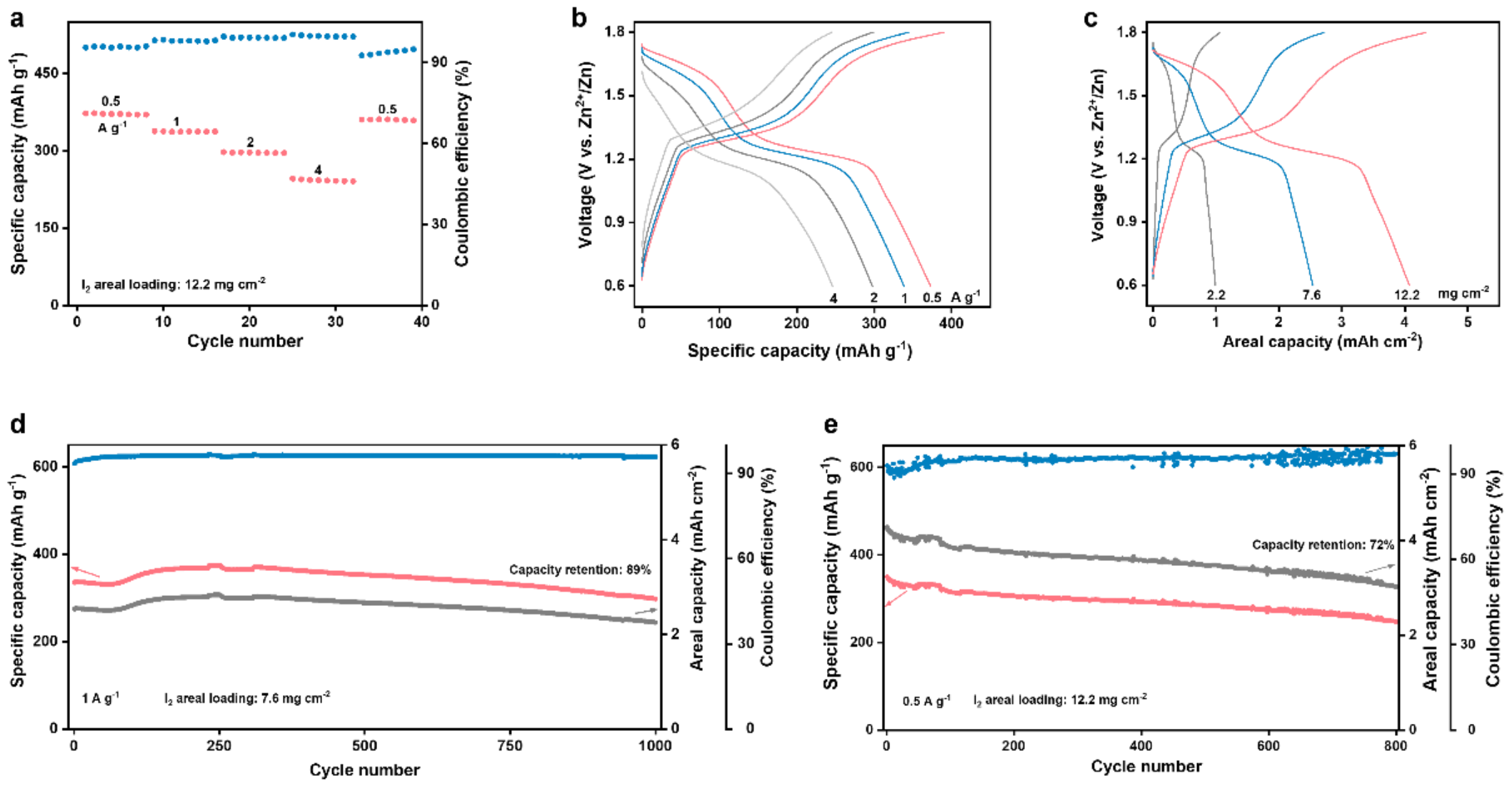

Fig. R31 Electrochemical performance of Zn-I_2 batteries in KA-PA-Nuc electrolytes at high iodine loadings. a, Rate performance at a high areal iodine loading of 12.2 mg cm^{-2}. b, Corresponding GCD profiles of a. c, GCD profiles showing areal capacities at different iodine loadings. d, Cycling performance at an areal iodine loading of 7.6 mg cm^{-2}. e, Cycling performance at an areal iodine loading of 12.2 mg cm^{-2}.

Referee #3

This manuscript presents an interesting and impactful study on the development of cost-effective, halogen-free aqueous electrolytes that enable reversible four-electron iodine redox reactions, overcoming a long-standing challenge in the field of aqueous metal-iodine batteries. The authors demonstrate a novel electrolyte design strategy guided by anionic chemistry, breaking away from traditional reliance on concentrated, halogen-rich systems. Such a universal anionic chemistry design principle successfully decouples hydrogen bonding from halogen bonding, allowing stable and reversible four-electron iodine redox reactions in a low-concentration and halogen-free aqueous environment. The universal electrolyte principle proposed here is broadly applicable and can inspire similar innovations across other aqueous redox systems. Therefore, the manuscript may be considered in Nature, but the following points must be addressed.

Response: We sincerely thank the reviewer for reviewing our manuscript. We greatly appreciate the positive assessment of our work and the constructive comments provided to further improve this manuscript. All points raised have been carefully addressed, and detailed point-by-point responses are provided below. We hope that the revised version meets your expectations.

Comment 1. The authors investigated the interfacial microenvironment of the electrolytes with varying anion polarities using in situ ATR-IR. However, the manuscript lacks a comparative analysis of high-concentration halogen-containing electrolytes. Specifically, what is the mass distribution at the electrode–electrolyte interface under such conditions, and how does this influence the electrochemical behavior?

Response: According to the comment, we have added detailed characterization and discussion of high-concentration, halogen-containing electrolytes in the revised manuscript. Specifically, the widely used high-concentration $ZnCl_2$ electrolyte (30m

$ZnCl_2$) was selected as a representative halogen-containing electrolyte for comparative analysis.

MD simulations were first conducted to explore the bulk properties of the high-concentration $ZnCl_2$ electrolyte, which revealed a water-deficient, anion-enriched solvation structure (Fig. R32). This is a typical feature for high-concentration electrolytes, where intensive ion coordination suppresses water activity. However, subsequent interfacial MD simulations revealed that the water molecule density remains high at the electrode-electrolyte surface, indicating a water-enriched interfacial microenvironment even under a highly concentrated condition (Fig. R33a-b). This finding was experimentally validated by *in situ* ATR-IR spectroscopy, where a strong peak at ~1640 cm^{-1} corresponds to the δ-HOH vibration of water molecules, confirming the substantial distribution of water molecules at the interface (Fig. R33c). Therefore, despite the "water-deficient" bulk solvation, the interfacial microenvironment remains water-enriched, which is unfavorable for I^+ stabilization and leads to inferior electrochemical stability. In contrast, our low-concentration, halogen-free KA–PA–Nuc electrolytes establish a water-deficient, anion-enriched interfacial microenvironment via the strongly polar anion. Concurrently, the kosmotropic anion effectively disrupts water–Nuc hydrogen bonding and enhances Nuc availability through specific anion–water–Nuc interactions, thereby enabling reversible four-electron iodine conversion with exceptional long-term stability. Therefore, corresponding data and discussion have been added in the revised manuscript: "*In addition, a widely used high-concentration $ZnCl_2$ electrolyte was examined as a representative halogen-containing system for further comparison. Despite its water-deficient bulk solvation structure, the interfacial microenvironment remains water-enriched even at ultrahigh concentration, underscoring the effectiveness of CH_3COO^- in regulating interfacial microenvironment (Supplementary Fig. 23-24)*".

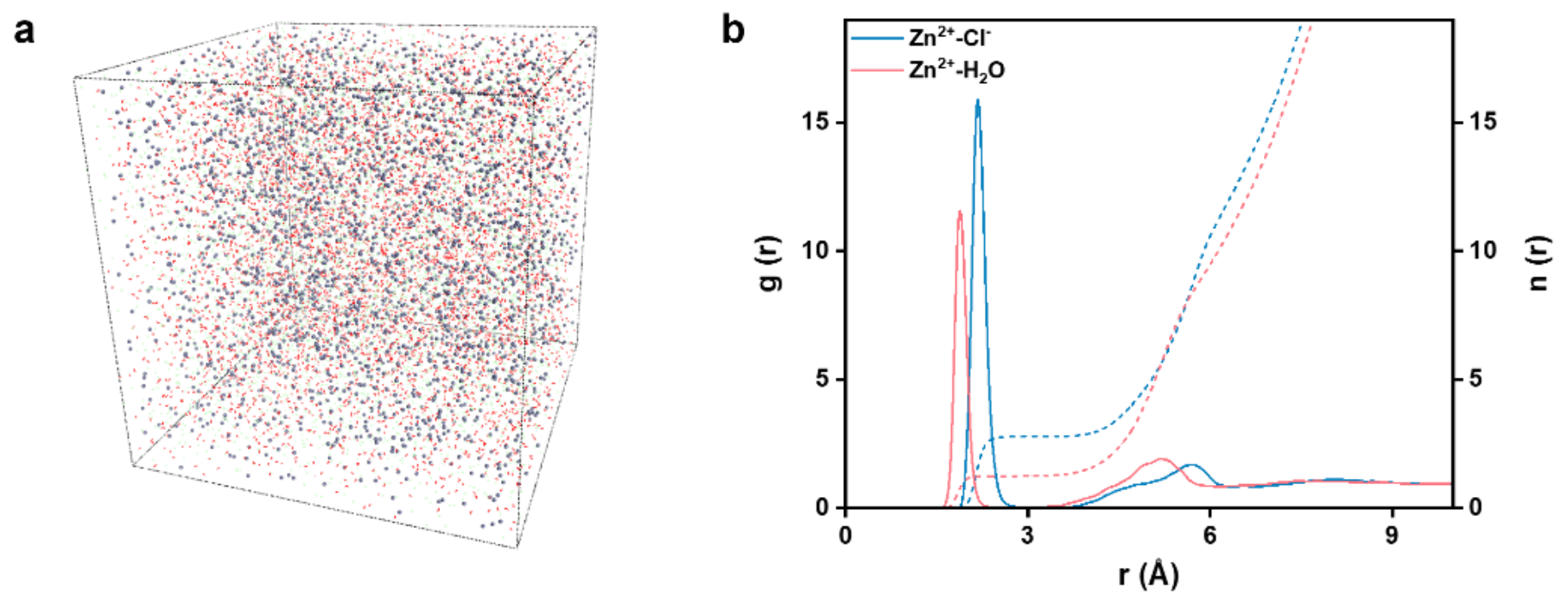

Fig. R32 Theoretical characterization of the bulk properties of the high-concentration $ZnCl_2$ electrolyte (30m $ZnCl_2$)**.** a, MD snapshot of the high-concentration $ZnCl_2$ electrolyte. b, Corresponding radial distribution function (RDF) and coordination number profiles of the high-concentration $ZnCl_2$ electrolyte.

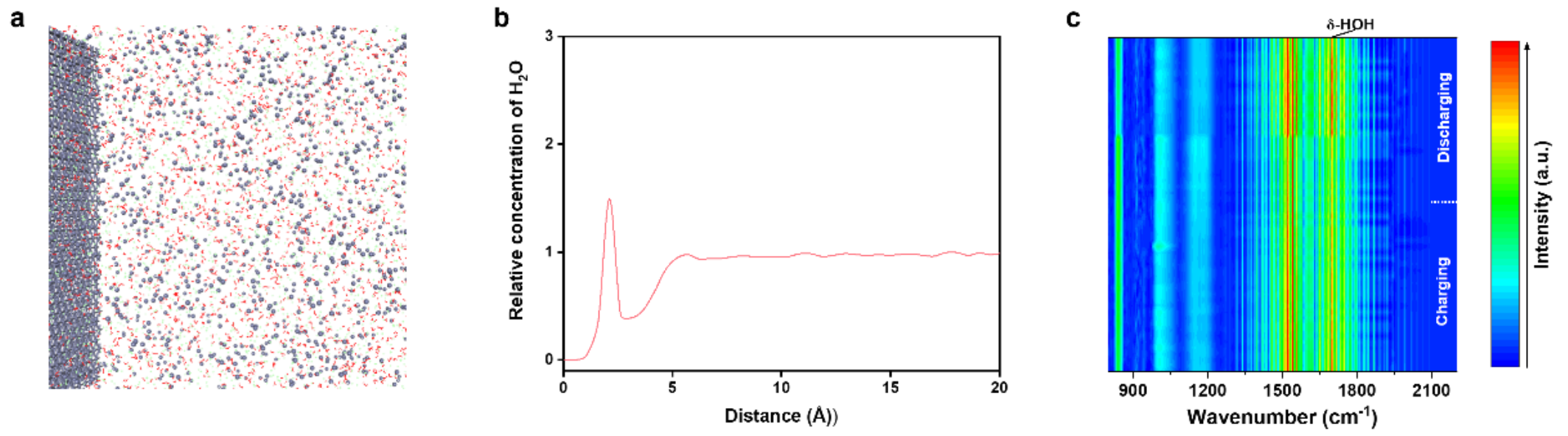

Fig. R33 Theoretical characterization of the interfacial properties of the high-concentration $ZnCl_2$ electrolyte (30m $ZnCl_2$)**.** a, MD snapshot of the interfacial structure in the high-concentration $ZnCl_2$ electrolyte. b, Corresponding interfacial cumulated density profiles of H_2O in the high-concentration $ZnCl_2$ electrolyte. c, *In situ* ATR-IR spectra in the high-concentration $ZnCl_2$ electrolyte.

Comment 2. I am worried that Figure 6f is overclaimed. The cost of LIB based on LFP is very, very cheap nowadays. This relies on two points, one is its very high energy density (denominator), the other is its very mature supply chain, leading to a low BOM (Numerator). The current aqueous batteries, with a much lower energy density (the

energy density of LPF-based LIB can be over 400Wh/L), make it impossible to have a lower Wh cost. I suggest the authors do a more realistic comparison instead of an exaggerated one. In fact, cost can be even more difficult than performance.

Response: We sincerely thank the reviewer for this insightful and constructive comment. We fully agree that LIBs, particularly LFP-based systems, benefit from their high energy density, highly optimized supply chain, and continually improving manufacturing processes, all of which contribute to progressively lower practical cell costs. However, we would like to clarify that the primary purpose of our techno-economic analysis is not to claim immediate cost superiority of 4e ZIBs over commercial LIBs, nor to suggest that the proposed system can currently surpass commercial LIBs. Instead, our analysis aims to benchmark the cost potential of the KA–PA–Nuc electrolyte relative to traditional halogen-containing electrolytes. Notably, all cost calculations were performed using the BatPaC model within a consistent and standardized modelling framework, which calculates cell costs using realistic electrochemical inputs and practical manufacturing data, ensuring the reliability and comparability of the output data. Among these data in Fig. 6, costs of LIBs were taken directly from BatPaC outputs without modification, while the KIB data were adopted from a publication that also used BatPaC model.

The key conclusion we aim to emphasize is that the KA–PA–Nuc electrolyte markedly reduces the cell cost of 4e ZIBs compared with halogen-containing electrolytes, primarily due to the elimination of costly high-concentration halogen electrolytes and expensive titanium current collectors. This highlights the substantial potential for cost reduction in the material and process levels by the KA-PA-Nuc electrolyte. In addition, we fully understand the reviewer's concern regarding the interpretation of the cell-cost comparison. Our intent is not to position emerging aqueous systems against mature LIB technologies, but rather to highlight the practical feasibility and cost potential of the KA–PA–Nuc electrolyte in advancing aqueous metal–iodine batteries. To avoid any potential misunderstanding or overclaiming, we

have added explicit clarification in the revised manuscript: "*Importantly, although the calculated cell cost of 4e ZIB appears lower than that of LIBs, this reflects a theoretical cost potential rather than immediate practical cost competitiveness, given the highly mature supply chain and manufacturing ecosystem of LIBs*."

Comment 3. The CEs, revealed in figures 4 and 6, are all very low. What are the reasons, the cathode, anode or other components?

Response: The relatively low CEs observed in Figures 4 and 6 are indeed noteworthy. This phenomenon primarily arises from the intrinsic characteristics of high-valent iodine redox process, which inherently involve multi-step electron transfer and transient intermediate species. Similar behavior has been widely reported in previous studies employing high-concentration, halogen-containing electrolytes, reflecting the inherent complexity and intrinsic instability of high-valent iodine redox chemistry.

As shown in Figure 4a, our low-concentration, halogen-free KA–PA–Nuc electrolyte (containing SO_4^{2-}) exhibits a low CE only in the initial cycle, followed by rapid stabilization with high Coulombic efficiency and excellent reversibility in subsequent cycles. This indicates that the interfacial microenvironment gradually equilibrates and that parasitic side reactions are effectively suppressed by the KA–PA–Nuc electrolyte. In contrast, electrolytes containing other anions display persistently low CEs due to they failed to regulate the hydrogen-bond network between water molecules and Nuc species, resulting in poorer reversibility (Fig. 4b–c). These results further highlight the critical role of SO_4^{2-} in disrupting water-Nuc interactions and stabilizing four-electron iodine conversion reactions.

In addition, we have identified another factor related to cell assembly that has a significant impact on the reversibility and stability of the battery. In the initial process, the iodine cathode was simply placed on the titanium mesh, and interfacial contact was achieved only through the pressure applied during cell sealing. This could cause minor

interfacial nonuniformities, affecting reversibility. In the revised procedure, we pre-pressed the iodine cathode into the titanium mesh using a roller press prior to assembly, which significantly improved the electrode–collector interface, resulting in markedly enhanced Coulombic efficiency and cycling stability. Accordingly, the data in Figure 6 has been updated in the revised manuscript to reflect these improvements.

Comment 4. In addition to the interfacial microenvironment, the solvation structure within the bulk electrolyte plays a critical role in governing electrochemical performances. Therefore, comprehensive investigations of the bulk properties (such as ion solvation structure and coordination environment) across different electrolyte systems must be provided.

Response: Following the suggestion, we have conducted comprehensive analyses of the bulk solvation structures across different electrolytes to complement our discussion about the interfacial microenvironment. MD simulations of the bare $ZnSO_4$ (KA) electrolyte reveal a typical solvation structure, in which Zn^{2+} is predominantly coordinated by water molecules with a Zn^{2+}-H_2O coordination number of approximately 6 (Fig. R34). Upon introducing different anions such as $CF_3SO_3^-$, CF_3COO^-, or CH_3COO^-, the overall solvation structure remains similar, with water molecules dominating the Zn^{2+} primary solvation sheath. Minor variations arise in the KA–CH_3COO^- electrolyte, where partial anion participation slightly reduces the Zn^{2+}-H_2O coordination number, suggesting limited anion incorporation into the solvation sheath (Fig. R35). Importantly, the addition of Nuc species does not significantly alter the primary solvation structure of the corresponding electrolytes (Fig. R36).

These results indicate that, despite subtle differences in anion participation, all electrolytes remain water-dominated bulk solvation structures. In addition, it is crucial to note that the iodine conversion reaction is primarily governed by interfacial microenvironment rather than bulk property. As demonstrated by MD simulations, *in situ* ATR-IR spectra, and *in situ* UV-vis spectra, electrolytes such as KA- $CF_3SO_3^-$-Nuc,

KA-CF_3COO^--Nuc, and KA-CH_3COO^--Nuc possess similar bulk solvation structures but exhibit drastically different electrochemical behaviors due to distinct interfacial microenvironments. This also explains why the high-concentration $ZnCl_2$ electrolyte still shows inferior electrochemical stability due to water-enriched interfacial microenvironment despite its anion-enriched solvation structure. Therefore, the bulk solvation analysis further highlights that the interfacial microenvironment dictated by anion polarity plays the decisive role in governing iodine conversion chemistry and electrochemical performance. Accordingly, related data and discussion have been incorporated into the revised manuscript: "*Molecular dynamics (MD) simulations were employed to visualize both the bulk properties and interfacial behavior of baseline electrolytes containing anions of varying polarity. Although subtle differences in anion participation, all electrolytes remain water-enriched bulk solvation structures (Supplementary Fig. 19-21). In contrast to the bulk properties, the interfacial microenvironment exhibits pronounced differences (Fig. 3d)*".

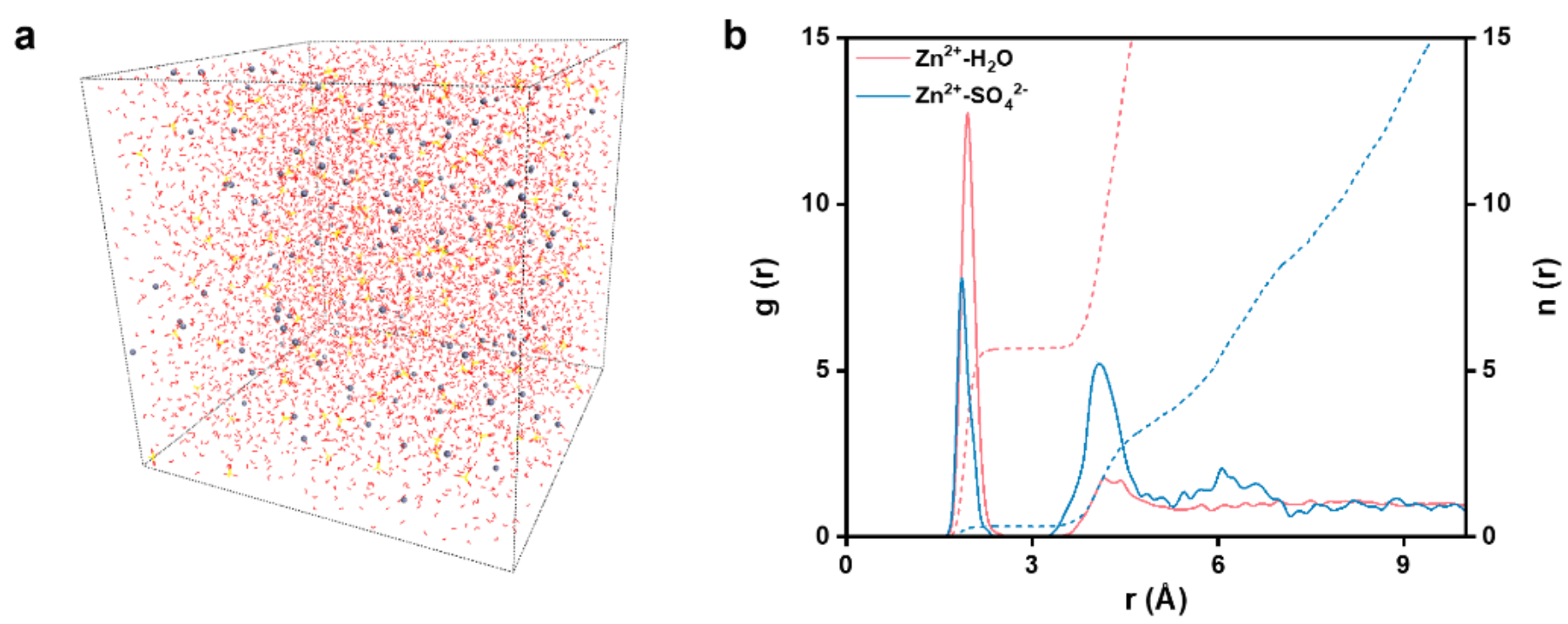

Fig. R34 Theoretical characterization of the bulk properties of the bare $ZnSO_4$ (KA) electrolyte. a, MD snapshot of the bare KA electrolyte. b, Corresponding radial distribution function (RDF) and coordination number profiles of the bare KA electrolyte.

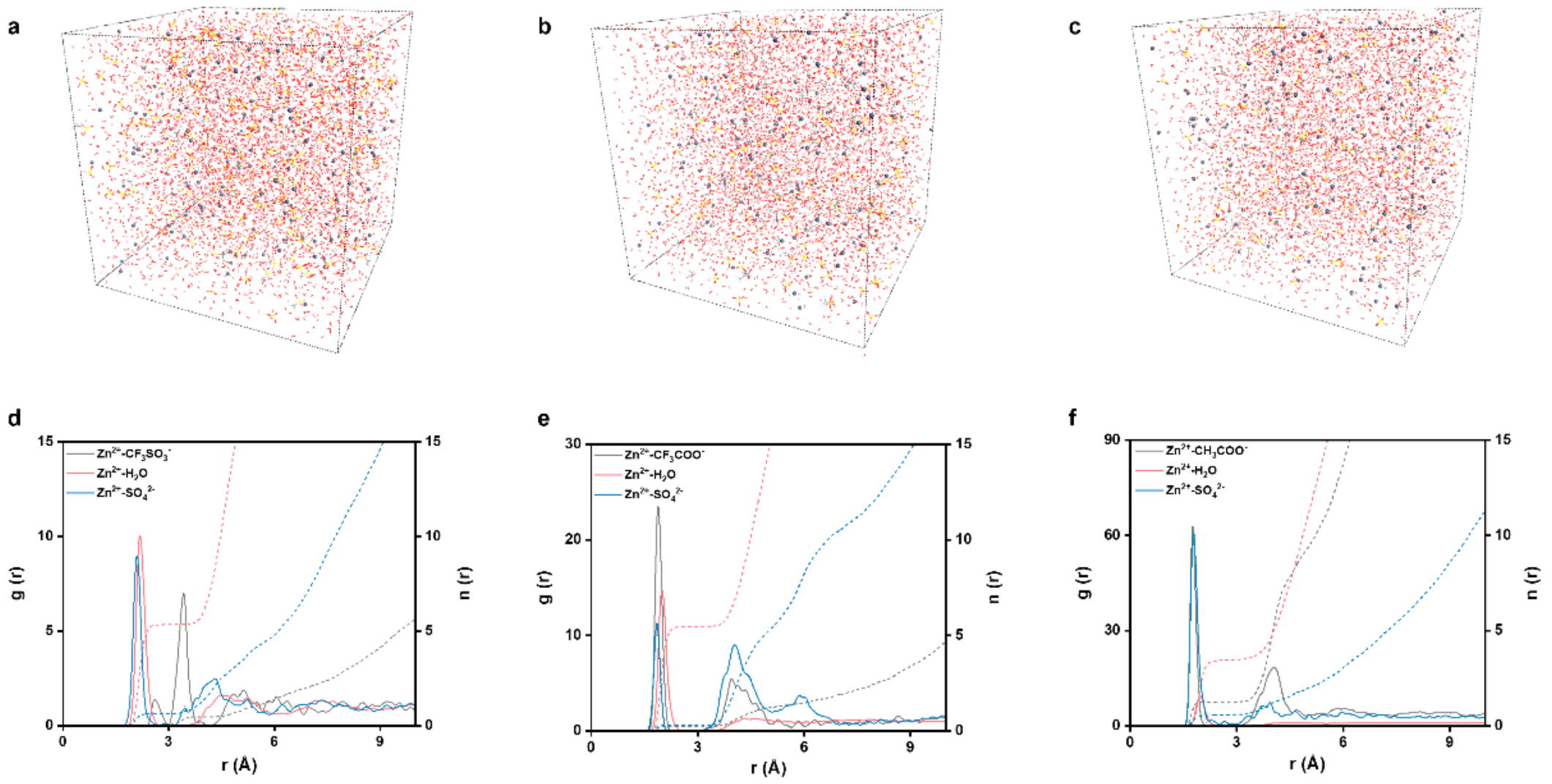

Fig. R35 Theoretical characterization of the bulk properties of the KA electrolyte with different polar anions. a-c, MD snapshot of the KA electrolyte with $CF_3SO_3^-$, CF_3COO^-, CH_3COO^-, respectively. d-e, Corresponding radial distribution function (RDF) and coordination number profiles of the KA electrolyte with $CF_3SO_3^-$, CF_3COO^-, CH_3COO^-, respectively.

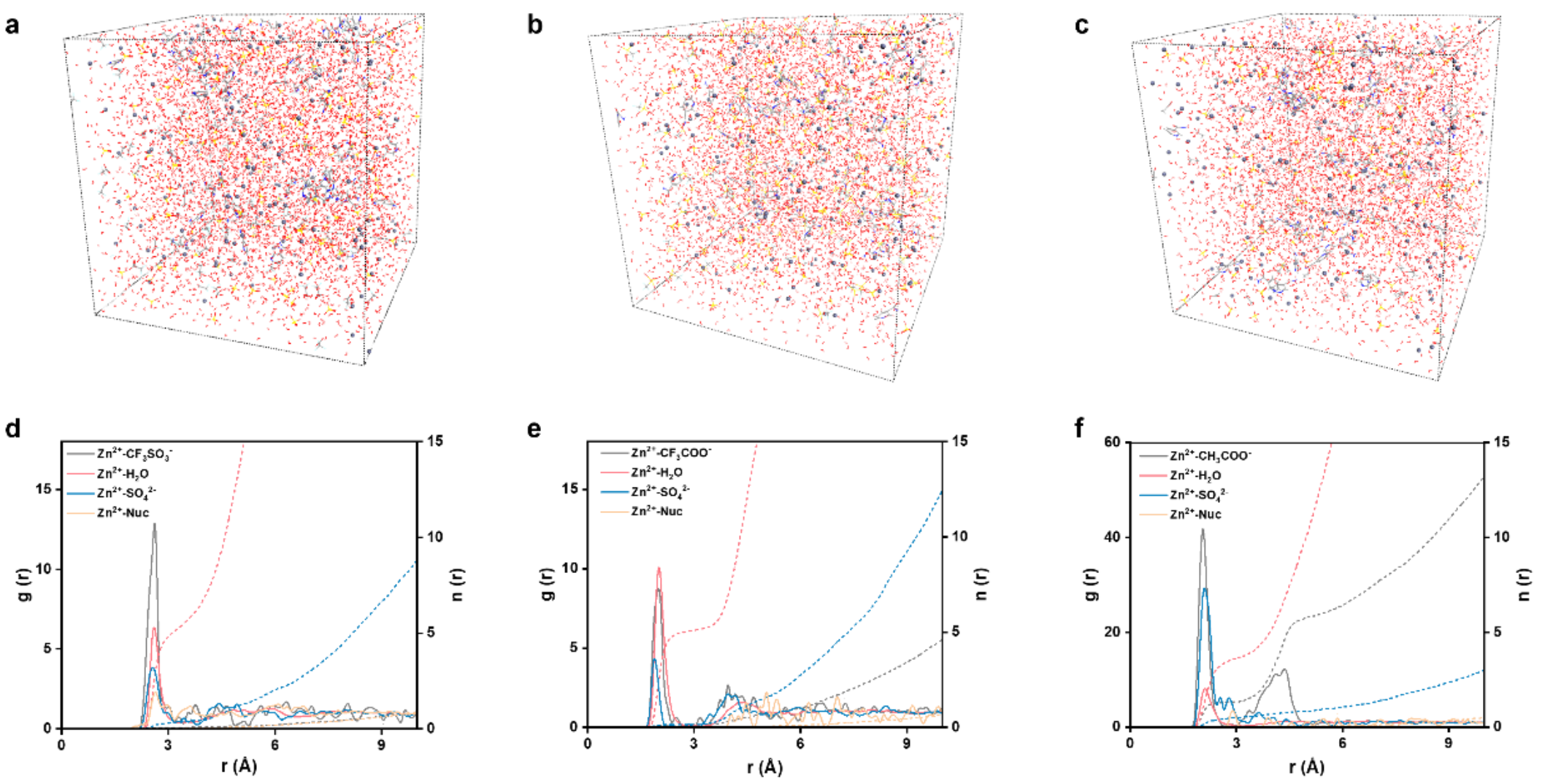

Fig. R36 Theoretical characterization of the bulk properties of the KA-Nuc electrolyte with different polar anions. a-c, MD snapshot of the KA-Nuc electrolyte with $CF_3SO_3^-$, CF_3COO^-, CH_3COO^-, respectively. d-e, Corresponding radial distribution function (RDF) and coordination number profiles of the KA-Nuc

electrolyte with $CF_3SO_3^-$, CF_3COO^-, CH_3COO^-, respectively.

Comment 5. The authors present the final optimized electrolyte formulation without detailing the rationale or optimization process behind its selection, lacking an explanation of how electrolyte optimization could address critical issues in four-electron iodine redox reactions.

Response: We had previously carried out systematic electrolyte optimization, which was not included in the original manuscript. According to the suggestion, we have now incorporated the corresponding data and discussion into the revised manuscript.

As shown in Fig. R37, the concentrations of KA, PA, and Nuc were systematically optimized to balance redox reversibility, capacity, and cycling stability. Specifically, the Coulombic efficiency of the batteries gradually increases with increasing KA concentration, while the specific capacity and long-term stability first improve and then decline when excessive KA is introduced (Fig. R37a, d). This indicates that an overly high KA concentration diminishes the functional contribution of PA, which plays a crucial role in tuning the interfacial environment. In contrast, increasing the PA concentration enhances the specific capacity by promoting the I^0/I^+ redox process. However, excessive PA leads to decreased Coulombic efficiency and cycling stability, likely because it sacrifices the functional contribution of KA in regulating anion–water–Nuc interactions (Fig. R37b, e).

Finally, by fixing the concentrations of KA and PA, the concentration of Nuc was investigated. The specific capacity, Coulombic efficiency, and cycling stability initially increase and then stabilize with increasing Nuc concentration, suggesting that an optimal Nuc content effectively participates in halogen-bond formation during the I^0/I^+ conversion reaction (Fig. R37c, f). Therefore, through this systematic optimization, the final KA–PA–Nuc electrolyte formulation was identified, which shows a well-balanced combination of redox reversibility, capacity, and cycling stability, thereby enabling

reversible four-electron iodine conversion reactions. Therefore, corresponding data and discussion have been added into the revised manuscript: "*As a result, a new low-concentration, halogen-free electrolyte standard, termed KA-PA-Nuc, is proposed and optimized (Supplementary Fig. 2-3)*".

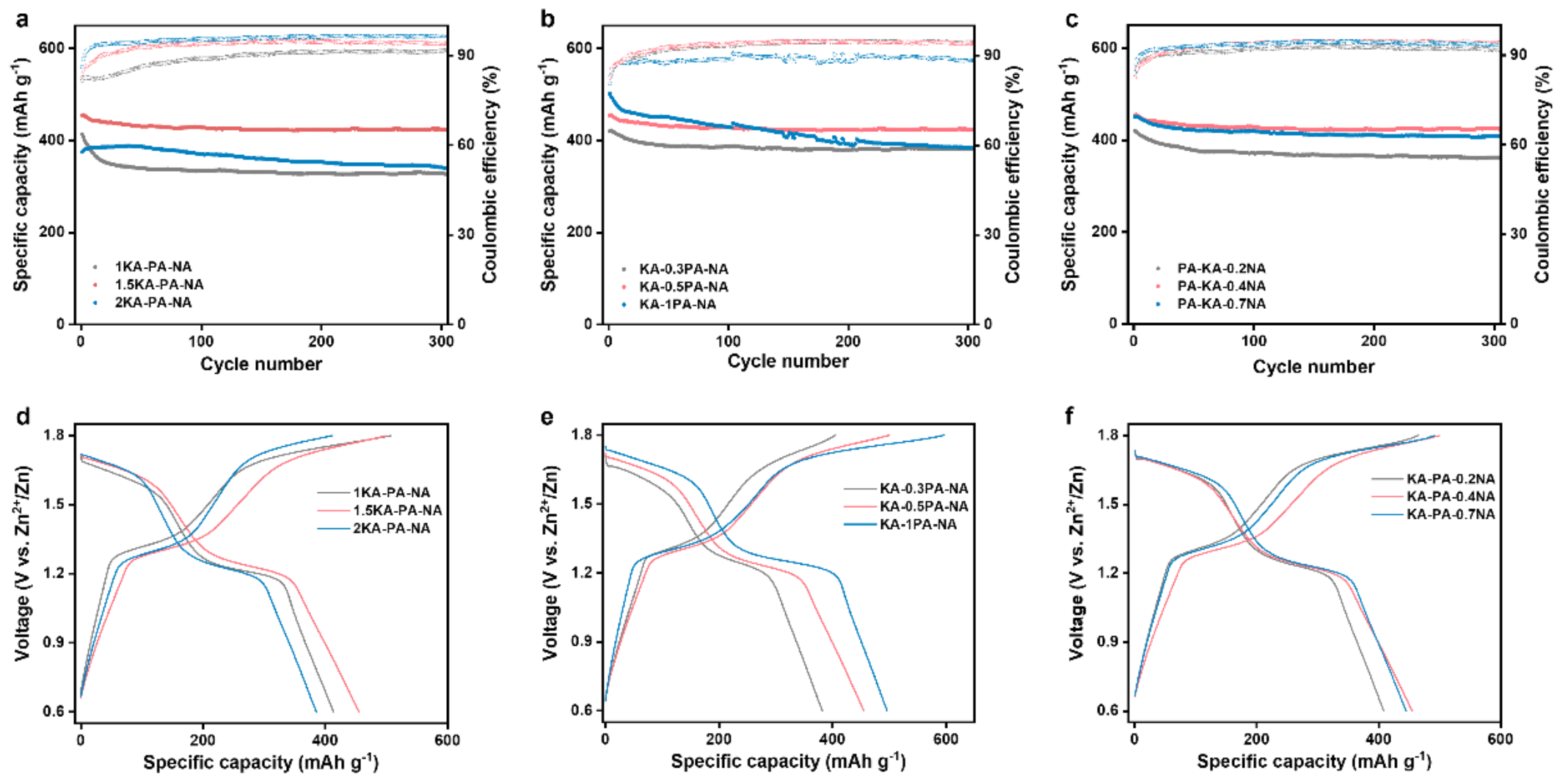

Fig. R37 Electrolyte optimization and performance evolution. a, Cycling stability in electrolytes with different KA concentrations. b, Cycling stability in electrolytes with different PA concentrations. c, Cycling stability in electrolytes with different Nuc concentrations. d, Corresponding GCD profiles in electrolytes with different KA concentrations. e, GCD profiles in electrolytes with different PA concentrations. f, GCD profiles in electrolytes with different Nuc concentrations.

Comment 6. The I^+ cation is well known to undergo hydrolysis in aqueous environments, posing a major challenge for achieving stable multi-electron iodine redox reactions. The authors propose that the electrophilic I^+ species is stabilized via the formation of linear halogen bonds with nucleophilic components in the designed electrolyte. However, this important claim warrants further evidence. Additional mechanistic insights—such as spectroscopic validation, structural characterization, or theoretical modeling—would be valuable to substantiate the proposed halogen-bonding interactions and their role in stabilizing I^+ in aqueous media.

Response: To substantiate the proposed halogen bond stabilization mechanism, we performed comprehensive electrochemical, spectroscopic, and theoretical investigations that collectively provide efficient evidence for the Nuc–I^+–Nuc halogen bond interaction. First, control electrolytes without Nuc species show no reduction peak or discharge plateau associated with the I^0/I^+ redox process (Fig. R2). The absence of I^+-related signals across all Nuc-free electrolytes demonstrates that I^+ cannot be stabilized by anions alone. By contrast, the presence of nucleophilic species enables successful I^+ conversion reaction, providing strong evidence that halogen bond interaction with Nuc species is essential for I^+ stabilization.

To directly probe halogen bond interaction, 2D ^1H-^{15}N HMBC NMR was employed, which is a sensitive tool for investigation of halogen bond formation. Pyridine was selected as a representative Nuc species. In the bare pyridine solution, coupled H2/H6-N1 and H3/H5-N1 signals appear at 314.78 ppm (Fig. R3a). Upon addition of molecular iodine (I_2), a small ^{15}N chemical shift is observed (Δ_{15N} = -22.76 ppm), consistent with weak interaction between pyridine and iodine (Fig. R3b). In sharp contrast, when pyridine is exposed to iodonium (I^+) species generated by an exchange reaction with corresponding silver analogues (Fig. R6a), a large ^{15}N chemical shift (Δ_{15N} = -107.84 ppm) is detected (Fig. R3c). This magnitude of chemical shift is characteristic of halogen bond formation, confirming direct Nuc–I^+ interaction and providing unambiguous spectroscopic evidence for the halogen bond stabilization mechanism.

To further identify the specific coordination configuration, binding energy calculations were conducted to evaluate possible geometries in our electrolyte system. The results reveal that the linear Nuc–I^+–Nuc halogen bond, accompanied by a counterion, is the most stable structure, exhibiting lower binding energy than anion-involved configurations (A^-–I^+–A^- and Nuc–I^+–A^-) (Fig. R4). These findings indicate that the linear Nuc–I^+–Nuc halogen bond is thermodynamically preferred, while anions only serve as counterions and do not directly coordinate with I^+. Moreover, when serving as counterions, different anions have negligible influence on the N–I bond

length or binding energy, further supporting their non-coordination in halogen bond formation (Fig. R5). This observation is fully corroborated by additional 2D ^{1}H-^{15}N HMBC spectra, where the ^{15}N chemical shifts of Nuc–I^+–Nuc complexes remain essentially unchanged across various counterions (Fig. R6). Therefore, the preferred coordination configuration in our electrolyte system is the linear Nuc–I^+–Nuc halogen bond.

In situ Raman spectra provides further validation. In the KA–PA electrolyte without Nuc species, no characteristic peak corresponding to I^+ complex appears during charge/discharge cycle (Fig. R30). By contrast, the KA–PA–Nuc electrolyte exhibits a pronounced and reversible Raman peak associated with Nuc–I^+–Nuc complex at ~210 cm^{-1} (Supplementary Fig. 49). Collectively, these electrochemical, spectroscopic, and theoretical results provide multiple evidence for the linear Nuc–I^+–Nuc halogen bond stabilization mechanism underlying the reversible four-electron iodine conversion reactions. Therefore, corresponding data and discussion about halogen-bond stabilization mechanism have been added into the revised manuscript: "*These halogen bonds are the foundation of four-electron iodine conversion reactions, which has been fully demonstrated through electrochemical comparison in Nuc-free electrolytes, nuclear magnetic resonance (NMR) spectroscopy, and theoretical calculations (Supplementary Fig. 6-10)*".

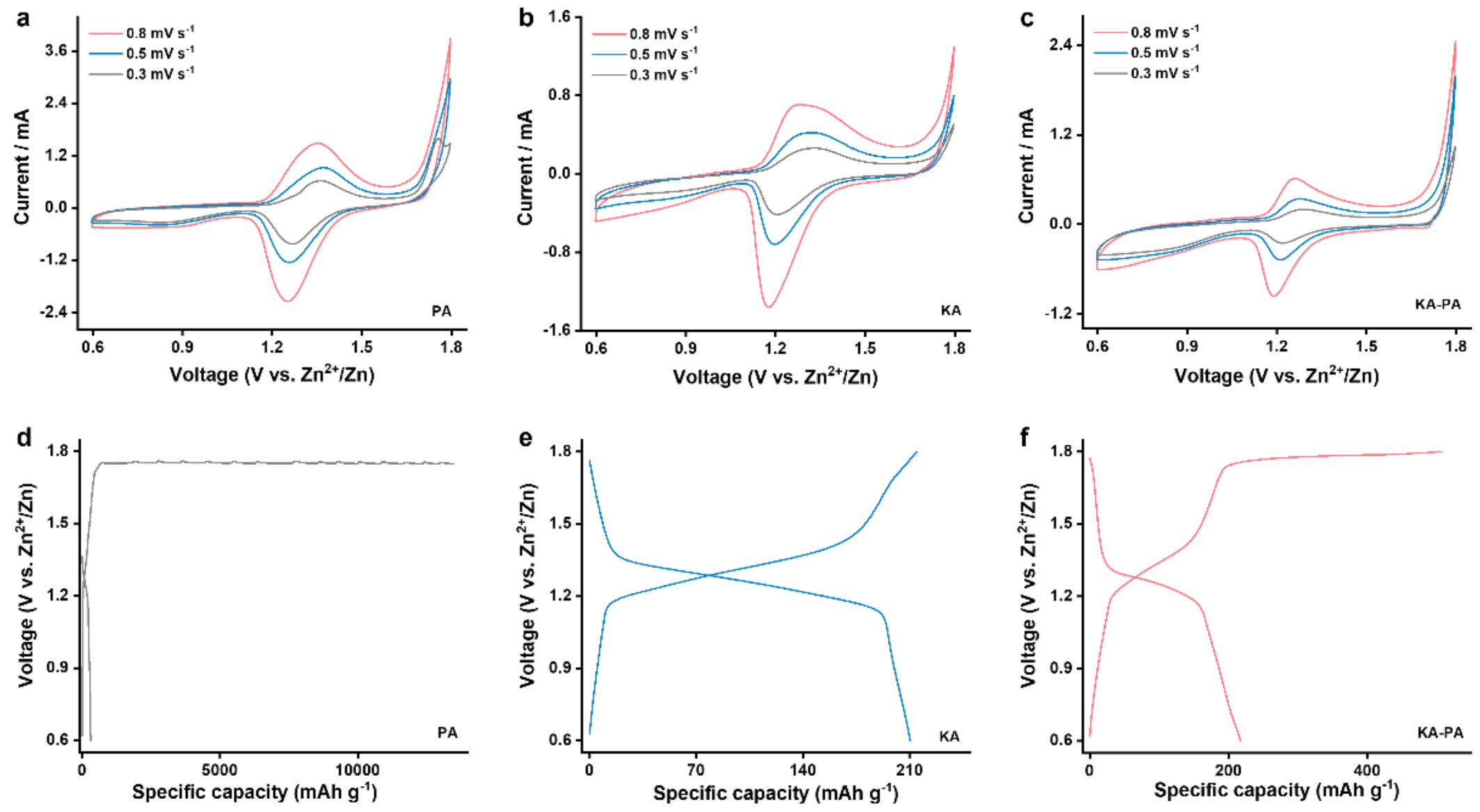

Fig. R2 Electrochemical performance of electrolytes without Nuc species. a-c, CV profiles of the iodine conversion reaction in PA, KA, and KA-PA electrolytes at various scan rates. d-f, GCD profiles of the iodine conversion reaction in PA, KA, and KA-PA electrolytes at 1 A g^{-1}.

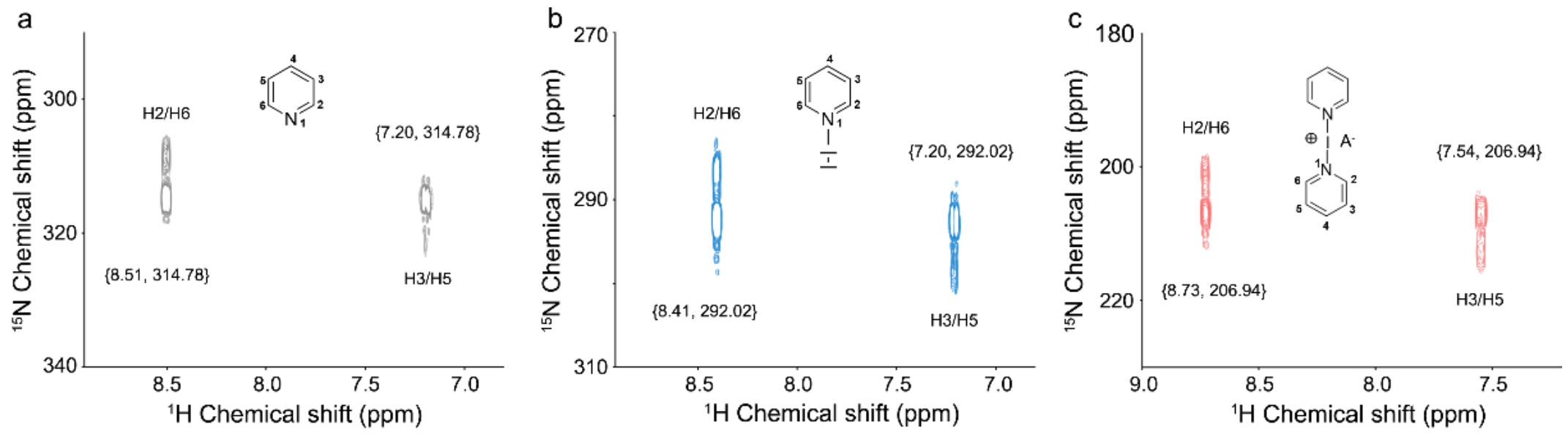

Fig. R3 Characterization of halogen bonds. a, 2D ^{1}H-^{15}N HMBC NMR spectrum of the bare Nuc solution (pyridine). b, 2D ^{1}H-^{15}N HMBC NMR spectrum of the pyridine solution with iodine. c, 2D ^{1}H-^{15}N HMBC NMR spectrum of the synthesized I^{+} complexes.

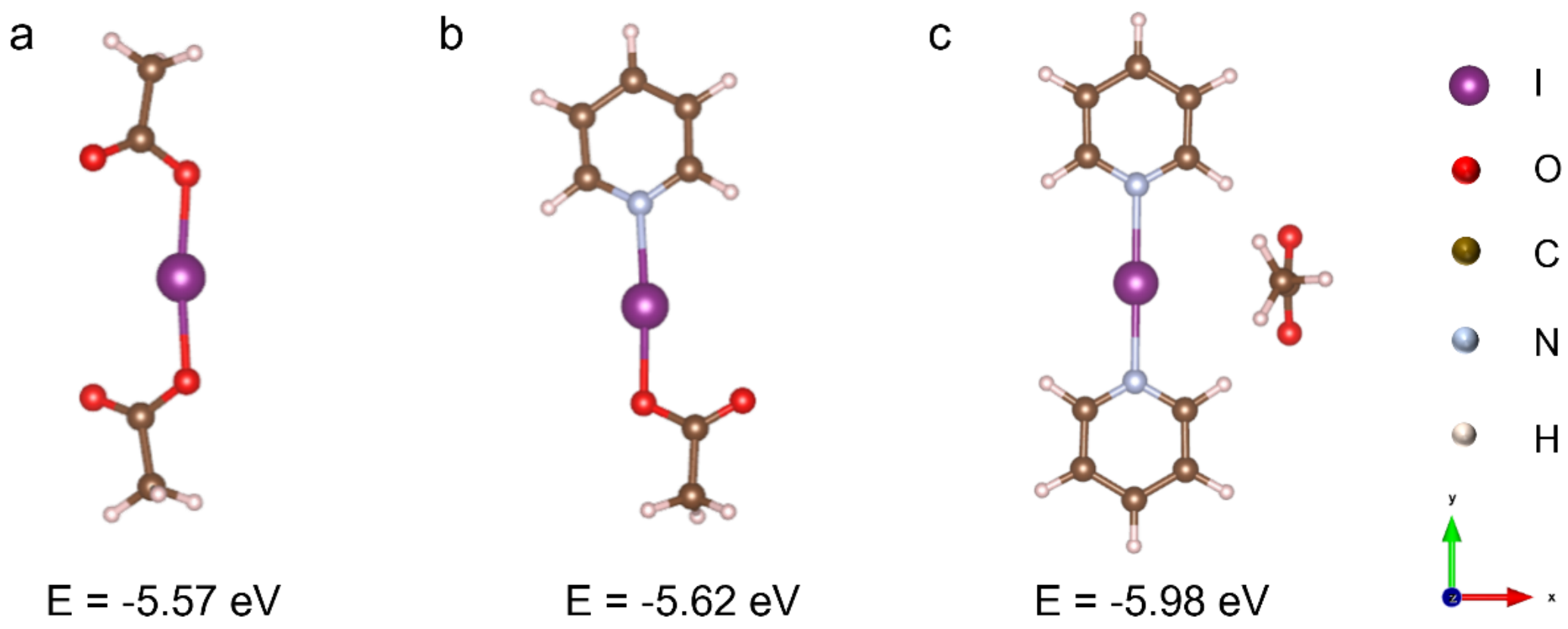

Fig. R4 Theoretical calculation of possible coordination configurations. a, The A^-–I^+–A^- coordination structure and corresponding binding energy. b, The Nuc–I^+–A^- coordination structure and corresponding binding energy. c, The Nuc–I^+–Nuc halogen bond and corresponding binding energy.

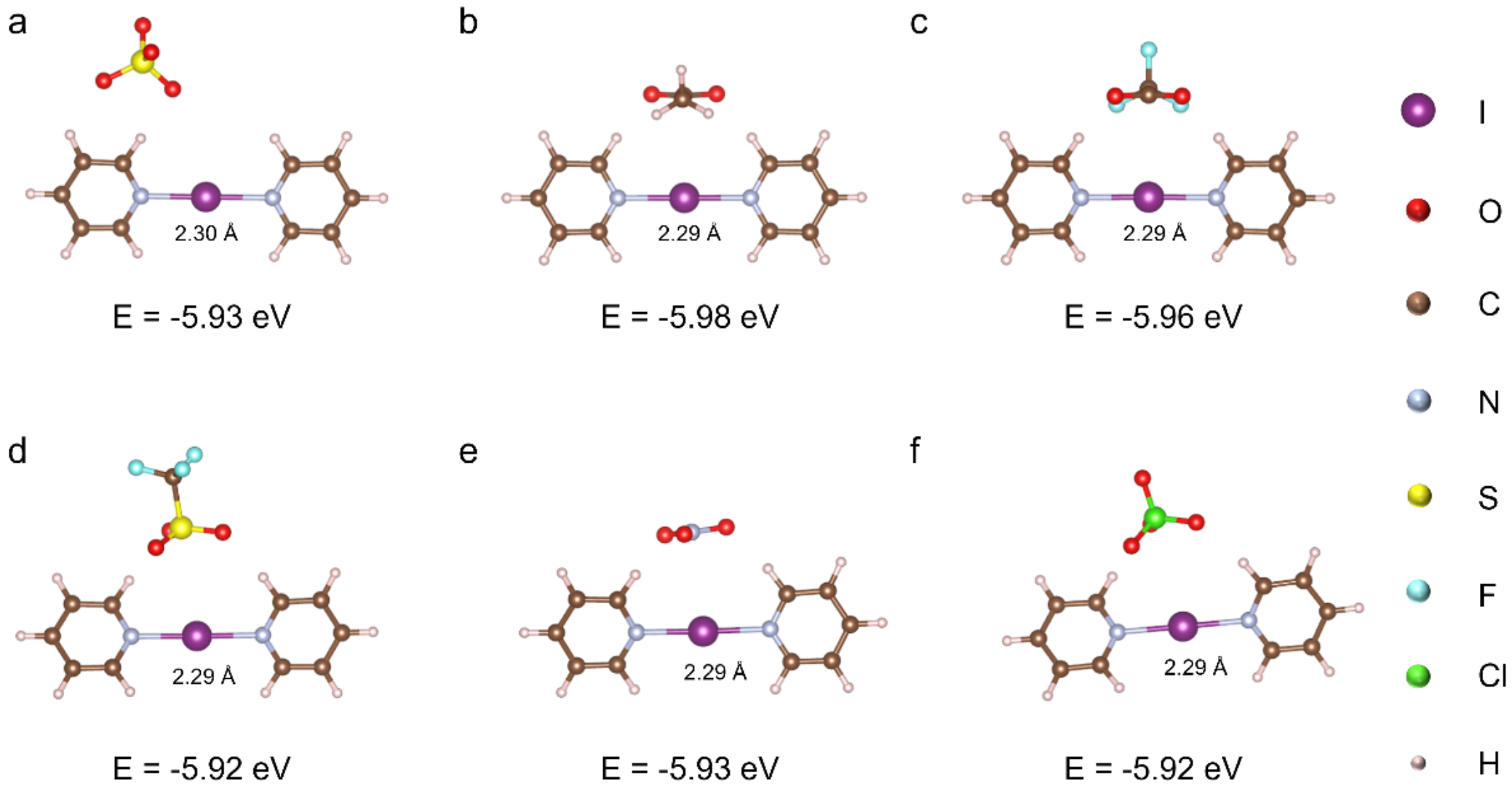

Fig. R5 Binding energy and bond length of Nuc–I^+–Nuc halogen bonds with different counterions. a, SO_4^{2-}. b, CH_3COO^-. c, CF_3COO^-. d, $CF_3SO_3^-$. e, NO_3^-. f, ClO_4^-.

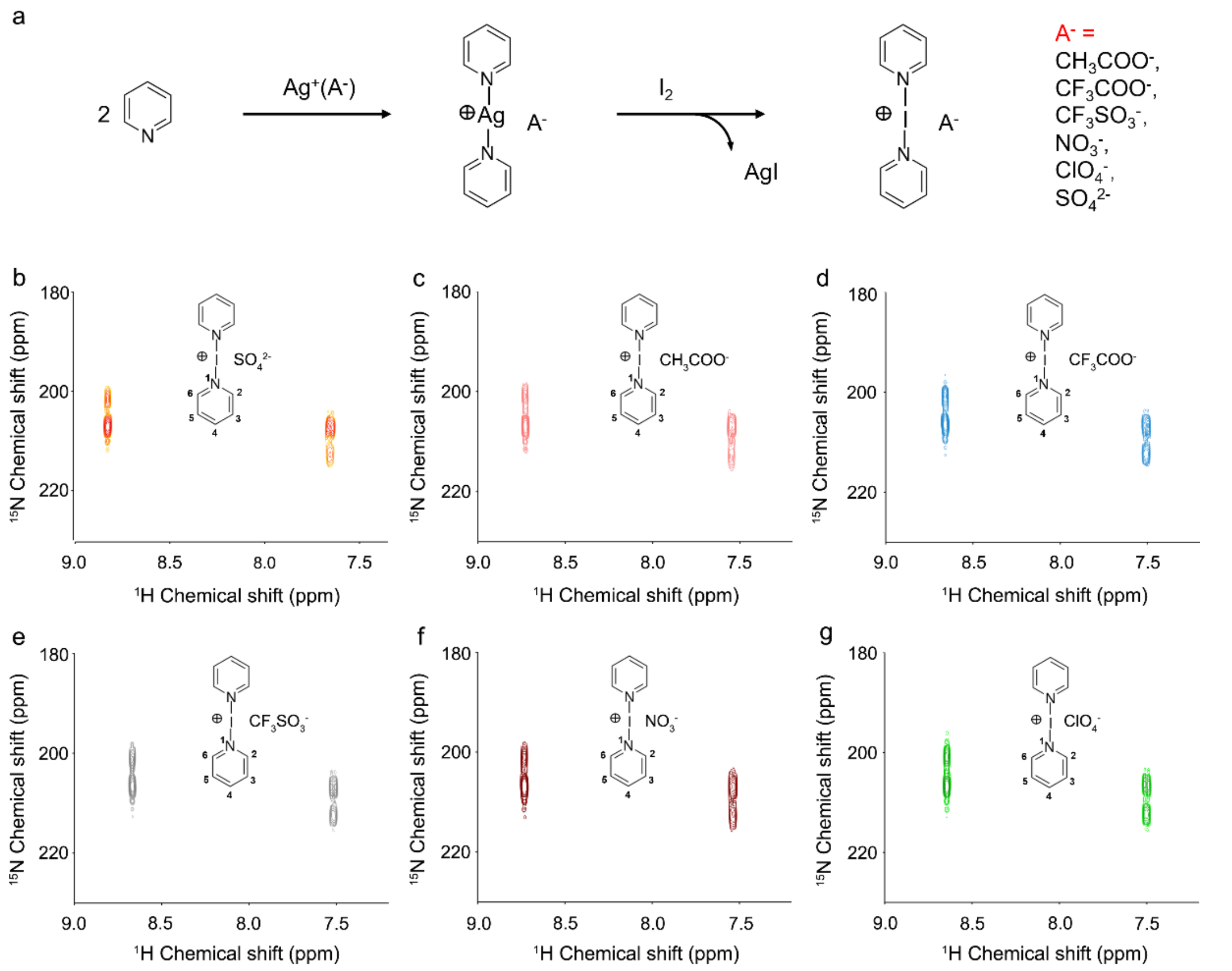

Fig. R6 Characterization of halogen bonds with different counterions. a, Schematic illustration of the synthesis of I^+ halogen bond complexes with different counterions via a cation exchange reaction. 2D ^1H-^{15}N HMBC NMR spectrum of the synthesized I^+ complexes with different counterions: b, SO_4^{2-}; c, CH_3COO^-; d, CF_3COO^-; e, $CF_3SO_3^-$; f, NO_3^-; g, ClO_4^-.

Comment 7. In the context of linear halogen bonding, the iodine cation (I^+) carries a positive charge, while the two coordinating nucleophilic species are electrically neutral. As a result, the resulting halogen-bonded complex is positively charged and requires counterions for charge compensation. This raises an important question regarding the role of anions in the proposed electrolyte system. Beyond modulating the interfacial microenvironment and influencing molecule-water interactions, do the anions also contribute to charge stabilization or participate in other critical functions?

Response: We sincerely appreciate the reviewer for this insightful and constructive comment regarding the role of anions in our electrolyte system. As detailed in our response to Comment 6, the I^+ stabilization mechanism is proved by the linear Nuc–I^+–Nuc halogen bond. Importantly, these analyses consistently show that anions do not directly coordinate to I^+. Specifically, binding-energy calculations indicate that the Nuc–I^+–Nuc configuration with counterion is more stable than anion-coordinating alternatives, while anions acting as counterions exhibit negligible influence on N–I bond length or binding energy (Fig. R4-5). These theoretical results are fully supported by 2D ^1H–^{15}N HMBC spectra, where the ^{15}N chemical shifts of Nuc–I^+–Nuc complexes remain essentially unchanged across different counterions, confirming that anions do not contribute to additional function in halogen bond complexes beyond their role as counterions (Fig. R6).

Although anions don't directly participate in the halogen bond mechanism, they play critical roles in modulating the interfacial microenvironment by creating water-deficient, anion-enrichment interface, and tuning hydrogen bond networks and Nuc availability. These effects collectively establish the necessary physicochemical conditions for the formation and stabilization of Nuc–I^+–Nuc halogen bond in aqueous system.

Comment 8. When presenting the analysis based on the DRT, it is important to also provide the corresponding electrochemical impedance spectroscopy (EIS) data. Analyzing the original EIS spectra alongside the DRT results will enhance the credibility of the interpretation and allow for a more comprehensive understanding of the underlying electrochemical processes.

Response: We fully agree that presenting the original EIS spectra alongside the DRT analysis is essential for a more reliable interpretation and a comprehensive understanding of the interfacial electrochemical processes. In the revised manuscript,

we have substantially expanded both the data presentation and the corresponding discussion. Specifically, we include EIS spectra for relevant electrodes and electrolytes at various charging states and charging rates, together with their corresponding DRT profiles, enabling direct comparison of impedance features and relaxation peak evolution. This integrated EIS–DRT analysis allows to unambiguously assign each relaxation peak to a specific electrochemical process, validate the potential-dependent impedance evolution, and identify the rate-limiting step with improved accuracy. Corresponding data and discussion are provided in the main text (Fig. 5) and in the supporting information (Supplementary Fig. 39-48).

Comment 9. The manuscript employs Ti mesh as the current collector; however, titanium is relatively expensive and may not be suitable for practical, large-scale applications. Could the authors clarify the rationale for selecting Ti mesh in this study? Additionally, it would be valuable to assess whether more cost-effective alternatives-such as stainless steel-could be used without compromising electrochemical performances. This would enhance the practical relevance and scalability of the proposed system. Data with other current collectors should be provided.

Response: Ti mesh was initially chosen to ensure direct comparison with previously reported four-electron iodine conversion systems, where Ti mesh is the most commonly used current collector due to its excellent corrosion resistance in halogen-containing electrolytes. In our study, halogen-containing electrolytes were also employed for comparative analysis. Thus, Ti mesh was adopted to maintain methodological consistency.

In response to the reviewer's suggestion about other current collectors, we have now systematically evaluated the compatibility of the KA–PA–Nuc electrolyte with other commercial current collectors, including carbon paper and stainless steel. When carbon paper was used as the cathode current collector, the Zn–I_2 battery in the KA–

PA–Nuc electrolyte exhibited reversible four-electron iodine conversion reactions and excellent rate capability, delivering reversible capacities of 504, 458, 418, 370, 335, and 310 mAh g^{-1} at current densities of 0.5, 1, 2, 4, 6, and 8 A g^{-1}, respectively (Fig R11a-b). Remarkably, the cell retained 86% of its capacity after 1,000 cycles at 1 A g^{-1}, indicating an outstanding cycling stability (Fig R11d). These results confirm the good compatibility of the KA–PA–Nuc electrolyte with carbon-based current collectors. In contrast, cells using carbon paper in the high-concentration halogen-containing electrolyte (30m $ZnCl_2$) failed to complete even one full cycle due to continuous corrosion of battery cases (Fig R12a). Persistent corrosion was also observed in the low-concentration halogen-containing electrolyte (1.5$ZnCl_2$-0.5PA-0.4Nuc), leading to rapid cell failure (Fig R12d).

Stainless steel, another inexpensive and widely used current collector, was also investigated. When employed as the cathode current collector, the battery in the KA–PA–Nuc electrolyte also displays excellent four-electron iodine conversion reactions, delivering reversible capacities of 501, 435, 380, 326, 293, and 270 mAh g^{-1} at current densities of 0.5, 1, 2, 4, 6, and 8 A g^{-1}, respectively (Fig R11c, e). In addition, a high capacity retention of 87% was maintained after 1,000 cycles at a current density of 1 A g^{-1} (Fig R11f). These results further prove the good compatibility of the KA–PA–Nuc electrolyte with stainless steel. However, batteries with stainless steel failed to operate properly in both high-concentration (30m $ZnCl_2$) and low-concentration halogen-containing electrolytes (1.5$ZnCl_2$-0.5PA-0.4Nuc) due to severe corrosion of both battery cases and stainless steel current collector, even under customized Swagelok cell configurations (Fig R12b-c, e-f).

Collectively, these findings demonstrate that the KA–PA–Nuc electrolyte exhibits broad compatibility with various commercial current collectors without compromising electrochemical performances. In contrast, halogen-containing electrolytes demand specialized cell configurations and expensive, corrosion-resistant materials for stable operation. This comparison underscores the economic and practical advantages of our

low-concentration, halogen-free KA–PA–Nuc electrolyte for cost-effective and sustainable energy storage solutions. Therefore, corresponding data and discussion have been incorporated into the revised manuscript: "*To assess the practical advantages of the KA-PA-Nuc electrolyte, its compatibility with other commercial current collectors was evaluated. In sharp contrast to halogen-containing electrolytes, which induce severe corrosion and rapid cell failure, the KA–PA–Nuc electrolyte exhibits excellent compatibility with various commercial current collectors (including stainless steel) without compromising electrochemical performance. This compatibility eliminates the reliance for specialized cell configurations and expensive titanium current collectors (Supplementary Fig. 58-59)*".

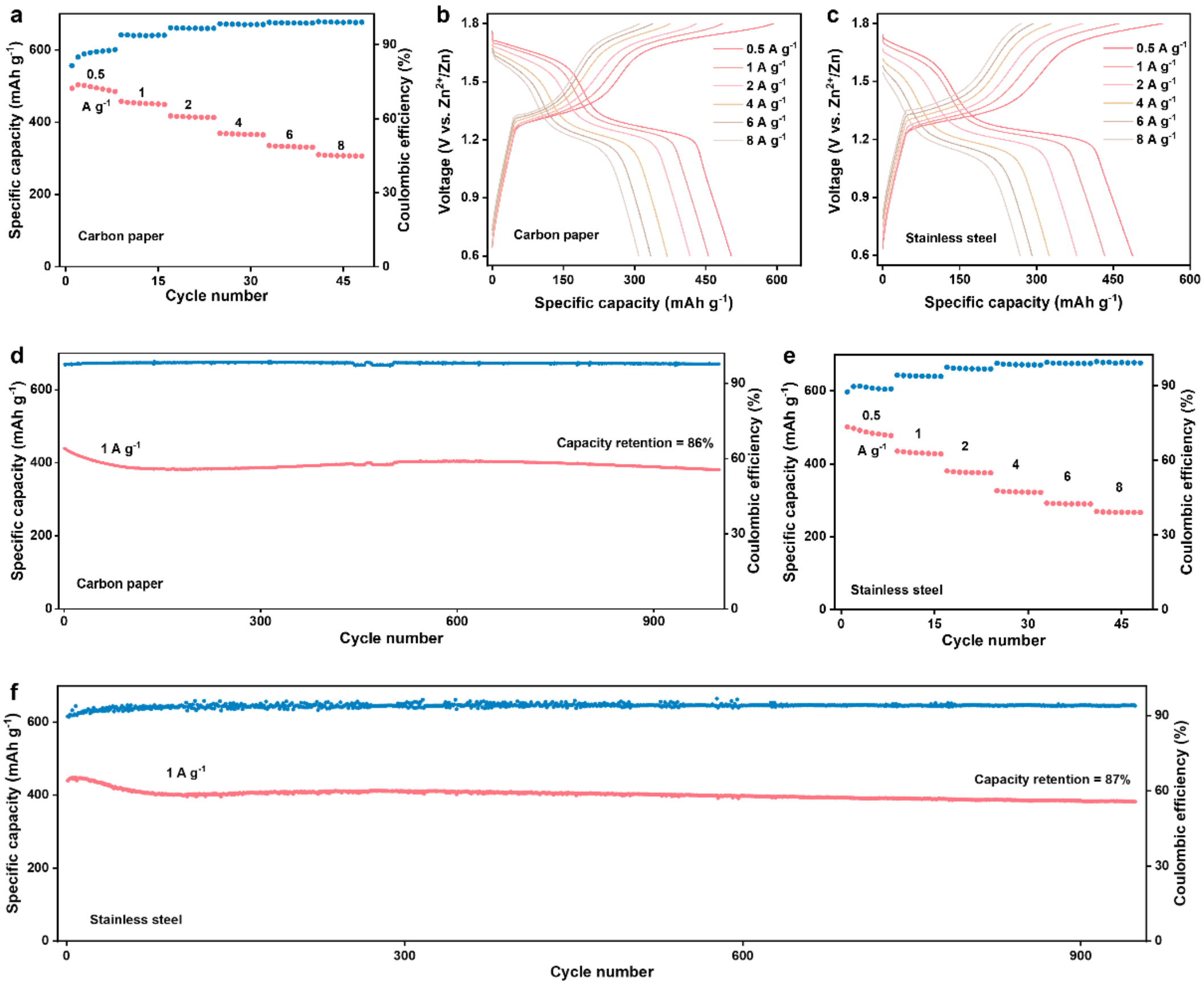

Fig. R11 Compatibility of the KA–PA–Nuc electrolyte with different commercial current collectors. a, Rate performance of Zn-I_2 batteries using carbon paper as the current collector. b, Corresponding GCD profiles of Zn-I_2 batteries using carbon paper as the current collector. c, GCD profiles of Zn-I_2 batteries using stainless steel as the

current collector. d, Cycling performance of Zn-I_2 batteries using carbon paper as the current collector. e, Rate performance of Zn-I_2 batteries using stainless steel as the current collector. f, Cycling performance of Zn-I_2 batteries using stainless steel as the current collector.

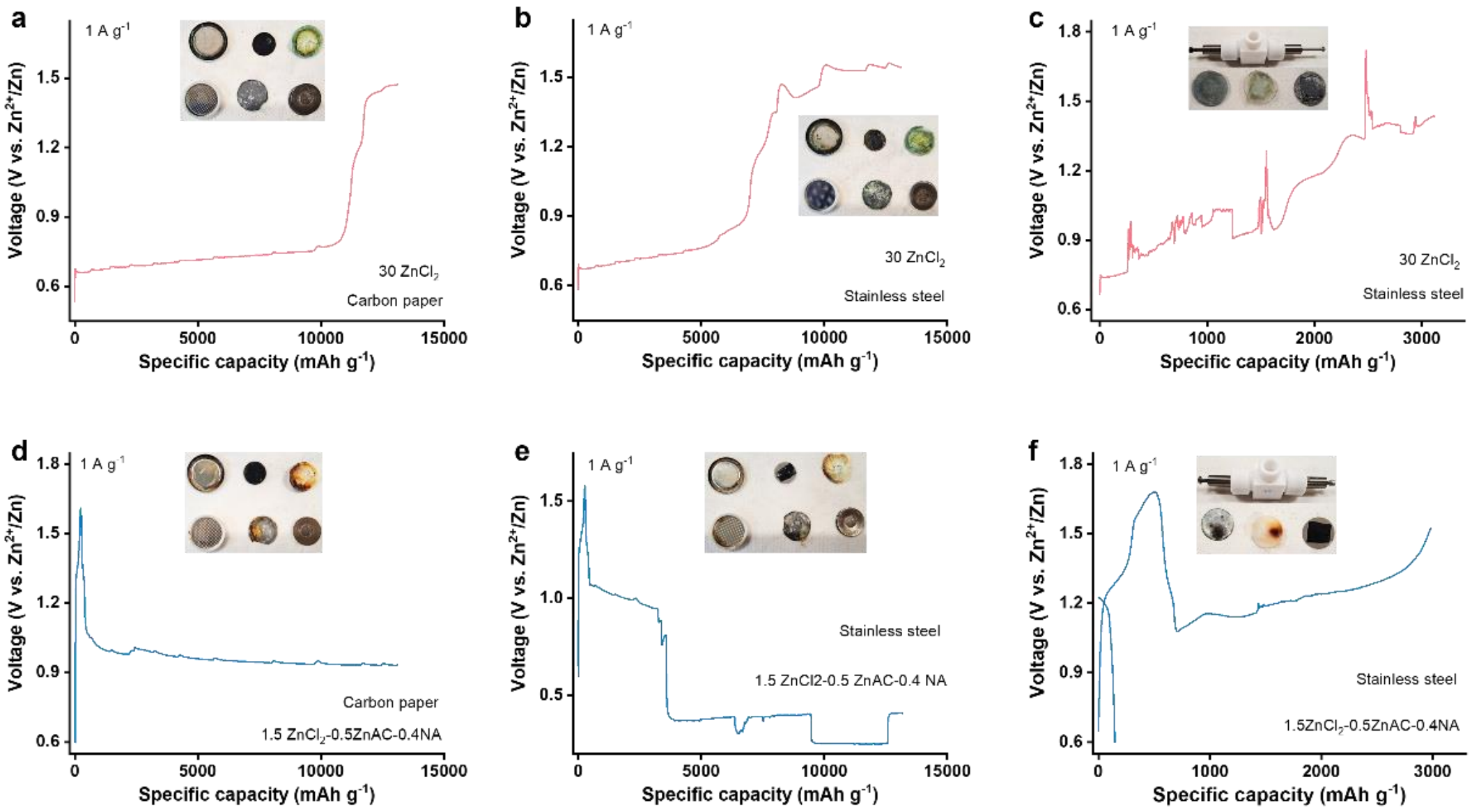

Fig. R12 Electrochemical behavior of different commercial current collectors in halogen-containing electrolytes. a, GCD profiles of Zn-I_2 batteries with 30m $ZnCl_2$ electrolyte using carbon paper as the current collector in the coin cell configuration. b, GCD profiles of Zn-I_2 batteries with 30m $ZnCl_2$ electrolyte using stainless steel as the current collector in the coin cell configuration. c, GCD profiles of Zn-I_2 batteries with 30m $ZnCl_2$ electrolyte using stainless steel as the current collector in the Swagelok cell configuration. d, GCD profiles of Zn-I_2 batteries with 1.5$ZnCl_2$-0.5PA-0.4Nuc electrolyte using carbon paper as the current collector in the coin cell configuration. e, GCD profiles of Zn-I_2 batteries with 1.5$ZnCl_2$-0.5PA-0.4Nuc electrolyte using stainless steel as the current collector in the coin cell configuration. f, GCD profiles of Zn-I_2 batteries with 1.5$ZnCl_2$-0.5PA-0.4Nuc electrolyte using stainless steel as the current collector in the Swagelok cell configuration. Insets show photographs of the corresponding cycled batteries.

Comment 10. To evaluate the performance of the proposed electrolyte standard in multi-electron aqueous Al–I_2 batteries, the authors employed activated carbon electrodes as both the reference and counter electrodes. However, the use of activated carbon as a reference electrode is not appropriate, as it does not provide a stable or well-defined potential. This compromises the accuracy of the electrochemical measurements. A standard three-electrode configuration can be used, incorporating a reference electrode with a known and stable potential.

Response: We sincerely thank the reviewer for pointing out the issue regarding the reference electrode. According to the comment, a standard three-electrode configuration was employed to accurately assess the potential of the KA-PA-Nuc electrolyte in four-electron aqueous Al-I_2 batteries. Specifically, an Ag/AgCl electrode was used as the reference electrode, an over-capacity AC electrode served as the counter electrode, and an iodine-AC electrode functioned as the working electrode. As shown in the Fig. R38, the iodine cathode shows two distinct and reversible redox peaks: one at approximately 0.3 V vs. Ag/AgCl, corresponding to the I^-/I^0 couple, and another at around 0.8 V vs. Ag/AgCl, attributed to the I^0/I^+ conversion reaction. These results confirm the effectiveness of the KA-PA-Nuc electrolyte in facilitating reversible four-electron iodine conversion reactions in aqueous Al-I_2 batteries. Accordingly, all relevant electrochemical data have been updated to ensure the accuracy and reliability of the measurements.

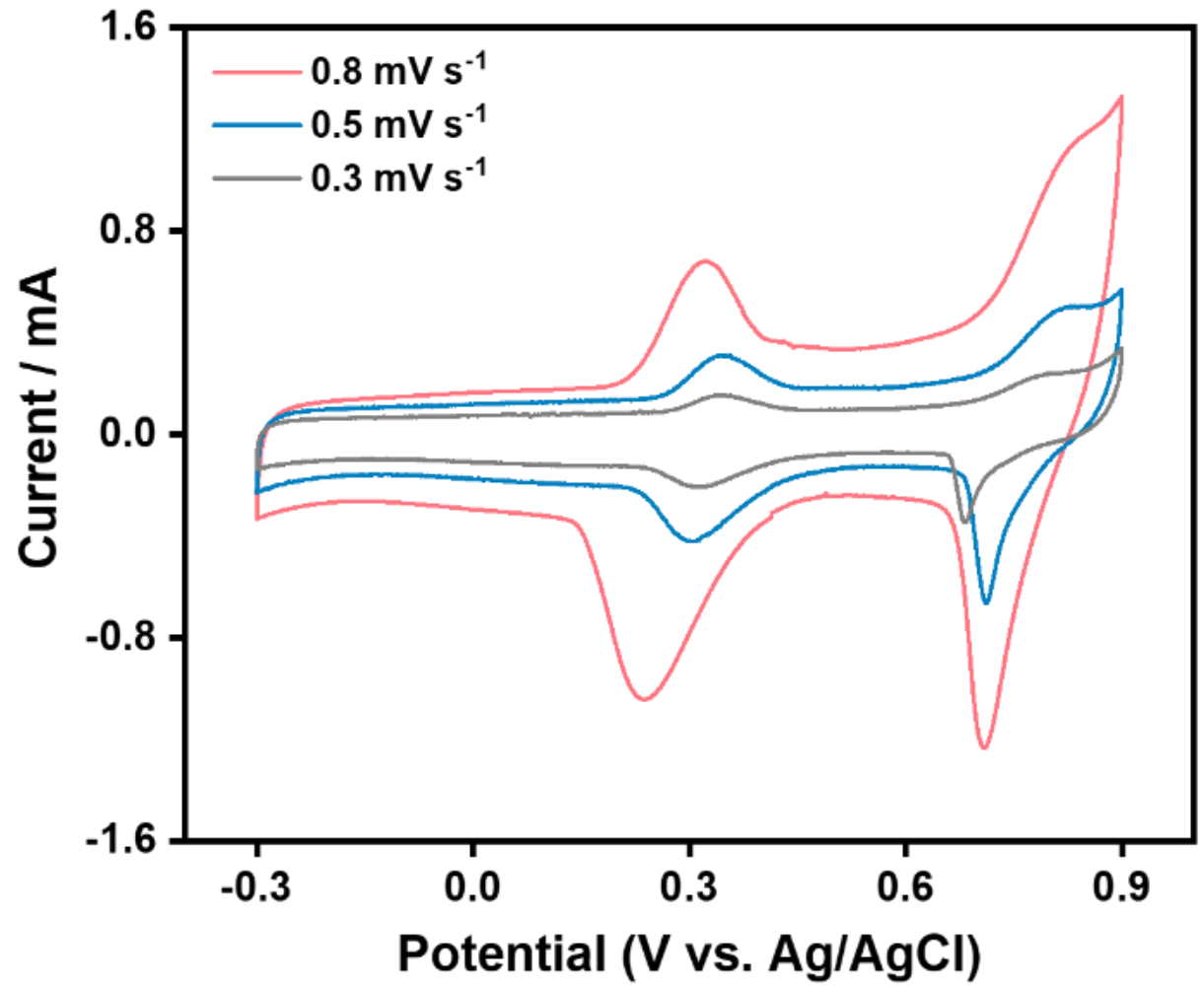

Fig. R38 CV profiles of aqueous Al-I_2 batteries in the KA-PA-Nuc electrolyte at various scan rates.

Manuscript Title: Low-Concentration and Non-Halogen Aqueous Electrolytes to Achieve Reversible Four-Electron Iodine Conversion Reactions

Manuscript number: 2025-04-08465B

Responses to referees

Referee #2

The authors have adequately addressed the comments by this reviewer and significantly improved the manuscript. I recommend to accept this work at its present form.

Response: We sincerely thank the reviewer for the positive evaluation of our work and for recommending its acceptance. We are particularly grateful for the reviewer's time, effort, and careful assessment throughout the review process. The insightful and constructive comments and suggestions have substantially improved the scientific rigor, clarity, mechanistic interpretation, and overall quality of this manuscript. We greatly appreciate the reviewer's valuable contributions to strengthening this work.

Referee #3

The authors' response generally meets my expectations. But the paper still has some issues to improve its quality:

Response: We sincerely thank the reviewer for the positive evaluation of previous responses and for the continued careful assessment of our work. We greatly appreciate the reviewer's time, effort, and thoughtful feedback throughout the review process. The reviewer's insightful and constructive comments have greatly helped us further improve the scientific quality, clarity, and overall presentation of the manuscript. In response to the reviewer's additional comments, we have carefully addressed each point in detail and revised the manuscript accordingly.

Comment 1. A lot of grammatical and typographical errors, such as (even on the first few pages)

industry grids should be industrial grid, and electronic devices call for should be and electronic devices calls for determined by the number of electron transfer should be determined by the number of electrons transferred

Giving that the competition between halogen should be Given that the competition between halogen while, anion-specific effect, should be while, anion-specific effects

Many others, the authors should carefully check.

Response: We sincerely thank the reviewer for the careful reading of our manuscript and for pointing out these grammatical and typographical issues. We greatly appreciate these helpful comments, which have contributed to improving the overall quality and clarity of the manuscript. In response, we have carefully proofread the entire manuscript and comprehensively corrected the grammatical, typographical, and language inconsistencies throughout the text, including all examples highlighted by the referee. In addition, we have undertaken a thorough line-by-line revision to further improve readability, precision, and overall presentation.

Comment 2. Another issue is that since the I-I+ reactions have been realized in halogen electrolytes, the high-voltage plateau contribution to the overall capacity should be compared with that of halogen electrolytes.

Response: We fully agree that, since the I^0/I^+ reaction has previously been realized in halogen-containing electrolytes, a direct comparison of the high-voltage plateau contribution to the overall capacity is important for evaluating the effectiveness of our proposed electrolyte design paradigm. In response, we have revised Supplementary Table 2 to additionally include a systematic comparison of the high-voltage plateau contribution reported for representative halogen-containing and high-concentration electrolyte systems in the literature. Based on this expanded comparison, it is evident that our proposed low-concentration, halogen-free electrolyte framework achieves a

higher contribution from the high-voltage plateau than many previously reported halogen-based systems.

These results further validate the effectiveness of the KA–PA–Nuc design paradigm in promoting reversible I^0/I^+ conversion, while avoiding the drawbacks associated with highly concentrated and halogen-containing electrolytes. Relevant data have been added to the revised Supplementary Information, which are presented as follows:

Supplementary Table 2 Comparison of electrochemical performances in various electrolytes for multielectron iodine redox reactions

Number	Electrolyte	Current density (A/g)/specific capacity (mAh/g)/ cycle number/capacity retention (%)/capacity decay per cycle (%)	I^0/I^+ capacity contribution (%)	Battery chemistry	Ref.
1	$2ZnCl_2$-1KCl	0.5/207/--/--<br>3/160/2800/80/0.0072	52.0<br>--	Zn-I_2	Energy Environ. Sci., 2021, 14, 407--413[1]
2	$5.9ZnCl_2$	0.24/422/100/95/0.05	~41.7	Zn-I_2	Journal of Power Sources, 580, 2023, 233296[2]
3	$30ZnCl_2$	2/470/2000/95.7/0.0022	~32.6	Zn-I_2	Nature Communications, 2023, 14:1856[3]
4	$20ZnCl_2$-1methyl ammonium iodide	--/--/--/--/--	--	Zn-I_2	Nanoscale, 2024, 16, 6596–6602[4]
5	4Znotf-6urea	1.3/219/700/91.3/0.0125<br>2.2/285/10000/51.6/0.0049	~35.1<br>~31.6	Zn-I_2	https://doi.org/10.1021/acsnano. 4c14451[5]
6	$19ZnCl_2$-19LiCl-8Acetonitrile	1/398/500/56/0.088	~41.5	Zn-I_2	Nano-Micro Lett., 2023, 15:126[6]
7	$7.5ZnCl_2$	1/405/250/63.2/0.1472<br>5/300/5000/60.1/0.008	~26.2<br>~21.3	Zn-I_2	Adv. Mater. 2024, 36, 2312246[7]
8	$15ZnCl_2$-1TEACl	0.5/425/200/86.9/0.0655<br>10/300/35000/47.5/0.0015	~46.5<br>~38.7	Zn-I_2	J. Am. Chem. Soc. 2024, 146, 21377−21388[8]
9	$10ZnCl_2$	0.5/422/50/50/1	--	Zn-I_2	J. Am. Chem. Soc. 2024, 146, 25108−25117[9]
10	15LiCl-$7LiNO_3$	1/243/300/86/0.047	--	S-I_2	Adv. Funct. Mater. 2023, 33, 2212644[10]
11	$2ZnCl_2$-16Liotf	1/425/1000/98.8/0.0012<br>2/400/10000/83.6/0.0017	~45.0<br>~36.8	Zn-I_2	Chem. Commun., 2024, 60, 7447[11]
12	$2ZnCl_2$-70%PEG	0.4/524.7/1500/97.6/0.0016<br>2/507.9/20000/81.5/0.00093	~47.6<br>~43.3	Zn-I_2	Adv. Mater. 2024, 2405473[12]
13	$19ZnCl_2$-19LiCl-	0.8/600/1000/85.4/0.0146	~42.4	Zn-I_2	Nature Communications,

	8Acetonitrile	2/500/6000/84.0/0.0027	--		2021, 12:170[13]
14	$3ZnSO_4$-$1ZnCl_2$	2.1/405/1200/75.0/0.0208	~39.5	Zn-I_2	Chem. Sci., 2024, 15, 3357[14]
15	$10ZnCl_2$-8Acetonitrile	1/490.9/1000/93.6/0.0064<br>2/470.5/10000/80.0/0.002	~43.1<br>~42.1	Zn-I_2	Science Bulletin 69 (2024) 1674 – 1685[15]
16	DMSO – $Zn(ClO_4)_2.6H_2O$ – NA	2/380/2000/80/0.01	~46.1<br>~42.6	Zn-I_2	Energy Environ. Sci., 2023, 16, 4502[16]
17	$3ZnSO_4$-3.5LiBr-$0.1LiNO_3$	--/635/500/67/0.066	--	Zn-I_2	ACS Nano 2022, 16, 20389−20399[17]
18	$1AlCl_3$-9LiTFSI	2/259/150/62.5/0.25	~38.6	Al-I_2	Small Methods 2021, 5, 2100611[18]
19	1.5KA-0.5PA-0.5Nuc	1/325/1000/83/0.017	39.4	Al-I_2	This work
	1.5KA-0.5PA-0.4Nuc	1/451/1000/96.0/0.004<br>2/407/2000/94.0/0.003	47.8<br>42.9	Zn-I_2	

Comment 3. When electrochemical impedance spectroscopy (EIS) measurements are presented in a complex impedance plot (i.e., the Nyquist plot), the graph must be ortho-normed (i.e., same scale on X and Y axes), with raw impedance data reported as symbols and fitted data as lines.

Response: We sincerely thank the reviewer for this valuable and technically important suggestion. In response, we have carefully revised all Nyquist plots throughout the manuscript accordingly. Specifically, the X and Y axes have been adjusted to the same scale for all relevant figures, and the raw impedance data are now presented as symbols, while the fitted results are shown as solid lines. These revisions have been implemented in the main text and Supplementary Information, including Figure 5b, Supplementary Fig. 40, Supplementary Fig. 42, Supplementary Fig. 44, Supplementary Fig. 46, and Supplementary Fig. 48. In addition, the corresponding fitting details have been further clarified in the Electrochemical measurements section.

Comment 4: The Al anode should be significantly more negative than Zn, but the charge-discharge profile of Al-I_2 is quite similar to that of Zn-I_2. Is the Al working, or is the Zn still working, since the Al-Zn alloy is used as an anode? What about the gas evolution of the Al-I_2 batteries?

Response: We sincerely thank the reviewer for raising this important and insightful

question. We agree that the working mechanism of the Al–I_2 batteries requires further clarification. Before addressing the specific concerns, we would like to first clarify three important points regarding the purpose and interpretation of the Al–I_2 experiments in this work.

First, the aqueous Al–I_2 batteries were introduced not as the central focus of this study, but rather as an additional model system to examine whether the proposed KA–PA–Nuc electrolyte design standard can be extended beyond Zn–I_2 chemistry to other aqueous metal–iodine systems. For this purpose, aqueous Al–I_2 batteries were assembled using Al–Zn alloy anodes following recently established strategies for aqueous Al-based batteries. In addition, the applicability of the KA–PA–Nuc electrolyte standard to aqueous Al–I_2 batteries is already supported by the three-electrode electrochemical measurements presented in this work. These results show that the KA–PA–Nuc electrolyte can activate and stabilize four-electron iodine conversion in the Al–I_2 configuration, confirming the generality of the KA–PA–Nuc standard toward multivalent metal–iodine systems.

Second, although Al metal is thermodynamically much more negative than Zn, practical aqueous Al batteries do not necessarily exhibit substantially higher full-cell voltages than aqueous Zn batteries. This is mainly because bare Al anodes suffer from severe passivation and competitive hydrogen evolution, leading to large polarization and poor reversibility. These limitations have been widely recognized challenges in aqueous Al battery systems. Therefore, instead of using bare Al foil, we employed an Al–Zn alloy anode, in which Al^{3+} is deposited onto and alloyed with Zn, enabling more reversible Al-related alloying/de-alloying chemistry while mitigating the severe polarization and gas evolution of bare Al. Corresponding working mechanisms and gas-evolution suppression effects have already been systematically validated in multiple recent studies through comprehensive characterizations including TEM/EDS, LSV, TOF-SIMS, XPS, ICP-OES, and synchrotron-based analyses (*Adv. Energy Mater., 2026, 0, e05262; Energy Environ. Sci., 2022, 15, 4572; Small, 2026, 0, e14994; J. Am. Chem. Soc. 2026, 148, 1220−1232; J. Am. Chem. Soc. 2020, 142, 15295−15304; Nat.*

Commun., 2025, 16, 6329).

Third, the primary focus of the present work is to establish the mechanistic role of the KA–PA–Nuc electrolyte standard in enabling reversible four-electron iodine conversion chemistry, rather than to comprehensively investigate aqueous Al anode chemistry itself. Therefore, we avoided overinterpreting the Al anode side and gas evolution behavior. Nevertheless, in our system, the electrochemical behaviors of Al–I_2 batteries are not identical to those of Zn–I_2 batteries, further indicating that the Al working mechanism. Furthermore, the excellent cycling stability of the Al–I_2 batteries in the KA–PA–Nuc electrolyte also indirectly suggests improved anode reversibility and mitigated side reactions/gas evolution under this electrolyte environment.

Following the reviewer's suggestion, we have added additional clarification and supporting references regarding the Al–Zn alloy anode chemistry in the revised manuscript. The corresponding statement added to the manuscript is as follows: "*Al–Zn alloy was selected as the anode owing to its improved stability and reversibility by taking advantage of reversible Al-related alloying/de-alloying chemistry.*[43,44]"

Referee #4

Having read the revised manuscript, the comments from three reviewers, and the rebuttal from the authors, I offer the following thoughts.

Overall, the authors have done an exceptionally thorough job in addressing the bulk of the comments from the reviewers, who, in turn, raised a comprehensive list of issues that certainly required attention. As a result, the revised manuscript is clearly, in my view, much stronger, particularly in providing more information on the key interactions between the three electrolyte species (KA, PA and Nuc). There are, though, aspects of the electrochemical behaviour that cast doubt on some of the claims made for this version of the iodine electrode.

Response: We sincerely thank the referee for the exceptionally careful evaluation of our revised manuscript and highly insightful and constructive comments. We greatly appreciate the referee's recognition that the revised manuscript has been substantially strengthened. More importantly, we are especially grateful to the referee for identifying several critical electrochemical and mechanistic issues that were insufficiently clarified in the previous revision. These comments have significantly helped us further improve the mechanistic depth of the manuscript.

In response to the reviewer's concerns, we have now systematically addressed all key issues through additional analyses, experiments, and expanded discussions in the revised manuscript. First, regarding the immobilization of $I^+(Nuc)_2$ species and their spatial distribution, in addition to the previously presented *in situ* UV–Vis results, we have now incorporated complementary Micro-CT analyses to provide more direct and intuitive evidence for the spatial distribution of iodine species. Combined with the spectroscopic and electrochemical analyses, these results further support the immobilization and stabilization of high-valent iodine intermediates within the cathode in the KA–PA–Nuc electrolyte. Moreover, we have expanded the discussion regarding the formation, migration, and electrochemical role of triiodide species, clarifying their relationship with the overall iodine conversion chemistry in different electrolytes.

Second, regarding the reviewer's question on whether the "water-deficient" interfacial environment is sufficiently deficient to suppress water-related side reactions, we have further clarified the underlying interfacial chemistry and mechanistic logic of the KA–PA–Nuc electrolyte design. Specifically, we now emphasize that the role of the KA–PA–Nuc standard is not to completely eliminate interfacial water owing to its low concentrations, but rather to reconstruct the interfacial microenvironment by regulating water and anion distributions, hydrogen-bonding networks, and halogen-bond stabilization. The polar anions enrich near the electrode interface and create water-deficient interfacial microenvironment, while the kosmotropic anions further reorganize the hydrogen-bonding network and increase the availability of nucleophilic species for halogen-bond stabilization. These effects collectively suppress water-

induced parasitic reactions and stabilize high-valent iodine intermediates, even though a certain amount of interfacial water remains present. By leveraging the synergistic interplay of interfacial chemistry, anionic chemistry, and halogen-bond stabilization, the KA–PA–Nuc standard enables reversible $I^-/I^0/I^+$ conversion chemistry in low-concentration and halogen-free aqueous electrolytes.

In addition, we have further clarified the origin of the unusually large capacity values and discussed the hydrogen bonding and bulk electrolytes properties. Most importantly, following the reviewer's comments regarding the low CE and capacity fading mechanism, we have now conducted a comprehensive and systematic investigation specifically aimed at clarifying the origin of low CE, the fundamental mechanism of capacity decay, and the intrinsic relationship between CE and capacity fading for Zn-I_2 batteries. Accordingly, the revised manuscript now includes: (i) revised CE-related plots with standardized axis scales; (ii) mechanistic analysis of the origin of low CE together with the establishment of distinct degradation models for I^-/I^0, I^0/I^+, and coupled $I^-/I^0/I^+$ conversion reactions; (iii) post-mortem characterizations of both low-loading and ultra-high-loading cells after long-term cycling; and (iv) systematic iodine inventory and redistribution analyses for cells with different iodine loadings. These results collectively reveal that the relatively low CE mainly originates from parasitic processes associated with both high-valent iodine species and triiodide intermediates. Importantly, the inconsistency between the cumulative CE loss and the actual capacity loss enables the redefinition of the capacity loss as reversible capacity loss and irreversible capacity loss for iodine conversion reactions due to the redistribution of iodine species and chemical reactions among different intermediates. Furthermore, we demonstrate that the long-term capacity fading behavior under high-loading conditions is intrinsically linked to a more pronounced triiodide-mediated capacity degradation pathway. Finally, after clarifying these key scientific mechanisms, we further discuss potential strategies and future perspectives for further improving the reversibility and stability of four-electron iodine conversion chemistry, particularly from the synergistic viewpoint of stabilization of I^+ species while suppression of

triiodide migration. We hope that these additional experiments, analyses, and expanded discussions can fully address the reviewer's concerns and meet the high standards expected for publication.

Comment 1: Comment 6 from R2 questions the evidence provided for the immobilization of $I^+(Nuc)_2$ at, or close to, the electrode surface once it is formed. The response, based on UV-vis monitoring of triiodide does not really answer the question posed. The formation and migration of triiodide will affect the characteristics of the first redox process but as long as it is stable in the bulk solution, there is no reason for it not to be eventually consumed in the first oxidation wave (charge capacity data seem to support this).

Response: To address the reviewer's key concern regarding the distribution of high-valent iodine species, we have conducted additional analyses and mechanistic discussion to further clarify and correlate the spatial localization, electrochemical behavior, and interfacial environment of iodine species in different electrolytes.

Beyond the previously presented *in situ* UV–Vis results, we have now incorporated complementary Micro-CT characterization performed at the fully charged state (1.8 V vs. Zn^{2+}/Zn) to directly visualize the spatial distribution of iodine species. The Micro-CT results provide intuitive spatial evidence that high-valent iodine species are confined within the cathode region in the electrolyte containing CH_3COO^-, with no obvious diffusion into the bulk electrolyte (Fig R1c). In the electrolyte containing $CF_3SO_3^-$, partial iodine species are observed in bulk electrolyte and near the anode side, indicating moderate iodine species diffusion (Fig R1a). In sharp contrast, a large amount of iodine species is observed in the bulk electrolyte and near the anode side in the electrolyte containing CF_3COO^-, suggesting a highly irreversible iodine conversion process accompanied by extensive migration of iodine species (Fig R1b). These observations are fully consistent with the *in situ* UV–Vis results. Collectively, the Micro-CT and *in situ* UV–Vis analyses strongly support the immobilization and

stabilization of high-valent iodine species within the cathode under the anion-enriched, water-deficient microenvironment established by the KA–PA–Nuc electrolyte standard.

As for the reviewer's concern regarding the role of triiodide species in the redox process, we have now conducted a comprehensive mechanistic investigation (please refer to the response to comment 3 for detailed discussion). These newly added results reveal that the formation and migration of triiodide species are intrinsically linked to the degradation mechanism of iodine conversion reactions. Specifically, for the I^-/I^0 conversion reaction, although triiodide species may partially participate in subsequent electrochemical reactions (corresponding to reversible loss), not all triiodide species can reversibly return to the electrode and be fully consumed by the end of first oxidation process, resulting in irreversible loss (Fig R3). Similar accumulated triiodide signals during cycling have also been observed in several recent studies employing iodine conversion chemistry (*J. Am. Chem. Soc., 2025, 147, 16350–16361; Angew. Chem. Int. Ed., 2025, DOI: 10.1002/anie.202510737; Energy Environ. Sci., 2025, 18, 4800–4810; Angew. Chem. Int. Ed., 2025, DOI: 10.1002/anie.202506006; Angew. Chem., 2025, DOI: 10.1002/ange.202525573; J. Am. Chem. Soc., 2024, 146, 6628–6637; Joule, 2025, 9, 7*). However, this phenomenon has generally been implicitly assumed or overlooked, and its direct correlation with irreversible iodine loss and electrochemical behavior has not been systematically discussed. Importantly, this irreversible loss becomes more pronounced within the water-enriched interfacial environments. Once triiodide species diffuse away from the confined interfacial region, they become increasingly susceptible to redistribution, shuttle-mediated migration, and parasitic side reactions, ultimately leading to irreversible loss. This mechanism explains the gradual capacity fading and moderate iodine-species diffusion observed in the electrolyte containing $CF_3SO_3^-$, even though only the I^-/I^0 conversion reaction is activated (Fig. 3c).

In contrast, for the coupled $I^-/I^0/I^+$ conversion reactions, the stability of high-valent iodine species becomes even more strongly dependent on the interfacial environment. Under water-enriched interfacial environment, high-valent iodine species exhibit substantially reduced stability and become more vulnerable to hydrolysis, producing

IO_3^- intermediates. These migrated intermediates can further undergo chemical reactions and ultimately generate additional triiodide species. Such processes not only destabilize I^+ species, but also accelerate the formation, migration, and redistribution of triiodide species, thereby aggravating shuttle effects and irreversible iodine loss. This mechanism explains the rapid capacity fading and extensive iodine species diffusion observed in the electrolyte containing $CF_3COO^–$ under highly irreversible I^0/I^+ conversion conditions (Fig. 3c). Remarkably, the electrolyte containing CH_3COO^- forms an anion-enriched, water-deficient interfacial environment that not only effectively confines and stabilizes I^+ species, but also suppresses triiodide migration and redistribution, thereby enabling a highly reversible four-electron iodine conversion reaction (Fig. 3c and Supplementary Fig. 28).

Collectively, these results clearly demonstrate that the spatial distribution and electrochemical behavior of iodine redox species are strongly governed by the interfacial microenvironment determined by the anion type, providing a much more comprehensive understanding of the relationships among spatial localization, electrochemical behavior, interfacial environment, and degradation mechanisms during four-electron iodine conversion reactions. Therefore, corresponding data and clarification have been incorporated into the revised manuscript: "*In situ UV–Vis spectra and Micro-CT, revealing the spatial distribution and reversibility of iodine redox species, are fully consistent with the electrochemical trends, highlighting the pivotal role of the anion-enriched, water-deficient interface created by the strongly polar anion in enabling reversible four-electron iodine conversion reactions (Supplementary Fig. 28-29)*"

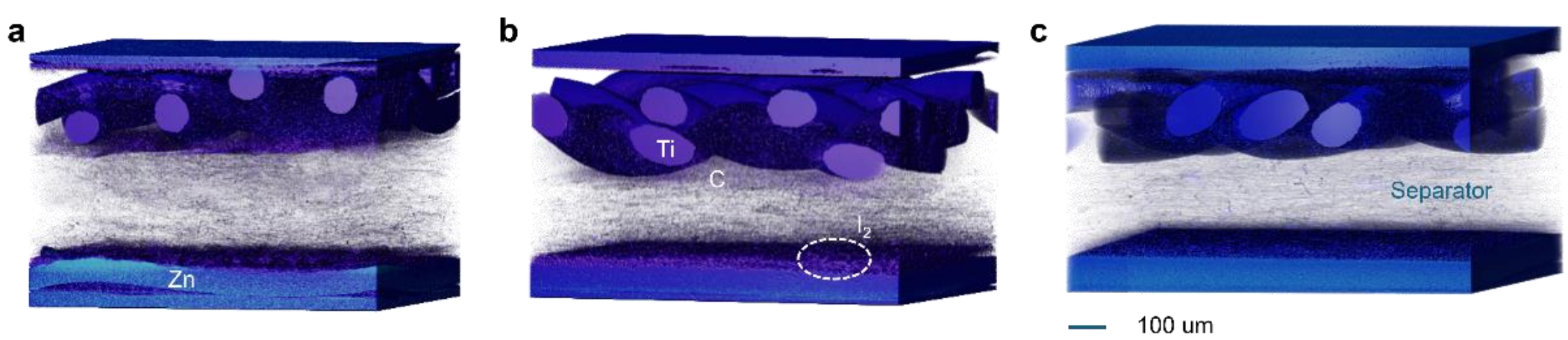

Fig. R1 Characterization of the spatial distribution of iodine species. a, X-ray micro-computed tomography (Micro-CT) volume rendering of the charged Zn-I_2 battery (1.8 V) in the electrolyte with $CF_3SO_3^-$. b, Micro-CT volume rendering of the charged Zn-I_2 battery (1.8 V) in the electrolyte with CF_3COO^-. c, Micro-CT volume rendering of the charged Zn-I_2 battery (1.8 V) in the electrolyte with CH_3COO^-.

Comment 2: Later in their response to this comment, the authors mention their ATR-IR results, shown in Fig.3g-i. For the most polar anion (acetate, Fig 3i), there is certainly a lower concentration of water present. But the concentration is still appreciable. The designation 'deficient' is accurate, but is it 'deficient enough' to prevent side reactions related to water?

Response: We sincerely thank the reviewer for this insightful and scientifically important comment. We fully agree that the term "water-deficient" should not imply the complete absence of interfacial water, particularly in low-concentration aqueous electrolytes. The reviewer's question regarding whether the interfacial environment is "deficient enough" to suppress water-related side reactions is therefore highly important for clarifying both the mechanism and the practical significance of the KA–PA–Nuc electrolyte design standard.

First, we would like to emphasize that the central concept of the KA–PA–Nuc standard is not to completely eliminate water from the electrolyte-electrode interface. Such complete exclusion of water is intrinsically difficult in aqueous systems, even under highly concentrated electrolyte conditions. Instead, the key mechanism of our strategy is to reconstruct the interfacial microenvironment through the synergistic interplay of kosmotropic anions (KA), polar anions (PA), and nucleophilic species (Nuc), thereby sufficiently suppressing water-related parasitic reactions while enabling and stabilizing the otherwise inaccessible four-electron iodine conversion chemistry under low-concentration, halogen-free conditions. Specifically, the strongly polar anion (CH_3COO^-) preferentially accumulates at the electrode surface, facilitating an anion-

enriched and water-deficient interfacial environment. Meanwhile, the kosmotropic anion (SO_4^{2-}) further reorganizes the hydrogen-bonding network of water and weakens the interaction between water molecules and nucleophilic species, thereby increasing the availability of nucleophilic species for halogen-bond stabilization. Therefore, the stabilization mechanism does not arise from a single anion effect, but rather from the synergistic cooperation between the KA and PA in regulating interfacial mass distribution, hydrogen-bonding structure, and halogen-bond stabilization chemistry.

Importantly, although interfacial water is not completely eliminated in our system, it is still "deficient enough" to sufficiently prevent water-related parasitic reactions. This is evidenced by the markedly improved electrochemical reversibility and cycling stability, as well as the significantly reduced interfacial water density even compared with high-concentration, halogen-containing electrolytes. In other words, the KA–PA–Nuc electrolyte does not completely eliminate interfacial water, but it sufficiently suppresses water-related side reactions to render reversible four-electron iodine conversion chemistry in low-concentration, halogen-free aqueous electrolytes.

We believe this distinction is mechanistically important and practically significant. Accordingly, the key advance of the KA–PA–Nuc electrolyte standard is not the complete elimination of interfacial water, but rather the successful transformation of a previously inaccessible reaction chemistry into a reversible and stable process from a more practical and effective perspective through the synergistic regulation of interfacial chemistry, hydrogen-bonding structure, and halogen-bond stabilization chemistry in low concentration, halogen-free electrolytes.

Comment 3: Comment 3 from R3 notes that the CEs are low. I would also add that specific values of CE are nowhere to be found in the manuscript. All CE axis scales (with the exception of SI Fig 53) are drawn with '90%' as the highest marked value. This gives the impression of a metric to which the authors do not wish to draw attention. In response to R3's comment, the authors provide a commentary that mainly draws

comparisons between their best and worst electrolyte formulations – this does not explain the issue. But they also note the following: 'This phenomenon primarily arises from the intrinsic characteristics of high-valent iodine redox process, which inherently involve multi-step electron transfer and transient intermediate species. Similar behavior has been widely reported in previous studies employing high-concentration, halogen-containing electrolytes, reflecting the inherent complexity and intrinsic instability of high-valent iodine redox chemistry'. I think this indicates that there are grounds for concern here. As is well-known, CE can be tied directly to battery cycleability. Exceptions occur with lead-acid and some nickel-based aqueous systems, where excess charge can feed a close-loop overcharge reaction that effectively maintains full capacity (other failure modes notwithstanding of course). In the present case though, one has to wonder about the effects of the 'instability' that is mentioned here.

I think that evidence of underlying instability becomes more obvious in the data provided for the higher loaded cells. SI Figs 54d,e show that capacity and CE plots become decidedly 'noisy' at the highest loading. Such behaviour, along with the increasing rate of capacity loss, is certainly consistent with the greater impact of effects related to instability. Another indicator of background instability is in SI Fig 51b (GCD performance at variable rates). Specifically, the apparent inverse relationship between rate and CE, where CE only approaches 100% at the highest rates, thereby indicating that side reactions are present.

At this point, my impression is that the authors have buttressed the chemical evidence supporting their claims as much as possible, in line with the comments made by the reviewers. They have not, though, addressed the issue of low CE, and nor have they sought to probe the reason(s) for the cells losing capacity. These two points are undoubtedly linked, so that a proper post mortem analysis could well have provided more insight into the behaviour of this Zn-I_2 system. As it stands, though, samples from the high- and ultra-high loading cells were, according to SI Figs 55-57, only taken up to the 80-cycle mark, by which point none of the cells had undergone any capacity loss. As presented, this analysis merely tells the reader that there are no indicators of

significant change in the electrodes or separators, after a relatively insignificant number of cycles. And yet, these cells do fail, as seen in SI Fig 54, where almost 30% loss is noted for the 12.2 mg/cm^2 cell. It is at this point where a much fuller understanding of cell behaviour would be obtained, through examination of electrodes, separators and electrolyte samples. Analyses should include a full iodine inventory, cast against the initial balance of active materials so that a true measure of the performance of this system can be established.

My concluding view is that this study cannot be considered suitable for publication until a thorough investigation is conducted into the low CE and how this is related to the underlying mechanism of capacity loss.

Response: We sincerely thank the reviewer for this highly insightful and constructive comment. We fully agree that coulombic efficiency (CE) is a key metric for assessing the reversibility and cycleability of batteries. We also agree that our previous revision did not sufficiently clarify the origin of relatively low CE, the fundamental mechanism of capacity decay, and the intrinsic relationship between CE and capacity fading. In response, we have conducted comprehensive and systematic mechanistic investigations to clarify these points.

First, we have revised all CE-related figures in the manuscript and Supplementary Information. The CE axis scales have been redrawn to provide clearer and more transparent presentation of the CE values. More importantly, to uncover the origin of the relatively low CE and capacity decay, we decoupled the four-electron iodine conversion chemistry into three representative processes: the decoupled I^0/I^+ conversion reaction, the decoupled I^-/I^0 conversion reaction, and the coupled $I^-/I^0/I^+$ conversion reactions. Their electrochemical behaviors and degradation mechanisms were then systematically analyzed.

For the decoupled I^0/I^+ conversion reaction, this reaction shows excellent cycling stability but a relatively low CE of approximately 94% (Fig. R2a). The corresponding GCD profiles show that the discharge capacity is lower than the charge capacity,

consistent with the observed CE values (Fig. R2c). Conventionally, CE is commonly used to estimate battery longevity according to:

$$Capacity\ \ retention = \prod_{n=1}^{cycle\ \ n} CE_{cycle\ \ n} \qquad (1)$$

However, comparison between the measured capacity retention and the capacity retention calculated from cumulative CE values reveals a substantial discrepancy (Fig. R2b). Under the testing conditions, where excess electrolyte and Zn anode were used, the dominant source of capacity decay can reasonably be attributed to iodine inventory loss from the cathode. The apparent discrepancy between calculated and measured capacity retention indicates that not all CE loss contributes to irreversible capacity fading. In other words, part of the iodine inventory loss is reversible. To further clarify this mechanism, we performed extensive *in situ* and post-mortem analyses of the I^0/I^+ conversion process. *In situ* Raman spectra reveal reversible conversion between I^+ and I^0 during cycling (Fig. R2d). *Ex situ* Raman spectra of cycled separators collected at different discharge states show negligible iodine-related signals and no visible iodine coloration on the separators, indicating no obvious iodine migration and supporting the excellent stability of this conversion process (Fig. R2e). Even after extending cycling to 700 cycles, only a weak IO_3^- signal was detected, without obvious I_2 or I_3^- features, suggesting that the irreversible capacity loss mainly originates from IO_3^--related side reactions. This conclusion is further supported by the Raman spectrum of extracted cathode solution at the charged state of 1.8 V, where characteristic signals of I^0, I^+, and IO_3^- are observed (Fig. R2e).

Mechanistically, within this voltage window, I_2 is electrochemically oxidized to I^+ during charging and reduced back to I_2 during discharge. However, I^+ is highly electrophilic and can undergo hydrolysis in aqueous electrolytes through nucleophilic attack by water molecules:

$$5I^+ + 3H_2O \rightarrow \ \ IO_3^- + 2I_2 + 6H^+ \qquad (2)$$

During charging, the I_2 generated by I^+ hydrolysis remains confined within the cathode

region and can be further oxidized, thereby contributing additional charge capacity. During discharge, however, hydrolysis of I^+ partially reduces the discharge capacity within that cycle. Together, these two processes result in relatively low CE. Importantly, the I_2 generated by I^+ hydrolysis during discharge remains confined within the cathode and can participate in subsequent electrochemical cycle. Therefore, this process represents reversible capacity loss rather than irreversible iodine inventory loss. By contrast, IO_3^- remains relatively stable within the applied voltage window and therefore causes irreversible iodine inventory loss. Based on these results, we propose an IO_3^--mediated degradation mechanism for the decoupled I^0/I^+ conversion reaction (Fig. R2f). This mechanism explains why the apparent CE is relatively low while the overall cycling stability remains excellent. It further suggests that, if excluding the extra charge capacity associated with hydrolysis generated I_2 during charging, the intrinsic reversibility of the I^0/I^+ process is higher than the apparent CE implies.

We next investigated the decoupled I^-/I^0 conversion reaction. This reaction shows good cycling stability and a higher CE of 98–99% (Fig. R3a, c). Nevertheless, the calculated capacity retention based on CE still deviates from the measured capacity retention (Fig. R3b), again indicating that not all CE loss directly translates into irreversible capacity fading. *In situ* Raman spectra confirm the generation of I_3^- during the I^-/I^0 conversion process (Fig. R3d). *Ex situ* Raman spectra of separators after prolonged cycling reveal clear I_3^- signals, indicating migration of dissolved I_3^- species into the separator region. At the end of charging, the I_3^- signals become substantially weaker but do not completely disappear, suggesting that most dissolved I_3^- species can return and participate in subsequent electrochemical cycling, whereas a small fraction remains unrecovered and contributes to irreversible iodine inventory loss (Fig. R3e). Accordingly, we propose an I_3^--mediated degradation mechanism for the decoupled I^-/I^0 conversion reaction (Fig. R3f). In this mechanism, I_3^- formation and dissolution during discharge cause partial discharge capacity loss and prevent CE from reaching 100%, while the recovery of most dissolved I_3^- during subsequent charging accounts for the reversible capacity loss.

Building on these analyses, we further investigated the coupled $I^-/I^0/I^+$ conversion reactions. Similar to the decoupled systems, the coupled reactions also exhibit a clear discrepancy between measured capacity retention and calculated capacity retention (Fig. R4a), confirming that not all apparent CE loss corresponds to irreversible degradation. Interestingly, in the coupled reactions, the discharge capacity of the I^-/I^0 plateau exceeds the corresponding charge capacity, indicating the existence of additional discharge capacity contribution (Fig. R4b, c, f). *Ex situ* XPS and Raman spectra confirm reversible $I^-/I^0/I^+$ conversion processes, whereas I_3^--related signals detected on cycled separators indicate that irreversible iodine inventory loss mainly arises from I_3^- migration (Fig. R4d, e). Importantly, in the coupled system, the presence of I^- and I_3^- fundamentally changes the fate of hydrolysis generated IO_3^-. Specifically, IO_3^- can chemically react with low-valence iodine species such as I^-:

$$IO_3^- + 5I^- + 6H^+ \rightarrow 3I_2 + 3H_2O \qquad (3)$$

The generated I_2 can subsequently participate in electrochemical discharge reactions, thereby contributing additional discharge capacity. Simultaneously, part of I^- can be chemically oxidized into I_2 and re-participate in the electrochemical process. These coupled chemical/electrochemical pathways explain why the discharge capacity of the I^-/I^0 plateau exceeds the corresponding charge capacity. Meanwhile, although some IO_3^- may migrate into the electrolyte at high potentials, it can rapidly react with migrated I^- or I_3^- to regenerate I_2 or I_3^-, explaining why IO_3^- is not detected on the separator. At lower potentials, the I_3^--mediated degradation pathway remains operative. Therefore, we propose a combined IO_3^-/I_3^--mediated degradation mechanism for the coupled $I^-/I^0/I^+$ conversion reactions (Fig. R4g).

This mechanism indicates that the relatively low CE observed in coupled four-electron iodine conversion chemistry arises from multiple coupled processes, including: (i) additional charge capacity associated with I^+ hydrolysis during charging, (ii) irreversible IO_3^--mediated iodine loss, and (iii) I_3^- dissolution/migration. Simultaneously, IO_3^--related chemical reactions and partial I_3^- recovery help maintain good cycling stability. These findings demonstrate that the apparent low CE does not

simply indicate direct irreversible failure but instead reflects a complex interplay between reversible and irreversible iodine inventory processes.

Critically, whether in the IO_3^--mediated degradation mechanism of the decoupled I^0/I^+ reaction, the I_3^--mediated degradation mechanism of the decoupled I^-/I^0 reaction, or the combined IO_3^-/I_3^--mediated degradation mechanism of the coupled four-electron reaction, the key prerequisite is that the generated iodine intermediates remain confined within the cathode or cathode/electrolyte interfacial region, so that they can be reutilized during subsequent electrochemical processes. Stabilization and immobilization of I^+ species are therefore fundamentally important. Without sufficient stabilization and immobilization of I^+ species, extensive hydrolysis would produce large quantities of IO_3^- and I_2, thereby accelerating iodine shuttling, redistribution, and irreversible iodine inventory loss. This is precisely the key role of the KA–PA–Nuc electrolyte standard, which not only confines and stabilizes I^+ species, but also suppresses iodine species migration and redistribution through the synergistic interplay of anion-specific interfacial chemistry and halogen-bond stabilization within an anion-enriched, water-deficient interfacial environment.

We further compared the capacity fading behavior and degradation mechanism under different mass loadings. For the coupled $I^-/I^0/I^+$ conversion reactions at different mass loadings, all systems exhibit discrepancies between measured and calculated capacity retention, together with larger discharge capacities than charge capacities for the I^-/I^0 plateau, indicating that they follow the same combined IO_3^-/I_3^--mediated degradation mechanism (Fig. R5a–c). Decoupled analyses further show that the I^0/I^+ conversion reaction exhibits excellent cycling stability across different mass loadings, suggesting comparable IO_3^--mediated inventory loss (Fig. R5d). In contrast, the decoupled I^-/I^0 conversion reaction shows faster capacity decay at high mass loading, indicating more severe I_3^--mediated iodine migration and redistribution (Fig. R5e). To directly visualize this process, we performed X-ray Micro-CT imaging of cycled Zn–I_2 batteries with different iodine loadings. The Micro-CT results provide direct spatial evidence of iodine migration and redistribution. At low mass loading, no obvious iodine

migration toward the bulk electrolyte or anode side is observed after 200 cycles, and only limited iodine accumulation appears even after 500 cycles (Fig. R5f, g). In sharp contrast, at high mass loading, obvious iodine migration into the bulk electrolyte and toward the anode side is observed after 200 cycles, and substantial iodine accumulation appears after 500 cycles (Fig. R5h, i). These observations directly confirm that accelerated capacity fading and noisier CE profiles under high loading conditions originate predominantly from intensified I_3^--mediated migration and redistribution pathways.

Additional post-mortem analyses after prolonged cycling were also conducted according to the reviewer's suggestion. SEM images show that iodine cathodes at different mass loadings maintain good structural integrity even after prolonged cycling, indicating that cathode structural evolution has negligible contribution to the observed electrochemical degradation and capacity fading (Fig. R6-7). XRD analyses of cycled Zn anodes reveal an additional diffraction peak at approximately 7.5°, assigned to $Zn_4SO_4(OH)_6 \cdot xH_2O$, a common interfacial byproduct in $ZnSO_4$-containing electrolytes. Compared with low mass loading Zn-I_2 batteries, the Zn anodes in high mass loading Zn-I_2 batteries show progressively stronger $Zn_4SO_4(OH)_6 \cdot xH_2O$ diffraction peaks with increasing cycling numbers. Consistently, SEM images also reveal rougher Zn surfaces under high mass loading conditions (Fig. R8-9). These behaviors originate from two coupled factors. First, compared with low loading systems, high loading Zn–I_2 batteries involve larger areal capacities during cycling, thereby amplifying Zn anode parasitic reactions. Second, under high loading conditions, more severe I_3^- migration and redistribution further accelerate Zn anode corrosion to a certain extent. This interpretation is fully consistent with the electrochemical results and the Micro-CT visualization of iodine redistribution. In addition, the XRD patterns of various iodine cathodes collected after different cycling stages and mass loadings remain nearly identical, and the FTIR spectra of electrolyte-containing separators after different cycling stages and mass loadings also show no obvious differences. These characterizations therefore do not provide additional mechanistically meaningful

information regarding the degradation process (Fig. R10). Accordingly, to avoid unnecessary redundancy of the revised manuscript, these less informative datasets were removed from the current version. Nevertheless, the comprehensive post-mortem analyses presented in this work including Micro-CT imaging, XPS characterization, Raman spectroscopy, and the systematic mechanistic investigations collectively provide sufficient and compelling evidence to elucidate the origin of low CE and the fundamental mechanism of capacity decay.

Overall, these newly added mechanistic investigations and post-mortem analyses demonstrate that: (i) the relatively low CE in four-electron iodine conversion chemistry does not simply originate from irreversible loss, but rather from coupled reversible and irreversible iodine inventory processes; (ii) capacity fading mainly arises from parasitic reactions involving both high-valent iodine species and triiodide intermediates; (iii) immobilization and stabilization of iodine intermediates within the cathode or interfacial region are fundamentally critical for achieving reversibility and stability; (iv) the critical role of the KA–PA–Nuc electrolyte standard in immobilizing and stabilizing iodine intermediates within anion-enriched, water-deficient interfacial environment through the synergistic interplay of anion-specific interfacial chemistry and halogen-bond stabilization. Importantly, although the KA–PA–Nuc electrolyte standard cannot completely eliminate these parasitic reactions, it can substantially mitigate them, thereby enabling highly reversible and stable four-electron iodine conversion chemistry even in low-concentration, halogen-free aqueous electrolytes.

These findings also provide several important directions for future development of four-electron iodine conversion chemistry. First, future studies should move beyond simply activating and stabilizing I^+ species and instead establish comprehensive iodine inventory management frameworks that effectively track the generation, migration, redistribution, and consumption of iodine intermediates. Correspondingly, more advanced operando and spatially resolved characterization techniques will become increasingly important for directly recognizing and tracking different iodine intermediates; Second, future efforts should simultaneously improve the stability of

both the I^0/I^+ and I^-/I^0 conversion reactions, rather than focusing solely on high-valent iodine activation and stabilization. In this context, further strategies for enhancing the immobilization or stabilization of iodine intermediates, including I^+, IO_3^-, and I_3^- species, will be critical for minimizing irreversible iodine inventory loss, especially under practically high mass loading conditions; Third, strategies for effectively modulating interfacial microenvironment in low-concentration, halogen-free aqueous systems. Therefore, the development of four-electron iodine conversion chemistry is far from simply activating and stabilizing I^+ species. More broadly, this work suggests that future advances in high-valent halogen electrochemistry may require moving beyond conventional bulk solvation design toward more effective and practical interfacial chemistry and interfacial microenvironment engineering strategies. We believe that the mechanistic insights established in this work not only clarify the origin of low CE and capacity fading in four-electron iodine conversion reactions, but also provide an important foundation for the future design of highly reversible, practical, and scalable high-valent aqueous halogen batteries. Corresponding data and discussion about post-mortem analyses and mechanistic investigations have been added into the revised manuscript and Supporting Information: "*Comprehensive post-mortem analyses and systematic mechanistic investigations further reveal that the relatively low Coulombic efficiency originates from coupled reversible and irreversible iodine inventory processes associated with I^+ hydrolysis and I_3^- migration. Importantly, the KA–PA–Nuc electrolyte effectively mitigates these parasitic pathways by stabilizing and confining iodine intermediates within the anion-enriched, water-deficient interfacial microenvironment (Supplementary Fig. 56-64)*".

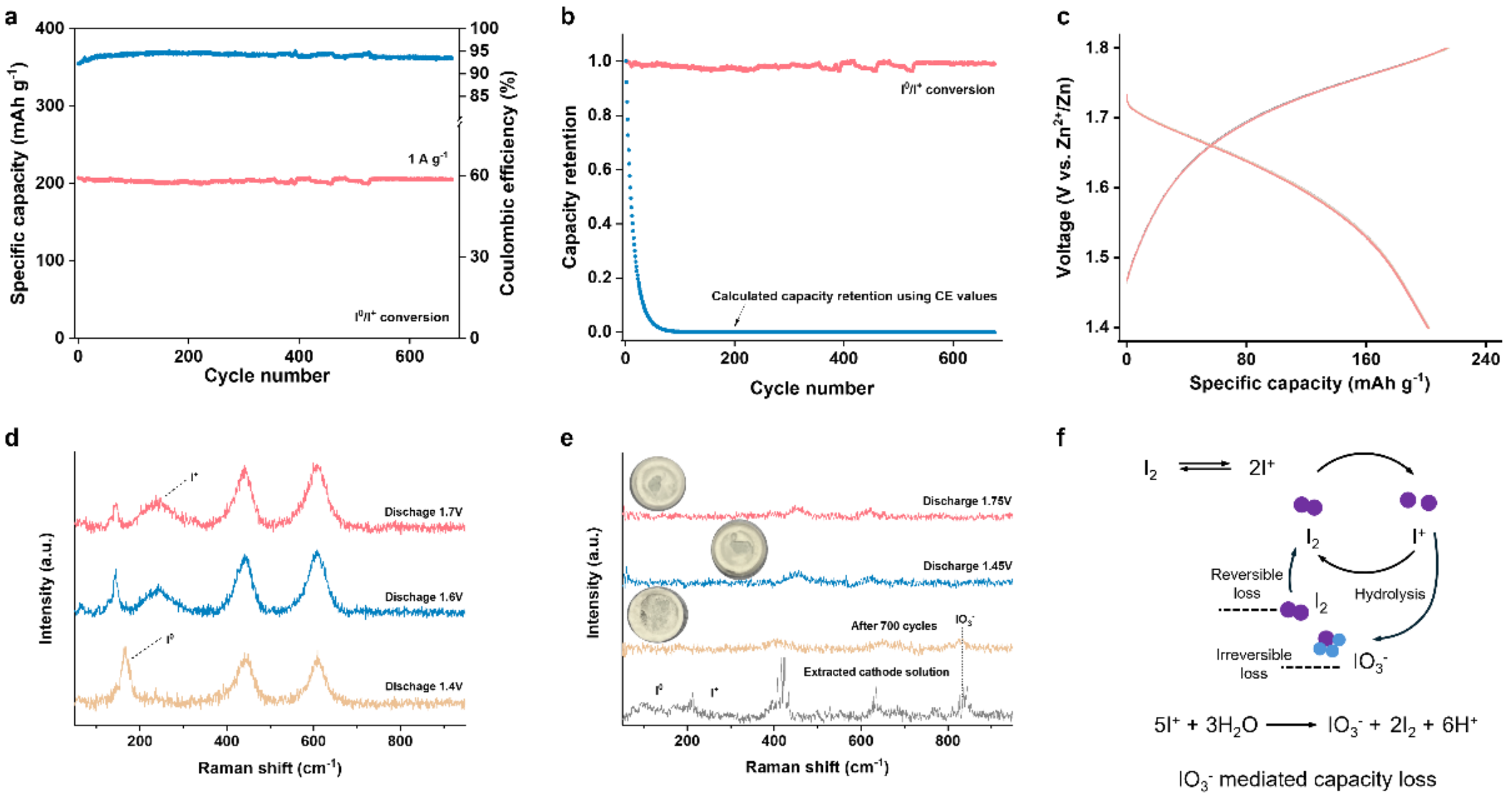

Fig. R2 Mechanistic analysis of the decoupled I^0/I^+ conversion reaction in the KA-PA-Nuc electrolyte. a, Cycling performance of the I^0/I^+ conversion reaction at 1 A g^{-1}. b, Comparison between the measured capacity retention and the calculated capacity retention from CE values of the I^0/I^+ conversion reaction. c, GCD profiles of the I^0/I^+ conversion reaction. d, *In situ* Raman spectra of the iodine cathode at different discharge states. e, *ex situ* Raman spectra of the cycled separators and extracted cathode solution; inset images show the corresponding photographs of the separators. f, Schematic illustration of the conversion processes and degradation mechanism of the I^0/I^+ conversion reaction.

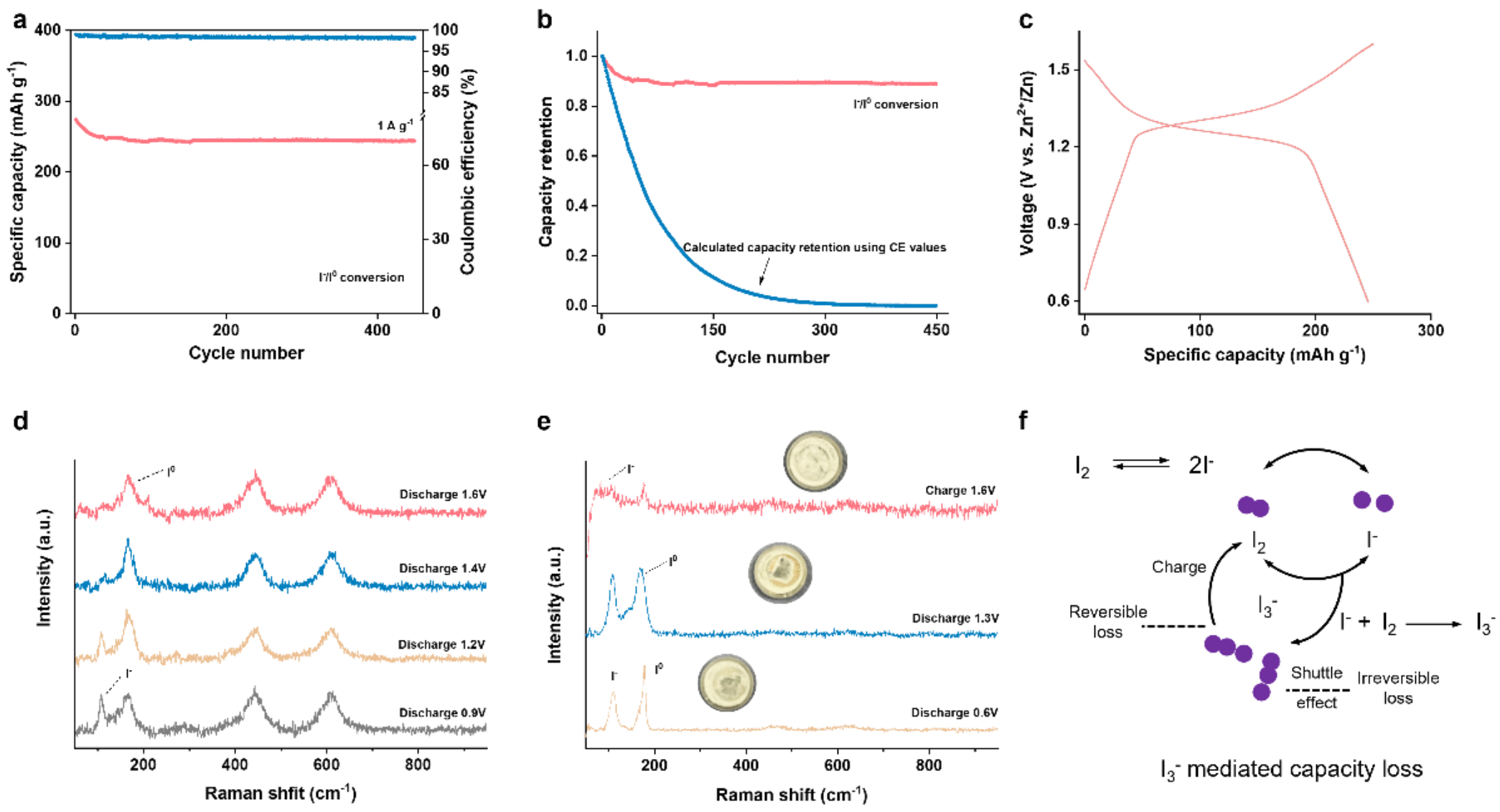

Fig. R3 Mechanistic analysis of the decoupled I^-/I^0 conversion reaction in the KA-PA-Nuc electrolyte. a, Cycling performance of the I^-/I^0 conversion reaction at 1 A g^{-1}. b, Comparison between the measured capacity retention and the calculated capacity retention from CE values of the I^-/I^0 conversion reaction. c, GCD profiles of the I^-/I^0 conversion reaction. d, *In situ* Raman spectra of the iodine cathode at different discharge states. e, *ex situ* Raman spectra of the cycled separators; inset images show the corresponding photographs of the separators. f, Schematic illustration of the conversion processes and degradation mechanism of the I^-/I^0 conversion reaction.

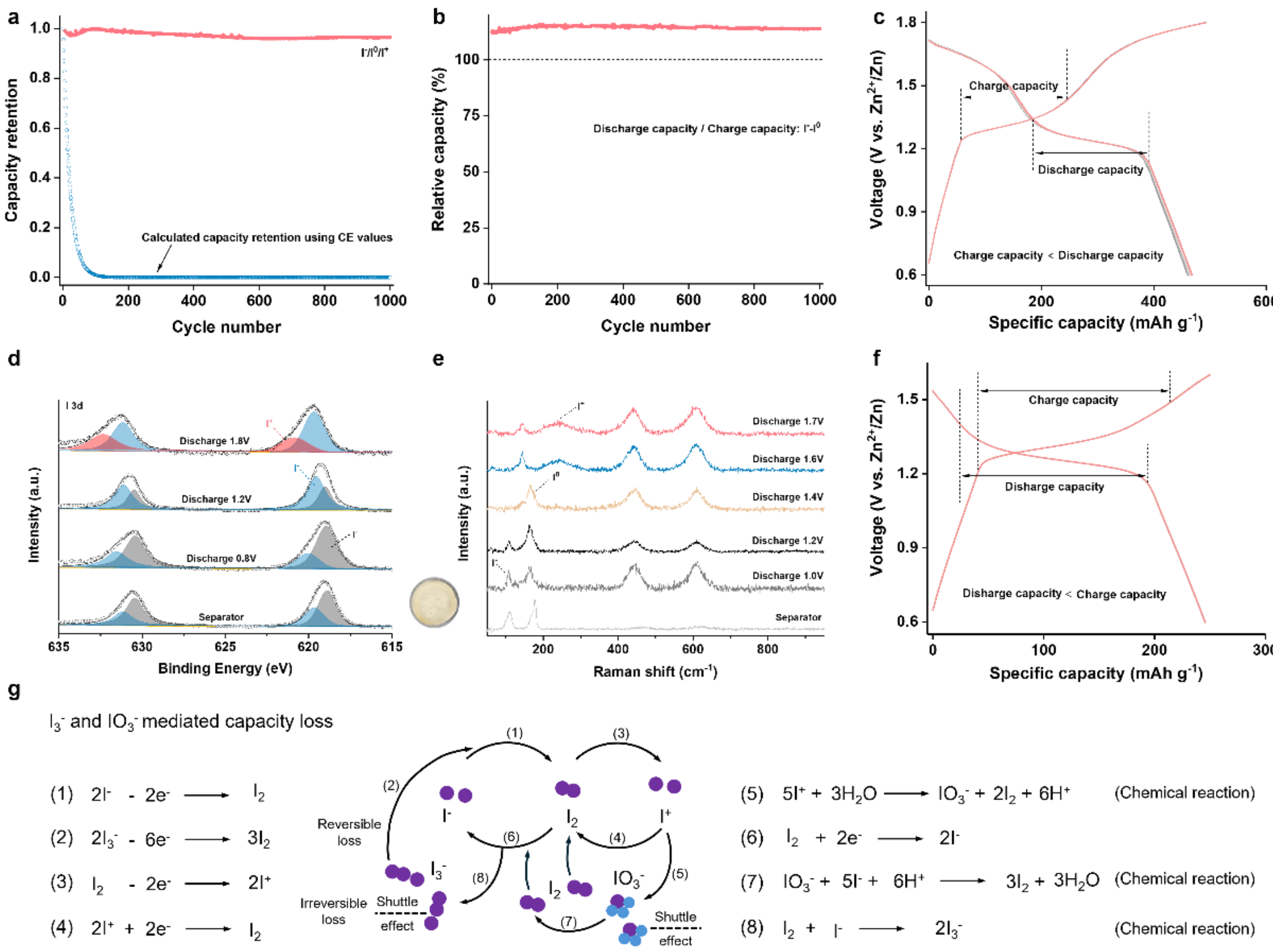

Fig. R4 Mechanistic analysis of the coupled $I^-/I^0/I^+$ conversion reactions in the KA-PA-Nuc electrolyte. a, Comparison between the measured capacity retention and the calculated capacity retention from CE values of the coupled $I^-/I^0/I^+$ conversion reactions. b, Relative capacity ratio between the discharge capacity and charge capacity of the I^-/I^0 plateaus in the coupled $I^-/I^0/I^+$ conversion reactions. c, GCD profiles of the coupled $I^-/I^0/I^+$ conversion reactions, showing that the discharge capacity of the first redox

process exceeds the corresponding charge capacity. d, XPS spectra of the cycled separator and iodine cathodes at different discharge states. e, Raman spectra of the cycled separator and iodine cathodes at different discharge states. f, GCD profiles of the $I^-/I^0/$ conversion reaction, showing that the discharge plateau is lower than the corresponding charge plateau. g, Schematic illustration of the conversion processes and degradation mechanism of the coupled $I^-/I^0/I^+$ conversion reactions.

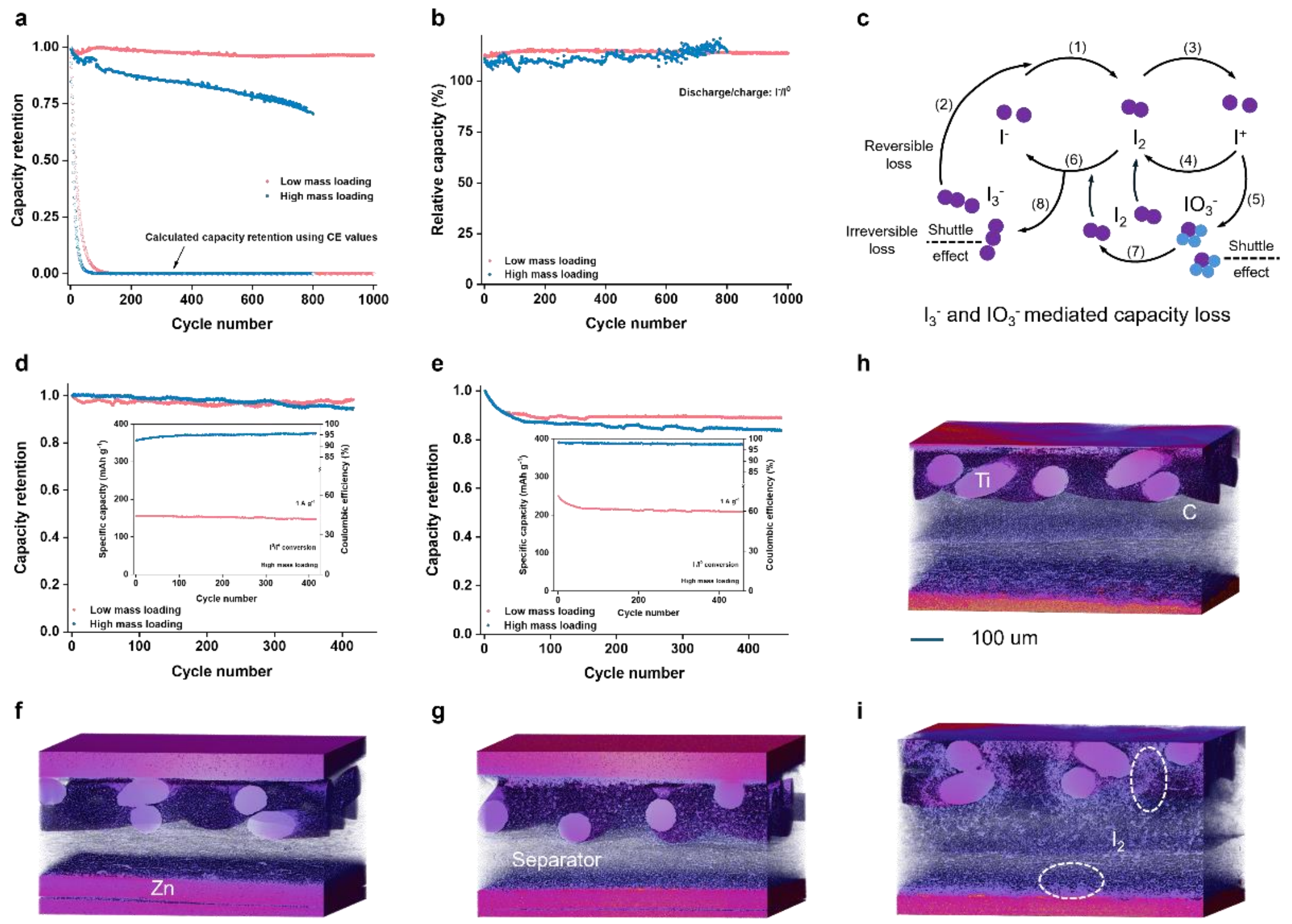

Fig. R5 Comparison of capacity fading behavior for the coupled $I^-/I^0/I^+$ conversion reactions at different mass loadings in the KA-PA-Nuc electrolyte. a, Comparison between the measured capacity retention and the calculated capacity retention at different mass loadings. b, Relative capacity ratio between the discharge capacity and charge capacity of the I^-/I^0 plateaus at different mass loadings. c, Schematic illustration of the conversion processes and degradation mechanism. d, Comparison of capacity retention of the decoupled I^0/I^+ conversion reaction at different mass loadings; inset shows the cycling performance of the decoupled I^0/I^+ conversion reaction at high mass loading. e, Comparison of capacity retention of the decoupled I^-/I^0 conversion reaction

at different mass loadings; inset shows the cycling performance of the decoupled I^-/I^0 conversion reaction at high mass loading. f, X-ray Micro-CT volume rendering of the low mass loading Zn-I_2 battery after 200 cycles of the coupled $I^-/I^0/I^+$ conversion reactions. g, X-ray Micro-CT volume rendering of the low mass loading Zn-I_2 battery after 500 cycles of the coupled $I^-/I^0/I^+$ conversion reactions. h, X-ray Micro-CT volume rendering of the high mass loading Zn-I_2 battery after 200 cycles of the coupled $I^-/I^0/I^+$ conversion reactions. i, X-ray Micro-CT volume rendering of the high mass loading Zn-I_2 battery after 500 cycles of the coupled $I^-/I^0/I^+$ conversion reactions. All X-ray Micro-CT images were collected at the end of the charge state of the first redox process.

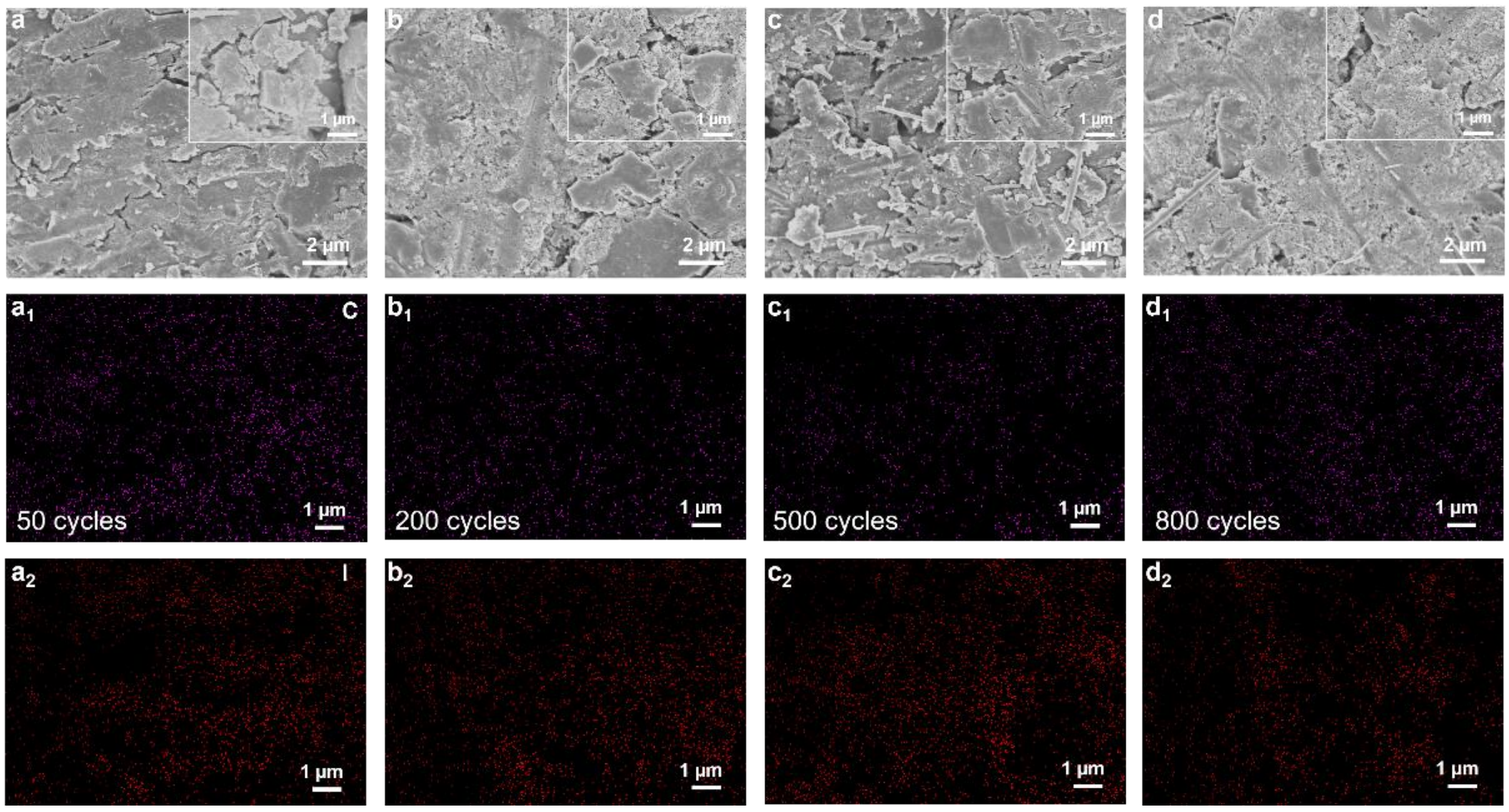

Fig. R6 Post-mortem analysis of cycled iodine cathodes at high mass loading. a-d, SEM images of the iodine cathodes after 50, 200, 500, and 800 cycles, respectively. a_1-d_1, EDS mappings of C element for the iodine cathodes after 50, 200, 500, and 800 cycles, respectively. a_2-d_2, EDS mappings of I element for the iodine cathodes after 50, 200, 500, and 800 cycles, respectively.

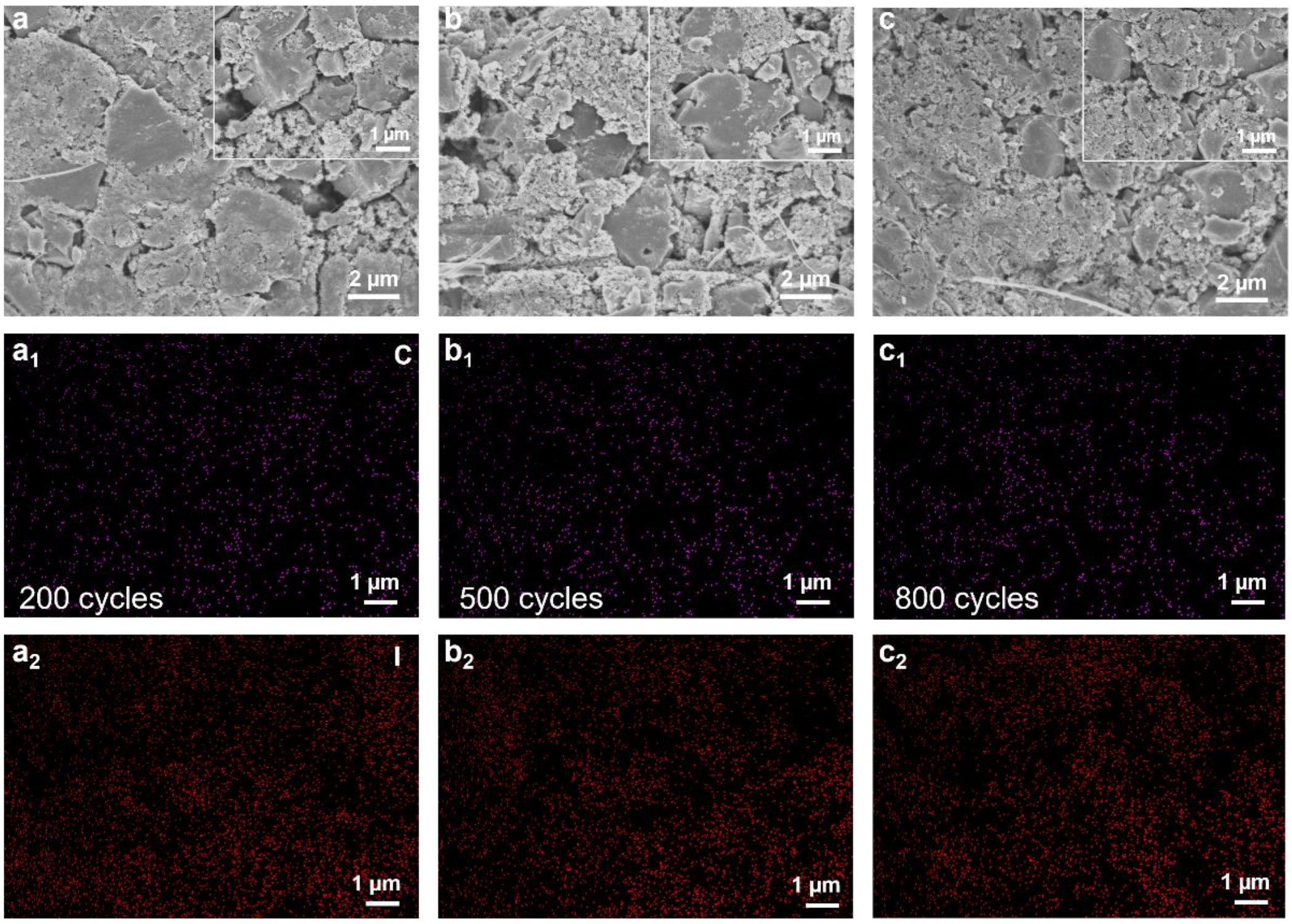

Fig. R7 Post-mortem analysis of cycled iodine cathodes at low mass loading after prolonged cycling. a-c, SEM images of the iodine cathodes after 200, 500, and 800 cycles, respectively. a_1-c_1, EDS mappings of C element for the iodine cathodes after 200, 500, and 800 cycles, respectively. a_2-c_2, EDS mappings of I element for the iodine cathodes after 200, 500, and 800 cycles, respectively.

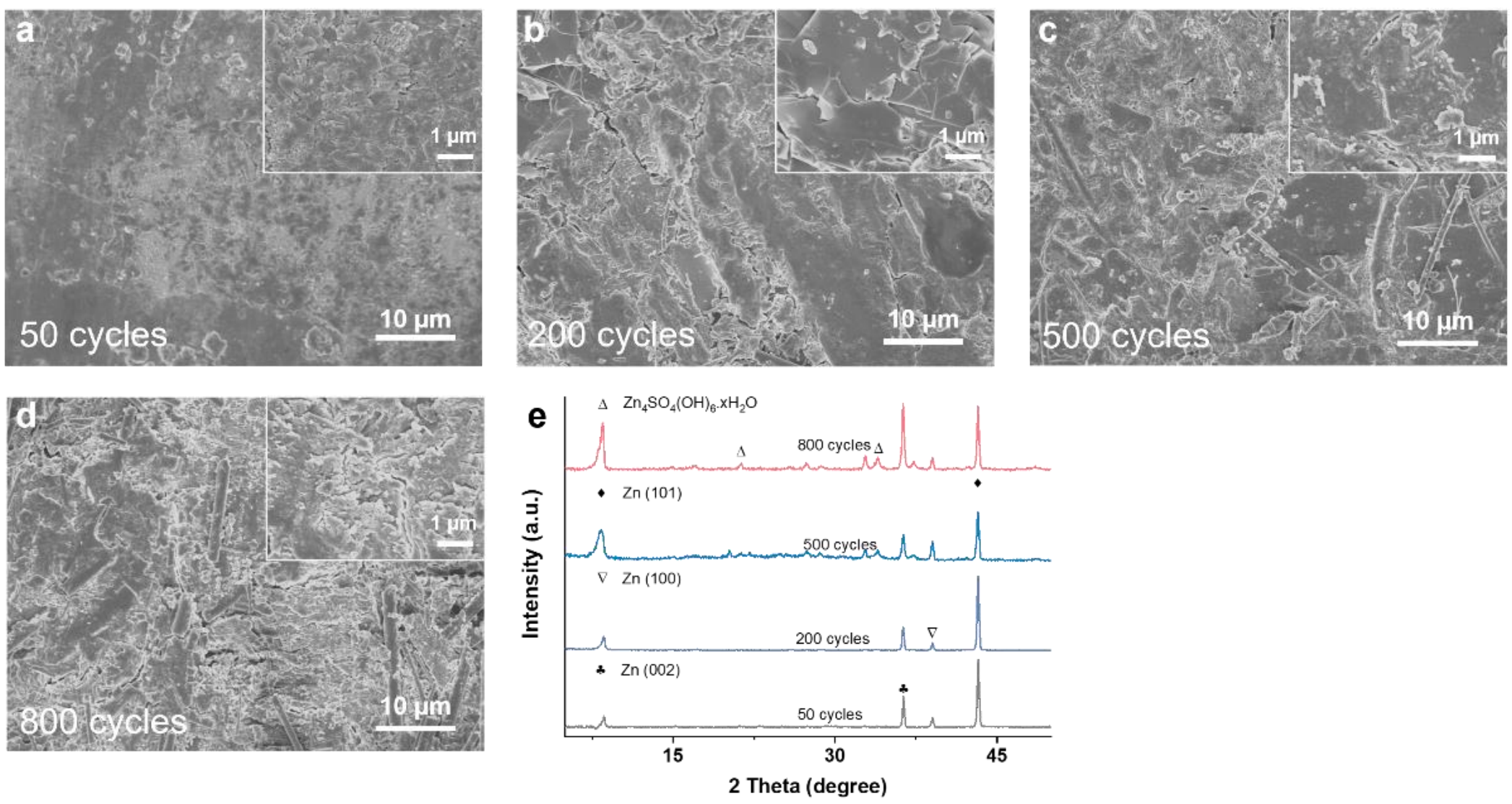

Fig. R8 Post-mortem analysis of cycled Zn anodes in high mass loading Zn-I$_2$ batteries. a-d, SEM images of the Zn anodes after 50, 200, 500, and 800 cycles, respectively. e, XRD patterns of the Zn anodes after various cycles.

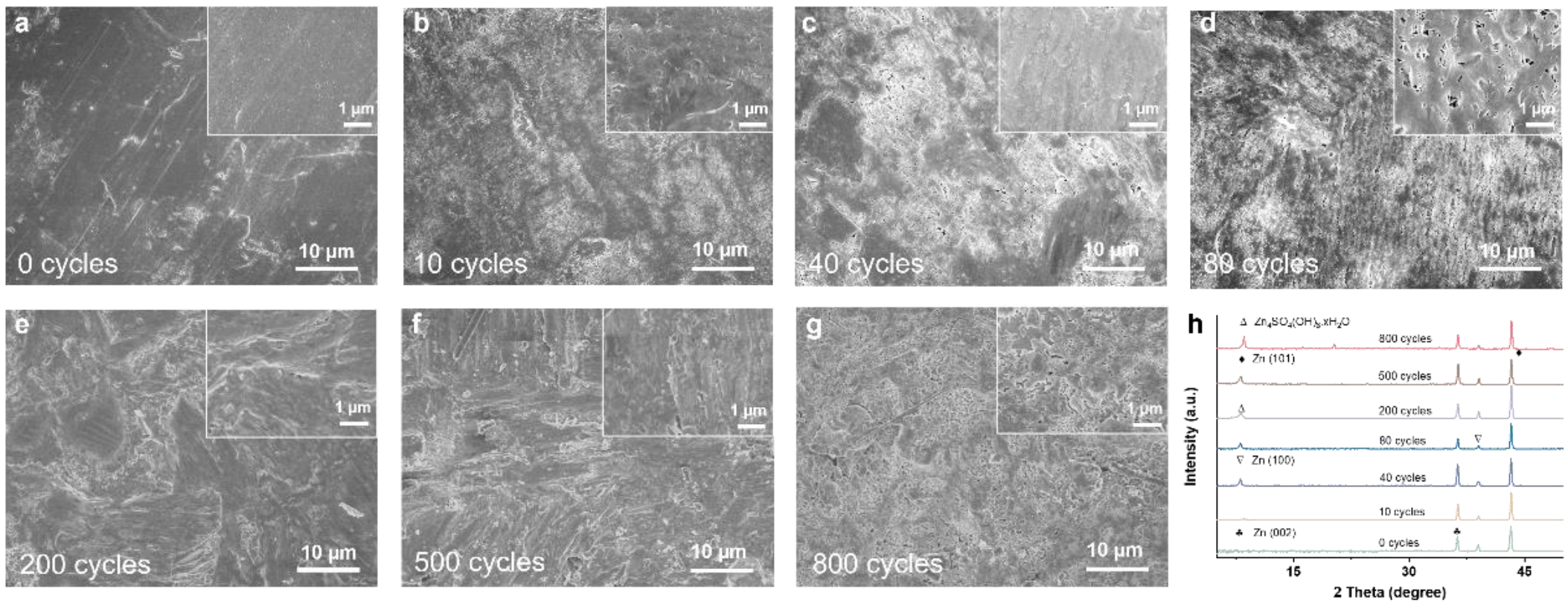

Fig. R9 Post-mortem analysis of cycled Zn anodes in low mass loading Zn-I$_2$ batteries. a-g, SEM images of the Zn anodes after 0, 10, 40, 80, 200, 500, and 800 cycles, respectively. e, XRD patterns of the Zn anodes after various cycles.

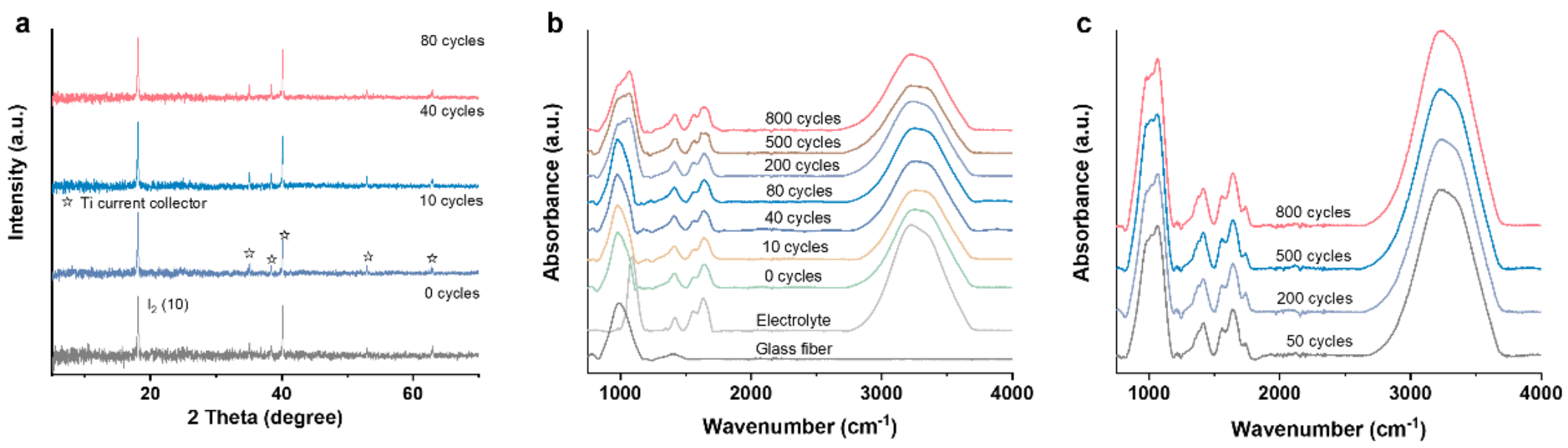

Fig. R10 Post-mortem analysis of cycled batteries. a, XRD patterns of the iodine cathodes after various cycles. b, FTIR spectra of KA-PA-NA electrolytes after various cycles in low mass loading Zn-I$_2$ batteries. c, FTIR spectra of KA-PA-NA electrolytes after various cycles in high mass loading Zn-I$_2$ batteries.

Comment 4: Final comments, related to minor side-issues. What is the reason for the very large capacity figures in the x-axis of SI Fig. 1a and of Fig. R26?

Response: We sincerely thank the reviewer for pointing out this issue. These large apparent capacities primarily arise from severe corrosion processes induced by halogen-containing electrolytes, rather than from iodine redox chemistry. As discussed in the introduction, previously reported four-electron iodine chemistry has generally relied on highly concentrated halogen-containing electrolytes to stabilize high-valent iodine species. However, these electrolytes are not only costly but also highly corrosive toward Zn anodes and metallic battery components. This interpretation is supported by the pronounced corrosion observed on both the Zn anode and the battery casing after charging in halogen-containing electrolytes, as shown in Supplementary Fig. 1b and Fig. R26. As a result, conventional coin-cell configurations cannot operate reliably under such electrolyte conditions, and specialized corrosion-resistant Swagelok cells are typically required for electrochemical evaluation of halogen-containing systems, as illustrated in Supplementary Fig. 1c. These observations further highlight the practical motivation behind establishing the KA–PA–Nuc electrolyte standard. To avoid further confusion, we have now added clarifications to the revised manuscript and figure captions, explicitly stating that the unusually large apparent capacities originate from corrosion-related parasitic processes in halogen-containing electrolytes rather than from iodine redox chemistry.

Comment 5: Final comments, related to minor side-issues. Comment 10 from R2 raises an apparently contentious issue regarding NMR proton shifts and hydrogen bonding. R2 argues for an upfield shift in the proton resonance, which is opposite to that proposed here. R2's argument appears sound, based on literature. The rebuttal talks in localized terms only, without considering the role of hydrogen bonding throughout bulk water. I think that a more extensive discussion of this issue is warranted.

Response: We agree that our previous response focused primarily on localized anion–

water–Nuc interactions and did not sufficiently discuss the broader role of the hydrogen-bond network throughout the bulk water. In response, we have expanded the mechanistic discussion and interpretation of the NMR results by considering both localized anion–water–Nuc interactions and the hydrogen bond network of bulk water.

As discussed in our previous response, the observed NMR results can be rationalized based on fundamental hydrogen-bonding principles. According to classical hydrogen bond theory, the hydrogen bond (X–H···Y) is a type of noncovalent interaction in which a hydrogen atom (H) covalently bonded to an electronegative atom (X) interacts with another electronegative atom (Y, such as O, N, or F). Formation or strengthening of such interactions usually polarizes the X–H bond, decreases the shielding of the hydrogen nucleus, and therefore leads to a downfield NMR shift. Conversely, increased electron density around electronegative atom Y enhances shielding and typically results in an upfield NMR shift. This interpretation aligns with the IUPAC definition of hydrogen bond: "The hydrogen bond leads to characteristic NMR signatures that typically include pronounced proton deshielding for H in X–H" (*Pure Appl. Chem., Vol. 83, No. 8, pp. 1637–1641, 2011*). These fundamental principles form the basis of our interpretation.

Importantly, the experimentally observed NMR signals inherently represent the dynamically averaged electronic environments arising from the coupled localized interactions throughout the bulk solution. In this context, localized interactions and bulk the extended hydrogen bond network are intrinsically interconnected rather than independent phenomena. Specifically, local anion–water interactions can propagate through and reorganize the surrounding hydrogen bond network of water, while the reorganized bulk-water structure can further modulate local interactions. Therefore, the measured NMR shifts reflect the averaged electronic structure generated by the coupled anion–water–Nuc interaction network across the entire electrolyte.

In our electrolyte system, kosmotropic anions (commonly referred to as "structure-making" anions), especially SO_4^{2-}, strongly interact with and polarize surrounding water molecules. Such interactions not only establish strong local anion–water

coordination but also reorganize and strengthen the hydrogen bond network throughout the bulk water. This enhanced polarization and increased structuring of water decrease proton shielding and therefore produce the observed downfield ^2H NMR shifts. Accordingly, the progressive downfield trend observed in the ^2H NMR spectra (SO_4^{2-} > CH_3COO^- > NO_3^- > $CF_3SO_3^-$ > ClO_4^-) reflects increasing anion-induced polarization and structuring of the bulk water hydrogen bond network, rather than merely a simple localized anion-water coordination. This interpretation is also consistent with the anion-specific effects and with previous electrolyte studies reporting similar downfield trends under strengthened anion–water interactions (*Adv. Mater. 2022, 34, 2110140; Nat. Sustain. 2025, 8, 1349–1359*).

More importantly, this distinction in water-anion coordination and bulk water structuring will significantly affect water-Nuc interactions. Strong anion–water coordination effectively polarizes and sequesters the hydration water around Nuc molecules, thereby weakening or reducing hydrogen bonds between water and the nitrogen atoms in Nuc. The weakened or reduced hydrogen bonds reduces electron donation from hydrogen to nitrogen, resulting in deshielding and corresponding downfield ^{15}N NMR shifts. Similarly, the observed downfield shift in the ^{15}N NMR spectra should not be interpreted simply as reduced localized water–Nuc hydrogen-bond. Rather, it reflects the average electronic environment around the Nuc nitrogen atoms through the bulk solution. This interpretation is further supported by our MD simulations, which reveal reduced Nuc–water hydrogen bond numbers and altered N···H distance distributions in the presence of kosmotropic anions. Collectively, the ^2H and ^{15}N NMR shifts give complementary evidence for anion-specific effects for harnessing anion–water–Nuc interaction network. These results further support the central mechanistic role of kosmotropic anions, particularly SO_4^{2-}, in reorganizing the bulk water hydrogen-bond network, weakening water–Nuc interactions, and ultimately promoting the stabilization of high-valent iodine intermediates through halogen bonding, thereby enabling reversible four-electron iodine conversion chemistry.

We hope that the revised discussion now provides a more comprehensive and

mechanistic interpretation of the NMR results by integrating local coordination chemistry, bulk water hydrogen bond network, and fundamental hydrogen bond theory. Accordingly, the relevant discussion has been revised in the manuscript.

Responses to referees #4

Comment 1. The authors have now examined in commendable detail the causes of capacity loss in these Zn-I_2 cells and then thoroughly explained the effects in terms of iodine chemistry. The complex relationship between charge passed in the various reactions and the many reaction pathways is well explained and, as posited by the authors, will help guide future research.

Response: We sincerely thank the reviewer for the careful and thoughtful evaluation of our revised manuscript and for the considerable time and effort devoted to assessing the additional experimental evidence and mechanistic analyses. We particularly appreciate the reviewer's positive recognition that the origins of capacity loss, the associated iodine chemistry and the complex relationships among the different reaction pathways have now been examined and clarified in substantially greater detail.

We also fully acknowledge the reviewer's further concerns regarding the remaining limitations of the present Zn-I_2 system. In response, we have carefully clarified each of these points individually in the following sections, including triiodide migration and Zn-side corrosion, the amount of excess Zn used in the experimental cells, the assumptions underlying the techno-economic analysis, the rate dependence of Coulombic efficiency and capacity retention, and the wording used to describe the mitigation of parasitic reactions. We sincerely hope that these additional clarifications and revisions fully address the reviewer's remaining concerns.

Comment 2. So where does all this new information leave us?

From the added discussion, it is clear that fugitive triodide, in particular, causes cycle-life-limiting effects. In the revised SI, the authors note that the abundance of basic zinc sulfate on the anode grows with cathode loading and cycle number. From the discussion in the rebuttal, but not included in the revised SI, they note that this is due to corrosion of zinc. This will come as no surprise to electrochemist readers. Most of the latter would agree that triiodide-related corrosion thwarted the progress of the Graetzel cell (before it was eventually killed off by cheap silicon).

Response: We thank the referee for this important and broader question. We agree that fugitive I_3^- can promote parasitic reactions and Zn corrosion, which may lead to cycle-life-limiting effects. However, the additional mechanistic and post-mortem analyses have helped us more clearly define the broader scientific understanding that emerges from the present work, extending beyond the established role of polyiodide-induced Zn corrosion. In addition, we would like to clarify the central contributions of this study at several levels.

First, the central scientific contribution of this work is the establishment of a halogen-free electrolyte chemistry framework for reversible four-electron iodine conversion and the associated mechanistic shift. Conventional four-electron iodine chemistry in aqueous environments has relied predominantly on highly concentrated, halogen-containing electrolytes, in which reactive hypervalent iodine species are stabilized through interhalogen complexation. Here, we establish a distinct KA-PA-Nuc electrolyte framework based on anionic chemistry and halogen bond chemistry that enables reversible $I^-/I^0/I^+$ chemistry without relying on this conventional electrolyte environment and stabilization mechanism. The central advance is therefore not simply an improvement in battery performance, nor do we intend to present the KA-PA-Nuc electrolyte as a complete solution to all degradation processes. Rather, this work establishes a different electrolyte chemistry framework and stabilization mechanism for

accessing reversible four-electron iodine redox chemistry in aqueous media.

Second, beyond establishing this electrolyte framework, this work provides a mechanistic understanding of how electrolyte chemistry governs iodine conversion across molecular, interfacial and electrochemical scales. Our combined experimental and theoretical results reveal how anionic chemistry regulates both the interfacial microenvironment and molecular interactions: PA controls the interfacial distribution of electrolyte species and water, whereas KA regulates water-Nuc interactions and thereby the availability of Nuc for halogen bond stabilization of I^+. These molecular and interfacial effects are further correlated with the interfacial electrochemical behaviour and ultimately with the iodine conversion pathways. The resulting mechanistic picture therefore connects electrolyte chemistry, interfacial microenvironment and molecular interactions, interfacial electrochemical processes, and iodine conversion chemistry, providing a mechanistic basis for the future design of multielectron aqueous halogen batteries.

Third, the additional analyses of interfacial electrochemical behaviour and iodine inventory provide a more process-resolved understanding of the interfacial reactions and degradation pathways underlying four-electron iodine conversion. We agree with the referee that polyiodide-induced corrosion is a well-established cause of cycle-life limitation. However, previous descriptions of aqueous iodine chemistry have commonly treated high-valent iodine instability as “hydrolysis” and lower-valent iodine loss as “polyiodide shuttling”, without resolving how these processes evolve and interact during four-electron conversion or how they contribute differently to apparent CE loss and persistent capacity decay. Here, *in situ* EIS-DRT analysis decouples the complex impedance response into specific electrochemical processes, revealing stable interfacial reaction dynamics in the KA-PA-Nuc electrolyte and identifying charge transfer as the dominant kinetic bottleneck under the investigated conditions. In parallel, decoupling the iodine chemistry into the I^0/I^+, I^-/I^0 and coupled $I^-/I^0/I^+$ processes identifies distinct IO_3^-- and I_3^--mediated degradation pathways and reveals how they become coupled during the full four-electron reaction. This analysis distinguishes

reversible iodine redistribution from irreversible iodine inventory loss, explaining why apparent CE loss does not translate directly into proportional capacity decay. Thus, rather than treating iodine-related degradation simply as hydrolysis or a single shuttle phenomenon, our analysis resolves the underlying chemical and electrochemical processes and connects their evolution to reaction kinetics, CE and long-term capacity decay. These insights further show that sustained four-electron iodine conversion requires not only stabilization of I^+ against hydrolysis, but broader management of the entire iodine inventory.

Fourth, we apologize if our previous presentation gave the impression that the relationship between I_3^- migration and Zn corrosion was discussed only in the rebuttal. This relationship is now explicitly addressed in the revised Supplementary Information (*Supplementary Information, page 74: These behaviors originate from two coupled factors. First, compared with low loading systems, high loading Zn-I_2 cells involve larger areal capacities during cycling, thereby amplifying Zn negative electrode parasitic reactions. Second, under high loading conditions, more severe I_3^- migration and redistribution further accelerate Zn negative electrode corrosion. This interpretation is consistent with the electrochemical results and the Micro-CT visualization of iodine redistribution. Accordingly, the more pronounced Zn-side degradation at higher loading should not be attributed solely to I_3^- migration. Moreover, I_3^- induced Zn degradation represents one pathway associated with irreversible iodine inventory loss, rather than the ultimate failure mode, while migrated iodine species may also become irreversibly retained within the separator or bulk electrolyte without necessarily reaching the Zn negative electrode.*). Specifically, the post-mortem analysis shows progressively stronger $Zn_4SO_4(OH)_6 \cdot xH_2O$ formation and rougher Zn surfaces with increasing iodine loading and cycling number. We attribute these observations to two coupled factors: larger areal capacities amplify Zn-side parasitic reactions, while more pronounced I_3^- migration and redistribution further accelerate Zn corrosion. However, these observations do not establish polyiodide-induced Zn corrosion as the sole or dominant cause of capacity decay. Rather, they identify it as one component of

the broader degradation processes that becomes increasingly relevant at higher iodine loadings and upon prolonged cycling. The coupled degradation mechanism identified above distinguishes reversible iodine redistribution from irreversible iodine inventory loss, with the latter being directly responsible for persistent capacity decay. Polyiodide-induced Zn corrosion represents one pathway contributing to this irreversible loss, while migrated iodine species can also become irreversibly retained within the separator and bulk electrolyte rather than being consumed exclusively through reactions at the Zn negative electrode.

Taken together, the new information therefore leads us to a more defined mechanistic picture than the conclusion that I_3^--induced corrosion limits cycle life. At a broad level, the work establishes a halogen-free electrolyte chemistry framework and a different stabilization mechanism for four-electron iodine conversion. At the mechanistic level, it reveals how anionic chemistry regulates molecular interactions and the interfacial microenvironment, and how these effects propagate into interfacial electrochemical behaviour and iodine conversion pathways. At the electrochemical reaction level, process-resolved electrochemical and iodine inventory analyses distinguish reversible redistribution from irreversible iodine inventory loss and identify how IO_3^-- and I_3^--mediated pathways become coupled during four-electron conversion. At the same time, the remaining degradation, including polyiodide-induced Zn corrosion, defines an important limitation. These findings indicate that further development will require not only stabilization of high-valent iodine species, but also broader control over the generation, migration and recovery of iodine intermediates, together with positive electrode confinement, separator engineering and negative electrode protection. We have revised the manuscript and Supplementary Information accordingly to more clearly reflect this mechanistic understanding, explicitly acknowledge the remaining degradation pathways and limitations, and define the corresponding directions for further development (*Main text, page 7; Supplementary Information, Mechanistic investigations and post-mortem analysis, pages 69-76*).

Comment 3. I am concerned that zinc anode failure limits cycle-life in a cell where the

zinc is presumably in significant excess. It's not clear how large the excess is though. While the technoeconomic (TE) modelling uses a factor of two, the Methods Section does not provide this parameter for the actual test cells. There is an interesting point here. While the authors show that a significant part of the charge loss responsible for low CE is associated with fundamentally reversible reactions, this is somewhat unimportant with respect to what appears to be the ultimate failure mode. Regardless of whether the triiodide formed is part of a reversible chain of reactions, the fact that some of it escapes the confines of the cathode and then reacts with the anode is clearly cell-life-limiting. In a way, the high degree of overall reversibility actually guarantees that a constant supply of triiodide will be available to react with the anode.

Response: We thank the reviewer for raising these important points regarding the amount of excess Zn, the distinction between the experimental cell configuration and the techno-economic modelling, and the interpretation of the ultimate failure mechanism.

First, we agree that the amount of excess Zn in the experimentally tested cells should be explicitly reported. Following the reviewer's comment and the Editor's guidance, we have therefore added corresponding parameters including Zn foil thickness, electrode dimensions, and iodine loading for the representative cell configurations to the revised manuscript and Supplementary Information.

Second, we clarify that the negative-to-positive capacity ratio of two used in the techno-economic analysis is a theoretical modelling assumption and does not represent the electrode balancing employed in the experimental cells. The TEA uses standardized and simplified modelling parameters to place different electrolyte chemistries on a broadly comparable basis and to examine their theoretical cost potential, particularly the implications of replacing highly concentrated halogen-containing electrolytes with the KA-PA-Nuc electrolyte, rather than to demonstrate immediate technological or economic competitiveness. We have now explicitly stated this distinction in both the calculation methodology and the corresponding discussion to avoid confusion. In accordance with the Editor's guidance, we have also moved the TEA entirely to the

Supplementary Information and revised the corresponding discussion to emphasize its theoretical nature while avoiding direct claims of competitiveness with established battery technologies (*Supplementary Information, pages 57-58, 79-83*).

Third, we believe that distinguishing reversible from irreversible iodine loss remains mechanistically important. We agree with the referee that I_3^--induced negative electrode corrosion represents an important degradation pathway and may become cycle-life limiting. However, our mechanistic analyses do not establish this process as the ultimate failure mechanism of the cell. Our mechanistic analyses instead indicate a broader coupled iodine inventory degradation mechanism. Specifically, the apparent charge loss contains both reversible iodine redistribution, in which iodine species remain available for subsequent electrochemical reutilization, and irreversible iodine inventory loss, in which iodine species becomes unavailable for subsequent cycling. It is this latter irreversible component that directly produces capacity decay. It is worth noting that this irreversible iodine inventory loss does not arise exclusively from polyiodide-induced Zn corrosion. Migrated iodine species can become irreversibly retained within the separator and bulk electrolyte, in addition to reaching the Zn negative electrode and participating in parasitic reactions. Thus, polyiodide-induced Zn corrosion represents one contribution to irreversible iodine inventory loss rather than the sole or ultimate failure mode.

Fourth, this distinction is important not only for interpreting the relatively low apparent CE, but also for identifying the mechanistic origin of capacity loss and the corresponding mitigation strategies. If degradation were considered solely as polyiodide crossover followed by Zn corrosion, the principal strategies would be to suppress polyiodide migration or protect the Zn negative electrode. In contrast, our results indicate that durable four-electron iodine chemistry requires simultaneous regulation of the entire iodine inventory: stabilization of high-valent iodine against hydrolysis, confinement of iodine intermediates to suppression of their irreversible distribution into the electrolyte and separator, and mitigation towards Zn negative electrode. The distinction between reversible redistribution and irreversible inventory

loss is therefore not merely an interpretation of CE; it identifies where iodine is lost, through which chemical pathway, and consequently which intervention is required to suppress that loss.

We have revised the corresponding discussion in the Supplementary Information accordingly, explicitly acknowledging Zn-side degradation as an important remaining limitation, particularly at increased iodine loading, while placing it within the broader iodine inventory degradation mechanism.

Supplementary Information, page 74

These observations confirm that accelerated capacity fading and noisier CE profiles under high loading conditions originate predominantly from intensified I_3^--mediated migration and redistribution pathways (Supplementary Fig. 59), therefore representing another remaining limitation of iodine conversion chemistry.

Supplementary Information, pages 74-75

Accordingly, the more pronounced Zn-side degradation at higher loading should not be attributed solely to I_3^- migration. Moreover, I_3^- induced Zn degradation represents one pathway associated with irreversible iodine inventory loss, rather than the ultimate failure mode, while migrated iodine species may also become irreversibly retained within the separator or bulk electrolyte without necessarily reaching the Zn negative electrode.

Comment 4. The TE analysis generates a comparison (Fig. 6f) in which the authors' version of Zn-I_2 betters all compared chemistries by a considerable margin. However, the analysis seems to be missing some details. My main question concerns the cathode. There is no costing of the actual cathode material (i.e., iodine deposited on to activated carbon substrate). Simply costing iodine as the cathode reduces the total cost unrealistically. For the anode, does the twofold excess relate to any of the cells made and tested in this study? In other words, given the corrosion issue, what order of cycle-life would this excess provide?

Response: We thank the reviewer for identifying these important limitations of the techno-economic analysis. We agree that the original analysis should not be interpreted as a complete cost assessment of the experimentally tested Zn-I_2 cells. In particular, costing iodine alone does not fully capture the cost of the activated carbon-iodine composite. We also clarify that the negative-to-positive capacity ratio of 2 used in the model is a theoretical assumption and does not correspond directly to the electrode balancing employed in our laboratory cells.

We would, however, like to clarify the original purpose and scope of this analysis. At the electrolyte level, the primary comparison was performed between the KA-PA-Nuc electrolyte and conventional highly concentrated, halogen-containing electrolytes under the same modelling framework. This comparison was intended to isolate the potential cost implications of the electrolyte design itself, particularly those arising from avoiding large quantities of concentrated halide salts and the associated use of more expensive corrosion-resistant current collectors. In this context, the analysis provides an illustrative comparison of the material-cost implications of different electrolyte chemistries for the same four-electron Zn-I_2 chemistry, rather than a prediction of the cost of the experimentally tested cells.

The broader cross-chemistry comparison serves a different purpose. Standardized and simplified modelling parameters representative of mature cell designs were used to place different battery chemistries on a broadly comparable basis and to examine the theoretical cost potential of four-electron Zn-I_2 chemistry. These parameters, including the assumed N/P ratio, are not derived from the present laboratory cells. The resulting estimates therefore represent a theoretical cost benchmark rather than evidence of immediate economic or technological competitiveness with established battery technologies. We have now made the theoretical nature and limitations of these assumptions explicit in the calculation methodology and associated discussion.

We agree with the reviewer that the original presentation, particularly the direct cross-chemistry comparison in Fig. 6f, could give a stronger impression of practical cost advantage than warranted by these assumptions. Accordingly, following the

reviewer's concern and the Editor's guidance, we have removed the techno-economic analysis and the corresponding figure from the main text to avoid any potential misinterpretation of the theoretical cost potential projected under standardized modelling assumptions as the immediate economic or technological competitiveness with Li-ion, K-ion or other battery technologies. The analysis retained in the Supplementary Information has been reframed as an illustrative theoretical assessment (*Supplementary Information, pages 57-58, 79-83*).

We therefore emphasize that the purpose of this analysis is to illustrate two aspects: the relative material-cost benefit of the KA-PA-Nuc electrolyte compared with conventional highly concentrated, halogen-containing electrolytes under equivalent modelling conditions, and the longer-term cost potential of four-electron Zn-I_2 chemistry under hypothetical practical cell design parameters. Neither comparison is intended to imply that the present laboratory cells have already achieved these practical parameters or an immediate cost advantage over mature battery technologies. We believe that removing this analysis from the main text while retaining a clearly qualified theoretical assessment in the Supplementary Information more appropriately reflects both its value and its limitations.

Comment 5. The point that I raised in my review regarding the sensitivity of cycle performance to current (rate) was not addressed directly. It is clear to me that this cell chemistry will become less efficient, and lose capacity at a greater rate, as the rates of charge and discharge are lowered. This would limit its application significantly.

Response: We thank the reviewer for returning to this important point, and we apologize that the inverse relationship between current density and CE was not addressed sufficiently in our previous response. In the previous revision, we considered this observation within our broader investigation of the origin of low CE and capacity fading, but we recognize that we did not explicitly discuss its rate dependence.

We agree with the reviewer that the lower CE observed at lower current densities indicates an increased contribution from parasitic processes. Our mechanistic analyses

suggest that this inverse rate-CE relationship arises primarily from the time-dependent evolution of iodine species. At lower current densities, each charge-discharge process proceeds over a longer period, providing more time for competing parasitic processes involving iodine intermediates, including I^+ hydrolysis and I_3^- redistribution. At higher current densities, the shorter reaction time reduces the extent of parasitic processes within each cycle, and the apparent CE consequently approaches 100%. We have now explicitly clarified this point in the revised discussion in the Supporting Information (*Supplementary Information, pages 72-73*).

Our mechanistic analyses further show that the relationship between apparent CE and irreversible capacity loss is more complex than a direct correspondence. By separately examining the I^0/I^+ and I^-/I^0 processes, we identified substantial discrepancies between the capacity retention expected from the cumulative CE and that measured experimentally, indicating that the apparent charge imbalance reflects both recoverable iodine species evolution and irreversible iodine inventory loss. For example, the decoupled I^0/I^+ process maintains stable cycling despite an apparent CE of approximately 94%. In this process, part of the apparent charge imbalance arises from I^+ hydrolysis-derived iodine species that remain within the positive electrode region and can subsequently re-enter the electrochemical reaction, whereas persistent IO_3^- formation contributes to irreversible iodine inventory loss. Similarly, during the I^-/I^0 process, most dissolved I_3^- can return to the positive electrode region and participate in subsequent cycling, whereas the unrecovered fraction contributes to irreversible iodine inventory loss.

This distinction is particularly relevant to the rate dependence highlighted by the reviewer. We agree that slower rate is less efficient and provides more time for parasitic processes, and we do not intend to suggest that decreasing the rate has no adverse effect on long-term cycling. Rather, our results show that the relationship among rate, apparent CE and irreversible capacity fading is not directly proportional. Although the lower CE at reduced current density reflects greater involvement of time-dependent parasitic processes, part of the associated iodine species evolution is recoverable and

therefore does not translate directly into permanent capacity loss. Consistent with this distinction, the KA-PA-Nuc cell retains 96% of its capacity after 1,000 cycles at 1 A g^{-1} despite the lower CE observed at this current density.

We nevertheless recognize the rate dependence identified by the reviewer as an important remaining limitation of the present four-electron iodine chemistry. Longer charge-discharge durations provide more time for parasitic processes, increasing the likelihood of irreversible iodine inventory loss. This behaviour is consistent with the degradation mechanisms identified in our mechanistic analyses and indicates that further progress will require cell-level strategies that complement the electrolyte framework established here and enable broader management of the entire iodine inventory. We have revised the Supplementary Information accordingly to explicitly discuss this rate dependence and acknowledge it as a remaining challenge.

Supplementary Information, pages 72-73

This distinction also explains the rate dependence of CE. At lower current densities, longer charge-discharge durations provide more time for parasitic processes, thereby lowering the apparent CE. However, because part of the associated iodine species evolution is recoverable, the decrease in CE does not translate proportionally into irreversible capacity loss. Nevertheless, the increased contribution of parasitic processes at lower rates remains an important limitation.

Comment 6. In the revised version, line 439 states: 'The KA–PA–Nuc electrolyte effectively mitigates these parasitic pathways…'

I disagree – 'effectively' implies that the issue of product migration has been neutralised when clearly it has not – the evidence shows that the corrosion of the zinc anode ultimately limits cycle-life.

Response: We thank the reviewer for pointing out that the term "effectively" could overstate the extent to which these parasitic processes are suppressed. We agree that the KA-PA-Nuc electrolyte does not eliminate these iodine-associated parasitic pathways.

We have therefore removed "effectively" from the revised manuscript and explicitly clarified this in the Supplementary Information (*Supplementary Information, page 73*).

Nevertheless, our experimental evidence supports a mitigating effect of the KA-PA-Nuc electrolyte on these processes. The purpose of the original statement was not to imply complete elimination of these parasitic pathways, but to describe the collective evidence showing that the anion-enriched, water-deficient interfacial microenvironment, together with halogen bond stabilization, promotes the stabilization and confinement of iodine intermediates. The electrochemical measurements show stable four-electron cycling, while the spectroscopic and spatial analyses demonstrate improved confinement and reversibility of iodine species in the KA-PA-Nuc electrolyte. Consistent with this interpretation, the mechanistic analyses show that iodine intermediates remaining within the positive electrode/interfacial region can be reutilized during subsequent electrochemical processes. This mitigation persists across different iodine loadings, although the residual iodine migration and associated Zn degradation become more evident at higher loadings. As discussed in detail in our response to Comment 2, a more pronounced Zn-side degradation at higher loading arises from two coupled contributions: the larger areal reaction capacity increases the extent of intrinsic Zn-side parasitic reactions, while increased iodine-species migration and redistribution further promote Zn-side degradation. Thus, a greater Zn-side degradation observed at higher loading should not be attributed solely to iodine species migration.

Supplementary Information, page 73

This is precisely the key role of the KA-PA-Nuc electrolyte framework, which not only confines and stabilizes I^+ species, but also suppresses iodine species migration and redistribution through the synergistic interplay of anion-specific interfacial chemistry and halogen bond stabilization within an anion-enriched, water-deficient interfacial environment. Notably, these effects mitigate, but do not eliminate the associated parasitic pathways.

Comment 7. My overall opinion is that this work is still not suitable for publication in Nature. While the authors have now described essentially every aspect of this system in great detail, I disagree with the contention that this cell chemistry is, in the guise presented here, competitive with contemporary battery technologies. The flaws in this version of Zn-I_2 that have been identified will have seriously deleterious operational consequences. The fact that these flaws are lessened by the new electrolyte is important, but does not remove them from consideration. The benchmark in battery performance set by lithium-ion, and being followed closely by sodium-ion, continues to improve. In response, any new competing chemistry must be better still. At present, Zn-I_2 does not look like a plausible contender, and therefore this study does not generate the impact required for publication here.

Response: We sincerely thank the reviewer for this broader assessment and for clearly articulating the basis of the remaining concern. We agree that the present Zn-I_2 cells, in their current laboratory configurations, should not be interpreted as immediately competitive alternative to mature Li-ion or rapidly developing Na-ion battery technologies. We also agree that the residual irreversible iodine inventory loss and associated Zn negative electrode parasitic reactions remain important limitations that must be addressed before this chemistry can be considered for practical deployment.

We would, however, respectfully clarify that the central scientific contribution and intended scope of this work lie in the electrolyte chemistry and four-electron iodine conversion chemistry, rather than in establishing Zn-I_2 batteries as direct competitors to Li-ion or Na-ion batteries or presenting the KA-PA-Nuc electrolyte as a complete solution to all cell-level degradation processes. Reversible four-electron iodine chemistry in aqueous environments has conventionally relied on highly concentrated, halogen-containing electrolytes and interhalogen complexation. Here, we establish a distinct halogen-free KA-PA-Nuc electrolyte chemistry framework based on anionic chemistry and halogen bond chemistry that enables reversible $I^-/I^0/I^+$ conversion without relying on the conventional electrolyte environment and interhalogen stabilization mechanism. The central advance is therefore the establishment of a

different electrolyte chemistry and stabilization mechanism for accessing reversible four-electron iodine redox chemistry in aqueous media, together with the associated mechanistic shift, rather than simply an improvement in battery performance.

The additional investigations performed during revision further establish how this electrolyte chemistry governs iodine conversion across molecular, interfacial and electrochemical scales. Our combined experimental and theoretical results reveal that anionic chemistry simultaneously regulates the interfacial microenvironment and molecular interactions: PA controls the interfacial distribution of electrolyte species and water, whereas KA regulates water-Nuc interactions and thereby the availability of Nuc for halogen bond stabilization of I^+. These molecular and interfacial effects are further linked to interfacial electrochemical behaviour and ultimately to the iodine conversion pathways. Process-resolved analyses further clarify how the relevant iodine reaction and degradation pathways evolve during four-electron conversion. As also recognized by the reviewer in Comment 1, by decoupling the I^0/I^+, I^-/I^0 and coupled $I^-/I^0/I^+$ processes, we distinguish reversible iodine inventory loss from irreversible iodine inventory loss and identify the distinct roles of I^+ hydrolysis, IO_3^- formation and I_3^- migration. These results explain why apparent CE loss does not translate directly into proportional irreversible capacity loss and reveal how the stabilization, migration, redistribution, recovery and irreversible loss of iodine intermediates become coupled during cycling. The resulting mechanistic picture therefore connects electrolyte chemistry, interfacial microenvironment and molecular interactions, interfacial electrochemical processes, and iodine conversion chemistry, providing a broader basis for understanding reversible multielectron aqueous iodine chemistry.

At the same time, we recognize that establishing this electrolyte and mechanistic framework does not resolve all cell-level limitations. At higher iodine loadings, residual iodine species migration and redistribution become more pronounced, accompanied by increased Zn-side degradation. As clarified in our responses to Comments 2, 5 and 6, the KA-PA-Nuc electrolyte mitigates, but does not eliminate these processes, and their increased contribution at higher loading and lower rates remains an important limitation.

Further progress will therefore require broader cell-level strategies that build on the progress established here to achieve broader management of the entire iodine inventory.

We also recognize that the framing of the previous manuscript could have conveyed a stronger claim of technological competitiveness than was supported by the experimental cells. We have therefore substantially revised the manuscript in accordance with both the reviewer's concerns and the Editor's guidance. Claims implying immediate practical, economic or commercial competitiveness have been removed or moderated. The techno-economic analysis and cross-technology comparison have been removed from the main text and retained only in the Supplementary Information as a theoretical assessment. Broader performance data have been moved to the Supplementary Information. The main text has been reorganized around the electrolyte chemistry and its interfacial, molecular and electrochemical mechanisms. The remaining cell-level limitations and degradation pathways are explicitly acknowledged.

Accordingly, we do not contend that the present $Zn\text{-}I_2$ cell has already reached the level required to compete directly with Li-ion or Na-ion technologies. Rather, the significance of this work lies in establishing a halogen-free electrolyte chemistry framework and a distinct stabilization mechanism for reversible $I^-/I^0/I^+$ conversion in aqueous environments, together with a mechanistic understanding of how electrolyte chemistry governs this conversion across interfacial, molecular and electrochemical levels and where the remaining degradation pathways arise. We have revised the manuscript throughout to ensure that its claims are confined to these experimentally supported scientific advances, while unresolved cell-level challenges are explicitly acknowledged.
