# Supplementary material for: Non-halogen aqueous electrolytes for four-electron iodine conversion
Source: Nature. 2026 Nov 11;659(8140):372–7. doi: 10.1038/s41586-026-11282-0 (PMC13754563; doi:10.1038/s41586-026-11282-0)
Supplement: Supplementary file 2 [file 41586_2026_11282_MOESM1_ESM.docx]

**Supplementary Information**

**Non-Halogen Aqueous Electrolytes for Four-Electron Iodine Conversion**

Ruwei Chen^1#^, Haobo Dong^1#^, Yuhang Dai^2^, Jingyu Wang^1^, Jie Chen^1^, Jiyang Liu^1^, Jianrui Feng^1^, Yunpeng Zhong^1^, Wei Zong^2^, Wei Zhang^1^, Hang Yang^1^, Zhe Cui^1^, Guanjie He^1^*

*^1^ Department of Chemistry, University College London, London WC1E 7JE, UK*

*^2^ Department of Engineering Science, University of Oxford, Parks Road, Oxford, OX 3PJ, UK*

* Corresponding author email: [g.he@ucl.ac.uk](mailto:g.he@ucl.ac.uk)

^#^ These authors contributed equally to this work.

**Contents**

**SUPPLEMENTARY FIGURES**……………………………………………...……...7

**Supplementary Fig. 1** Electrochemical behavior of halogen-containing electrolytes…………………………………………………………………………….7

**Supplementary Fig. 2** Comparison of electrolyte characteristics……………………8

**Supplementary Fig. 3** The correlations and functions of three components in the proposed KA-PA-Nuc electrolyte framework…………………………………………9

**Supplementary Fig. 4** Electrolyte optimization and performance evolution………..10

**Supplementary Fig. 5** Comparison of electrochemical stability windows…………..11

**Supplementary Fig. 6** Molecular electrostatic potential of selected Nuc species………………………………………………………………………………..12

**Supplementary Fig. 7** Electrochemical performance of electrolytes without Nuc species………………………………………………………………………………..13

**Supplementary Fig. 8** Characterization of halogen bonds………………………….13

**Supplementary Fig. 9** Theoretical calculation of possible coordination configurations………………………………………………………………………..14

**Supplementary Fig. 10** Binding energy and bond length of Nuc-I^+^-Nuc halogen bonds with different counterions……………………………………………………………14

**Supplementary Fig. 11** Characterization of halogen bonds with different counterions………………………………………………………………………..….15

**Supplementary Fig. 12** Analysis of electrochemical products in the PA-Nuc electrolyte…………………………………………………………………………….17

**Supplementary Fig. 13** Electrochemical performance of the AC positive electrode in PA-Nuc electrolytes………………………………………………………………......17

**Supplementary Fig. 14** Comparison of electrochemical performance using urea as Nuc…………………………………………………………………………………...19

**Supplementary Fig. 15** Comparison of electrochemical performance using acetamide as Nuc………………………………………………………………………………...20

**Supplementary Fig. 16** Comparison of electrochemical performance using nicotinamide as Nuc………………………………………………………………….21

**Supplementary Fig. 17** Comparison of electrochemical performance using pyridine as Nuc…………………………………………………………………………………...22

**Supplementary Fig. 18** Electrochemical performance comparison of electrolytes with identical ionic strength……………………………………………………………….23

**Supplementary Fig. 19** Electrostatic potentials of three anions with similar structures……………………………………………………………………………..24

**Supplementary Fig. 20** Theoretical characterization of the bulk properties of the bare ZnSO_4_ (KA) electrolyte………………………………………………………………24

**Supplementary Fig. 21** Theoretical characterization of the bulk properties of the KA electrolyte with different polar anions………………………………………………...25

**Supplementary Fig. 22** Theoretical characterization of the bulk properties of the KA-Nuc electrolyte with different polar anions…………………………………………..26

**Supplementary Fig. 23** FTIR spectra of different electrolytes………………….…..27

**Supplementary Fig. 24** Theoretical characterization of the bulk properties of the highly concentrated ZnCl_2_ electrolyte (30 m ZnCl_2_)………………………….……..28

**Supplementary Fig. 25** Theoretical characterization of the interfacial properties of the highly concentrated ZnCl_2_ electrolyte (30 m ZnCl_2_)………………………………....28

**Supplementary Fig. 26** Differential capacitance curves in electrolytes with different anions………………………………………………………………………………...29

**Supplementary Fig. 27** Evolution of the interfacial environment in KA (SO_4_^2-^) electrolytes with different anions……………………………………………………30

**Supplementary Fig. 28** GCD profiles in electrolytes containing different polar anions………………………………………………………………………………...31

**Supplementary Fig. 29** Characterization of the spatial distribution of iodine species………………………………………………………………………………..32

**Supplementary Fig. 30** Characterization of the spatial distribution of iodine species………………………………………………………………………………..32

**Supplementary Fig. 31** Comparison of hydrogen bond and halogen bond………...35

**Supplementary Fig. 32** The anion-specific effects and typical anion-water-molecule interactions…………………………………………………………………………...36

**Supplementary Fig. 33** Cycling stability in electrolytes with different anions introduced into the same polar anion baseline……………………………………….36

**Supplementary Fig. 34** Electrochemical performance in the electrolyte containing CH_3_COO^-^……………………………………………………………………………..37

**Supplementary Fig. 35** Electrochemical performance in the electrolyte containing NO_3_^-^…………………………………………………………………………………..37

**Supplementary Fig. 36** Molecular structure of Nuc species…………………………38

**Supplementary Fig. 37** ^13^C NMR spectra of electrolytes containing different anions………………………………………………………………………………...38

**Supplementary Fig. 38** ^15^N NMR spectra…………………………………………..39

**Supplementary Fig. 39** Solubility test of Nuc species……………………………...39

**Supplementary Fig. 40** Theoretical calculations of water-Nuc interactions in electrolytes with different anions…………………………………………………….40

**Supplementary Fig. 41** In-depth analysis of interfacial electrochemical behaviors……………………………………………………………………………..41

**Supplementary Fig. 42** DRT profiles of the Zn negative electrode in different electrolytes at various charging states……………………………………………….45

**Supplementary Fig. 43** *In situ* EIS profiles of the iodine positive electrode in different electrolytes at various charging states……………………………………………….46

**Supplementary Fig. 44** Identification of the rate-limiting step…………………….47

**Supplementary Fig. 45** *In situ* EIS profiles of the two-electrode full cell at different charging rates………………………………………………………………………...49

**Supplementary Fig. 46** Corresponding DRT spectra of the two-electrode full cell at different charging rates………………………………………………………………49

**Supplementary Fig. 47** *In situ* EIS profiles of the individual iodine positive electrode at different charging rates…………………………………………………………...50

**Supplementary Fig. 48** Corresponding DRT spectra of the individual iodine positive electrode at different charging rates………………………………………………….50

**Supplementary Fig. 49** *In situ* EIS profiles of the individual Zn negative electrode at different charging rates………………………………………………………...……51

**Supplementary Fig. 50** Corresponding DRT spectra of the individual Zn negative electrode at different charging rates…………………………………………………51

**Supplementary Fig. 51** *In situ* Raman spectroscopy in the electrolyte with Nuc…………………………………………………………………………………...52

**Supplementary Fig. 52** *In situ* Raman spectroscopy in the electrolyte without Nuc…………………………………………………………………………………...52

**Supplementary Fig. 53** Electrochemical performance comparison………………...53

**Supplementary Fig. 54** GCD profiles in the 30 m ZnCl_2_ electrolyte at various current densities……………………………………………………………………………....53

**Supplementary Fig. 55** Cycling performance of Zn-I_2_ cells in the KA-PA-Nuc electrolyte at 2 A g^-1^………………………………………………………………….54

**Supplementary Fig. 56** Cycling performance of Zn-I_2_ pouch cell with a small size of 3 cm × 4 cm at a current density of 1A g^-1^…………………………………………….54

**Supplementary Fig. 57** Electrochemical performances of aqueous Al-I_2_ batteries…………………………………………………....…………………….…...55

**Supplementary Fig. 58** Electrochemical performances of aqueous Zn-I_2_ and Al-I_2_ batteries..……………………………………………………………….…………….56

**Supplementary Fig. 59** Electrochemical performance of Zn-I_2_ batteries in KA-PA-Nuc electrolytes at high iodine loadings.…………………..……….…………………….59

**Supplementary Fig. 60** Compatibility of the KA-PA-Nuc electrolyte with commercial current collectors.………………..…………………………………….…………….61

**Supplementary Fig. 61** Electrochemical behavior of commercial current collectors in halogen-containing electrolytes…………………………………….……………….62

**Supplementary Fig. 62** Comparison of capacity fading behavior for the coupled I^-^/I^0^/I^+^ conversion reactions at different mass loadings in the KA-PA-Nuc electrolyte……………………………………………………… ………………..….64

**Supplementary Fig. 63** Post-mortem analysis of cycled iodine positive electrodes at high mass loading.……..…………………………………………………………….65

**Supplementary Fig. 64** Post-mortem analysis of cycled iodine positive electrodes at low mass loading.……..…………………...……………………………………...….66

**Supplementary Fig. 65** Post-mortem analysis of cycled iodine positive electrodes at low mass loading after prolonged cycling……..…………………...………….…….67

**Supplementary Fig. 66** Post-mortem analysis of cycled Zn negative electrodes in high mass loading Zn-I_2_ batteries………………………………………………………….68

**Supplementary Fig. 67** Post-mortem analysis of cycled Zn negative electrodes in low mass loading Zn-I_2_ batteries.………………………………….………………….….69

**SUPPLEMENTARY TABLES**……………………………………………...……..77

**Supplementary Table 1** Comparison of aqueous electrolytes for multielectron iodine redox reactions………………………………………………………………………77

**Supplementary Table 2** Comparison of electrochemical performances in various electrolytes for multielectron iodine redox reactions………………………….……78

**Supplementary Table 3** Cost inputs of different batteries for techno-economic model analysis using BatPaC 5.0 modelling software……………………………………...81

**Supplementary Table 4** Cell materials cost structure of LIB (LMO-LTO) calculated using BatPaC……………………………….…………………………………….….83

**Reference**……………………………………………………………………………84

**Supplementary Fig. 1 Electrochemical behavior of halogen-containing electrolytes.** a, GCD profiles of Zn-I_2_ cells in halogen-containing electrolytes using a coin cell configuration with an iodine mass loading of 2.2 mg cm^-2^; the unusually large apparent capacity originates from corrosion-induced parasitic processes in halogen-containing electrolytes. b, Digital images of battery components after charging in halogen-containing electrolytes. c, Photograph of a customized Swagelok cell used for halogen-containing electrolytes.

In previous studies, the four-electron iodine redox chemistry has traditionally relied on highly concentrated, corrosive halogen-containing electrolytes to stabilize hypervalent iodine cations. However, these electrolytes are highly corrosive, posing significant challenges to battery components. As demonstrated in Supplementary Fig. 1a, Zn-I_2_ cells using halogen-containing electrolytes fail to function properly in a coin cell configuration. Upon disassembly, severe corrosion is observed on Zn negative electrode and battery cases (Supplementary Fig. 1b). Consequently, conventional battery configurations are unsuitable for halogen-containing electrolytes. Electrochemical studies under such conditions typically require the use of specialized Swagelok cell configuration (Supplementary Fig. 1c), which not only increases costs but also limits practical application scenarios.

**Supplementary Fig. 2 Comparison of electrolyte characteristics.** a, Polarization curves of the KA-PA-Nuc electrolyte and a representative highly concentrated, halogen-containing electrolyte (30 m ZnCl_2_). b, Comparison of the electrolyte concentration and cost of reported aqueous metal-I_2_ battery electrolytes.

The electrolyte characteristics were further evaluated. The KA-PA-Nuc electrolyte exhibits substantially reduced corrosivity, enabling stable operation in conventional cell configurations and avoiding the compatibility constraints imposed by corrosive halogen-containing electrolytes (Supplementary Fig. 2a). Comparison with previously reported aqueous metal-I_2_ electrolytes further contextualizes the KA-PA-Nuc electrolyte in terms of electrolyte concentration and cost (Supplementary Fig. 2b). The corresponding references are provided in Supplementary Table 1.

**Supplementary Fig. 3** The correlations and functions of three components in the proposed KA-PA-Nuc electrolyte framework.

**Supplementary Fig. 4 Electrolyte optimization and performance evolution.** a, Cycling stability in electrolytes with different KA concentrations. b, Cycling stability in electrolytes with different PA concentrations. c, Cycling stability in electrolytes with different Nuc concentrations. d, Corresponding GCD profiles in electrolytes with different KA concentrations. e, GCD profiles in electrolytes with different PA concentrations. f, GCD profiles in electrolytes with different Nuc concentrations. All electrochemical measurements were performed using Zn-I_2_ coin cells with an iodine mass loading of 2.2 mg cm^-2^.

As shown in Supplementary Fig. 4, the concentrations of KA, PA, and Nuc were systematically optimized to balance redox reversibility, capacity, and cycling stability. Increasing the KA concentration progressively improves the Coulombic efficiency (CE), whereas the specific capacity and long-term cycling stability initially improve and subsequently decline at higher KA concentrations (Supplementary Fig. 4a, d). This trend suggests that excessive KA may reduce the relative contribution of PA to regulation of the interfacial microenvironment. In contrast, increasing the PA concentration enhances the specific capacity by promoting the I^0^/I^+^ redox process, whereas excessive PA decreases the CE and cycling stability, consistent with a reduced contribution of KA to the regulation of anion-water-Nuc interactions (Supplementary Fig. 4b, e). With the KA and PA concentrations fixed, the influence of Nuc concentration was subsequently examined. The specific capacity, CE and cycling stability initially improve and then reach a plateau with increasing Nuc concentration, consistent with sufficient Nuc availability for halogen bond stabilization during the I^0^/I^+^ conversion (Supplementary Fig. 4c, f). Therefore, through this systematic optimization, the final KA-PA-Nuc electrolyte formulation was selected to provide a balance among redox reversibility, capacity and cycling stability, thereby supporting reversible four-electron iodine conversion.

**Supplementary Fig. 5 Comparison of electrochemical stability windows.** a, Linear sweep voltammetry (LSV) measurements in different electrolytes using urea (U) as Nuc. b, LSV measurements in different electrolytes using acetamide (AE) as Nuc. c, LSV measurements in different electrolytes using nicotinamide (NA) as Nuc. d, LSV measurements in different electrolytes using pyridine (PD) as Nuc. All LSV measurements were performed using a three-electrode configuration consisting of a Ti mesh working electrode, an activated carbon counter electrode, and an Ag/AgCl reference electrode.

The LSV tests were performed using a three-electrode configuration consisting of a Ti mesh working electrode, an activated carbon counter electrode, and an Ag/AgCl reference electrode at a scan rate of 2 mV s^-1^ (Supplementary Fig. 5). For electrolytes containing urea as the Nuc species, the KA-PA-U electrolyte exhibits a hydrogen evolution onset potential below -1.0 V vs. Ag/AgCl and an oxygen evolution onset potential above 1.2 V vs. Ag/AgCl (Supplementary Fig. 5a). Compared with the PA-U and KA-U electrolytes, the KA-PA-U electrolyte therefore provides an electrochemical stability window that encompasses the potential range used for four-electron iodine conversion. Similar electrochemical stability windows are observed when acetamide, nicotinamide and pyridine are used as the Nuc species (Supplementary Fig. 5b-d), indicating that the corresponding KA-PA-Nuc electrolytes are compatible with the potential range employed for four-electron iodine conversion in this study.

**Supplementary Fig. 6** **Molecular electrostatic potential of selected Nuc species.** a, Pyridine (PD). b, Nicotinamide (NA). c, Urea (U). d, Acetamide (AE).

**Halogen bond stabilization mechanism:**

**Supplementary Fig. 7 Electrochemical performance of electrolytes without Nuc species.** a-c, CV profiles of the iodine conversion in PA, KA, and KA-PA electrolytes at various scan rates. d-f, GCD profiles of the iodine conversion in PA, KA, and KA-PA electrolytes at 1 A g^-1^. All electrochemical measurements were performed using Zn-I_2_ coin cells with an iodine mass loading of 2.2 mg cm^-2^.

**Supplementary Fig. 8 Characterization of halogen bonds.** a, 2D ^1^H-^15^N HMBC NMR spectrum of the bare Nuc solution (pyridine). b, 2D ^1^H-^15^N HMBC NMR spectrum of the pyridine solution with iodine. c, 2D ^1^H-^15^N HMBC NMR spectrum of the synthesized I^+^ complexes.

**Supplementary Fig. 9 Theoretical calculation of possible coordination configurations.** a, The A^-^-I⁺-A^-^ coordination structure and corresponding binding energy. b, The Nuc-I⁺-A^-^ coordination structure and corresponding binding energy. c, The Nuc-I⁺-Nuc halogen bond and corresponding binding energy.

**Supplementary Fig. 10 Binding energy and bond length of Nuc-I⁺-Nuc halogen bonds with different counterions.** a, SO_4_^2-^. b, CH_3_COO^-^. c, CF_3_COO^-^. d, CF_3_SO_3_^-^. e, NO_3_^-^. f, ClO_4_^-^.

**Supplementary Fig. 11 Characterization of halogen bonds with different counterions.** a, Schematic illustration of the synthesis of I^+^ halogen bond complexes with different counterions via a cation exchange reaction. 2D ^1^H-^15^N HMBC NMR spectrum of the synthesized I^+^ complexes with different counterions: b, SO_4_^2-^; c, CH_3_COO^-^; d, CF_3_COO^-^; e, CF_3_SO_3_^-^; f, NO_3_^-^; g, ClO_4_^-^.

To substantiate the proposed halogen bond stabilization mechanism, comprehensive electrochemical, spectroscopic, and theoretical investigations were performed. First, control electrolytes without Nuc species show no reduction peak or discharge plateau associated with the I^0^/I^+^ redox process (Supplementary Fig. 7). The absence of I^+^-related electrochemical features across all Nuc-free electrolytes demonstrates that anions alone are insufficient to stabilize I^+^ under these conditions. By contrast, the presence of Nuc species enables successful I^+^ conversion, providing evidence that halogen bond interaction with Nuc species is essential for I^+^ stabilization.

To directly probe halogen bond interaction, 2D ^1^H-^15^N HMBC NMR was employed, which is a sensitive tool for investigation of halogen bond formation. Pyridine was selected as a representative Nuc species. In the bare pyridine solution, coupled H2/H6-N1 and H3/H5-N1 signals appear at 314.78 ppm (Supplementary Fig. 8a). Upon addition of molecular iodine (I_2_), a small ^15^N chemical shift is observed (Δ_15N_ = -22.76 ppm), consistent with weak interaction between pyridine and iodine (Supplementary Fig. 8b). In sharp contrast, when pyridine is exposed to iodonium (I^+^) species generated by an exchange reaction with corresponding silver analogues (Supplementary Fig. 11a), a large ^15^N chemical shift (Δ_15N_ = -107.84 ppm) is detected (Supplementary Fig. 8c). This magnitude of chemical shift is characteristic of halogen bond formation, confirming direct Nuc-I^+^ interaction and providing unambiguous spectroscopic evidence for the halogen bond stabilization mechanism.

To further identify the specific coordination configuration, binding energy calculations were conducted to evaluate possible geometries in our electrolyte system. The results reveal that the linear Nuc-I^+^-Nuc halogen bond, accompanied by a counterion, is the most stable structure, exhibiting lower binding energy than anion-involved configurations (A^-^-I^+^-A^-^ and Nuc-I^+^-A^-^) (Supplementary Fig. 9). These findings indicate that the linear Nuc-I^+^-Nuc halogen bond is thermodynamically preferred, while anions serve as counterions. Moreover, when serving as counterions, different anions have negligible influence on the N-I bond length or binding energy, further supporting their role in halogen bond formation (Supplementary Fig. 10). This observation is fully corroborated by additional 2D ^1^H-^15^N HMBC spectra, where the ¹⁵N chemical shifts of Nuc-I⁺-Nuc complexes remain essentially unchanged across various counterions (Supplementary Fig. 11). Therefore, the preferred coordination configuration in our electrolyte system is the linear Nuc-I⁺-Nuc halogen bond. Collectively, these electrochemical, spectroscopic, and theoretical results provide multiple evidence for the linear Nuc-I⁺-Nuc halogen bond stabilization mechanism underlying the reversible four-electron iodine conversion.

**Supplementary Fig. 12 Analysis of electrochemical products in the PA-Nuc electrolyte.** a, Charging profile of the iodine positive electrode in the PA-Nuc electrolyte. The inset shows the corresponding picture of the charged beaker cell. b, UV-Vis spectra of the PA-Nuc electrolyte at different states of charge. A home-made two-electrode Zn-I_2_ beaker cell with an iodine mass loading of 2.2 mg cm^-2^ was used.

**Supplementary Fig. 13 Electrochemical performance of the AC positive electrode in PA-Nuc electrolytes.** a, GCD profile of the AC positive electrode in the PA-AE electrolyte at 1 A g^-1^. b, GCD profile of the AC positive electrode in the PA-U electrolyte1 A g^-1^. Measurements were performed using Zn-AC coin cells with an AC mass loading of 2.2 mg cm^-2^.

To disclose the nature of the reactions occurring during the second charge plateau, ultraviolet-visible spectroscopy (UV-Vis) was utilized to monitor the electrolyte composition at different states of charge. A home-made beaker cell was assembled to facilitate electrolyte sampling at defined charging states for *ex situ* characterization. As shown in Supplementary Fig. 12a, the charging profile in the beaker cell closely matches that of the coin-cell configuration, confirming comparable electrochemical behavior. At the early charging stages (points A and B), the UV-Vis spectra of the extracted electrolytes show no significant difference compared to the bare electrolyte, suggesting a relatively stable I^-^/I^0^ conversion reaction (Supplementary Fig. 12b). However, as the charging states approaching the second charging plateau, distinct absorption features corresponding to I^-^ and IO_3_^-^ species emerge and intensify, indicating continuous and irreversible oxidation processes involving iodine species.^1^

To further assess whether iodine chemistry alone accounts for the excessive capacity, electrochemical performance of the activated carbon (AC) positive electrode in PA-Nuc electrolytes was investigated (Supplementary Fig. 13). The AC electrode was prepared and tested following identical protocols to the iodine electrode but contained no iodine species. Interestingly, the AC positive electrode also exhibits a prolonged charging plateau at high voltage region in PA-Nuc electrolytes, demonstrating that an additional irreversible process occurs. Given the chemical stability of the AC electrode, this observation strongly suggests electrolyte oxidation under high voltages. This interpretation aligns with electrochemical stability window measurements (Supplementary Fig. 5), which reveal that PA-Nuc electrolytes possess the lowest oxygen evolution potential among the tested systems, attributable to their relatively lower salt concentration. This reduced anodic stability promotes parasitic water oxidation at the voltage range corresponding to the second plateau. Taken together, these findings indicate that the large capacity associated with the second plateau arises from a combination of irreversible oxidation of iodine species and parasitic oxygen evolution reaction originating from electrolyte decomposition.

**Supplementary Fig. 14 Comparison of electrochemical performance using urea as Nuc.** a, Cycling stability in the PA-U electrolyte. b, Cycling stability in the KA-U electrolyte. c, Cycling stability in the KA-PA-U electrolyte. d, GCD profiles in the PA-U electrolyte at different cycles. e, GCD profiles in the KA-U electrolyte at different cycles. f, GCD profiles in the KA-PA-U electrolyte at different cycles. All electrochemical measurements were performed using Zn-I_2_ coin cells with an iodine mass loading of 2.2 mg cm^-2^.

**Supplementary Fig. 15 Comparison of electrochemical performance using acetamide as Nuc.** a, Cycling stability in the PA-AE electrolyte. b, Cycling stability in the KA-AE electrolyte. c, Cycling stability in the KA-PA-AE electrolyte. d, GCD profiles in the PA-AE electrolyte at different cycles. e, GCD profiles in the KA-AE electrolyte at different cycles. f, GCD profiles in the KA-PA-AE electrolyte at different cycles. All electrochemical measurements were performed using Zn-I_2_ coin cells with an iodine mass loading of 2.2 mg cm^-2^.

**Supplementary Fig. 16 Comparison of electrochemical performance using nicotinamide as Nuc.** a, Cycling stability in the PA-NA electrolyte. b, Cycling stability in the KA-NA electrolyte. c, Cycling stability in the KA-PA-NA electrolyte. d, GCD profiles in the PA-NA electrolyte at different cycles. e, GCD profiles in the KA-NA electrolyte at different cycles. f, GCD profiles in the KA-PA-NA electrolyte at different cycles. All electrochemical measurements were performed using Zn-I_2_ coin cells with an iodine mass loading of 2.2 mg cm^-2^.

**Supplementary Fig. 17 Comparison of electrochemical performance using pyridine as Nuc.** a, Cycling stability in the PA-PD electrolyte. b, Cycling stability in the KA-PD electrolyte. c, Cycling stability in the KA-PA-PD electrolyte. d, GCD profiles in the PA-PD electrolyte at different cycles. e, GCD profiles in the KA-PD electrolyte at different cycles. f, GCD profiles in the KA-PA-PD electrolyte at different cycles. All electrochemical measurements were performed using Zn-I_2_ coin cells with an iodine mass loading of 2.2 mg cm^-2^.

**Supplementary Fig. 18 Electrochemical performance comparison of electrolytes with identical ionic strength.** a, CV profiles of iodine conversion using urea as Nuc. b, CV profiles using acetamide as Nuc. c, CV profiles using nicotinamide as Nuc. d-f, Corresponding GCD profiles for a-c at 1 A g^-1^, respectively. All electrochemical measurements were performed using Zn-I_2_ coin cells with an iodine mass loading of 2.2 mg cm^-2^.

**Supplementary Fig. 19 Electrostatic potentials of three anions with similar structures.** a, CH_3_COO^-^. b, CF_3_COO^-^. c, CF_3_SO_3_^-^.

**Supplementary Fig. 20 Theoretical characterization of the bulk properties of the bare ZnSO_4_ (KA) electrolyte.** a, MD snapshot of the bare KA electrolyte. b, Corresponding radial distribution function (RDF) and coordination number profiles of the bare KA electrolyte.

**Supplementary Fig. 21 Theoretical characterization of the bulk properties of the KA electrolyte with different polar anions.** a-c, MD snapshot of the KA electrolyte with CF_3_SO_3_^-^, CF_3_COO^-^, CH_3_COO^-^, respectively. d-e, Corresponding RDF and coordination number profiles of the KA electrolyte with CF_3_SO_3_^-^, CF_3_COO^-^, CH_3_COO^-^, respectively.

**Supplementary Fig. 22** **Theoretical characterization of the bulk properties of the KA-Nuc electrolyte with different polar anions.** a-c, MD snapshot of the KA-Nuc electrolyte with CF_3_SO_3_^-^, CF_3_COO^-^, CH_3_COO^-^, respectively. d-e, Corresponding RDF and coordination number profiles of the KA-Nuc electrolyte with CF_3_SO_3_^-^, CF_3_COO^-^, CH_3_COO^-^, respectively.

**Supplementary Fig. 23 FTIR spectra of different electrolytes.** a, FTIR spectrum of Zn (CF_3_SO_3_)_2_ electrolyte. b, FTIR spectrum of Zn (CF_3_COO)_2_ electrolyte. c, FTIR spectrum of Zn (CH_3_COO)_2_ electrolyte. d, FTIR spectrum of ZnSO_4_ electrolyte.

**Supplementary Fig. 24** **Theoretical characterization of the bulk properties of the highly concentrated** **ZnCl_2_ electrolyte (30 m ZnCl_2_).** a, MD snapshot of the highly concentrated ZnCl_2_ electrolyte. b, Corresponding RDF and coordination number profiles of the highly concentrated ZnCl_2_ electrolyte.

**Supplementary Fig. 25 Theoretical characterization of the interfacial properties of the highly concentrated ZnCl_2_ electrolyte (30 m ZnCl_2_).** a, MD snapshot of the interfacial structure in the highly concentrated ZnCl_2_ electrolyte. b, Corresponding interfacial cumulated density profiles of H_2_O in the highly concentrated ZnCl_2_ electrolyte. c, *In situ* ATR-IR spectra in the highly concentrated ZnCl_2_ electrolyte.

**Supplementary Fig. 26** Differential capacitance curves in electrolytes with different anions. Measurements were performed using Zn-Ti coin cells.

**Supplementary Fig. 27 Evolution of the interfacial environment in KA (SO_4_^2-^) electrolytes with different anions.** a, Differential capacitance curves of KA electrolytes before and after the addition of CH_3_COO^-^. b, Differential capacitance curves of KA electrolytes before and after the addition of CF_3_COO^-^. c, Differential capacitance curves of KA electrolytes before and after the addition of CF_3_SO_3_^-^. d, Schematic illustration of the interfacial evolution in KA electrolytes upon introduction of CH_3_COO^-^. e, Schematic illustration of the interfacial evolution in KA electrolytes upon introduction of CF_3_COO^-^. f, Schematic illustration of the interfacial evolution in KA electrolytes upon introduction of CF_3_SO_3_^-^. Differential capacitance measurements were performed using Zn-Ti coin cells.

**Supplementary Fig. 28 GCD profiles in electrolytes containing different polar anions.** a, GCD profiles in the electrolyte containing CF_3_SO_3_^-^ at different cycles. b, GCD profiles in the electrolyte containing CF_3_COO^-^ at different cycles. c, GCD profiles in the electrolyte containing CH_3_COO^-^ at different cycles. All measurements were performed using Zn-I_2_ coin cells with an iodine mass loading of 2.2 mg cm^-2^.

**Supplementary Fig. 29 Characterization of the spatial distribution of iodine species.** a-c, *In-situ* UV-Vis spectra in electrolytes with CF_3_SO_3_^-^, CF_3_COO^-^, CH_3_COO^-^, respectively. d-f, Corresponding GCD profiles in electrolytes with CF_3_SO_3_^-^, CF_3_COO^-^, CH_3_COO^-^, respectively. Electrochemical measurements were performed using two-electrode Zn-I_2_ cuvette cells with an iodine mass loading of 2.2 mg cm^-2^; photographs of the corresponding cell configurations are shown in the insets.

**Supplementary Fig. 30 Characterization of the spatial distribution of iodine species.** a, X-ray micro-computed tomography (Micro-CT) volume rendering of the charged Zn-I_2_ cell (1.8 V) in the electrolyte with CF_3_SO_3_^-^. b, Micro-CT volume rendering of the charged Zn-I_2_ cell (1.8 V) in the electrolyte with CF_3_COO^-^. c, Micro-CT volume rendering of the charged Zn-I_2_ cell (1.8 V) in the electrolyte with CH_3_COO^-^.

To systematically correlate the spatial localization, electrochemical behavior, and interfacial environment of iodine species in different electrolytes, *in situ* UV-Vis spectra and complementary X-ray Micro-CT characterization were employed. *In situ* UV-Vis spectra were first employed to monitor the evolution of dissolved polyiodide species during cycling, thereby providing indirect evidence regarding the stability and spatial distribution of oxidation products in different electrolytes (Supplementary Fig. 29). The characteristic absorption peak at ~350 nm corresponds to I_3_^-^ species, whose appearance reflects the instability of iodine oxidation products and the migration of iodine species into the bulk electrolyte. In the electrolyte containing CF_3_SO_3_^-^, the I_3_^-^ absorption peak gradually intensifies during cycling, indicating moderate iodine-species diffusion associated with a partially irreversible iodine conversion process (Supplementary Fig. 29a). Consistently, the electrolyte near the electrode surface gradually changes from transparent to light yellow after cycling, further confirming moderate iodine migration (Supplementary Fig. 29d). In the electrolyte containing CF_3_COO^-^, the I_3_^-^ absorption peak increases rapidly with cycling, indicating severe iodine species migration and irreversible iodine conversion (Supplementary Fig. 29b). Correspondingly, the electrolyte becomes uniformly yellow, further confirming extensive iodine diffusion into the bulk electrolyte (Supplementary Fig. 29e). In sharp contrast, in the electrolyte containing CH_3_COO^-^, no discernible I_3_^-^ absorption peak appears during prolonged cycling, while the electrolyte near the electrode surface remains transparent (Supplementary Fig. 29c, f). These observations indicate that the oxidation products are confined within the electrode region and the iodine conversion process is reversible in the electrolyte containing CH_3_COO^-^.

To further directly visualize the spatial distribution of iodine species, X-ray Micro-CT characterization was performed at the fully charged state (1.8 V). The Micro-CT results provide intuitive spatial evidence regarding the localization and redistribution behavior of iodine species. In the electrolyte containing CH_3_COO^-^, high valent iodine species remain confined within the positive electrode region, with no obvious diffusion into the bulk electrolyte or toward the negative electrode side (Supplementary Fig. 30c). In the electrolyte containing CF_3_SO_3_^-^, partial iodine species are observed in the bulk electrolyte and near the negative electrode side, indicating moderate iodine species diffusion (Supplementary Fig. 30a). In sharp contrast, in the electrolyte containing CF_3_COO^-^, a large amount of iodine species is observed throughout the bulk electrolyte and near the negative electrode side, indicating extensive iodine migration and irreversible iodine conversion (Supplementary Fig. 30b). Importantly, these Micro-CT observations are consistent with the *in situ* UV-Vis results.

Notably, the migration tendency of iodine species, as revealed by *in situ* UV-Vis spectra and Micro-CT analyses, follows the order CF_3_COO^-^ ˃ CF_3_SO_3_^-^ ˃ CH_3_COO^-^, reflecting distinct interfacial behaviors among the three electrolytes. Corresponding GCD profiles support this trend. In the electrolyte containing CF_3_SO_3_^-^, only one pair of redox plateaus associated with I^-^/I^0^ couple appears in GCD profiles (Supplementary Fig. 29d), indicating that the I^0^/I^+^ conversion is not activated in this electrolyte environment. Compared with I^0^/I^+^ conversion, I^-^/I^0^ conversion is relatively stable, thereby the overall reaction is relatively reversible and the absorption signal is moderate. In contrast, the electrolyte containing CF_3_COO^-^ activates the I^0^/I^+^ conversion but in an irreversible manner, leading to severe side reactions and extensive iodine species migration (Supplementary Fig. 29e). Notably, the electrolyte containing CH_3_COO^-^ forms an anion-enriched, water-deficient interface that confines the oxidation products in the electrode, thereby enabling reversible four-electron iodine conversion (Supplementary Fig. 29f). These observations are consistent with the electrochemical performance trends, where the electrolyte containing CH_3_COO^-^ shows the highest capacity and the best long-term stability, the electrolyte containing CF_3_SO_3_^-^ exhibits moderate cycling stability but the lowest capacity, and the electrolyte containing CF_3_COO^-^ displays the poorest cycling performance (Fig. 3c). Collectively, these results demonstrate that the spatial distribution, migration behavior, and reversibility of iodine redox species are strongly governed by the interfacial microenvironment determined by the anion chemistry. More importantly, the KA-PA-Nuc electrolyte containing CH_3_COO^-^ establishes an anion-enriched, water-deficient interfacial microenvironment capable of immobilizing and stabilizing high-valent iodine species within the positive electrode region, thereby enabling reversible four-electron iodine conversion chemistry in halogen-free aqueous electrolytes.

**Supplementary Fig. 31 Comparison of hydrogen bond and halogen bond.** a, Charge density difference when the Nuc molecule (taking nicotinamide as an example) connected to H of water. b, Charge density difference when the Nuc molecule connected to I^+^ cation.

**Supplementary Fig. 32 The anion-specific effects and typical anion-water-molecule interactions.** a, The general order of anions in the anion-specific effects with kosmotropic anions on the left side and chaotropic anions on the right side. b, Hydrogen bonds between water molecules and organic molecules (taking nicotinamide as an example) are destabilized through polarization by kosmotropic anions. c, Direct binding of chaotropic anions to the hydrophobic surface of organic molecules. d, Hydrogen bonds between water molecules and organic molecules are facilitated through the direct binding by chaotropic anions.

**Supplementary Fig. 33 Cycling stability in electrolytes with different anions introduced into the same polar anion baseline.** Electrochemical measurements were performed using Zn-I_2_ coin cells with an iodine mass loading of 2.2 mg cm^-2^.

**Supplementary Fig. 34 Electrochemical performance in the electrolyte containing CH_3_COO^-^.** a, Cycling stability in the electrolyte containing 1.5M CH_3_COO^-^. b, GCD profiles in the electrolyte containing 1.5M CH_3_COO^-^ at different cycles. Electrochemical measurements were performed using Zn-I_2_ coin cells with an iodine mass loading of 2.2 mg cm^-2^.

**Supplementary Fig. 35** **Electrochemical performance in the electrolyte containing NO_3_^-^.** a, Cycling stability in the electrolyte containing NO_3_^-^. b, GCD profiles in the electrolyte containing NO_3_^-^ at different cycles. c, 2D ^1^H-^15^N HMBC NMR spectrum of the electrolyte containing NO_3_^-^. Electrochemical measurements were performed using Zn-I_2_ coin cells with an iodine mass loading of 2.2 mg cm^-2^.

**Supplementary Fig. 36 Molecular structure of Nuc species.** a, Molecular structure of the target Nuc molecule with labeled hydrogen atom positions. b, Molecular structure of the target Nuc molecule with labeled carbon atom positions.

**Supplementary Fig. 37** ^13^C NMR spectra of electrolytes containing different anions.

**Supplementary Fig. 38 ^15^N NMR spectra.** a-b, ^15^N NMR spectra of electrolytes containing different anions.

**Supplementary Fig. 39 Solubility test of Nuc species.** a, The solubility of the selected Nuc species in different solutions. b, Corresponding photographs of a.

**Supplementary Fig. 40 Theoretical calculations of water-Nuc interactions in electrolytes with different anions.** a, The radial distribution function of N-H distance between Nuc and water molecules in the presence of different anions. b, Simulated hydrogen bond numbers between Nuc and water molecules in the presence of different anions.

**DRT peak assignments and identification of electrochemical processes**

**Supplementary Fig. 41 In-depth analysis of interfacial electrochemical behaviors.** a, Schematic illustration of the specialized three-electrode configuration, consisting of a working electrode (WE), a counter electrode (CE), and a Zn reference. b, Nyquist plots of two-electrode full cell and individual electrodes. c, Corresponding DRT profiles of two-electrode full cell and individual electrodes. d, Nyquist plot of the Zn negative electrode at 1.78 V. e, Corresponding DRT profile of d. f, Nyquist plot of the iodine positive electrode at 1.78 V vs. Zn/Zn^2+^. g, Corresponding DRT profile of f. h, DRT profiles of the AC positive electrode at different potentials. The iodine loading on the positive electrode was 2.2 mg cm^-2^.

Electrochemical impedance spectroscopy (EIS) is a powerful, non-destructive diagnostic tool that evaluates battery impedance across a wide frequency range, providing valuable information about various electrochemical processes. However, accurately interpreting EIS data can be challenging due to the overlapping frequency responses arising from different electrochemical processes. In this context, the distribution of relaxation time (DRT) technology can deconvolute these overlapping and intertwined electrochemical processes into distinct relaxation processes on different time scales, thereby improving the accuracy of interpretation. Nevertheless, the accurate identification of individual electrochemical processes in a full cell using DRT is still difficult. Full cell comprises a negative electrode and a positive electrode detached by a separator containing electrolyte, where electrochemical processes occur and interact simultaneously at the positive electrode, negative electrode, and multiple interfaces. Such intricate compositions and interactions elevate the challenge of accurate identification of individual electrochemical process, which in turn hinders a precise mechanistic understanding.

To resolve this challenge and enable more reliable peak assignments, we integrated an additional reference electrode into the full cell configuration to establish three-electrode setup. In this setup, a thin zinc wire was placed between the positive electrode and negative electrode (Supplementary Fig. 41a), allowing the decoupling of individual electrode responses, so that the properties of each electrode and their contribution to the overall performance of the cell can be investigated individually. As shown in Nyquist plot (Supplementary Fig. 41b), the impedance of the two-electrode full cell closely matches the sum of two individual electrode impedances, confirming the reliability and accuracy of the reference electrode in decoupling individual electrode contribution of the full cell during *in-situ* EIS measurements. Similarly, the corresponding DRT spectra demonstrates that the DRT profile of the two-electrode full cell is approximately equivalent to the combination of DRT profiles of the individual electrodes, further validating the reliability and accuracy of this methodology (Supplementary Fig. 41c). Notably, both EIS and DRT spectra indicate that the overall impedance of the full cell is predominantly governed by the Zn negative electrode.

To accurately assign each characteristic relaxation process, systematic control experiments and comprehensive analyses were conducted. The Nyquist plots of both negative electrode and positive electrode show a small arc and a pronounced semicircle in the high-frequency region, followed by an oblique line at low frequencies (Supplementary Fig. 41d, f). Typically, the semicircle corresponds to the parallel combination of the charge-transfer resistance (R_ct_) and a capacitive element, whereas the oblique line reflects the ion diffusion process within the electrode. Corresponding DRT spectra reveal six distinct relaxation peaks (τ_1_-τ_6_) across different frequency domains (Supplementary Fig. 41e, g). Among them, τ_1_-τ_4_ appear in the high-frequency region and are associated primarily with charge transfer related processes, while τ_5_-τ_6_ are found in the low-frequency region and correspond to ion transport or diffusion phenomena. Specifically, the τ_1_ peak is located at a very high frequency with a very small characteristic time scale of approximately 10^-^⁵ s. This process is attributed to the intrinsic resistance likely from current collectors, electrode interfaces and electrode particles (R_s_), which is consistent with its fast relaxation time. Moreover, this peak remains stable within different electrolytes across various potentials, further supporting its assignment (Extended Data Fig. 1). The τ_2_ peak, observed at around 10^⁻4^ s, is largely electrolyte-dependent and electrode-independent (Extended Data Fig. 1). It is prominent in electrolytes containing PA (KA (SO_4_^2-^)-PA (CH_3_COO^-^)-Nuc and CF_3_SO_3_^-^-PA (CH_3_COO^-^)-Nuc) but absent in the electrolyte without PA (KA (SO_4_^2-^)-CF_3_SO_3_^-^-Nuc). Given its timescale and electrolyte sensitivity, τ_2_ is assigned to the SEI resistance (R_sei_) at electrode/electrolyte interface. This assignment aligns with theoretical calculations and *in situ* FTIR analyses, both of which reveal the preferential adsorption of PA and the formation of an anion-rich interface that facilitates the four-electron iodine conversion process.

The mid-frequency region manifests two distinct peaks (τ_3_ and τ_4_), which fall within the typical category of charge transfer behavior. The τ_3_ peak appears in both the Zn negative electrode and iodine positive electrode across all potentials, whereas the τ_4_ peak emerges only in the potential range associated with the I^-^/I^0^ and I^0^/I^+^ redox reactions of the iodine positive electrode but persists in the Zn negative electrode over all potentials (Extended Data Fig. 1 and Supplementary Figs. 42-43). Moreover, τ_3_ peak is electrode-dependent, its magnitude varies significantly among the Zn negative electrode, iodine positive electrode, and activated carbon (AC) positive electrode with diverse surface properties. Meanwhile, τ_4_ is completely absent in the AC positive electrode without iodine redox species across all potentials (Supplementary Fig. 41h). During charging, the electrolyte ions migrate toward the electrode surfaces under the applied electric field, overcoming interfacial impedance barriers. Zn²⁺ ions undergo reduction on the negative electrode surface to form metallic zinc, while iodine species undergo oxidation at the positive electrode once the potential reaches the corresponding redox threshold. Based on the observed behavior and correlation with electrochemical processes, τ_3_ is assigned to the interfacial resistance associated with ion migration and surface adsorption, whereas τ_4_ is attributed to the charge-transfer process involving redox species (Zn^2+^ and iodine). At lower frequencies (< 10 Hz), τ₅ and τ₆ appear, corresponding to ion transport and diffusion processes (R_id_). These peaks reflect limitations in mass transport as ions penetrate deeper into the electrode, consistent with their large relaxation time and slower kinetics. As a result, all peaks have been reliably assigned to their corresponding electrochemical processes leveraging this *in-situ* decoupling methodology.

These assignments enable electrolyte chemistry to be correlated with the underlying interfacial electrochemical processes and kinetics. Specifically, the τ_4_ peak of the iodine positive electrode in the CF_3_SO_3_^-^-PA (CH_3_COO^-^)-Nuc and KA (SO_4_^2-^)-CF_3_SO_3_^-^-Nuc electrolytes gradually splits into two distinct peaks within the potential range associated with I^+^ formation, indicating the emergence of competing reactions and incomplete I^+^ conversion (Extended Data Fig. 1b-c, e-f). By contrast, the τ₄ response remains stable in the KA (SO_4_^2-^)-PA (CH_3_COO^-^)-Nuc electrolyte throughout iodine conversion, consistent with suppressed side reactions and reversible iodine redox chemistry (Extended Data Fig. 1a, d). This comparison therefore reveals that PA-mediated interfacial regulation and KA-mediated molecular regulation act cooperatively to maintain stable interfacial electrochemical behaviors during four-electron iodine conversion.

**Supplementary Fig. 42 DRT profiles of the Zn negative electrode in different electrolytes at various charging states**. a, KA (SO_4_^2-^)-PA (CH_3_COO^-^)-Nuc electrolyte. b, CF_3_SO_3_^-^-PA (CH_3_COO^-^)-Nuc electrolyte. c, KA (SO_4_^2-^)-CF_3_SO_3_^-^-Nuc electrolyte. Measurements were performed using the specialized three-electrode configuration shown in Supplementary Fig. 41a.

**Supplementary Fig. 43 *In situ* EIS profiles of the iodine positive electrode in different electrolytes at various charging states**. a, KA (SO_4_^2-^)-PA (CH_3_COO^-^)-Nuc electrolyte. b, CF_3_SO_3_^-^-PA (CH_3_COO^-^)-Nuc electrolyte. c, KA (SO_4_^2-^)-CF_3_SO_3_^-^-Nuc electrolyte. Measurements were performed using the specialized three-electrode configuration shown in Supplementary Fig. 41a.

**Rate-limiting step identification:**

**Supplementary Fig. 44 Identification of the rate-limiting step.** a, Fitted charge transfer resistance (R_ct_) and ion diffusion resistance (R_id_) at different charging rates for the two-electrode full cell (Zn-I_2_). b, Fitted R_ct_ and R_id_ at different charging rates for the individual iodine positive electrode. c, Fitted R_ct_ and R_id_ at different charging rates for the individual Zn negative electrode. d, Demonstration of the fitting process based on DRT results for the two-electrode full cell. e, Demonstration of the fitting process based on DRT results for the individual iodine positive electrode. f, Demonstration of the fitting process based on DRT results for the individual Zn negative electrode. The EIS data were processed using DRT analysis, and the impedance contributions of different electrochemical processes (R_s_, R_sei_, R_ct_, and R_id_) were quantified by integrating the corresponding peak areas.

To identify the rate-limiting step, EIS curves and corresponding DRT plots of the two-electrode full cell, individual iodine positive electrode, and Zn negative electrode under different charging rates were obtained leveraging the *in situ* decoupling methodology. All DRT plots exhibit six distinct relaxation peaks associated with different electrochemical processes: intrinsic ohmic resistance (R_s_-τ_1_), SEI resistance (R_sei_-τ_2_), charge transfer resistance (R_ct_-τ_3-4_), and ion diffusion resistance (R_id_-τ_5-6_). As shown in Supplementary Figs. 45-50, the relative contributions of these four electrochemical processes vary across different voltages and charging rates. To quantitatively assess these variations, the peak areas and corresponding area percentages were fitted and summarized (Supplementary Fig. 44d-f). For the Zn negative electrode, R_ct_ consistently dominates the total impedance across all voltages and charging rates, indicating that Zn deposition is primarily governed by charge transfer kinetics (Supplementary Fig. 44c and Supplementary Figs. 49-50). Although R_id_ slightly increases at higher charging rates due to enhanced mass transport limitations, R_ct_ remains the decisive step controlling the overall reaction kinetics. In contrast, the iodine positive electrode exhibits distinct behavior: R_ct_ dominates within the redox regions, whereas R_id_ becomes more significant in non-redox voltage ranges (Supplementary Fig. 44b and Supplementary Figs. 47-48). Therefore, the iodine conversion is the charge transfer governed electrochemical process. Consequently, for the two-electrode full cell, the overall rate-limiting step can be attributed to charge transfer at both electrodes (Supplementary Fig. 44a and Supplementary Figs. 45-46).

In summary, the *in situ* EIS-DRT analyses identify charge transfer, rather than ion transport, governs the rate-limiting step under both moderate and fast charging conditions. Although mass-transport impedance slightly increases at high rates, its contribution remains secondary due to the intrinsically high ionic conductivity of aqueous electrolyte. Both the iodine conversion at the positive electrode and zinc deposition at the negative electrode involve multi-step electron transfer processes, reinforcing that the overall rate limitation originates from charge-transfer kinetics rather than bulk ion diffusion. This finding provides a mechanistic insight of broad relevance to aqueous systems coupling metal negative electrodes with conversion-type positive electrodes. It suggests that more attention should focus on accelerating interfacial electron transfer rather than solely improving ionic transport. By clearly identifying the rate-limiting step, this work establishes a mechanistic foundation and strategic direction for designing fast-charging aqueous metal batteries.

**Supplementary Fig. 45 *In situ* EIS profiles of the two-electrode full cell at different charging rates**. a, 0.5 A g^-1^. b, 2 A g^-1^. Measurements were performed using the specialized three-electrode configuration shown in Supplementary Fig. 41a.

**Supplementary Fig. 46 Corresponding DRT spectra of the two-electrode full cell at different charging rates**. a, 0.5 A g^-1^. b, 2 A g^-1^.

**Supplementary Fig. 47 *In situ* EIS profiles of the individual iodine positive electrode at different charging rates**. a, 0.5 A g^-1^. b, 2 A g^-1^. Measurements were performed using the specialized three-electrode configuration shown in Supplementary Fig. 41a.

**Supplementary Fig. 48 Corresponding DRT spectra of the individual iodine positive electrode at different charging rates**. a, 0.5 A g^-1^. b, 2 A g^-1^.

**Supplementary Fig. 49 *In situ* EIS profiles of the individual Zn negative electrode at different charging rates**. a, 0.5 A g^-1^. b, 2 A g^-1^. Measurements were performed using the specialized three-electrode configuration shown in Supplementary Fig. 41a.

**Supplementary Fig. 50 Corresponding DRT spectra of the individual Zn negative electrode at different charging rates**. a, 0.5 A g^-1^. b, 2 A g^-1^.

**Supplementary Fig. 51 *In situ* Raman spectroscopy in the electrolyte with Nuc.** a, GCD profiles for *in situ* Raman test. b, *In situ* Raman spectroscopy of the iodine positive electrode in the KA (SO_4_^2-^)-PA (CH_3_COO^-^)-Nuc electrolyte. c, Corresponding contour plots of b. Electrochemical measurements were performed using an in situ two-electrode Zn-I_2_ cell with an iodine mass loading of 2.2 mg cm^-2^.

**Supplementary Fig. 52 *In situ* Raman spectroscopy in the electrolyte without Nuc.** a, *In situ* Raman spectroscopy in the KA-PA electrolyte without Nuc. b, Corresponding contour plots of a.

**Electrochemical performance and techno-economic analysis**

**Supplementary Fig. 53** **Electrochemical performance comparison.** a, CV profiles of Zn-I_2_ cells in the KA-PA-Nuc electrolyte at various scan rates. b, Comparison of rate capability of Zn-I_2_ cells in 30 m ZnCl_2_ and KA-PA-Nuc electrolytes. c, Corresponding GCD profiles in the KA-PA-Nuc electrolyte. Measurements with the KA-PA-Nuc electrolyte were performed using coin cells, whereas those with 30 m ZnCl_2_ were performed using Swagelok cells. The iodine mass loading was 2.2 mg cm^-2^.

**Supplementary Fig. 54 GCD profiles in the 30 m ZnCl_2_ electrolyte at various current densities.** Measurements were performed using Zn-I_2_ Swagelok cells with an iodine mass loading of 2.2 mg cm^-2^.

**Supplementary Fig. 55 Cycling performance of Zn-I_2_ cells in the KA-PA-Nuc electrolyte at 2 A g^-1^.** Measurements were performed using coin cells with an iodine mass loading of 2.2 mg cm^-2^.

**Supplementary Fig. 56 Cycling performance of Zn-I_2_ pouch cell with a small size of 3 cm × 4 cm at a current density of 1A g^-1^.** The iodine mass loading was 2.2 mg cm^-^².

**Supplementary Fig. 57 Electrochemical performances of aqueous Al-I_2_ batteries.** a, CV profiles of aqueous Al-I_2_ cells in the KA-PA-Nuc electrolyte at various scan rates. b, GCD profile of aqueous Al-I_2_ cells using Al-Zn alloy as negative electrodes in the KA-PA-Nuc electrolyte. Electrochemical measurements were performed using coin cells comprising an Al-Zn alloy negative electrode and an iodine positive electrode with an iodine mass loading of 2.2 mg cm^-2^.

**Supplementary Fig. 58 Electrochemical performances of aqueous Zn-I_2_ and Al-I_2_ batteries.** a, Cycling performance of Zn-I_2_ cells using 30 m ZnCl_2_ and KA-PA-Nuc electrolytes at 1 A g^-1^. Measurements with the KA-PA-Nuc electrolyte were performed using coin cells, whereas those with 30 m ZnCl_2_ were performed using Swagelok cells. b, Cycling performance of Al-I_2_ cells using the KA-PA-Nuc electrolyte at 1 A g^-1^. c, Cycling performance of Zn-I_2_ pouch cell, inset shows corresponding digital image. d, Cost structure of 4e ZIB cell using the KA-PA-Nuc electrolyte. e, Cost structure of 4e ZIB cell using the 30 m ZnCl_2_ electrolyte. f, Comparison of cell cost of different batteries. The iodine mass loading was 2.2 mg cm^-2^ for all cell configurations except the pouch cell in c.

Following the elucidation of the synergistic mechanism of the KA-PA-Nuc electrolyte framework, its applicability in four-electron aqueous metal-iodine batteries was investigated. The electrochemical performance was first evaluated in aqueous Zn-I_2_ cells. CV profiles in the KA-PA-Nuc electrolyte retain two pairs of redox features over scan rates from 0.1 to 1 mV s^-1^, consistent with reversible I^-^/I^0^/I^+^ conversion (Supplementary Fig. 53a). The cells using the KA-PA-Nuc electrolyte deliver reversible capacities of 487, 459, 422, 362, 324 and 299 mAh g^-1^ at 0.5, 1, 2, 4, 6 and 8 A g^-1^, respectively (Supplementary Fig. 53b, c). For comparison, cells using 30 m ZnCl_2_ show lower capacities and a larger capacity decline at lower current densities (Supplementary Fig. 53b and Supplementary Fig. 54).

Long-term cycling further confirms sustained four-electron iodine conversion in the KA-PA-Nuc electrolyte. At an iodine loading of 2.2 mg cm^-2^, Zn-I_2_ cells retain 96% of their initial capacity after 1,000 cycles at 1 A g^-1^, compared with 42% for cells using 30 m ZnCl_2_ under the corresponding test conditions (Supplementary Fig. 58a). At 2 A g^-1^, cells using the KA-PA-Nuc electrolyte retain 94% of their capacity after 2,000 cycles (Supplementary Fig. 55). The KA-PA-Nuc electrolyte was further evaluated in Zn-I_2_ pouch-cell configuration, which delivers an initial capacity of 1.1 Ah and retains 0.96 Ah after 600 cycles (Supplementary Fig. 56, 58c). To extend the application of the KA-PA-Nuc electrolyte framework to other aqueous metal-iodine systems, aqueous Al-I_2_ batteries were assembled using Al-Zn alloys as negative electrodes. Al-Zn alloy was selected as the negative electrode owing to its improved stability and reversibility by taking advantage of reversible Al-related alloying/de-alloying chemistry.^2,3^ These cells present two pairs of reversible plateaus associated with four-electron iodine conversion in the KA-PA-Nuc electrolyte (Supplementary Fig. 57). Long-term cycling tests demonstrate a reversible capacity of 327 mAh g^-1^ with a capacity retention of 83% after 1000 cycles at 1 A g^-1^, outperforming the reported four-electron aqueous Al-I_2_ battery that utilized a high-concentration, halogen-containing electrolyte (Supplementary Fig. 58b).^4^ These results demonstrate that the KA-PA-Nuc electrolyte framework supports reversible four-electron iodine conversion across Zn-I_2_ and Al-I_2_ chemistries and different cell configurations.

A techno-economic analysis (TEA) was additionally performed using BatPaC 5.0 to examine the theoretical cell cost potential of different electrolytes under the specified modelling assumptions (Supplementary Fig. 58d-f and Supplementary Tables 3-4).^5^ The model estimates cell costs of 67 USD kWh^-1^ for four-electron aqueous Zn-I_2_ batteries (4e ZIBs) using the KA-PA-Nuc electrolyte and 425 USD kWh^-1^ for the 4e ZIBs using 30 m ZnCl_2_ electrolyte. This difference illustrates the potential for cost reduction enabled by the KA-PA-Nuc electrolyte, primarily through eliminating costly highly concentrated halogen-containing electrolytes and expensive titanium current collectors. A broader cross-chemistry comparison was performed under standardized modelling assumptions, providing a comparable basis for assessing the theoretical cost potential of 4e ZIBs alongside representative Li-ion and K-ion battery chemistries (Supplementary Fig. 58f and Supplementary Tables 3-4). The resulting estimates therefore represent a theoretical cost benchmark rather than immediate competitiveness with established battery technologies. Overall, the TEA indicates the potential cost advantages of 4e ZIBs enabled by the KA-PA-Nuc electrolyte, while identifying further optimization of cell components and validation under practical cell-design conditions will be required to translate this theoretical potential into practical economic feasibility.

**Electrochemical performance at high iodine mass loadings**

**Supplementary Fig. 59 Electrochemical performance of Zn-I_2_ batteries in KA-PA-Nuc electrolytes at high iodine loadings.** a, Rate performance at a high areal iodine loading of 12.2 mg cm^-2^. b, Corresponding GCD profiles of a. c, GCD profiles showing areal capacities at different iodine loadings. d, Cycling performance at an areal iodine loading of 7.6 mg cm^-2^. e, Cycling performance at an areal iodine loading of 12.2 mg cm^-2^. Measurements were performed using coin cells.

The electrochemical performance of Zn-I_2_ batteries using the KA-PA-Nuc electrolyte was further evaluated at increased iodine loadings. Four-electron iodine conversion is retained across the investigated iodine loadings, with the areal capacity increasing to 4.1 mAh cm^-2^ at 12.2 mg cm^-2^ (Supplementary Fig. 59c). At this loading, the cells deliver reversible capacities of 373, 339, 298 and 247 mAh g^-1^ at current densities of 0.5, 1, 2 and 4 A g^-1^, respectively (Supplementary Fig. 59a, b). The influence of iodine loading becomes more evident during extended cycling. At 7.6 mg cm^-2^, the cells deliver a capacity of 336 mAh g^-1^ and retain 89% of their capacity after 1,000 cycles at 1 A g^-1^ (Supplementary Fig. 59d). Increasing the iodine loading to 12.2 mg cm^-2^ results in a capacity of 350 mAh g^-1^ and 72% capacity retention after 800 cycles at 0.5 A g^-1^, accompanied by greater fluctuations in CE (Supplementary Fig. 59e). These results show that the KA-PA-Nuc electrolyte sustains four-electron iodine conversion at increased iodine loadings and areal capacities, while the increased capacity fading and CE fluctuations at higher loadings reveal remaining limitations in long-term cycling stability. The origins of these loading-dependent limitations are examined in the subsequent mechanistic and post-mortem analyses.

**Compatibility of the KA-PA-Nuc electrolyte with commercial current collectors**

**Supplementary Fig. 60 Compatibility of the KA-PA-Nuc electrolyte with commercial current collectors.** a, Rate performance of Zn-I_2_ cells using carbon paper as the current collector. b, Corresponding GCD profiles of Zn-I_2_ cells using carbon paper as the current collector. c, GCD profiles of Zn-I_2_ cells using stainless steel as the current collector. d, Cycling performance of Zn-I_2_ cells using carbon paper as the current collector. e, Rate performance of Zn-I_2_ cells using stainless steel as the current collector. f, Cycling performance of Zn-I_2_ cells using stainless steel as the current collector. The iodine positive electrodes on carbon paper were prepared by PVDF-based slurry coating with an iodine mass loading of 1.5 mg cm^-2^, whereas those on stainless steel were prepared using PTFE-based electrode films with an iodine mass loading of 2.2 mg cm^-2^. Measurements were performed using coin cells.

**Supplementary Fig. 61 Electrochemical behavior of commercial current collectors in halogen-containing electrolytes.** a, GCD profiles of Zn-I_2_ cells using 30 m ZnCl_2_ electrolyte with carbon paper as the current collector in the coin cell configuration. b, GCD profiles of Zn-I_2_ cells using 30 m ZnCl_2_ electrolyte with stainless steel as the current collector in the coin cell configuration. c, GCD profiles of Zn-I_2_ cells using 30 m ZnCl_2_ electrolyte with stainless steel as the current collector in the Swagelok cell configuration. d, GCD profiles of Zn-I_2_ cells using 1.5 ZnCl_2_-0.5 PA-0.4 Nuc electrolyte with carbon paper as the current collector in the coin cell configuration. e, GCD profiles of Zn-I_2_ cells using 1.5 ZnCl_2_-0.5 PA-0.4 Nuc electrolyte with stainless steel as the current collector in the coin cell configuration. f, GCD profiles of Zn-I_2_ cells using 1.5 ZnCl_2_-0.5 PA-0.4 Nuc electrolyte with stainless steel as the current collector in the Swagelok cell configuration. Insets show photographs of the corresponding cycled cells. The unusually large apparent capacity arises from corrosion-induced parasitic processes in halogen-containing electrolytes. The iodine positive electrodes on carbon paper were prepared by PVDF-based slurry coating with an iodine mass loading of 1.5 mg cm^-2^, whereas those on stainless steel were prepared using PTFE-based electrode films with an iodine mass loading of 2.2 mg cm^-2^.

Carbon paper and stainless steel were selected as representative commercial current collectors to examine their compatibility with the KA-PA-Nuc electrolyte. With carbon paper as the positive electrode current collector, the Zn-I_2_ cells using the KA-PA-Nuc electrolyte exhibit reversible four-electron iodine conversion and deliver reversible capacities of 504, 458, 418, 370, 335, and 310 mAh g^-1^ at current densities of 0.5, 1, 2, 4, 6, and 8 A g^-1^, respectively (Supplementary Fig. 60a-b). The cells retain 86% of their capacity after 1,000 cycles at 1 A g^-1^, demonstrating the compatibility of the KA-PA-Nuc electrolyte with carbon-based current collectors (Supplementary Fig. 60d). By comparison, cells using carbon paper in the highly concentrated, halogen-containing electrolyte (30 m ZnCl_2_) fail to complete a full cycle because of continuous corrosion of the cell components (Supplementary Fig. 61a). Corrosion and rapid cell failure are also observed with the halogen-containing 1.5 ZnCl_2_-0.5 PA-0.4 Nuc electrolyte (Supplementary Fig. 61d).

Similar compatibility is observed with stainless steel as the positive electrode current collector. Zn-I_2_ cells using the KA-PA-Nuc electrolyte deliver reversible capacities of 501, 435, 380, 326, 293, and 270 mAh g^-1^ at current densities of 0.5, 1, 2, 4, 6, and 8 A g^-1^, respectively (Supplementary Fig. 60c, e). The cells using the KA-PA-Nuc electrolyte retain 87% of their capacity after 1,000 cycles at a current density of 1 A g^-1^ (Supplementary Fig. 60f). However, cells using stainless steel with either 30 m ZnCl_2_ or 1.5 ZnCl_2_-0.5 PA-0.4 Nuc exhibit pronounced corrosion of the current collector and cell components, preventing stable cycling in both coin cell and Swagelok cell configurations (Supplementary Fig. 61b-c, e-f). Together, these results show that the halogen-free KA-PA-Nuc electrolyte is compatible with both carbon paper and stainless steel current collectors while sustaining four-electron iodine conversion. The comparison with halogen-containing electrolytes further illustrates how electrolyte corrosivity influences the selection of cell components and configurations, providing an additional consideration for the implementation of four-electron aqueous iodine chemistry.

**Mechanistic investigations and post-mortem analysis:**

**Supplementary Fig. 62 Comparison of capacity fading behavior for the coupled I^-^/I^0^/I^+^ conversion reactions at different mass loadings in the KA-PA-Nuc electrolyte.** a, Comparison between the measured capacity retention and the calculated capacity retention at different mass loadings. b, Relative capacity ratio between the discharge capacity and charge capacity of the I^-^/I^0^ plateaus at different mass loadings. c, Schematic illustration of the conversion processes and degradation mechanism. d, Comparison of capacity retention of the decoupled I^0^/I^+^ conversion reaction at different mass loadings; inset shows the cycling performance of the decoupled I^0^/I^+^ conversion reaction at high mass loading. e, Comparison of capacity retention of the decoupled I^-^/I^0^ conversion reaction at different mass loadings; inset shows the cycling performance of the decoupled I^-^/I^0^ conversion reaction at high mass loading. f, X-ray Micro-CT volume rendering of the low mass loading Zn-I_2_ battery after 200 cycles of the coupled I^-^/I^0^/I^+^ conversion reactions. g, X-ray Micro-CT volume rendering of the low mass loading Zn-I_2_ battery after 500 cycles of the coupled I^-^/I^0^/I^+^ conversion reactions. h, X-ray Micro-CT volume rendering of the high mass loading Zn-I_2_ battery after 200 cycles of the coupled I^-^/I^0^/I^+^ conversion reactions. i, X-ray Micro-CT volume rendering of the high mass loading Zn-I_2_ battery after 500 cycles of the coupled I^-^/I^0^/I^+^ conversion reactions. All X-ray Micro-CT images were collected at the end of the charge state of the first redox process. Electrochemical performance measurements were performed using Zn-I_2_ coin cells, whereas Zn-I_2_ Swagelok cells were used for *ex situ* characterization to facilitate cell disassembly and post-cycling sample collection.

**Supplementary Fig. 63 Post-mortem analysis of cycled iodine positive electrodes at high mass loading.** a-d, SEM images of the iodine positive electrodes after 50, 200, 500, and 800 cycles, respectively. a_1_-d_1_, EDS mappings of C element for the iodine positive electrodes after 50, 200, 500, and 800 cycles, respectively. a_2_-d_2_, EDS mappings of I element for the iodine positive electrodes after 50, 200, 500, and 800 cycles, respectively. Electrochemical measurements were performed using Zn-I_2_ coin cells.

**Supplementary Fig. 64 Post-mortem analysis of cycled iodine positive electrodes at low mass loading.** a, SEM image of the pristine iodine positive electrode. b-d, SEM images of the iodine positive electrodes after 10, 40, 80 cycles, respectively. a_1_, EDS mapping of C element for the pristine iodine positive electrode. b_1_-d_1_, EDS mappings of C element for the iodine positive electrodes after 10, 40, 80 cycles, respectively. a_2_, EDS mapping of I element for the pristine iodine positive electrode. b_2_-d_2_, EDS mappings of I element for the iodine positive electrodes after 10, 40, 80 cycles, respectively. Electrochemical measurements were performed using Zn-I_2_ coin cells.

**Supplementary Fig. 65 Post-mortem analysis of cycled iodine positive electrodes at low mass loading after prolonged cycling.** a-c, SEM images of the iodine positive electrodes after 200, 500, and 800 cycles, respectively. a_1_-c_1_, EDS mappings of C element for the iodine positive electrodes after 200, 500, and 800 cycles, respectively. a_2_-c_2_, EDS mappings of I element for the iodine positive electrodes after 200, 500, and 800 cycles, respectively. Electrochemical measurements were performed using Zn-I_2_ coin cells.

**Supplementary Fig. 66 Post-mortem analysis of cycled Zn negative electrodes in high mass loading Zn-I_2_ batteries.** a-d, SEM images of the Zn negative electrodes after 50, 200, 500, and 800 cycles, respectively. e, XRD patterns of the Zn negative electrodes after various cycles. Electrochemical measurements were performed using Zn-I_2_ coin cells.

**Supplementary Fig. 67 Post-mortem analysis of cycled Zn negative electrodes in low mass loading Zn-I_2_ batteries.** a-g, SEM images of the Zn negative electrodes after 0, 10, 40, 80, 200, 500, and 800 cycles, respectively. e, XRD patterns of the Zn negative electrodes after various cycles. Electrochemical measurements were performed using Zn-I_2_ coin cells.

To clarify the origin of the relatively low CE, the mechanism of capacity decay, and the intrinsic relationship between CE and capacity fading, systematic mechanistic investigations and comprehensive post-mortem analysis were conducted. Specifically, the four-electron iodine conversion chemistry was decoupled into three representative processes: the decoupled I^0^/I^+^ conversion, the decoupled I^-^/I^0^ conversion, and the coupled I^-^/I^0^/I^+^ conversion. Their electrochemical behaviors and associated degradation pathways were then systematically analyzed.

For the decoupled I^0^/I^+^ conversion, this reaction shows stable cycling but a relatively low CE of approximately 94% (Extended Data Fig. 2a). The corresponding GCD profiles show that the discharge capacity is lower than the charge capacity, consistent with the observed CE values (Extended Data Fig. 2c). Conventionally, CE is commonly used to estimate battery longevity according to:

$Capacity retention=\prod_{n=1}^{cycle n} {CE}_{cycle n}$	(1)

However, comparison between the measured capacity retention and that calculated from cumulative CE values reveals a substantial discrepancy (Extended Data Fig. 2b). Under the present testing conditions, in which excess electrolyte and Zn negative electrode were used, the dominant source of capacity decay can reasonably be attributed to iodine inventory loss from the positive electrode. The apparent discrepancy between calculated and measured capacity retention indicates that not all apparent CE loss contributes to irreversible capacity fading. In other words, part of the iodine inventory loss is reversible. To further clarify this mechanism, extensive *in situ* and post-mortem analyses of the I^0^/I^+^ conversion process were performed. *In situ* Raman spectra reveal reversible conversion between I^+^ and I^0^ during cycling (Extended Data Fig. 2d). *Ex situ* Raman spectra of cycled separators collected at different discharge states show negligible iodine-related signals and no visible iodine coloration, indicating no obvious iodine migration and supporting the good stability of this conversion process (Extended Data Fig. 2e). Even after extending cycling to 700 cycles, only a weak IO_3_^-^ signal is detected, without obvious I_2_ or I_3_^-^-related features, suggesting that the irreversible capacity loss mainly originates from IO_3_^-^-related side reactions. This interpretation is further supported by the Raman spectrum of extracted positive electrode solution at the charged state of 1.8 V, where characteristic signals of I^0^, I^+^, and IO_3_^-^ are observed (Extended Data Fig. 2e).

Mechanistically, within this voltage window, I_2_ is electrochemically oxidized to I^+^ during charging and reduced back to I_2_ during discharge. However, I^+^ is strongly electrophilic and can undergo hydrolysis in aqueous electrolytes through nucleophilic attack by water molecules:

$5I^{+}+3H_{2}O\to$ ${IO}_{3}^{-}+2I_{2}+6H^{+}$ (2)

During charging, the I_2_ generated through I^+^ hydrolysis remains confined within the positive electrode region and can be further oxidized, thereby contributing additional charge capacity. During discharge, however, hydrolysis of I^+^ partially reduces the discharge capacity within that cycle. Together, these two processes result in relatively low apparent CE. Importantly, I_2_ generated through I^+^ hydrolysis during discharge remains confined within the positive electrode and can participate in subsequent electrochemical cycle. Therefore, this process represents reversible capacity loss rather than irreversible iodine inventory loss. By contrast, IO_3_^-^ remains relatively stable within the applied voltage window and therefore causes irreversible iodine inventory loss. Based on these results, an IO_3_^-^-mediated degradation mechanism is proposed for the decoupled I^0^/I^+^ conversion (Extended Data Fig. 2f). This mechanism explains why the apparent CE is relatively low while the overall cycling stability remains excellent. It further suggests that, if excluding the extra charge capacity associated with hydrolysis generated I_2_ during charging, the intrinsic reversibility of the I^0^/I^+^ process is higher than the apparent CE implies.

We next investigated the decoupled I^-^/I^0^ conversion. This reaction shows good cycling stability and a higher CE of 98-99% (Extended Data Fig. 3a, c). Nevertheless, the calculated capacity retention based on CE still deviates from the measured capacity retention (Extended Data Fig. 3b), again indicating that not all CE loss directly translates into irreversible capacity fading. *In situ* Raman spectra confirm the generation of I_3_^-^ during the I^-^/I^0^ conversion process (Extended Data Fig. 3d). *Ex situ* Raman spectra of separators after prolonged cycling reveal clear I_3_^-^ signals, indicating migration of dissolved I_3_^-^ species into the separator region. At the end of charging, the I_3_^-^ signals become weaker but do not completely disappear, suggesting that most dissolved I_3_^-^ species can return and participate in subsequent electrochemical cycling, whereas a small fraction remains unrecovered and contributes to irreversible iodine inventory loss (Extended Data Fig. 3e). Accordingly, an I_3_^-^-mediated degradation mechanism is proposed for the decoupled I^-^/I^0^ conversion (Extended Data Fig. 3f). In this mechanism, I_3_^-^ formation and dissolution during discharge cause partial discharge capacity loss and prevent CE from reaching 100%, while the recovery of most dissolved I_3_^-^ during subsequent charging accounts for the reversible capacity loss.

Building on these analyses, we further investigated the coupled I^-^/I^0^/I^+^ conversion. Similar to the decoupled systems, the coupled reactions also exhibit a clear discrepancy between measured capacity retention and calculated capacity retention (Extended Data Fig. 4a), confirming that not all apparent CE loss corresponds to irreversible degradation. Interestingly, during the coupled reactions, the discharge capacity of the I^-^/I^0^ plateau exceeds the corresponding charge capacity, indicating the existence of additional discharge capacity contribution (Extended Data Fig. 4b, c). By contrast, the decoupled I^-^/I^0^ conversion shows a lower discharge capacity than charge capacity (Extended Data Fig. 4f). *Ex situ* XPS and Raman spectra confirm reversible I^-^/I^0^/I^+^ conversion processes, whereas I_3_^-^-related signals detected on cycled separators indicate that irreversible iodine inventory loss mainly arises from I_3_^-^ migration (Extended Data Fig. 4d, e). Importantly, in the coupled system, the presence of I^-^ and I_3_^-^ fundamentally changes the fate of hydrolysis generated IO_3_^-^. Specifically, IO_3_^-^ can chemically react with low-valence iodine species such as I^-^：

${IO}_{3}^{-}+5I^{-}+6H^{+}\to$ $3I_{2}+3H_{2}O$ (3)

The generated I_2_ can subsequently participate in electrochemical discharge reactions, thereby contributing additional discharge capacity. Simultaneously, part of I^-^ can be chemically oxidized into I_2_ and re-participate in the electrochemical process. These coupled chemical/electrochemical pathways explain why the discharge capacity of the I^-^/I^0^ plateau exceeds the corresponding charge capacity. Meanwhile, although some IO_3_^-^ may migrate into the electrolyte at high potentials, it can rapidly react with migrated I^-^ or I_3_^-^ to regenerate I_2_ or I_3_^-^, explaining why IO_3_^-^ is not detected on the separator. At lower potentials, the I_3_^-^-mediated degradation pathway remains operative. Therefore, we propose a combined IO_3_^-^/I_3_^-^-mediated degradation mechanism for the coupled I^-^/I^0^/I^+^ conversion (Extended Data Fig. 4g).

This mechanism indicates that the relatively low CE observed in the coupled four-electron iodine conversion arises from multiple coupled processes, including: (i) additional charge capacity associated with I⁺ hydrolysis during charging, (ii) irreversible IO_3_^-^-mediated iodine loss, and (iii) I_3_^-^ dissolution/migration. Simultaneously, IO_3_^-^-related chemical reactions and partial I_3_^-^ recovery help maintain good cycling stability. These findings demonstrate that the apparent low CE does not simply indicate direct irreversible failure but instead reflects a complex interplay between reversible and irreversible iodine inventory processes. This distinction also explains the rate dependence of CE. At lower current densities, longer charge-discharge durations provide more time for parasitic processes, thereby lowering the apparent CE. However, because part of the associated iodine species evolution is recoverable, the decrease in CE does not translate proportionally into irreversible capacity loss. Nevertheless, the increased contribution of parasitic processes at lower rates remains an important limitation.

Critically, whether in the IO_3_^-^-mediated degradation mechanism of the decoupled I^0^/I^+^ reaction, the I_3_^-^-mediated degradation mechanism of the decoupled I^-^/I^0^ reaction, or the combined IO_3_^-^/ I_3_^-^-mediated degradation mechanism of the coupled four-electron reaction, the key prerequisite is that the generated iodine intermediates remain confined within the positive electrode or positive electrode/electrolyte interfacial region, so that they can be reutilized during subsequent electrochemical processes. Stabilization and immobilization of I^+^ species are therefore fundamentally important. Without sufficient stabilization and immobilization of I^+^ species, extensive hydrolysis would produce large quantities of IO_3_^-^ and I_2_, thereby accelerating iodine shuttling, redistribution, and irreversible iodine inventory loss. This is precisely the key role of the KA-PA-Nuc electrolyte framework, which not only confines and stabilizes I^+^ species, but also suppresses iodine species migration and redistribution through the synergistic interplay of anion-specific interfacial chemistry and halogen bond stabilization within an anion-enriched, water-deficient interfacial environment. Notably, these effects mitigate, but do not eliminate the associated parasitic pathways.

We further compared the capacity fading behavior and degradation mechanism under different mass loadings. For the coupled I^-^/I^0^/I^+^ conversion at different mass loadings, all systems exhibit discrepancies between measured and calculated capacity retention, together with larger discharge capacities than charge capacities for the I^-^/I^0^ plateau, indicating that they follow the same combined IO_3_^-^/ I_3_^-^-mediated degradation mechanism (Supplementary Fig. 62a-c). Decoupled analyses further show that the I^0^/I^+^ conversion exhibits excellent cycling stability across different mass loadings, suggesting comparable IO_3_^-^-mediated inventory loss (Supplementary Fig. 62d). In contrast, the decoupled I^-^/I^0^ conversion shows faster capacity decay at high mass loading, indicating more severe I_3_^-^-mediated iodine migration and redistribution (Supplementary Fig. 62e). To directly visualize this process, we performed X-ray Micro-CT imaging of cycled Zn-I_2_ cells with different iodine loadings. The Micro-CT results provide direct spatial evidence of iodine migration and redistribution. At low mass loading, no obvious iodine migration toward the bulk electrolyte or negative electrode side is observed after 200 cycles, and only limited iodine accumulation appears even after 500 cycles (Supplementary Fig. 62f, g). In sharp contrast, at high mass loading, obvious iodine migration into the bulk electrolyte and toward the negative electrode side is observed after 200 cycles, and substantial iodine accumulation appears after 500 cycles (Supplementary Fig. 62h, i). These observations confirm that accelerated capacity fading and noisier CE profiles under high loading conditions originate predominantly from intensified I_3_^-^-mediated migration and redistribution pathways (Supplementary Fig. 59), therefore representing another remaining limitation of iodine conversion chemistry.

Additional post-mortem analyses after prolonged cycling were also conducted. SEM images show that iodine positive electrodes at different mass loadings maintain good structural integrity even after prolonged cycling, indicating that positive electrode structural evolution has negligible contribution to the observed electrochemical degradation and capacity fading (Supplementary Figs. 63-65). XRD analyses of cycled Zn negative electrodes reveal an additional diffraction peak at approximately 7.5°, assigned to Zn_4_SO_4_(OH)_6_·xH_2_O, a common interfacial byproduct in ZnSO_4_-containing electrolytes. Compared with low mass loading Zn-I_2_ cells, the Zn negative electrodes in high mass loading Zn-I_2_ cells show progressively stronger Zn_4_SO_4_(OH)_6_·xH_2_O diffraction peaks with increasing cycling numbers. Consistently, SEM images also reveal rougher Zn surfaces under high mass loading conditions (Supplementary Figs. 66-67). These behaviors originate from two coupled factors. First, compared with low loading systems, high loading Zn-I_2_ cells involve larger areal capacities during cycling, thereby amplifying Zn negative electrode parasitic reactions. Second, under high loading conditions, more severe I_3_^-^ migration and redistribution further accelerate Zn negative electrode corrosion. This interpretation is consistent with the electrochemical results and the Micro-CT visualization of iodine redistribution. Accordingly, a more pronounced Zn-side degradation at higher loading should not be attributed solely to I_3_^-^ migration. Moreover, I_3_^-^ induced Zn degradation represents one pathway associated with irreversible iodine inventory loss, rather than the ultimate failure mode, while migrated iodine species may also become irreversibly retained within the separator or bulk electrolyte without necessarily reaching the Zn negative electrode. Therefore, these comprehensive post-mortem analyses and the systematic mechanistic investigations collectively provide sufficient and compelling evidence to elucidate the origin of low CE and the fundamental mechanism of capacity decay.

Overall, these mechanistic investigations and post-mortem analyses demonstrate that: (i) the relatively apparent low CE in four-electron iodine conversion does not simply originate from irreversible loss, but rather from coupled reversible and irreversible iodine inventory processes; (ii) capacity fading mainly arises from parasitic reactions involving both high-valent iodine species and triiodide intermediates; (iii) immobilization and stabilization of iodine intermediates within the positive electrode or interfacial region are fundamentally critical for achieving reversibility and stability; (iv) the critical role of the KA-PA-Nuc electrolyte framework in immobilizing and stabilizing iodine intermediates within anion-enriched, water-deficient interfacial environment through the synergistic interplay of anion-specific interfacial chemistry and halogen bond stabilization.

These findings also provide several important directions for future development of four-electron iodine conversion chemistry. First, future studies should move beyond simply activating and stabilizing I^+^ species and instead establish comprehensive iodine inventory management frameworks that effectively track the generation, migration, redistribution, and consumption of iodine intermediates. Correspondingly, more advanced operando and spatially resolved characterization techniques will become increasingly important for directly recognizing and tracking different iodine intermediates. Second, future efforts should simultaneously improve the stability of both the I^0^/I^+^ and I^-^/I^0^ conversion reactions, rather than focusing solely on high-valent iodine activation and stabilization. In this context, further strategies for enhancing the immobilization or stabilization of iodine intermediates, including I^+^, IO_3_^-^, and I_3_^-^ species, will be critical for minimizing irreversible iodine inventory loss, especially under practically high mass loading conditions. Third, at the cell level, strategies should be integrated with positive electrode confinement, separator engineering and negative electrode protection to suppress irreversible iodine redistribution. More broadly, the present results indicate that further advances in four-electron aqueous iodine chemistry will require coordinated regulation across molecular, interfacial and cell levels, rather than focusing solely on the activation and stabilization of high-valent iodine species. We believe that the mechanistic insights established in this work not only clarify the origin of low CE and capacity fading in four-electron iodine conversion, but also provide a basis for understanding and addressing the remaining limitations of reversible multielectron aqueous halogen chemistry.

**Supplementary Table 1** Comparison of aqueous electrolytes for multielectron iodine redox reactions

| Number | Cathode | Electrolyte | Concentration (mol/L) | Electrolyte cost (US$/kg) | Conversion reaction | Halogen | Battery chemistry | Ref. |
| --- | --- | --- | --- | --- | --- | --- | --- | --- |
| 1 | Ti_3_C_2_I_2_ MXene | ZnCl_2_-KCl | 3 | 28.5 | I^−^/I^0^/I^+^ | Cl^-^ | Zn-I_2_ | Energy Environ. Sci., 2021, 14, 407-413^6^ |
| 2 | Activated porous corncob carbon-I_2_ | ZnCl_2_ | 5.9 | 70.8 | I^−^/I^0^/I^+^ | Cl^-^ | Zn-I_2_ | J. Power Sources, 580, 2023, 233296^7^ |
| 3 | AC-I_2_ | ZnCl_2_ | 30 | 359.9 | I^−^/I^0^/I^+^, Cl^-^/Cl^0^ | Cl^-^ | Zn-I_2_ | Nat. Commun., 2023, 14, 1856^8^ |
| 4 | Reduced graphene oxide fiber | ZnCl_2_-methyl ammonium iodide | 21 | 291.6 | I^−^/I^0^/I^+^ | Cl^-^, I^-^ | Zn-I_2_ | Nanoscale, 2024, 16, 6596-6602^9^ |
| 5 | PVP-I | Znotf-urea | 10 | 540.3 | I^−^/I^0^/I^+^ | -- | Zn-I_2_ | ACS Nano, 2025, 19 (2), 2633-2640^10^ |
| 6 | Fe SAC-MNC-I_2_ | ZnCl_2_-LiCl-Acetonitrile | 46 | 444.3 | I^−^/I^0^/I^+^ | Cl^-^ | Zn-I_2_ | Nano-Micro Lett., 2023, 15, 126^11^ |
| 7 | Co_9_S_8_@NC | ZnCl_2_ | 7.5 | 90.0 | I^−^/I^0^/I^+^ | Cl^-^ | Zn-I_2_ | Adv. Mater., 2024, 36, 2312246^12^ |
| 8 | AC-I_2_ | ZnCl_2_-TEACl | 16 | 188.1 | I^−^/I^0^/I^+^ | Cl^-^ | Zn-I_2_ | J. Am. Chem. Soc., 2024, 146, 21377-21388^13^ |
| 9 | Iodobenzene | ZnCl_2_ | 10 | 120.0 | I^+^/I^3+^ | Cl^-^ | Zn-I_2_ | J. Am. Chem. Soc., 2024, 146, 25108-25117^14^ |
| 10 | Carbon cloth-I_2_ | LiCl-LiNO_3_ | 22 | 237.9 | I^0^/I^+^ | Cl^-^ | S-I_2_ | Adv. Funct. Mater., 2023, 33, 2212644^15^ |
| 11 | AC-I_2_ | ZnCl_2_-Liotf | 18 | 1023.7 | I^−^/I^0^/I^+^ | Cl^-^ | Zn-I_2_ | Chem. Commun., 2024,  60, 7447^16^ |
| 12 | FeSA-NC-I_2_ | ZnCl_2_-PEG | 6 | 32.7 | I^−^/I^0^/I^+^ | Cl^-^ | Zn-I_2_ | Adv. Mater., 2024, 2405473^17^ |
| 13 | PAC-I_2_ | ZnCl_2_-LiCl-Acetonitrile | 46 | 444.3 | I^−^/I^0^/I^+^ | Cl^-^ | Zn-I_2_ | Nat. Commun., 2021, 12, 170^18^ |
| 14 | Quaternary  ammonium dichloroiodate | ZnSO_4_- ZnCl_2_ | 4 | 23.2 | I^−^/I^0^/I^+^ | Cl^-^ | Zn-I_2_ | Chem. Sci., 2024, 15, 3357^19^ |
| 15 | Fe_SA_-NC-I_2_ | ZnCl_2_-Acetonitrile | 18 | 128.5 | I^−^/I^0^/I^+^ | Cl^-^ | Zn-I_2_ | Sci. Bull., 2024, 69, 1674-1685^20^ |
| 16 | AC-I_2_ | DMSO–Zn (ClO_4_)_2_.6H_2_O–NA | 15 | 120 | I^−^/I^0^/I^+^ | -- | Zn-I_2_ | Energy Environ. Sci., 2023, 16, 4502^21^ |
| 17 | AC-I_2_ | ZnSO_4_-LiBr-LiNO_3_ | 6.6 | 37.8 | I^−^/I^0^/I^+^, Br^-^/Br^0^ | Br^-^ | Zn-I_2_ | ACS Nano, 2022, 16, 20389-20399^22^ |
| 18 | I_2_@ZIF-8-C | AlCl_3_-LiTFSI | 10 | 1470.8 | I^−^/I^0^/I^+^ | Cl^-^ | Al-I_2_ | Small Methods, 2021, 5, 2100611^4^ |
| 19 | AC-I_2_ | AlCl_3_-ChCl | 4 | 67.2 | I^−^/I^0^/I^+^ | Cl^-^ | Al-I_2_ | Joule, 2026, 10, 102502^23^ |
| 20 | AC-I_2_ | ZnCl_2_-EG | 17 | 51.9 | I^−^/I^0^/I^+^ | Cl^-^ | Zn-I_2_ | Adv. Funct. Mater., 2026, 36, e75378^24^ |
| 21 | Co-SAs@NC-I_2_ | ZnCl_2_ | 15 | 179.9 | I^−^/I^0^/I^+^ | Cl^-^ | Zn-I_2_ | Angew. Chem. Int. Ed., 2026, 0, e9322658^25^ |
| 22 | AC-I_2_ | ZnSO_4_-BHDACl | 2.5 | 527.4 | I^−^/I^0^/I^+^ | Cl^-^ | Zn-I_2_ | ACS Nano, 2026, 20, 23464-23476^26^ |
| 23 | AC-I_2_ | Zn (ClO_4_)_2_-GuHCl | 3.5 | 983.5 | I^−^/I^0^/I^+^ | Cl^-^ | Zn-I_2_ | ACS Energy Lett., 2026, 11, 6361-6370^27^ |
| 24 | AC-I_2_ | ZnSO_4_-MImCl | 2.2 | 21.9 | I^−^/I^0^/I^+^ | Cl^-^ | Zn-I_2_ | J. Am. Chem. Soc., 2026, 148, 34958-34968^28^ |
| 25 | AC-I_2_ | ZnSO_4_-EMImBr | 3 | 61.7 | I^−^/I^0^/I^+^ | Br^-^ | Zn-I_2_ | ACS Nano, 2026, 20, 16180-16190^29^ |
| 26 | AC-C_18_I_3_ | ZnSO_4_-ZnCl_2_ | 3.2 | 13.6 | I^−^/I^0^/I^+^ | Cl^-^ | Zn-I_2_ | Angew. Chem. Int. Ed., 2026, 0, e8492354^30^ |
| 27 | AC-I_2_ | ZnSO_4_-ChCl-TES-Na | 7.5 | 343.5 | I^−^/I^0^/I^+^ | Cl^-^ | Zn-I_2_ | Adv. Mater., 2026, 38, e73681^31^ |
| 28 | AC-I_2_ | ZnSO_4_-NH_4_Cl | 2.5 | 13.1 | I^−^/I^0^/I^+^ | Cl^-^ | Zn-I_2_ | Adv. Mater., 2026, 38, e73355^32^ |
| 29 | AC-I_2_ | Zn (ClO_4_)_2_-ZnCl_2_-Gly-PVA | 4.6 | 502.4 | I^−^/I^0^/I^+^ | Cl^-^ | Zn-I_2_ | Angew. Chem. Int. Ed., 2025, 137, e202514375^33^ |
| 30 | AC-I_2_ | KA-PA-Nuc | 2.5 | 17.5 | I^−^/I^0^/I^+^ | -- | Al-I_2_ | This work |
|  |  |  | 2.4 | 9.4 | I^−^/I^0^/I^+^ | -- | Zn-I_2_ |  |

Price information on electrolyte components was mainly taken form <https://www.aladdin-e.com> (a major reagent supplier). Electrolyte concentration and price were calculated based on all components in each electrolyte, including salts, additives, and solvents (excepting water).

**Supplementary Table 2** Comparison of electrochemical performances in various electrolytes for multielectron iodine redox reactions

| Number | Electrolyte | Current density (A/g)/specific capacity (mAh/g)/ cycle number/capacity retention (%)/capacity decay per cycle (%) | I^0^/I^+^ capacity contribution (%) | Battery chemistry | Ref. |
| --- | --- | --- | --- | --- | --- |
| 1 | 2 ZnCl_2_-1 KCl | 0.5/207/--/--  3/160/2800/80/0.0072 | 52.0  -- | Zn-I_2_ | Energy Environ. Sci., 2021, 14, 407-413^6^ |
| 2 | 5.9 ZnCl_2_ | 0.24/422/100/95/0.05 | ~41.7 | Zn-I_2_ | J. Power Sources‌, 580, 2023, 233296^7^ |
| 3 | 30 ZnCl_2_ | 2/470/2000/95.7/0.0022 | ~32.6 | Zn-I_2_ | Nat. Commun., 2023, 14, 1856^8^ |
| 4 | 20 ZnCl_2_-1 methyl ammonium iodide | --/--/--/--/-- | -- | Zn-I_2_ | Nanoscale, 2024, 16, 6596-6602^9^ |
| 5 | 4 Znotf-6 urea | 1.3/219/700/91.3/0.0125  2.2/285/10000/51.6/0.0049 | ~35.1  ~31.6 | Zn-I_2_ | ACS Nano, 2025, 19 (2), 2633-2640^10^ |
| 6 | 19 ZnCl_2_-19 LiCl-8 Acetonitrile | 1/398/500/56/0.088 | ~41.5 | Zn-I_2_ | Nano-Micro Lett., 2023, 15:126^11^ |
| 7 | 7.5 ZnCl_2_ | 1/405/250/63.2/0.1472  5/300/5000/60.1/0.008 | ~26.2  ~21.3 | Zn-I_2_ | Adv. Mater., 2024, 36, 2312246^12^ |
| 8 | 15 ZnCl_2_-1 TEACl | 0.5/425/200/86.9/0.0655  10/300/35000/47.5/0.0015 | ~46.5  ~38.7 | Zn-I_2_ | J. Am. Chem. Soc., 2024, 146, 21377-21388^13^ |
| 9 | 10 ZnCl_2_ | 0.5/422/50/50/1 | -- | Zn-I_2_ | J. Am. Chem. Soc., 2024, 146, 25108-25117^14^ |
| 10 | 15 LiCl-7 LiNO_3_ | 1/243/300/86/0.047 | -- | S-I_2_ | Adv. Funct. Mater., 2023, 33, 2212644^15^ |
| 11 | 2 ZnCl_2_-16 Liotf | 1/425/1000/98.8/0.0012  2/400/10000/83.6/0.0017 | ~45.0  ~36.8 | Zn-I_2_ | Chem. Commun., 2024,  60, 7447^16^ |
| 12 | 2 ZnCl_2_-70% PEG | 0.4/524.7/1500/97.6/0.0016  2/507.9/20000/81.5/0.00093 | ~47.6  ~43.3 | Zn-I_2_ | Adv. Mater., 2024, 2405473^17^ |
| 13 | 19 ZnCl_2_-19 LiCl-8 Acetonitrile | 0.8/600/1000/85.4/0.0146  2/500/6000/84.0/0.0027 | ~42.4  -- | Zn-I_2_ | Nat. Commun., 2021, 12, 170^18^ |
| 14 | 3 ZnSO_4_-1 ZnCl_2_ | 2.1/405/1200/75.0/0.0208 | ~39.5 | Zn-I_2_ | Chem. Sci., 2024, 15, 3357^19^ |
| 15 | 10 ZnCl_2_-8 Acetonitrile | 1/490.9/1000/93.6/0.0064  2/470.5/10000/80.0/0.002 | ~43.1  ~42.1 | Zn-I_2_ | Sci. Bull., 2024, 69, 1674-1685^20^ |
| 16 | DMSO-Zn (ClO_4_)_2_.6H_2_O-NA | 2/380/2000/80/0.01 | ~46.1  ~42.6 | Zn-I_2_ | Energy Environ. Sci., 2023, 16, 4502^21^ |
| 17 | 3 ZnSO_4_-3.5 LiBr-0.1 LiNO_3_ | --/635/500/67/0.066 | -- | Zn-I_2_ | ACS Nano, 2022, 16, 20389-20399^22^ |
| 18 | 1 AlCl_3_-9 LiTFSI | 2/259/150/62.5/0.25 | ~38.6 | Al-I_2_ | Small Methods, 2021, 5, 2100611^4^ |
| 19 | 3 AlCl_3_-1 ChCl | 0.5/340/200/96/0.02  2/300/1100/56/0.04 | ~38.5 | Al-I_2_ | Joule, 2026, 10, 102502^23^ |
| 20 | 1 ZnCl_2_-EG | 1/386/5000/77/0.0046 | ~46.5 | Zn-I_2_ | Adv. Funct. Mater., 2026, 36, e75378^24^ |
| 21 | 15 ZnCl_2_ | 1/404.1/400/81.8/0.0455 | ~44.5 | Zn-I_2_ | Angew. Chem. Int. Ed., 2026, 0, e9322658^25^ |
| 22 | 2 ZnSO_4_-0.5 BHDACl | 0.8/374.7/2000/88.1/0.006 | ~45 | Zn-I_2_ | ACS Nano, 2026, 20, 23464-23476^26^ |
| 23 | 3 Zn (ClO_4_)_2_-0.5 GuHCl | 0.4/280/4000/64.3/0.009 | ~50 | Zn-I_2_ | ACS Energy Lett., 2026, 11, 6361-6370^27^ |
| 24 | 2 ZnSO_4_-0.2 MImCl | 0.8/400/3000/95/0.002 | ~48.8 | Zn-I_2_ | J. Am. Chem. Soc., 2026, 148, 34958-34968^28^ |
| 25 | 2 ZnSO_4_-1 EMImBr | 1/300/2000/70/0.015 | ~49 | Zn-I_2_ | ACS Nano, 2026, 20, 16180-16190^29^ |
| 26 | 3 ZnSO_4_-0.2 ZnCl_2_ | 0.5/463/8500/78.4/0.003 | ~38.9 | Zn-I_2_ | Angew. Chem. Int. Ed., 2026, 0, e8492354^30^ |
| 27 | 2 ZnSO_4_-5 ChCl-0.5 TES-Na | 5/333/2500/93/0.0028 | ~47.6 | Zn-I_2_ | Adv. Mater., 2026, 38, e73681^31^ |
| 28 | 2 ZnSO_4_-0.5 NH_4_Cl | 10/228/2000/88/0.006 | ~43.4 | Zn-I_2_ | Adv. Mater., 2026, 38, e73355^32^ |
| 29 | 1.5 Zn (ClO_4_)_2_-1 ZnCl_2_-15% Gly-0.1 PVA | 2/402.46/1000/88.27/0.012 | ~45.4 | Zn-I_2_ | Angew. Chem. Int. Ed., 2025, 137, e202514375^33^ |
| 30 | 1.5KA-0.5PA-0.5Nuc | 1/325/1000/83/0.017 | 39.4 | Al-I_2_ | This work |
|  | 1.5KA-0.5PA-0.4Nuc | 1/451/1000/96.0/0.004  2/407/2000/94.0/0.003 | 47.8  42.9 | Zn-I_2_ |  |

**Techno-economic model analysis**

The techno-economic analysis was performed using BatPaC 5.0 modelling software.^5^ This model calculates the energy density, specific energy, and cost of a battery cell or pack based on the specific capacity and electrochemical properties of the positive electrode and negative electrode active materials. Additionally, it incorporates bulk density and cost data for active materials, carbon, binder, solvent, electrolyte, current collectors, and other essential components. The model assumes a typical multilayer component stack, where electrode layers are double-sidedly coated onto current collector foils and interleaved with porous separators and electrolyte to form a complete cell. Multiple cells are then interconnected in series and parallel configurations to achieve the required pack voltage, energy capacity, and power output.

The present analysis was used as an illustrative theoretical assessment under standardized modelling assumptions rather than as a cost estimate for the experimentally tested cells. Two comparisons were considered. First, four-electron Zn-I_2_ batteries (4e ZIBs) using the KA-PA-Nuc and 30 m ZnCl_2_ electrolytes were modelled within the same framework to examine the material-cost implications associated with the different electrolyte chemistries. Second, a broader cross-chemistry comparison was performed to provide a theoretical cost benchmark for 4e ZIBs relative to representative Li-ion and K-ion battery chemistries under standardized cell-design assumptions. These calculations are not intended to represent experimentally demonstrated economics or immediate economic or technological competitiveness with established battery technologies.

The data for the K-ion battery (KIB) was obtained from previous publication, where the calculations were also performed using the BatPaC modeling software.^34^ For comparison, data for five commonly used Li-ion batteries (LIBs) chemistries: LiMn_2_O_4_- Li_4_Ti_5_O_12_ (LMO-LTO), LiCoO_2_-Graphite (LCO-G), Li(Ni_0.8_Mn_0.1_Co_0.1_)O_2_-Graphite (NMC811-G), LiFePO_4_-Graphite (LFP-G), and LiNi_0.8_Co_0.15_Al_0.05_O_2_-Graphite (NCA-G), was obtained from BatPaC 5.0 modelling software. These LIBs were selected for comparison as they represent the most widely commercialized battery chemistries.

Taking LMO-LTO as an example, the parameters used for calculation are shown in Supplementary Table 3. The model assumes a typical multilayer component stack, where electrode layers are double-sidedly coated onto current collector foils and interleaved with porous separators and electrolytes. Both the negative electrode and positive electrode contain carbon additive and binder, while the negative electrode, positive electrode, and separator include a void fraction. Aluminum foil serves as the current collector for both the positive electrode and negative electrode, with a thickness of 20 µm and a cost of 0.20 USD m^-2^. The binder, carbon additive, and solvent used in both electrodes are identical, with costs of 15.00, 7.00, and 2.70 USD kg^-1^ for PVDF binder, conductive carbon, and NMP solvent, respectively. The positive electrode has a specific capacity of 118.60 mAh g^-1^ and a cost of 8.00 USD kg^-1^, while the negative electrode has a specific capacity of 165.00 mAh g^-1^ and a cost of 15.00 USD kg^-1^. All these parameters were taken from the BatPaC 5.0 modelling software. Based on these inputs, the calculated material cost structure for the cell is provided in Supplementary Table 4, where the positive electrode and negative electrode contribute the most to the total material costs.

**Supplementary Table 3** Cost inputs of different batteries for techno-economic model analysis using BatPaC 5.0 modelling software

|  | **LIB**  **(LMO-LTO)** | **4e ZIB**  **(30 m ZnCl_2_)** | **4e ZIB**  **(KA-PA-Nuc)** |
| --- | --- | --- | --- |
| **(1) Positive electrode** | | | |
| Material | LiMn_2_O_4_ | I_2_ | I_2_ |
| Specific capacity (mAh g^-1^) | 118.60 | 422.00 | 422.00 |
| Positive electrode binder | PVDF | PTFE | PTFE |
| Positive electrode additive | Conductive carbon | Conductive carbon | Conductive carbon |
| Solvent | NMP | Water | Water |
| Positive electrode foil material | Aluminum | Titanium | Stainless steel |
| **(2) Negative electrode** | | | |
| Material | Li_4_Ti_5_O_12_ | Zn | Zn |
| Specific capacity (mAh g^-1^) | 165.00 | 819.00 | 819.00 |
| Negative-to-positive capacity ratio | 1.02 | 2.00 | 2.00 |
| Negative electrode foil material | Aluminum | -- | -- |
| Negative electrode binder | PVDF | -- | -- |
| Solvent | NMP | -- | -- |
| **(3) Separator** | | | |
| Separator thickness (µm) | 15.00 | 40.00 | 40.00 |
| **(4) Electrolyte** | | | |
| Electrolyte composition | 1.2 M LiPF_6_ in EC/EMC | 30 M ZnCl_2_ in water | 1.5KA-0.5PA-0.4Nuc in water |
| Electrolyte density (g cm^-3^) | 1.20 | 2.33 | 1.27 |
| **（5）Material costs** | | | |
| Price of active material for positive electrode (USD kg^-1^) | 8.00 | 0.80 | 0.80 |
| Price of carbon additive for positive electrode (USD kg^-1^) | 7.00 | 7.00 | 7.00 |
| Price of binder for positive electrode (USD kg^-1^) | 15.00 | 10.00 | 10.00 |
| Price of solvent for positive electrode (USD kg^-1^) | 2.70 | 0.00 | 0.00 |
| Price of active material for negative electrode (USD kg^-1^) | 15.00 | 2.36 | 2.36 |
| Price of carbon additive for negative electrode (USD kg^-1^) | 7.00 | -- | -- |
| Price of binder for negative electrode (USD kg^-1^) | 15.00 | -- | -- |
| Price of solvent for negative electrode (USD kg^-1^) | 2.70 | -- | -- |
| Price of positive electrode current collector (USD m^-2^) | 0.20 | 13.15 | 1.22 |
| Price of negative electrode current collector (USD m^-2^) | 0.20 | -- | -- |
| Price of separator (USD m^-2^) | 0.90 | 0.38 | 0.38 |
| Price of electrolyte (USD L^-1^) | 9.00 | 359.90 | 9.40 |

**Supplementary Table 4** Cell materials cost structure of LIB (LMO-LTO) calculated using BatPaC

| Component | Positive electrode | Negative electrode | Electrolyte | Current collector | Others |
| --- | --- | --- | --- | --- | --- |
| Proportion (%) | 32.55 | 42.41 | 9.57 | 2.75 | 12.72 |

The 4e ZIBs were modelled using the same BatPaC framework. The model assumes a multilayer component stack, where the positive electrode consists of a double-sided coating of active material, carbon additive, and binder on a current collector foil, interleaved with a porous separator and electrolyte. Zn foil was used as negative electrode directly without any current collector, conductive carbon, and binder. For this theoretical model, a negative-to-positive capacity ratio (N/P ratio) of 2.00 was assumed to explore the cost potential under a hypothetical cell design with reduced excess Zn. This value is a modelling assumption and does not correspond to the electrode balancing used in the laboratory cells investigated in this study. All solvents used for electrode fabrication and electrolyte preparation were water. All parameters for solvent, carbon additive, and binder were taken from BatPaC 5.0 modelling software. The price of the Zn negative electrode, separator, and stainless steel current collector was obtained from previous studies conducted by our group, while the cost of the positive electrode materials and current collectors was estimated based on market prices (https://alibaba.com).^35^ The treatment of the positive electrode in this model is simplified. The density of electrolytes was calculated based on measurements. Electrolyte density was determined through direct measurements. Given the high corrosiveness of halogen-based electrolytes, titanium foil was selected as the current collector for a 4e ZIB using the 30 m ZnCl_2_ electrolyte. In contrast, stainless steel foil was used as the current collector in a 4e ZIB with the KA-PA-Nuc electrolyte due to its halogen-free nature and improved compatibility.

**Reference**

1	Ma, W., Li, J., Wang, H., Lei, C. & Liang, X. High efficiency alkaline iodine batteries with multi‐electron transfer enabled by Bi/Bi_2_O_3_ redox mediator. *Angew. Chem. Int. Ed.,* **63**, e202410994 (2024).

2	Dou, Q. *et al.* Unveiling solvation structure and desolvation dynamics of hybrid electrolytes for ultralong cyclability and facile kinetics of Zn-Al alloy anodes. *Energy Environ. Sci.,* **15**, 4572-4583 (2022).

3	Liu, Y. *et al.* Solvation-structure design of multicomponent eutectic electrolytes enabling Al-rich alloy growth in aqueous aluminum-ion batteries. *J. Am. Chem. Soc.,* **148**, 1220-1232 (2026).

4	Yang, S. *et al.* High-rate aqueous aluminum-ion batteries enabled by confined iodine conversion chemistry. *Small Methods,* **5**, e2100611 (2021).

5	Knehr, K. W., Kubal, J. J., Nelson, P. A. & Ahmed, S. Battery performance and cost modeling for electric-drive vehicles (a manual for BatPaC v5. 0). (Argonne National Lab.(ANL), Argonne, IL (United States), 2022).

6	Li, X. *et al.* Activating the I^0^/I^+^ redox couple in an aqueous I_2_-Zn battery to achieve a high voltage plateau. *Energy Environ. Sci.,* **14**, 407-413 (2021).

7	Han, W. & Li, X. Electrode and electrolyte additive synergy effect for improving the capacity and supporting a high voltage plateau of aqueous rechargeable zinc iodine batteries. *J. Power Sources,* **580** (2023).

8	Liang, G. *et al.* Development of rechargeable high-energy hybrid zinc-iodine aqueous batteries exploiting reversible chlorine-based redox reaction. *Nat. Commun.,* **14**, 1856 (2023).

9	Wang, J. *et al.* Rational electrolyte design and electrode regulation for boosting high-capacity Zn-iodine fiber-shaped batteries with four-electron redox reactions. *Nanoscale,* **16**, 6596-6602 (2024).

10	Li, C. *et al.* Urea chelation of I^+^ for high-voltage aqueous zinc-iodine batteries. *ACS Nano,* **19**, 2633-2640 (2025).

11	Yang, X. *et al.* Aqueous zinc batteries with ultra-fast redox kinetics and high iodine utilization enabled by iron single atom catalysts. *Nano-Micro Lett.,* **15**, 126 (2023).

12	Hu, T. *et al.* Development of inverse-opal-structured charge-deficient Co_9_S_8_@nitrogen-doped-carbon to catalytically enable high energy and high power for the two-electron transfer I^+^/I^-^ electrode. *Adv. Mater.,* **36**, e2312246 (2024).

13	Zong, W. *et al.* Dynamical janus interface design for reversible and fast-charging zinc-iodine battery under extreme operating conditions. *J. Am. Chem. Soc.,* **146**, 21377-21388 (2024).

14	Jiang, P. *et al.* Energetic hypervalent organoiodine electrochemistry for aqueous zinc batteries. *J. Am. Chem. Soc.,* **146**, 25108-25117 (2024).

15	Wei, C. *et al.* A Universal strategy toward low‐cost aqueous sulfur-iodine batteries. *Adv. Funct. Mater.,* **33** (2023).

16	Liu, T. *et al.* Triflate anion chemistry for enhanced four-electron zinc-iodine aqueous batteries. *Chem. Commun.,* **60**, 7447-7450 (2024).

17	Liu, T. *et al.* Aqueous electrolyte with weak hydrogen bonds for four-electron zinc-iodine battery operates in a wide temperature range. *Adv. Mater.,* **36**, e2405473 (2024).

18	Zou, Y. *et al.* A four-electron Zn-I_2_ aqueous battery enabled by reversible I^-^/I_2_/I^+^ conversion. *Nat. Commun.,* **12**, 170 (2021).

19	Jiang, P. *et al.* Stabilized four-electron aqueous zinc-iodine batteries by quaternary ammonium complexation. *Chem. Sci.,* **15**, 3357-3364 (2024).

20	Liu, T. *et al.* Practical four-electron zinc-iodine aqueous batteries enabled by orbital hybridization induced adsorption-catalysis. *Sci. Bull.,* **69**, 1674-1685 (2024).

21	Li, W. *et al.* Designing ternary hydrated eutectic electrolyte capable of four-electron conversion for advanced Zn-I_2_ full batteries. *Energy Environ. Sci.,* **16**, 4502-4510 (2023).

22	Lv, S. *et al.* A High-performance quasi-solid-state aqueous zinc-dual halogen battery. *ACS Nano,* **16**, 20389-20399 (2022).

23	Yang, H. *et al.* Interfacial ion depletion at high concentration guides electrolyte design for aqueous aluminum-iodine batteries. *Joule,* **10**, 102502 (2026).

24	Ma, D. *et al.* Low‐concentration eutectic electrolyte enables four‐electron zinc‐iodine batteries. *Adv. Funct. Mater.,* **36**, e75378 (2026).

25	Zhao, Z. *et al.* Catalyst-electrolyte synergy enables ultrafast and long‐life two‐electron aqueous zinc-iodine batteries. *Angew. Chem. Int. Ed.,* **0**, e9322658 (2026).

26	Hu, Y. *et al.* Hydrophilic cation-mediated interfacial chemistry for advanced multi-electron Zn-halogen batteries. *ACS nano,* **20**, 23464-23476 (2026).

27	Chen, Y. *et al.* Tailoring coordination chemistry for robust four-electron redox in aqueous zinc-iodine pouch cells at low temperature. *ACS Energy Lett.,* **11**, 6361-6370 (2026).

28	Chen, Y. *et al.* Unveiling dual-interface coordination orchestration for durable aqueous zinc-iodine pouch cells with four-electron chemistry. *J. Am. Chem. Soc.,* **148**, 34958-34968 (2026).

29	Xu, Z. *et al.* Polyhalide ionic liquid phase-separation strategy enables high-performance four-electron transfer zinc-iodine batteries. *ACS nano,* **20**, 16180-16190 (2026).

30	Bao, X. *et al.* Hydrophobic quaternary ammonium iodides for highly stable four-electron zinc-iodine batteries. *Angew. Chem. Int. Ed.,* **0**, e8492354 (2026).

31	Wang, S. *et al.* Steric coordination modulated iodine chemistry with four‐electron conversion for zinc‐iodine batteries. *Adv. Mater.,* **38**, e73681 (2026).

32	Xu, J. *et al.* Ion‐replenishing interlayer and tailored electrolyte jointly activate four‐electron zinc-iodine batteries. *Adv. Mater.,* **38**, e73355 (2026).

33	Luo, X. *et al.* Synergistic effects of electrolyte additives in a dual‐salt system for high‐performance four electron aqueous zinc-iodine batteries across a wide temperature range. *Angew. Chem. Int. Ed.,* **137**, e202514375 (2025).

34	Dhir, S., Wheeler, S., Capone, I. & Pasta, M. Outlook on K-ion batteries. *Chem,* **6**, 2442-2460 (2020).

35	Liu, Y. *Industrialization of aqueous zinc-ion batteries: mechanism, material, device, industrial chain and commercialization perspectives*, UCL (University College London), (2024).
